# C(*sp^3^*)─S Bond Formation via the Synergy of Oxidative With Reductive Photocatalysts Through Photoredox and Dual Hydrogen Atom Transfer Processes

**DOI:** 10.1002/advs.202415936

**Published:** 2025-06-23

**Authors:** Chao Zhou, Qianli Liang, Xinyi Zhu, Xue Zhang, Limei Liu, Yicheng Zhang, Jie Liu, Xiaoyu Xie, Lei Wang

**Affiliations:** ^1^ College of Chemistry and Materials Science Key Laboratory of Green and Precise Synthetic Chemistry Ministry of Education Huaibei Normal University Huaibei Anhui 235000 P. R. China; ^2^ Advanced Research Institute and School of Pharmaceutical Sciences Taizhou University Taizhou Zhejiang 318000 P. R. China; ^3^ College of Material Chemistry and Chemical Engineering Key Laboratory of Organosilicon Chemistry and Material Technology Ministry of Education Hangzhou Normal University Hangzhou Zhejiang 311121 P. R. China

**Keywords:** C(sp^3^)–S bond formation, dual hydrogen atom transfer, dual‐catalytic system, Photoredox, polysulfide anions, tetrabutylammonium decatungstate

## Abstract

A primary objective of organic synthesis is to establish a catalytic methodology that is mild, straightforward, and economically efficient. Thioesters are widely employed in the realms of physiology, pharmacology, and agriculture. It is imperative to continuously expand the range of sulfur‐containing precursors to keep pace with the cutting‐edge advancements in the field of organic sulfur chemistry. This research has uncovered that polysulfide anions (K_2_S_x_), which serve as reducing catalysts, can also effectively act as sulfur reagents, providing the formation of C(*sp* ^3^)–S bonds through a photoredox catalysis with an oxidative photocatalyst tetrabutylammonium decatungstate (TBADT) and dual hydrogen atom transfer (DHAT) process. In a pioneering study, a combinatorial strategy of an oxidative photocatalyst TBADT is presented with a reductive photocatalyst K_2_S_x_, enabled a photo‐induced three‐component coupling reaction of simple aldehydes with alkanes containing C*(sp^3^*)─H and polysulfide anions. A numbers of thioester derivatives are successfully obtained in good yields, while a by‐product H_2_S is captured and identified by gas chromatography analysis. Concurrently, density functional theory (DFT) calculations provided the theoretical support of the reaction mechanism.

## Graphical abstract

The research reveals that polysulfide anions (K_2_S_x_), which serve as reducing catalysts, can also effectively act as sulfur reagents, providing the formation of C(*sp^3^*)─S bonds through photoredox catalysis with an oxidative photocatalyst tetrabutylammonium decatungstate (TBADT) and dual hydrogen atom transfer (DHAT) process.

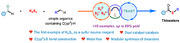

## 1 Introduction

**Figure 1:**
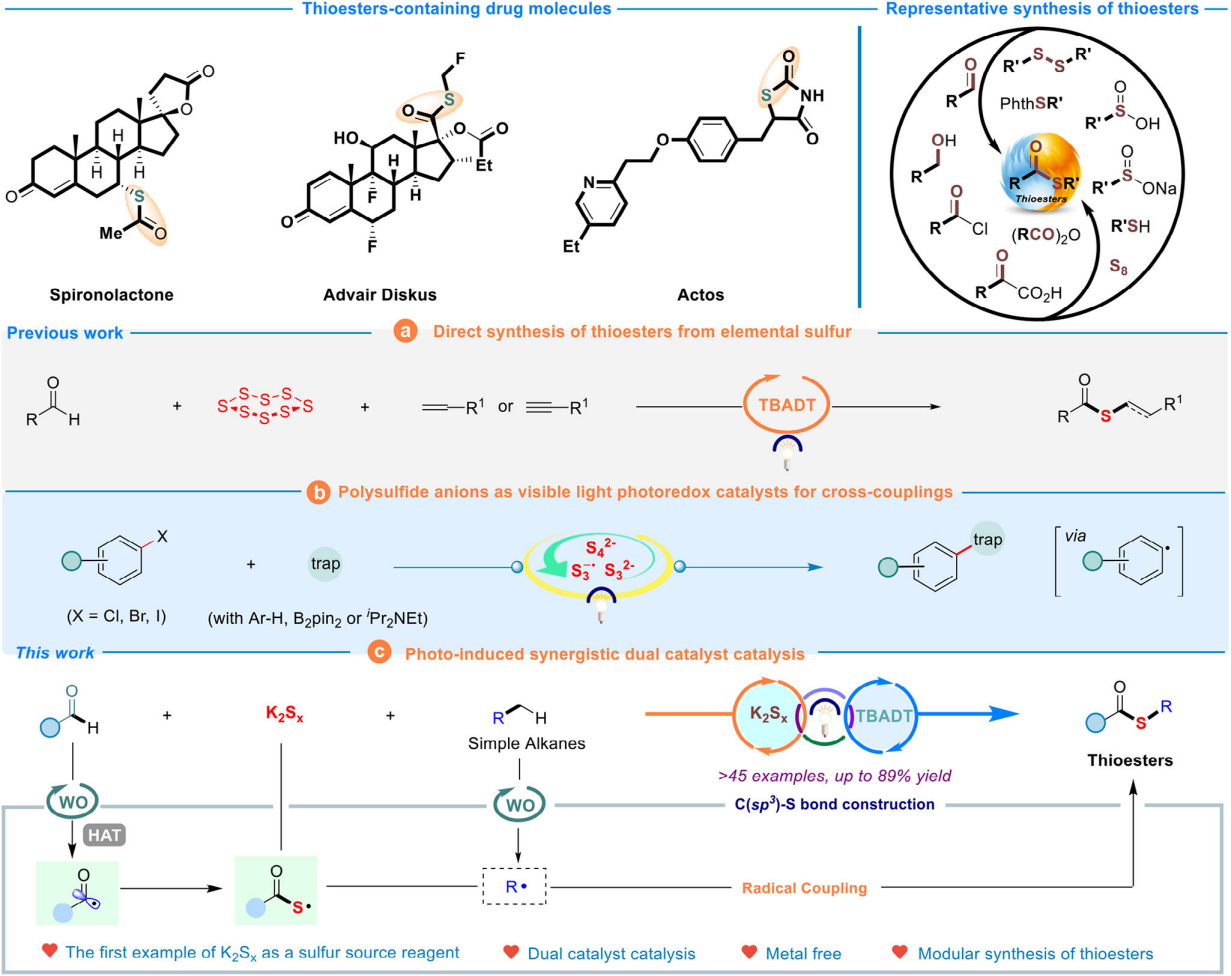
The background of this research and synthesis of thioesters by synergistic catalysis strategy.

The development of various pharmaceuticals has a profound impact on the well‐being of human society as a whole. According to statistics, seven of the ten best‐selling drugs in the United States were organic sulfur‐containing compounds (**Figure** **1**).^[^ ^1^ ^]^ It is the importance and utility of sulfur‐containing compounds^[^ ^2^ ^]^ that have driven the rapid development of strategies for constructing C(*sp^3^*)–S bond.^[^ ^3^ ^]^ Among the organosulfur compounds, the thioester represents a chemically labile and energy‐dense bond that is prevalent in many coenzymes (such as acetyl‐CoA) and plays a crucial role in myriad metabolic pathways in human physiology. Thioesters are widely used as a structural element in the structure of a large number of pharmacological drugs and are indispensability in molecular therapeutics. The intricate biological functions of thioesters, coupled with their extensive utility in the design and synthesis of drugs, place them at the forefront of chemical and biological research.^[^ ^4^ ^]^ So, developing a practical method for the preparation of thioester backbone from simple starting materials under mild and metal‐free conditions is highly desirable. The generation of acyl and sulfhydryl radicals through photocatalytic activation,^[^ ^5^ ^]^ or electrochemical catalysis,^[^ ^6^ ^]^ followed by the synthesis of thioester derivatives via a free radical coupling, represents one of the most extensively utilized methodologies in the literature (Figure 1).^[^ ^7^ ^]^ The group of Wu demonstrated that tetrabutylammonium decatungstate (TBADT) was able to photocatalyzed multicomponent coupling for the synthesis of thioesters using visible light (Figure 1a). It is worth mentioning that aldehyde derivatives and elemental sulfur, alkenyl/alkynyl compounds are synthesized as a series of products with pharmacological properties through a hydrogen atom transfer (HAT) process and radical polarity inversion.^[^ ^8^ ^]^ By means of the photoredox strategy, a series of thioesters^[^ ^9^ ^]^ have been developed in a rapid manner through the cross‐coupling of S sources^[^ ^10^ ^]^ and acyl radicals. For instance, Zhao group independently reported the use of sphosphoranyl radical mediated cleavage to produce acyl radicals coupled with diphenylene sulfide radicals to produce thioester compounds in high yields.^[^ ^11^ ^]^ It should be noted that Chiba group developed polysulfide anions (K_2_S_x_) as photo‐catalyst with strong single‐electron reductive ability in 2021 (Figure 1b),^[^ ^12^ ^]^ and achieved numbers of visible‐light‐induced organic transformations,^[^ ^13^ ^]^ which enriched reducing‐type photocatalysts.

The burgeoning development of the visible light sector in recent years has led to the synergistic catalytic reactions combining transition metals with photocatalysts being applied across a spectrum of diverse reaction types.^[^ ^14^ ^]^ By examining the principles underlying photoredox reactions, it becomes feasible to integrate two distinct categories of oxidative photocatalyst and reductive photocatalyst to synthesize thioester compounds through a synergistic catalytic approach. Consequently, we have independently employed TBADT as an oxidative photocatalyst^[^ ^15^ ^]^ and K_2_S_x_ as reductive photocatalyst, using polysulfide anions as sulfur source reagents to facilitate the formation of C(*sp^3^*)─S bonds via a radical coupling mechanism (Figure 1c). The innovative aspect of this methodology lies in the dual photocatalytic system comprising both oxidative and reductive photocatalysts, which synergistically enables multicomponent synthesis of thioester derivatives through a combination of SET and dual hydrogen atom transfer (DHAT) processes. In addition, aldehydes are typically more cost‐effective and widely accessible compared to thiocarboxylic acids. Notably, polysulfide anions exhibit bifunctional behavior in this reaction system‐serving a dual role as both a reductive photocatalyst and an effective sulfur source. This synergistic catalysis not only expands the synthetic strategies within the domain but also significantly broaden the range of sulfur precursors available for thioester synthesis, thereby providing a valuable experimental framework for organosulfur compound synthesis.

## 2 Optimization of Reaction Conditions

A multi‐component redox reaction of benzaldehyde (**1**), polysulfide anion (**2**, K_2_S_x_) as a sulfur source, and acetone (**3**) containing C(*sp^3^*)–H, as well as solvent was investigated under UV light irradiation at room temperature. After extensive optimization (**Table** **1**, see also the Supporting Information for more details), we were pleased to find that the desired thioester **4** was obtained in an isolated yield of 89% using tetrabutylammonium decatungstate (TBADT) as an effective photocatalyst in the presence of trifluoroacetic acid (TFA) (entry 1). Although moderate to low yields were obtained when other sulfur sources were involved in the reactions (entries 2−5), K_2_S_x_ with TFA (1.5 equiv.) stood out to afford product in excellent yield. Notably, it was found that adding TFA resulted in an enhancement of product yield greatly (entry 6). It may be attributed to the strong acidity of TFA, which activates the substrates including aldehyde and C─H coupling partner (acetone or acetonitrile). Moreover, the addition of inorganic acid such as TFA or acetic acid as an additive in the system can accelerate the liberation of H_2_S, promoting the chemical equilibrium, as well as the regeneration of the catalytic cycle. The controlled experiments demonstrate that UV light and photocatalyst are essential prerequisites for achieving successful and efficient conversion (entry 7). We also explored the efficacy of other commonly used photocatalysts, such as anthraquinone, Acr^+^‐Mes^−^, [Ir(dtbbpy)[dF(CF_3_)ppy]_2_]PF_6_ and Ru(bpy)_3_(PF_6_)_2_, were not as good as that of TBADT (entries 8−11). As for the reaction media, a reaction of acetone in 1.5 equiv. as a reactant with benzaldehyde (**1**), and polysulfide anions (**2**, K_2_S_x_) as sulfur source in acetonitrile as the solvent, providing a three‐component reaction product of benzaldehyde‐S‐acetonitrile **38** as the corresponding product. It is indicated that the concentration of C–H coupling partner used in the reaction plays an important role in this three‐component coupling reaction.

**Table 1:** Optimization of thioester synthesis from benzaldehyde with K_2_S_x_ ^a)^

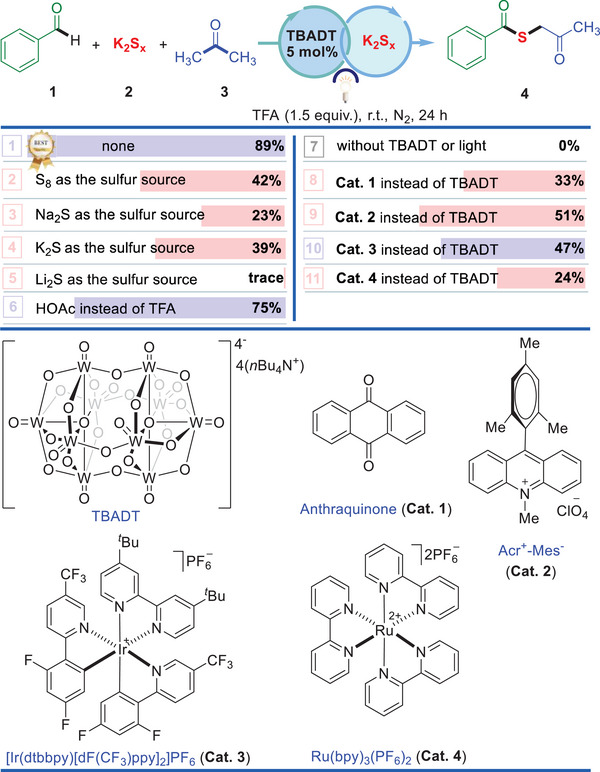

^a)^ Reaction conditions: benzaldehyde (**1**, 0.1 mmol), K_2_S_x_ (**2**, 0.1 mmol), acetone (**3**, 2.5 mL), TBADT (5.0 mol%), TFA (1.5 equiv.), N_2_, UV light (365 nm, 3 W) irradiation, rt, 24 h, isolated yield of the product.

## 3 Substrate Scope

With the optimized reaction conditions in hand, we next evaluated a range of aldehydes and their derivatives for the construction C(*sp^3^*)–S bonds with polysulfide anions (**Table** **2**). A wide array of benzaldehydes proved to be effective partners. For example, the electron‐donating functionalities (*para*‐Me, *para*‐Et, *para*‐*^i^* Pr, and *para*‐*^t^* Bu) were well tolerated and furnished the desired products **5**–**8** in 68%–73% yields. The reactions employing benzaldehydes with an electron‐withdrawing substitution (*para*‐F, *para*‐Cl, *para*‐CF_3_, and *para*‐CO_2_CH_3_) proceeded smoothly under the standard reaction conditions, providing the corresponding products **9**−**12** in moderate yields. Furthermore, the reactions of *meta*‐Me, *meta‐*MeO*, meta*‐F, *meta*‐Cl, *meta*‐Br, and *meta*‐CF_3_ substituted benzaldehydes reacted with **2** and acetone to afford the products **13**–**18** in 64−76% yields under the present reaction conditions. Then, several *ortho*‐substituted substrates were investigated, and the anticipated products **19**–**21** were obtained in 71–81% isolated yields. Meanwhile, disubstituted‐benzaldehydes worked well in current protocol, giving the target products **22**–**25** in moderate yields. Moreover, 1‐naphthyl and 2‐naphthyl also turned out to be pertinent substrates for this TBADT‐catalyzed reactions, generating the products **26**–**28** in good to high efficiency. Gladly, furan aldehyde and thiophenal derivatives also participated in the reactions and the products **29**–**32** could be obtained in 53–76% yields. It is also worth mentioning that it is not affected by the length of the aliphatic carbon chain, and the corresponding products **33**–**35** were isolated in 66–79% yields. Except aliphatic carbon chain, cyclopentanecarboxaldehyde and cyclohexanecarboxaldehyde also underwent the three‐component reaction with **2** and acetone to generate the desired products **36** and **37** in 82% and 71% yields, respectively.

**Table 2:** Preparation of thioesters^a)^

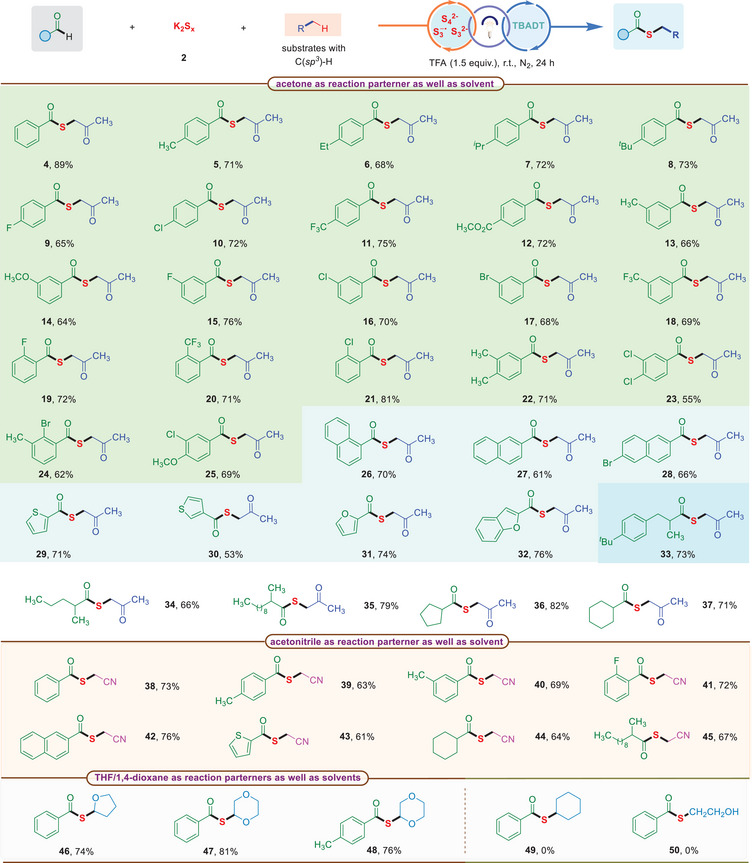

^a)^ Reaction conditions: aldehyde (0.1 mmol), K_2_S_x_ (**2**, 0.1 mmol), TBADT (5.0 mol%), TFA (1.5 equiv.), C(*sp^3^*)–H partner (2.5 mL) as well as solvent, N_2_, UV light (365 nm, 3 W) irradiation, rt, 24 h, isolated yield of the product.

Then, we are curious about whether aldehydes could still work for other multicomponents. Reaction substrates bearing diverse different solvents. To our delight, it proved successful, and acetonitrile was good coupling partner, enabling the production of **38** in 73% yield. Additionally, we investigated a variety of arylaldehydes bearing either an electron‐donating group (*para*‐Me or *meta*‐Me) or an electron‐withdrawing (*ortho*‐F) group, affording the corresponding products **39**−**41** in good yields (up to 72%). Notably, 2‐naphthaldehyde and thiophenal aldehydes reacted with **2** and acetonitrile, affording products **42**−**43** in good to high yields. The aliphatic aldehydes, such as cyclohexanecarboxaldehyde and 2‐methylundecanal were also attempted, giving the desired products **44** and **45** in 64% and 67% yields, respectively. Inspired by the aforementioned results, we have conducted reactions between benzaldehyde and alkane solvents, specifically tetrahydrofuran and 1,4‐dioxane, under standardized conditions to systematically assess the scope and compatibility of the reaction parameters involved. Interestingly, when tetrahydrofuran and 1,4‐dioxane were employed as solvents in this transformation, the desired products **46**−**48** were obtained with yields of 74%, 81% and 76%, respectively. It was observed that alternative alkane solvents, such as cyclohexane and ethanol, did not yield the desired products **49**−**50** in the multi‐component radical coupling reaction under established experimental conditions.

## 4 Mechanistic Studies

**Figure 2:**
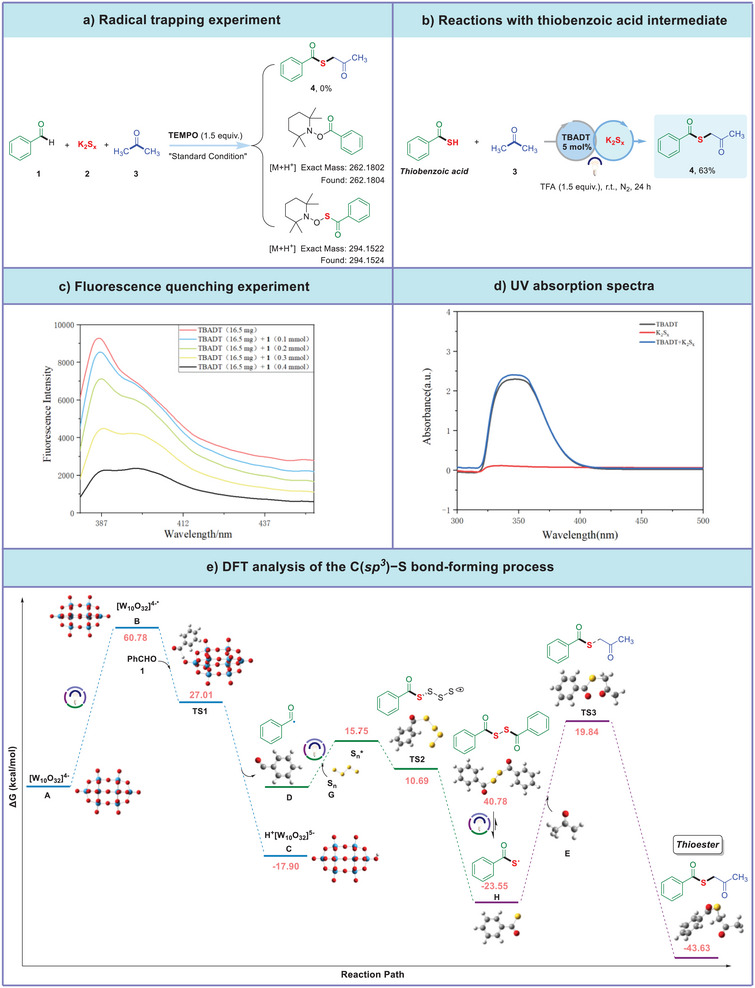
Mechanistic studies on the formation of thioester derivatives.

In order to gain insight into the possible mechanism of this UV light‐induced three‐component reaction, the following exploratory experiments were carried out (**Figure** **2**). When TEMPO (2,2,6,6‐tetramethyl‐1‐piperidinyloxy), a well‐known radical‐capturing reagent, was added into the reaction system under the standard conditions, the reaction was completely inhibited, while an adduct of TEMPO with benzoyl radical was formed, which was detected by HPLC‐HRMS analysis (see SI for detail), indicating that this transformation is involved in a free radical pathway (Figure 2a). Additionally, when thiobenzoic acid was employed to replace benzaldehyde, a comparable result was obtained (63% yield of **4**). It is reasoned that the thiobenzoic acid might be a reaction intermediate (Figure 2b). Subsequently, Stern‐Volmer fluorescence quenching experiments were conducted by mixing the photocatalyst TBADT with benzaldehyde. As shown in Figure 2c, the fluorescence emission intensity of TBADT in acetone was quenched more significantly with the increase of benzaldehyde. To gain further insight into the reaction mechanism, we conducted optical absorption measurements. As depicted in Figure 2d, the co‐mixing of acetonitrile solution containing TBADT and K_2_S_x_ resulted in a distinct bathochromic shift in the UV–vis absorption spectrum, which is characterized by pronounced absorption in the UV‐light region (325–375 nm). This spectral observation is indicative of a congruence between the experimental data and the wavelength range employed in the irradiation process. To elucidate the reaction mechanism of the dual‐catalytic system, quantum chemical computations were conducted employing density functional theory (DFT) methods (for further details, please refer to the Supporting Information). As shown in Figure 2e, benzaldehyde, under the catalysis of TBADT generates the benzoyl radical **D**, which then reacts with the intermediate S_n_, derived from potassium polysulfide, to form the benzoyl sulfide radical **H**. This radical **H** subsequently undergoes rapid coupling with the free radical **E**, leading to the formation of the final product. The synthesis of thioesters through this methodology has been validated by DFT computational simulations, with the obtained data, encompassing transition state intermediates, corroborating the proposed reaction mechanism.

**Figure 3:**
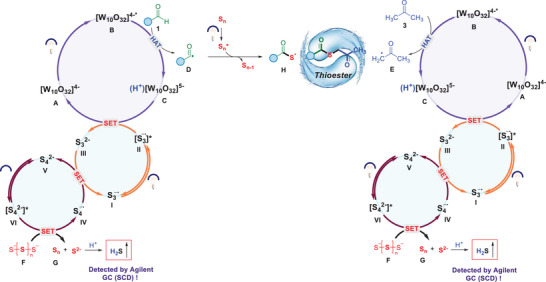
Plausible reaction mechanism.

Based on the experimental and theoretical studies, a plausible reaction mechanism was proposed (**Figure** **3**). At first, photoexcitation of decatungstate anion **A** ([W_10_O_32_]^4−^), followed by intersystem crossing, generates its excited state **B** (*[W_10_O_32_]^4−^), which abstracted a hydrogen atom from benzaldehyde **1** via a hydrogen atom transfer (HAT) process, generating a reducing photocatalyst **C** (H^+^[W_10_O_32_]^5−^) and benzoyl radical **D**, rather than abstracted a hydrogen atom from acetone via HAT process owing to the difference of bond dissociation energy (BDE) of C─H bond in benzaldehyde (88.7 ± 2.6 kcal/mol) and BDE of C─H bond in acetone (95.9 ± 0.7 kcal mol^−1^).^[^ ^16^ ^]^ Subsequently, with the formation of S_n_^*^, radical **D** was reduced to carbonylsulfide radical **H** via a single electron transfer pathway facilitated by **G** (polysulfide anions). In addition, the potential energy surface (PES) scanning calculations were carried out for disulfide bond cleavage under light irradiation (Supporting Information). The results indicated that the total energy of the system exhibited a significant downward trend while the S─S bond length of disulfide gradually increased. Based on the thermodynamic principle that “the lower of the energy, the more stable of the system,” it can be inferred that under light irradiation, it facilitates disulfide bond cleavage and drives the system toward a lower‐energy, more stable state. Therefore, the formation of carbonylsulfide radical **H** and the following the reaction to thioester product are favorable under photochemical conditions, which was supported by qualitative analysis of model reaction mixture with gas chromatography (qualitative results of time‐product/intermediate/substrate, shown in Table R2, Figures S7,S8 and S9 of SI for detail). Following the oxidation of acetone **3** with catalyst **B** (*[W_10_O_32_]^4−^) to form a corresponding radical **E** via a HAT process, it was then coupled with carbonylsulfide radical **H** to facilitate the formation of the target product thioester through a radical cross‐coupling reaction. Especially, **I** and **V** were excited their excited state **II** and **VI** under UV‐light irradiation, which has a higher reducing ability. Subsequently, the excited state [S_3_^−^]* and (H^+^[W_10_O_32_]^5−^) undergo a single electron transfer (SET) process that complete the catalytic cycle of TBADT while generating **III**. Meanwhile, **F** was reduced by **VI** to produce S_n_ and S^2−^ anion and combine with H^+^ to release hydrogen sulfide as gas, which was confirmed by an Agilent gas chromatography using sulfur chemiluminescence detector. Finally, after single electron transfer involving **IV** and **III**, K_2_S_x_ completes the catalytic cycle.

## 5 Conclusion

We have developed a multi‐component coupling method for an exceptionally efficient dual‐catalytic system to synthesize structurally diverse thioesters derivatives from commercially available aldehydes, polysulfide anions, and simple organics containing C(*sp^3^*)─H by direct photocatalysis and dual hydrogen atom transfer (DHAT) processes. The strategy that utilizes K_2_S_x_ as a precursor for sulfur‐based reagents and a reducing photocatalyst, in synergize with TBADT to catalyze the formation of C(*sp^3^*)–S bonds through free radical cross‐coupling reactions, exhibits significant innovation. Furthermore, methodologies for the synthesis of thioester derivatives, employing a strategy based on C(*sp^3^*)─S bonds, were developed and demonstrated functional group tolerance and broad substrate scope through a series of functionalized substrates. It is our contention that the establishment of a synergistic photocatalytic system for the synthesis of thioester compounds will pave the way for novel opportunities in the realms of organic synthesis, pharmaceuticals, and functional materials.

## Conflict of Interest

The authors declare no conflict of interest.

## Supporting information



Supporting Information

## Data Availability

The data that support the findings of this study are available from the corresponding author upon reasonable request.
