# Supplementary material for: C(sp3 )─S Bond Formation via the Synergy of Oxidative With Reductive Photocatalysts Through Photoredox and Dual Hydrogen Atom Transfer Processes
Source: Adv Sci (Weinh). 2025 Jun 23;12(35):e15936. doi: 10.1002/advs.202415936 (PMC12462969; doi:10.1002/advs.202415936)
Supplement: Supplementary file 1 — Supporting Information [file ADVS-12-e15936-s001.pdf]

ADVANCED SCIENCE
Open Access

Supporting Information

for *Adv. Sci.*, DOI 10.1002/advs.202415936

C(*sp*^{3})—S Bond Formation via the Synergy of Oxidative With Reductive Photocatalysts Through Photoredox and Dual Hydrogen Atom Transfer Processes

Chao Zhou, Qianli Liang, Xinyi Zhu, Xue Zhang, Limei Liu, Yicheng Zhang, Jie Liu, Xiaoyu Xie* and Lei Wang**

Supporting Information

C(sp^3)–S Bond Formation *via* The Synergy of Oxidative with Reductive Photocatalysts through Photoredox and Dual Hydrogen Atom Transfer Processes

Chao Zhou,[a] Qianli Liang,[a] Xinyi Zhu,[a] Xue Zhang,[a] Limei Liu,[a] Yicheng Zhang,*[,a] Jie Liu,[a] Xiaoyu Xie,*[,a] and Lei Wang*[,a,b,c]

[a]*College of Chemistry and Materials Science, Key Laboratory of Green and Precise Synthetic Chemistry, Ministry of Education, Huaibei Normal University, Huaibei, Anhui 235000, P. R. China*
[b]*Advanced Research Institute and School of Pharmaceutical Sciences, Taizhou University, Taizhou, Zhejiang 318000, P. R. China*
[c]*College of Material Chemistry and Chemical Engineering, Key Laboratory of Organosilicon Chemistry and Material Technology, Ministry of Education, Hangzhou Normal University, Hangzhou, Zhejiang, 311121, P. R. China*
Corresponding authors: E-mail: leiwang88@hotmail.com; lbqzhych@163.com; chemxie@163.com

Table of Contents

1. General Information 2
2. Experimental Section 3
3. Mechanistic Studies 5
4. Characterization Data for the Products 24
5. References 38
6. Copies of ^1H, ^{13}C NMR and ^{19}F NMR Spectra 38

1. General Information

The following starting materials were purchased from commercial suppliers. Analytical thin layer chromatography (TLC) was performed on precoated silica gel 60 F_{254} plates. Compounds were visualized by exposure to UV light. Flash column chromatography was performed with silica gel (300–400 meshes). NMR spectra were recorded on a 600 MHz spectrometer at ambient temperature, and chemical shifts were given in dimensionless δ values and were frequency referenced relative to TMS in ^{1}H, ^{13}C and ^{19}F NMR spectroscopy. The peak patterns are indicated as follows: **s**, singlet; **d**, doublet; **t**, triplet; **q**, quartet; **m**, multiplet; and **dd**, doublet of doublet. The coupling constants, *J*, are reported in Hertz (Hz). HRMS data were recorded on an Agilent Technologies 6540 UHD ESI-TOF mass spectrometer.

2. Experimental Section

2.1 Repsentative procedure for the catalytic three-component reaction

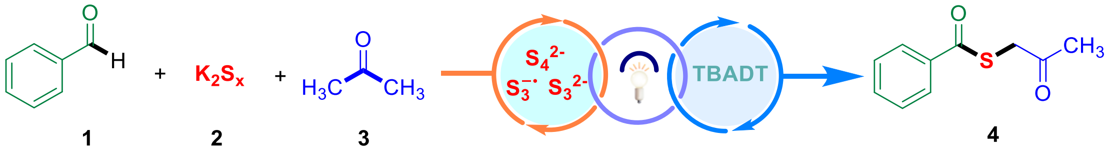

Polysulfide anions (K_2S_x) (**2**, 0.10 mmol) and TBADT (16.6 mg, 5 mol%) were added to an oven-dried reaction tube equipped with magnetic stirring bar (**Figure S1**). The benzaldehyde (**1**, 0.10 mmol), anhydrous acetone (**3**, 2.5 mL) and TFA (1.5 equiv.) were then added under nitrogen atmosphere. The tube was screw-capped and stirred at room temperature under irradiation of UV light (365 nm, 3 W) was placed 1.0 inches for 24 h. The product **4** as colorless oil was obtained by silica gel column chromatography (petroleum ether/ethyl acetate = 20:1).

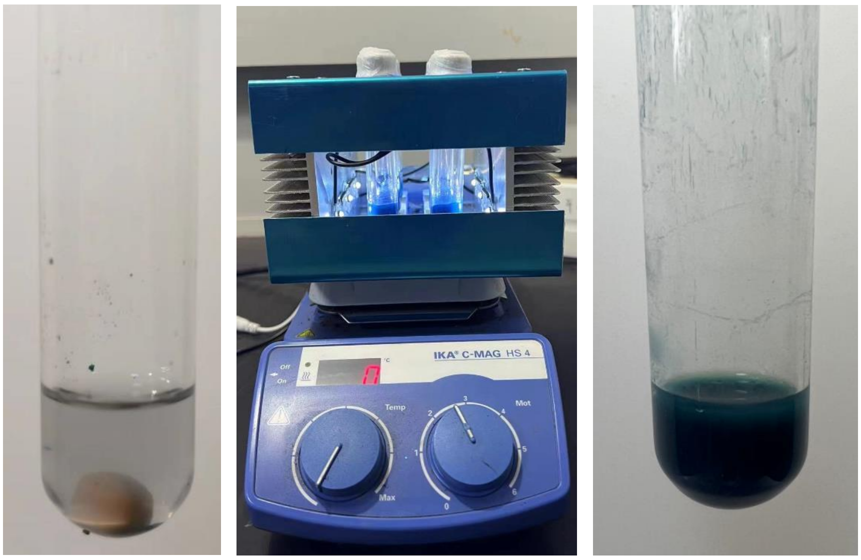

Figure S1. The photocatalysis setup

2.2 Further optimization of the reaction conditions[a] (Table R1)

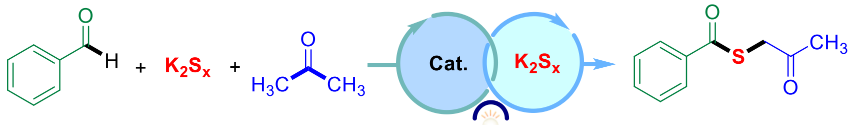

1 **2** **3** **4**

Entry	Catalyst	Additive	[S]	Light source	Yield (%)[b]
1	**Cat. 1**	TFA	K_2S_x	365 nm	33
2	**Cat. 2**	TFA	K_2S_x	365 nm	51
3	**Cat. 3**	TFA	K_2S_x	365 nm	47
4	**Cat. 4**	TFA	K_2S_x	365 nm	24
5	**Cat. 5**	TFA	K_2S_x	365 nm	<10
6	**Cat. 6**	TFA	K_2S_x	365 nm	29
7	TBADT	TFA	K_2S_x	365 nm	89
8	TBADT	HOAc	K_2S_x	365 nm	75
9	TBADT	Na_2CO_3	K_2S_x	365 nm	28
10	TBADT	$NaHCO_3$	K_2S_x	365 nm	36
11	TBADT	TFA	K_2S_x	390 nm	42
12	TBADT	TFA	K_2S_x	425 nm	26
13	TBADT	TFA	K_2S_x	450 nm	trace
14	TBADT	TFA	K_2S_x	520 nm	n.r.
15	TBADT	TFA	S_8	365 nm	42
16	TBADT	TFA	Na_2S	365 nm	23
17	TBADT	TFA	K_2S	365 nm	39
18	TBADT	TFA	Li_2S	365 nm	trace
19	–	TFA	K_2S_x	365 nm	0
20	TBADT	–	K_2S_x	365 nm	54
21	TBADT	TFA	–	365 nm	0
22	TBADT	TFA	K_2S_x	–	0
23	TBADT	TFA	K_2S_x	365 nm	58[c]

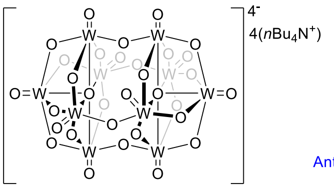

TBADT

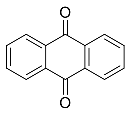

Anthraquinone (**Cat. 1**)

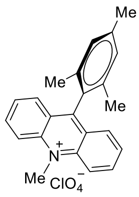

Acr^+-Mes^- (**Cat. 2**)

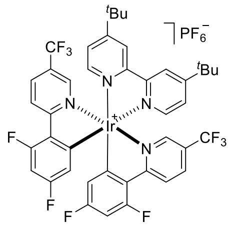

$[Ir(dtbbpy)[dF(CF_3)ppy]_2]PF_6$ (**Cat. 3**)

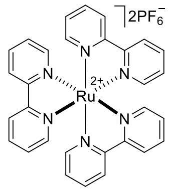

$Ru(bpy)_3(PF_6)_2$ (**Cat. 4**)

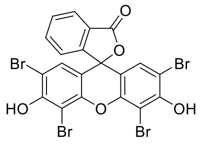

Acid Red 87 (**Cat. 5**)

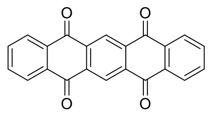

Pentacenetetrone (**Cat. 6**)

[a]Reaction conditions: **1** (0.10 mmol), **2** (0.1 mmol), catalyst (5 mol%), additive (1.5 equiv) and acetone (**3**, 2.5 mL), r.t., N_2, under light irradiation for 24 h. [b]Isolated yield. [c]Under air.

3. Mechanistic studies

3.1 The radical inhibiting and capturing experiment

A 15 mL oven-dried reaction tube equipped with a magnetic stirrer bar was charged with benzaldehyde (**1**, 0.10 mmol), K_2S_x (**2**, 0.10 mmol), TBADT (16.6 mg, 5 mol%), 2,2,6,6-tetramethyl-1-piperinedinyloxy (TEMPO, 23.4 mg, 0.15 mmol), acetone (**3**, 2.5 mL) and TFA (1.5 equiv.). The reaction mixture was exposed to UV light (365 nm, 3 W) irradiation under nitrogen at room temperature stirring for 24 h. In the reaction mixture, no desired product **4** was detected, while adducts of TEMPO with benzoyl radical and thioester radical were formed, which were detected by HPLC/HRMS analysis (**Figure S2**).

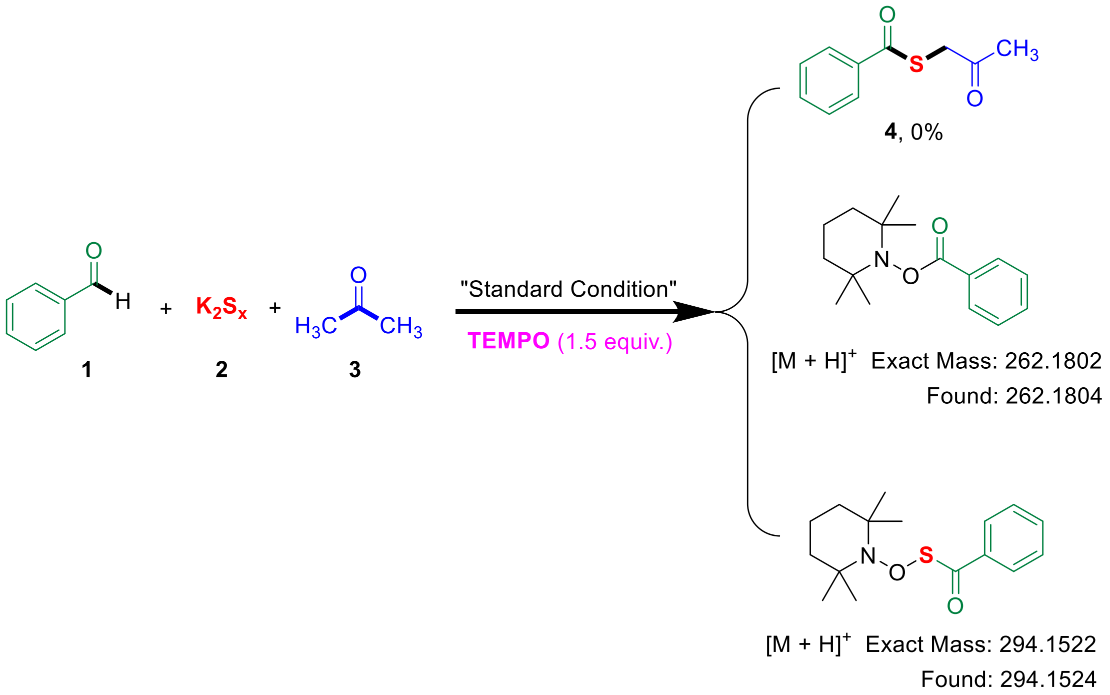

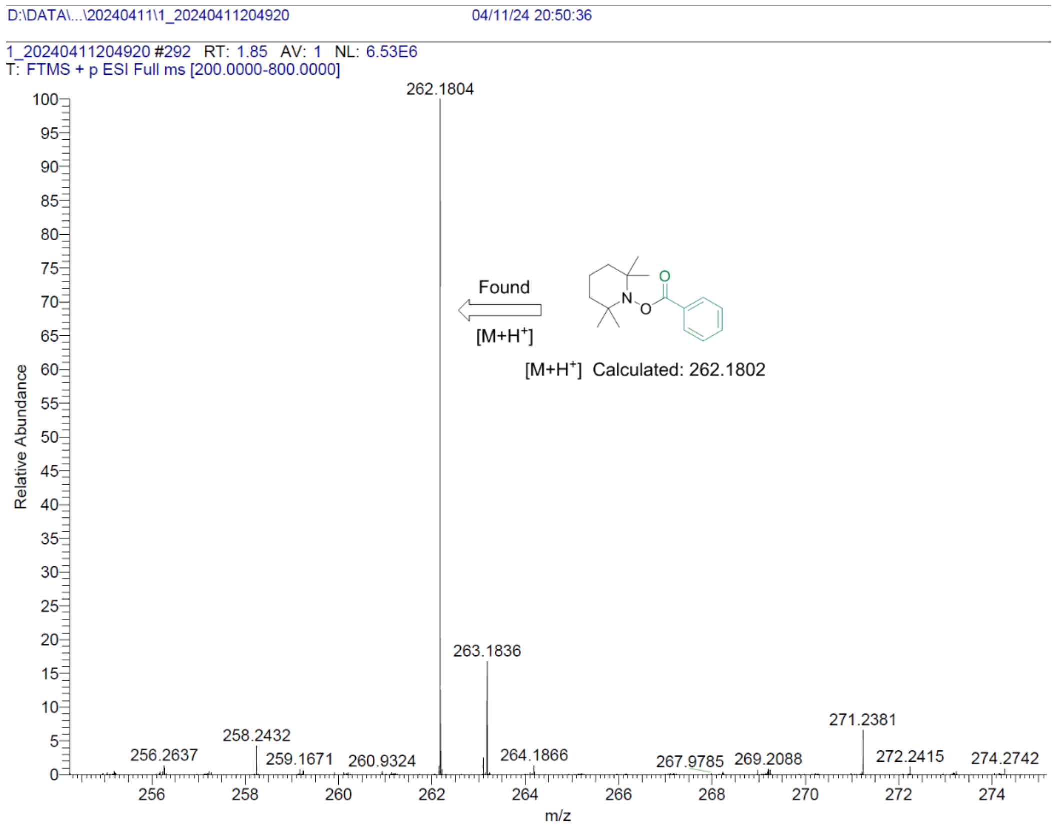

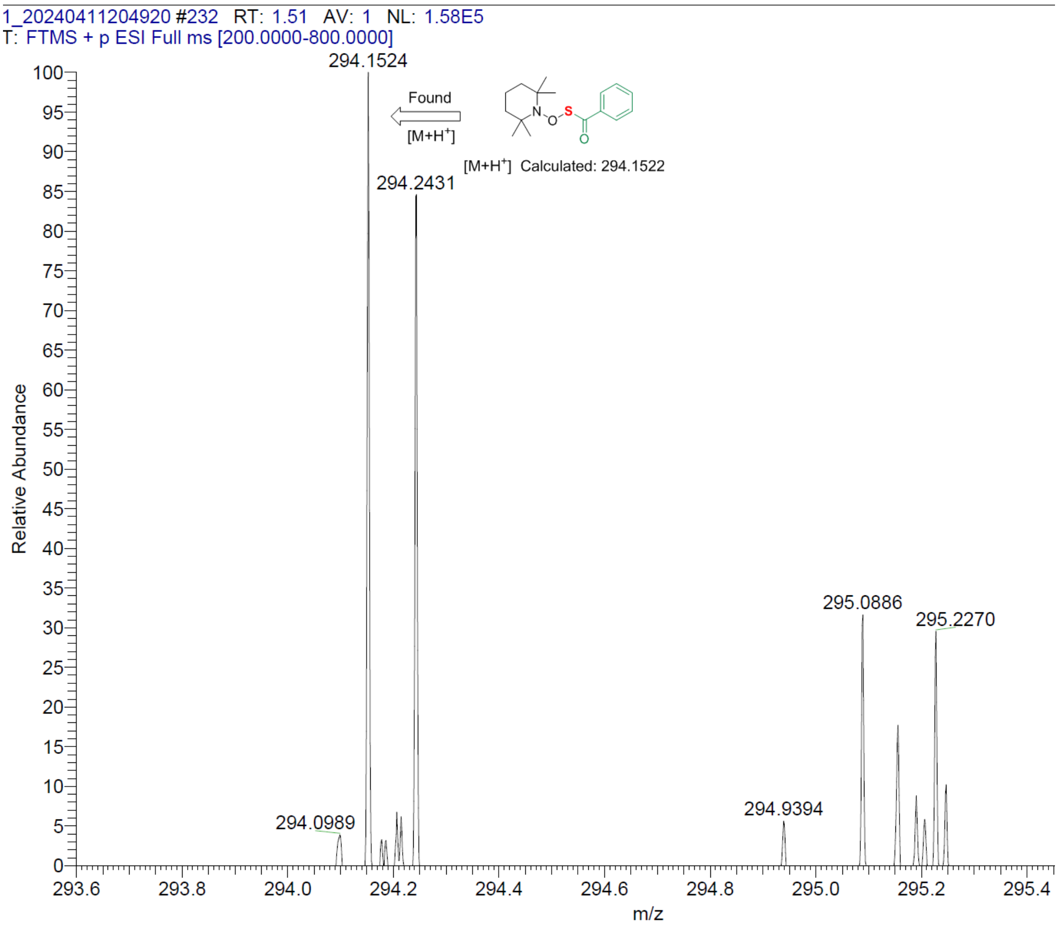

Figure S2. HRMS analysis of adducts of radical intermediate with TEMPO

3.2 Reaction of thiobenzoic acid with acetone

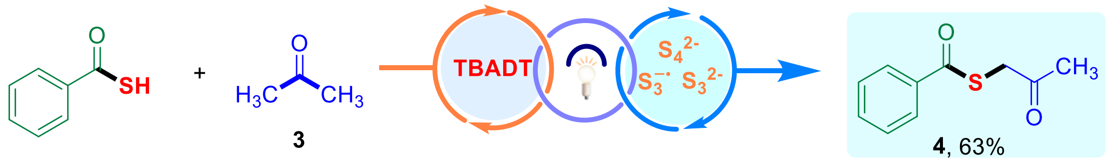

TBADT (5 mol%, 16.6 mg) was added to an oven-dried screw-top pressure reaction tube equipped with a magnetic stirring bar. Acetone (**3**, 2.5 mL), TFA (1.5 equiv.) and thiobenzoic acid (0.1 mmol) were then added under nitrogen atmosphere. The reaction mixture was exposed to UV light (365 nm, 3 W) irradiation at room temperature stirring for 24 h. The reaction mixture was purified by silica gel column chromatography (petroleum ether/ethyl acetate = 20: 1) to give the desired product **4** in 63% yield as a colorless oil.

3.3 Fluorescence quenching experiment of TBADT with 1

Stern-Volmer fluorescence quenching experiment was conducted *via* adding the appropriate amount of benzaldehyde (**1**, 0.1–0.4 mmol, respectively) and solution of TBADT in dry acetone in a reaction tube at room temperature under N_2 atmosphere. the sample was irradiated at 365 nm and the fluorescence was measured from 360 nm to 450 nm (**Figure S3**).

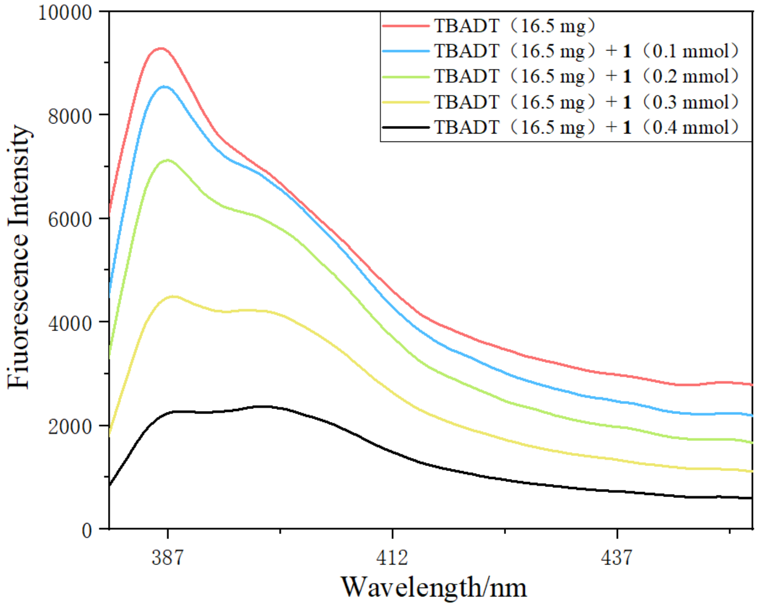

Figure S3. Fluorescence quenching experiment of TBADT with **1** in acetone

3.4 UV absorption spectra

The UV absorption spectra of TBADT (5 mol%, 16.6 mg), and K_2S_x (**2**, 0.10 mmol) in acetone were recorded in 1.0 cm path quartz cuvettes by using a Thermo Nanodrop 2000 UV spectrometer, respectively. The obtained charge-transfer bands in UV absorption spectra were shown in **Figure S4**.

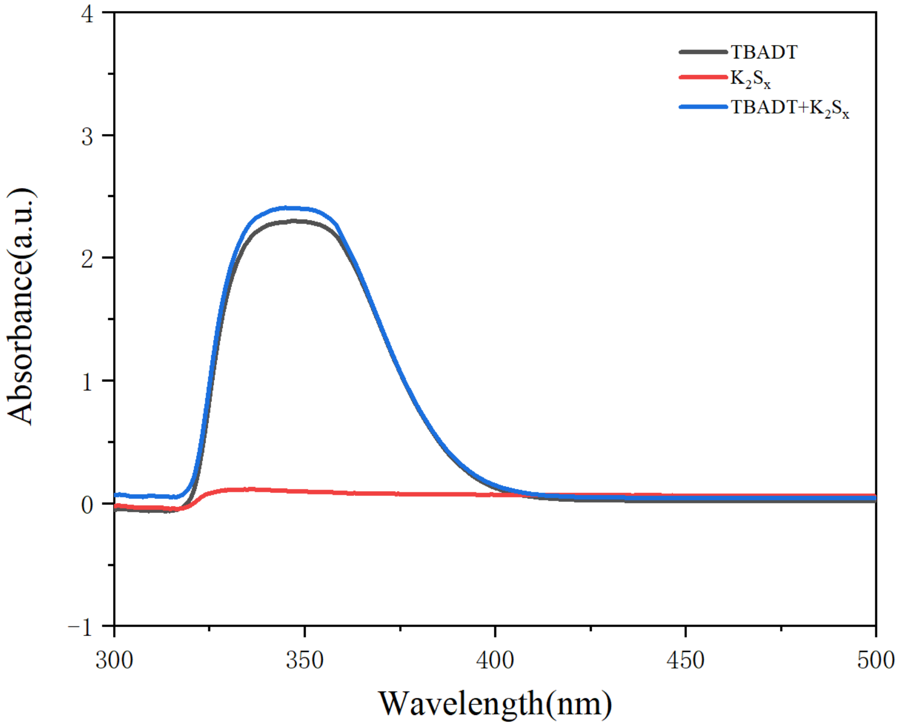

Figure S4. UV absorption spectra of TBADT, K_2S_x (**2**), and the interaction between TBADT and **2**

3.5 The detection of H_2S by gas chromatography

A 15 mL oven-dried reaction tube equipped with a magnetic stirrer bar was charged with benzaldehyde (**1**, 0.10 mmol), K_2S_x (**2**, 0.10 mmol), TBADT (16.6 mg, 5 mol%), acetone (**3**, 2.5 mL) and TFA (1.5 equiv). The reaction mixture was exposed to UV light (365 nm, 3 W) irradiation under nitrogen at room temperature stirring for 24 h. After completion of the reaction, the resulting gas from the reaction mixture was directly determined by Agilent gas chromatography analysis, and the concentration of H_2S was found to be 340 ppm (**Figure S5**).

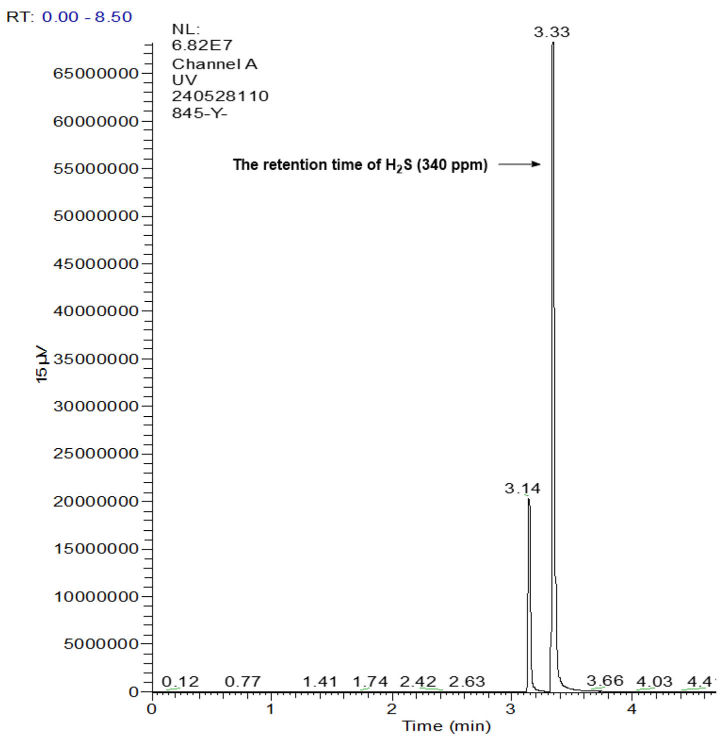

Figure S5. Analysis of the resulting H_2S gas by gas chromatography (SCD)

3.6 Potential energy surface (PES) scanning of disulfide

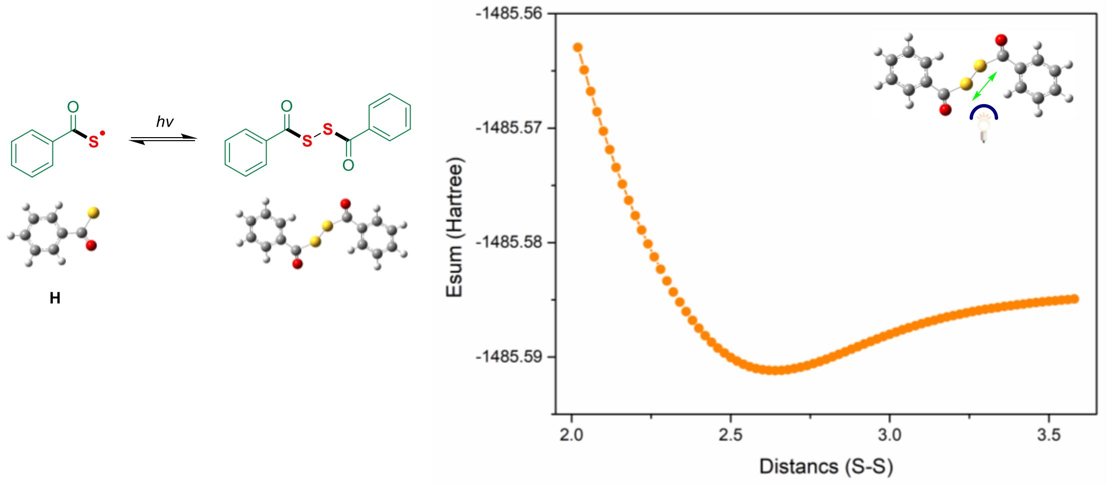

Figure S6. Potential energy surface (PES) scanning calculations of disulfide

3.7 Qualitative analysis of model reaction mixture by gas chromatography

To gain a clearer understanding of the reaction progression, six parallel reactions were run at varying time intervals. Upon completion of the reactions, the mixtures were filtered and subjected to gas chromatography (GC-2010 Pro) analysis to qualitatively assess the crude yields of starting material (**1**), intermediate disulfide,

and product (**4**). Experimental results showed that the intermediate disulfide was not detected in the reactions at different reaction time (5 h, 10 h, 15 h, 18 h, 20 h, and 24 h, **Table R2**). The amount of desired product **4** was increased and the amount of starting material **1** was decreased along with the reaction time increased (**Figure S7**, **Figure S8** and **Figure S9**). The corresponding benzoyl sulfide radical **H** generated in the reaction undergoes rapid a coupling with the free radical **E**, leading to the formation of the final product (**4**), which is also supported by DFT calculation, shown in Figure 2 of main text.

Table R2. Analysis of the reaction mixture along with reaction time[a]

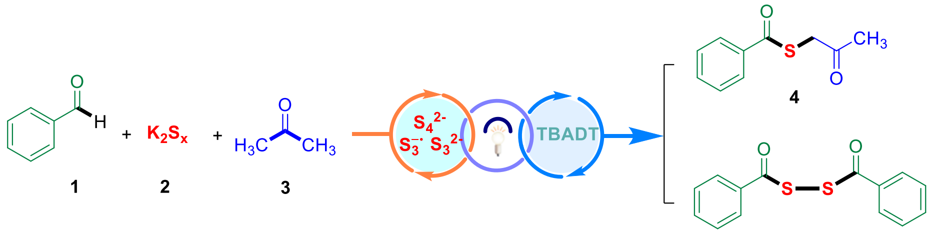

Entry	Time (h)	Recovery of **1** (%)[b]	Yield of **4** (%)[b]	Yield of disulfide (%)[b]
1	5	87	13	N. D.[c]
2	10	72	27	N. D.
3	15	53	46	N. D.
4	18	30	69	N. D.
5	20	24	76	N. D.
6	24	5	95	N. D.

[a]Reaction conditions: **1** (0.10 mmol), **2** (0.1 mmol), TBADT (5 mol%), TFA (1.5 equiv.) and acetone (**3**, 2.5 mL), r.t., N_2, under light irradiation. [b]Determined by GC analysis. [c]N. D = Not detected.

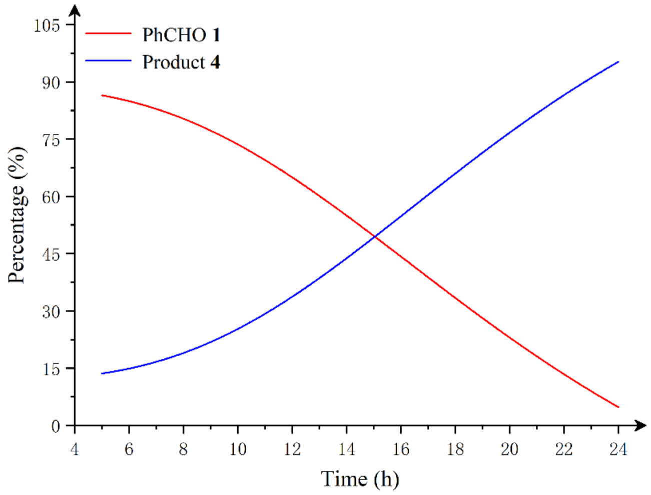

Figure S7. The recovery of PhCHO **1** (red) and obtained product **4** (blue) with reaction time.

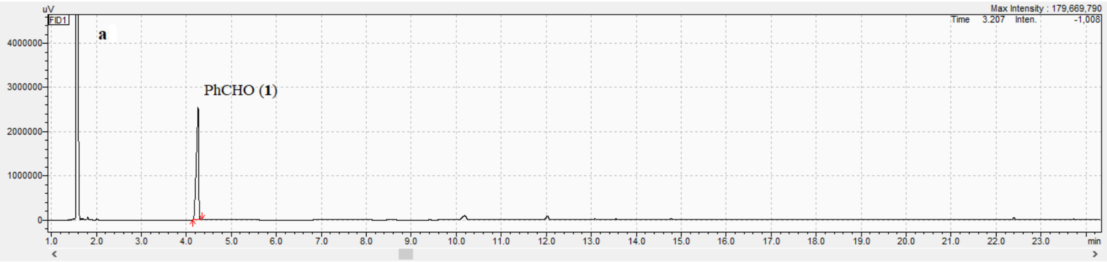

结果视图 - 峰表

峰表 化合物 组 校准曲线

峰号	保留时间	面积	高度	标记	面积%
1	4.262	8989072	2527893	M	100.000
总计		8989072	2527893		100.000

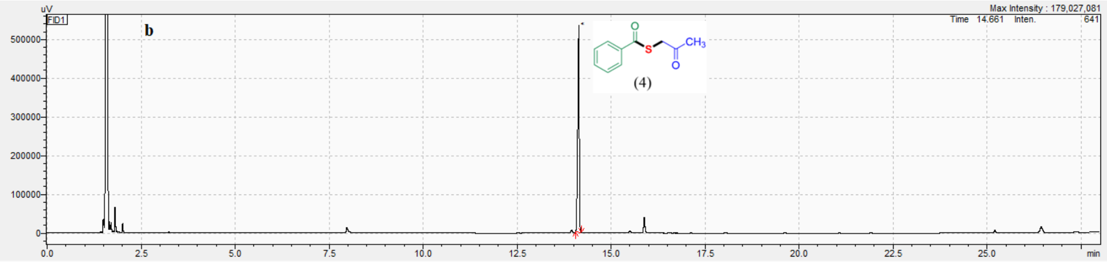

结果视图 - 峰表

峰表 化合物 组 校准曲线

峰号	保留时间	面积	高度	标记	浓度	面积%
1	14.141	1514193	524093	M	0.000	100.000
总计		1514193	524093		0.000	100.000

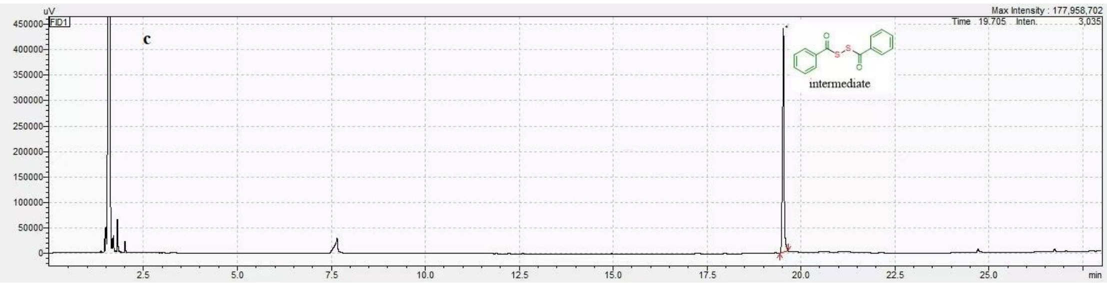

结果视图 - 峰表

峰表 化合物 组 校准曲线

峰号	保留时间	面积	高度	标记	浓度	面积%
1	19.533	1303850	437728	M	0.000	100.000
总计		1303850	437728		0.000	100.000

Figure S8. GC analysis of standard samples [**a:** PhCHO **1** (4.26 sec.), **b:** product **4** (14.14 sec.), **c:** intermediate disulfide (19.52 sec.)]

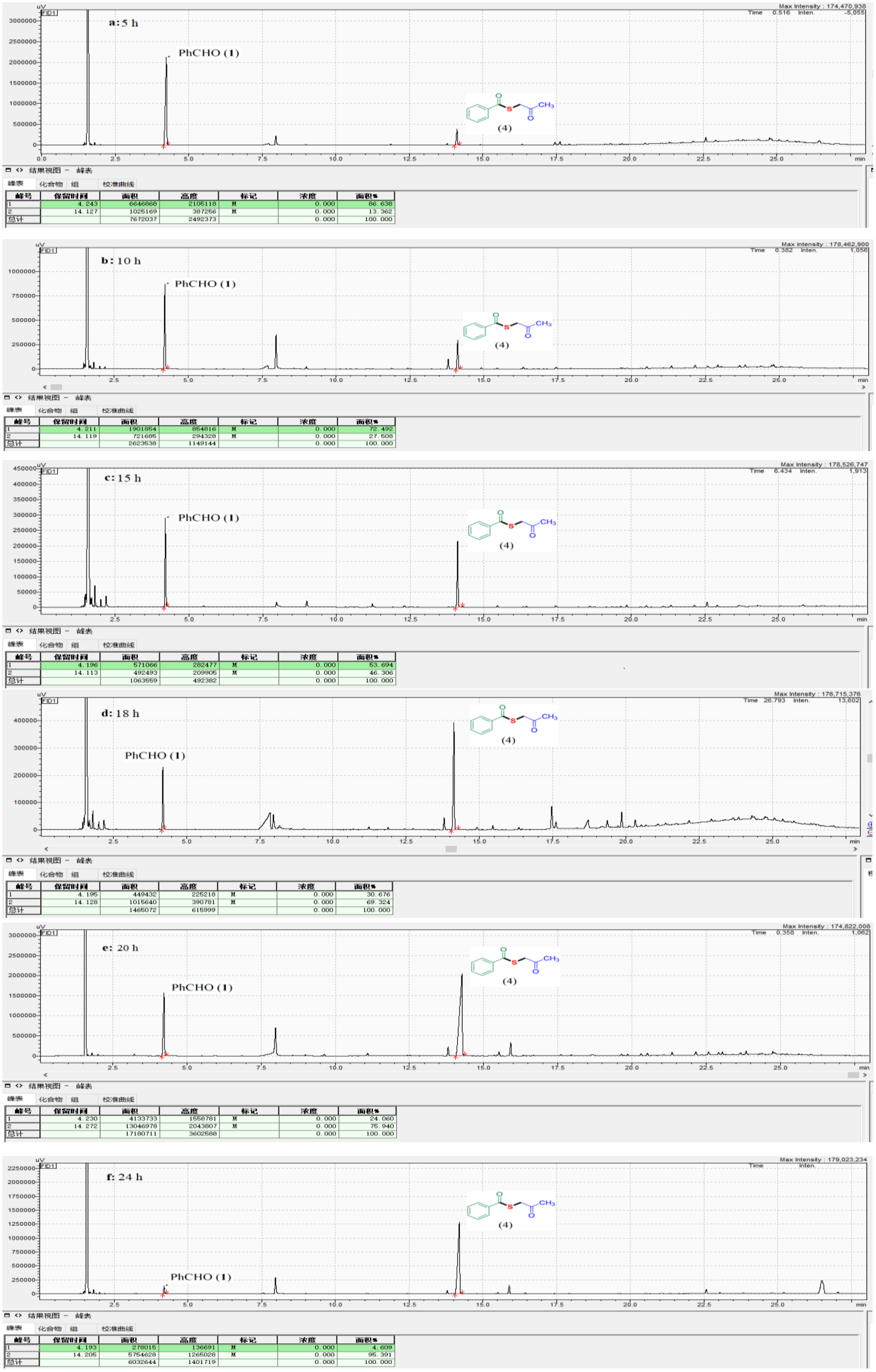

Figure S9. GC analysis of reaction mixture at different time (**a:** 5 h, **b:** 10 h, **c:** 15 h, **d:** 18 h, **e:** 20 h, **f:** 24 h).

3.8 Data related to DFT (density functional theory) theoretical calculations

Name	$[W_{10}O_{32}]^{4-}$		
charge	-4	multiplicity	1
level of opt &freq	b3lyp/def2svp, PCM (Acetonitrile)		
electronic energy	-3079.489372 a.u.		
Temperature	298.15K		
Zero point energy (ZEP)	0.167850 a.u.		
Thermal correction to U	0.168290 a.u.		
Thermal correction to H	0.1638735 a.u.		
Thermal correction to G	0.096321 a.u.		
level of sp	b3lyp/def2tzvp, SMD (Acetonitrile)		
Sum of electronic energy and thermal correction to G: -3079.659096 a.u.			

Coordinate:

W	-1.91597100	-0.18412700	-2.34253400
W	-4.28909400	0.00854000	0.00725000
W	-1.90144600	0.18573800	2.34114500
W	-1.90170100	2.34133400	-0.18470900
W	-1.91654100	-2.34490500	0.18490700
O	-2.12965400	-0.31667700	-4.04353800
O	-3.80608700	1.86311500	-0.14460900
O	-3.80415400	-0.14344800	-1.86000800
O	-1.94831700	0.00739700	0.00426200
O	-2.12910500	4.04186800	-0.31612000
O	-6.01753400	0.00587700	0.00267500
O	-3.80597200	0.14025600	1.86273600
O	-2.13133600	-4.04634600	0.31705100
O	-1.89002300	-2.00924200	-1.71957500
O	-1.89826300	2.01139700	1.72327000
O	-0.00131700	-0.17750700	-2.27879100
O	0.00157300	2.28184500	-0.17400800
O	-3.80456900	-1.85977500	0.13953300
O	-1.89570800	-1.72111300	2.00808300
O	-1.89431900	1.72051900	-2.01270900
O	-2.12835800	0.31400700	4.04080600
W	1.91617500	0.18413200	2.34258300
W	4.28907800	-0.00855500	-0.00722000
W	1.90167100	-0.18579200	-2.34125600
W	1.90151300	-2.34123500	0.18473500
W	1.91633700	2.34487100	-0.18490000
O	2.12974800	0.31673900	4.04359500
O	3.80598900	-1.86307900	0.14447300
O	3.80436600	0.14340500	1.86010200

O	1.94832900	-0.00746200	-0.00404800
O	2.12895300	-4.04178700	0.31609900
O	6.01753900	-0.00591400	-0.00285100
O	3.80588800	-0.14025300	-1.86287000
O	2.13141800	4.04627000	-0.31705000
O	1.88999700	2.00918400	1.71961600
O	1.89821500	-2.01135200	-1.72331000
O	0.00132200	0.17750800	2.27886300
O	-0.00166500	-2.28169600	0.17406700
O	3.80447000	1.85963200	-0.13955700
O	1.89564500	1.72104600	-2.00801200
O	1.89439000	-1.72050200	2.01279100
O	2.12832800	-0.31392800	-4.04097400

Name	$[W_{10}O_{32}]^{4-*}$		
charge	-4	multiplicity	3
level of opt &freq	b3lyp/def2svp, PCM (Acetonitrile)		
electronic energy	-3079.550361 a.u.		
Temperature	298.15K		
Zero point energy (ZEP)	0.170370 a.u.		
Thermal correction to U	0.167900 a.u.		
Thermal correction to H	0.167842 a.u.		
Thermal correction to G	0.096364 a.u.		
level of sp	b3lyp/def2tzvp, SMD (Acetonitrile)		
Sum of electronic energy and thermal correction to G: -3079.56223091 a.u.			

Coordinate:

W	1.89475100	-2.40623900	0.21553200
W	4.40882300	-0.00110300	-0.00056000
W	1.89750300	2.40608800	-0.21644200
W	1.89741500	0.21579200	2.40616100
W	1.89465900	-0.21613200	-2.40607400
O	2.15850400	-4.09661000	0.36653400
O	3.79151600	0.16180400	1.80232300
O	3.79043500	-1.80280500	0.16179400
O	1.73759700	-0.00124500	-0.00149900
O	2.16058700	0.36688900	4.09657600
O	6.13556000	-0.00253900	-0.00223700
O	3.79120700	1.80217300	-0.16199000
O	2.15887000	-0.36738900	-4.09636300
O	1.90962800	-2.01134100	-1.68141600
O	1.90988700	2.01020900	1.68043000
O	0.01235800	-2.31004700	0.20401000
O	0.01397200	0.20391200	2.30877900

O	3.78993700	-0.16187900	-1.80245400
O	1.90958000	1.68091000	-2.01129600
O	1.90981400	-1.68101500	2.01033100
O	2.16078600	4.09648900	-0.36738300
W	-1.91977500	2.34355700	-0.20706100
W	-4.29930200	0.00193500	0.00168300
W	-1.92298400	-2.34358700	0.20770100
W	-1.92306000	-0.20728700	-2.34375200
W	-1.91981800	0.20745600	2.34318500
O	-2.13832400	4.04365000	-0.35715100
O	-3.82672700	-0.16571400	-1.86198300
O	-3.82654100	1.86364700	-0.16563900
O	-1.95248800	0.00058700	0.00093000
O	-2.14177200	-0.35728400	-4.04386500
O	-6.03047200	0.00153800	0.00164500
O	-3.82670000	-1.86226400	0.16554700
O	-2.13810800	0.35737700	4.04334700
O	-1.89850100	2.02429300	1.69491800
O	-1.89804300	-2.02357400	-1.69435000
O	0.01391300	2.30903000	-0.20415900
O	0.01223400	-0.20419500	-2.31005400
O	-3.82658600	0.16543900	1.86389100
O	-1.89809100	-1.69501900	2.02402400
O	-1.89838300	1.69430500	-2.02381900
O	-2.14161300	-4.04376900	0.35712700

Name	**TS1**		
charge	-4	multiplicity	1
level of opt &freq	b3lyp/def2svp, PCM (Acetonitrile)		
electronic energy	-3424.940856 a.u.		
Temperature	298.15K		
Zero point energy (ZEP)	0.170381 a.u.		
Thermal correction to U	0.17002700 a.u.		
Thermal correction to H	0.169026 a.u.		
Thermal correction to G	0.106730 a.u.		
level of sp	b3lyp/def2tzvp, SMD (Acetonitrile)		
Sum of electronic energy and thermal correction to G: -3424.95019 a.u.			

Coordinate:

W	-1.78243600	0.57557500	2.08471900
W	-3.78860100	-1.17238300	0.05598500
W	-1.24163800	-1.76470300	-2.00626300
W	-0.99673800	-2.55991900	1.22549700
W	-2.00365800	1.39209700	-1.15320800

O	-2.20409800	1.36650500	3.55345600
O	-3.02609000	-2.64642300	0.91867200
O	-3.55990200	-0.21118100	1.70559700
O	-1.63734900	-0.65982700	0.03890000
O	-0.92887500	-4.04995300	2.08513600
O	-5.65384100	-1.40245000	0.08163700
O	-3.22343500	-1.89145700	-1.58961700
O	-2.60705700	2.76884800	-1.98715700
O	-2.09178400	1.87273100	0.74557000
O	-0.95267700	-3.08470000	-0.67476700
O	0.10177100	0.85924000	2.05202200
O	0.82464900	-2.15442000	1.05481100
O	-3.76230000	0.52850000	-0.78929400
O	-1.72126700	0.07859100	-2.49902600
O	-1.32115900	-1.27994500	2.57719500
O	-1.32657800	-2.65294000	-3.47776400
W	2.40288500	-0.64637700	-2.17989700
W	4.50473500	0.84908200	-0.04829600
W	1.98098200	1.19459500	2.11700200
W	1.67355600	2.36442200	-0.97933500
W	2.73250900	-1.81574500	0.91671200
O	2.73424600	-1.29127500	-3.74147500
O	3.65346300	2.34440600	-0.89957700
O	4.22178200	-0.10756300	-1.72245200
O	2.22824000	0.28699500	-0.02426000
O	1.51020400	3.92714900	-1.68365000
O	6.19073900	1.24484900	-0.07621700
O	3.88056600	1.53184600	1.62506100
O	3.29479100	-3.28653000	1.61414700
O	2.74139600	-2.15046400	-0.94028400
O	1.62326800	2.69105600	0.88234900
O	0.55108700	-1.19716100	-2.03328000
O	-0.15904600	1.81144700	-1.04194000
O	4.45318400	-0.91209900	0.78091000
O	2.42919600	-0.61552300	2.45232700
O	1.93453700	1.15027800	-2.51268400
O	2.06285300	1.93336400	3.67083000
C	-6.31248900	2.33620900	-0.10888100
C	-6.98119900	1.10951500	-0.55962400
O	-7.82153000	0.94932400	-1.38787400
C	-5.39883900	2.29569700	0.95419700
H	-5.20071000	1.36255500	1.47608600
C	-4.70139200	3.44764900	1.31243000
H	-3.97831100	3.40440200	2.12830600

C	-4.91487000	4.63632000	0.61080100
H	-4.35629800	5.53536900	0.88183200
C	-5.83701200	4.68572600	-0.44583100
H	-5.99680200	5.61927600	-0.98973700
C	-6.53906000	3.54049600	-0.80850400
H	-7.24694100	3.55283300	-1.63960300
H	-6.26175300	-0.62535300	-0.07866800

Name	$H^{+}[W_{10}O_{32}]^{5-}$		
charge	-3	multiplicity	1
level of opt &freq	b3lyp/def2svp, PCM (Acetonitrile)		
electronic energy	-3079.934901 a.u.		
Temperature	298.15K		
Zero point energy (ZEP)	0.180371 a.u.		
Thermal correction to U	0.180103 a.u.		
Thermal correction to H	0.178856 a.u.		
Thermal correction to G	0.126362 a.u.		
level of sp	b3lyp/def2tzvp, SMD (Acetonitrile)		
Sum of electronic energy and thermal correction to G: -3079.94484169 a.u.			

Coordinate:

W	-1.91960200	-0.60558900	-2.27184700
W	-4.29546500	0.00659100	0.00209900
W	-1.91053200	0.60481500	2.26783400
W	-1.90944200	2.26833000	-0.60605800
W	-1.92151600	-2.27391400	0.60454100
O	-2.13130500	-1.04361300	-3.92107700
O	-3.81354600	1.80358500	-0.48202300
O	-3.80834700	-0.47999700	-1.80645500
O	-1.95468500	0.00695800	0.00163800
O	-2.13785200	3.91690000	-1.04291300
O	-6.02389600	0.00164600	-0.00359600
O	-3.81449400	0.47195300	1.80367900
O	-2.13530400	-3.92362700	1.04185300
O	-1.89310900	-2.28801000	-1.32921700
O	-1.90790000	2.28871600	1.33013400
O	-0.00502500	-0.58589400	-2.20849300
O	-0.00614100	2.21340600	-0.58293300
O	-3.80982000	-1.80661500	0.47039000
O	-1.90313100	-1.33082300	2.28494700
O	-1.89960800	1.32732000	-2.29171800
O	-2.13941900	1.03800400	3.91609000
W	1.90708500	0.60680800	2.27324200
W	4.28272800	-0.00539500	-0.00071900

W	1.89803600	-0.60368300	-2.26658500
W	1.89653300	-2.26702300	0.60741300
W	1.90859100	2.27508700	-0.60318000
O	2.11867800	1.04489000	3.92247000
O	3.80072800	-1.80236900	0.48323100
O	3.79583800	0.48117700	1.80790300
O	1.94197700	-0.00577800	-0.00006800
O	2.12498000	-3.91561900	1.04422600
O	6.01118100	-0.00050900	0.00477800
O	3.80168900	-0.47076900	-1.80246300
O	2.12266500	3.92475800	-1.04049000
O	1.88036200	2.28916600	1.33061600
O	1.89513100	-2.28747400	-1.32883300
O	-0.00769100	0.58711400	2.20991200
O	-0.00667200	-2.21204400	0.58431200
O	3.79700100	1.80767500	-0.46904000
O	1.89034800	1.33197500	-2.28351700
O	1.88695800	-1.32608300	2.29314400
O	2.12666900	-1.03675200	-3.91492100
H	6.33502900	-0.60011900	-0.67138000

Name	**D**		
charge	0	multiplicity	2
level of opt &freq	b3lyp/6-31G(d,p), PCM (Acetonitrile)		
electronic energy	-344.676922774 a.u.		
Temperature	298.15K		
Zero point energy (ZEP)	0.097717 a.u.		
Thermal correction to U	0.104001 a.u.		
Thermal correction to H	0.104945 a.u.		
Thermal correction to G	0.066509 a.u.		
level of sp	b3lyp/6-311g(d,p), SMD (Acetonitrile)		
Sum of electronic energy and thermal correction to G: -344.977032 a.u.			

Coordinate:

C	-0.56779900	-0.23421100	0.00020200
C	-2.01661900	-0.51122300	-0.00152800
O	-2.93296400	0.25058700	0.00015300
C	0.33891300	-1.30791100	0.00077000
H	-0.04600700	-2.33011500	0.00135400
C	1.71239900	-1.05802500	-0.00010400
H	2.42066600	-1.88935500	0.00146200
C	2.17948900	0.26128900	-0.00083600
H	3.25479600	0.45578000	-0.00150500
C	1.27880200	1.33624000	0.00012200

H	1.65291300	2.36266300	-0.00055200
C	-0.09318500	1.09369000	0.00062300
H	-0.81065900	1.91723600	0.00251700

Name	**S_4**		
charge	-2	multiplicity	1
level of opt &freq	b3lyp/6-31G(d,p), PCM (Acetonitrile)		
electronic energy	-1593.04046291 a.u.		
Temperature	298.15K		
Zero point energy (ZEP)	0.003840 a.u.		
Thermal correction to U	0.009606 a.u.		
Thermal correction to H	0.010550 a.u.		
Thermal correction to G	-0.027157 a.u.		
level of sp	b3lyp/6-311g(d,p), SMD (Acetonitrile)		
Sum of electronic energy and thermal correction to G: -1593.156529 a.u.			

Coordinate:

S	-1.07379300	2.37107900	0.11108600
S	-1.07379300	0.27444000	-0.11108600
S	1.07379300	-0.27444000	-0.11108600
S	1.07379300	-2.37107900	0.11108600

Name	**S_4^***		
charge	-2	multiplicity	3
level of opt &freq	b3lyp/6-31G(d,p), PCM (Acetonitrile)		
electronic energy	-1593.01337092 a.u.		
Temperature	298.15K		
Zero point energy (ZEP)	0.003211 a.u.		
Thermal correction to U	0.008662 a.u.		
Thermal correction to H	0.009606 a.u.		
Thermal correction to G	-0.029145 a.u.		
level of sp	b3lyp/6-311g(d,p), SMD (Acetonitrile)		
Sum of electronic energy and thermal correction to G: -1593.179313 a.u.			

Coordinate:

S	-1.15505800	2.88687600	0.03899100
S	-1.15505800	0.82402600	-0.03899100
S	1.15505800	-0.82402600	-0.03899100
S	1.15505800	-2.88687600	0.03899100

Name	**S_3**		
charge	-2	multiplicity	1

level of opt &freq	b3lyp/6-31G(d,p), PCM (Acetonitrile)
electronic energy	-1194.83201400 a.u.
Temperature	298.15K
Zero point energy (ZEP)	0.002343 a.u.
Thermal correction to U	0.006353 a.u.
Thermal correction to H	0.007297 a.u.
Thermal correction to G	-0.025717 a.u.
level of sp	b3lyp/6-311g(d,p), SMD (Acetonitrile)
Sum of electronic energy and thermal correction to G: -1194.945105 a.u.	

Coordinate:

S	1.77346700	-0.40103600	0.00000000
S	0.00000000	0.80326500	0.00000000
S	-1.77346700	-0.40223000	0.00000000

Name	**TS2**		
charge	-2	multiplicity	2
level of opt &freq	b3lyp/6-31G(d,p), PCM (Acetonitrile)		
electronic energy	-1937.953412 a.u.		
Temperature	298.15K		
Zero point energy (ZEP)	0.101391 a.u.		
Thermal correction to U	0.112326 a.u.		
Thermal correction to H	0.113271 a.u.		
Thermal correction to G	0.062478 a.u.		
level of sp	b3lyp/6-311g(d,p), SMD (Acetonitrile)		
Sum of electronic energy and thermal correction to G: -1937.924975 a.u.			

Coordinate:

S	-1.76495300	-2.18348100	-1.04331800
S	-2.99218300	-0.65343200	-0.30024400
S	-1.98859500	0.47162600	1.20307200
S	-0.88648100	2.07512500	0.40988100
C	1.73893000	0.52659000	-0.15500300
C	1.02163500	1.67948900	-0.61426800
O	1.02294000	2.24039900	-1.71029600
C	1.42973300	-0.21407900	1.01508700
H	0.52130800	0.00498900	1.55814900
C	2.26963800	-1.22928900	1.45409200
H	2.00034200	-1.78701200	2.34837600
C	3.44104700	-1.55761200	0.75624100
H	4.08885100	-2.35572900	1.10657700
C	3.75454200	-0.84202900	-0.40943700
H	4.65054600	-1.09224200	-0.97337700
C	2.92769400	0.17510000	-0.86308500
H	3.17150100	0.72037900	-1.76937200

Name	**H**		
charge	0	multiplicity	2
level of opt &freq	b3lyp/6-31G(d,p), PCM (Acetonitrile)		
electronic energy	-743.177094198 a.u.		
Temperature	298.15K		
Zero point energy (ZEP)	0.100379 a.u.		
Thermal correction to U	0.107900 a.u.		
Thermal correction to H	0.108844 a.u.		
Thermal correction to G	0.066366 a.u.		
level of sp	b3lyp/6-311g(d,p), SMD (Acetonitrile)		
Sum of electronic energy and thermal correction to G: -743.224303 a.u.			

Coordinate:

C	0.11922500	0.17906300	0.00005400
C	-1.31944600	0.48568600	0.00002800
O	-1.80094500	1.61662100	0.00077800
S	-2.57157400	-0.76521800	-0.00040100
C	0.58191200	-1.14291400	0.00038400
H	-0.12495900	-1.96770000	0.00077200
C	1.95015800	-1.39735200	0.00032200
H	2.31079200	-2.42019600	0.00059500
C	2.85605800	-0.33285600	-0.00007100
H	3.92266800	-0.53330100	-0.00013300
C	2.39652800	0.98784400	-0.00037700
H	3.10419500	1.81004200	-0.00067600
C	1.03020400	1.24693700	-0.00030800
H	0.65222400	2.26323300	-0.00055800

Name	**E**		
charge	0	multiplicity	2
level of opt &freq	b3lyp/6-31G(d,p), PCM (Acetonitrile)		
electronic energy	-192.516813849 a.u.		
Temperature	298.15K		
Zero point energy (ZEP)	0.070566 a.u.		
Thermal correction to U	0.075607 a.u.		
Thermal correction to H	0.076551 a.u.		
Thermal correction to G	0.042360 a.u.		
level of sp	b3lyp/6-311g(d,p), SMD (Acetonitrile)		
Sum of electronic energy and thermal correction to G: -192.526170 a.u.			

Coordinate:

C	1.12225600	-0.88100700	0.00045800

C	0.08468800	0.11910300	-0.00039300
C	-1.35758800	-0.35075100	-0.00023900
H	-2.02653700	0.51036300	-0.01327700
H	-1.56281500	-0.95786500	0.88797400
H	-1.55729400	-0.98181300	-0.87268200
O	0.37449500	1.32580000	-0.00015700
H	0.89705600	-1.94158600	-0.00120900
H	2.15750100	-0.55957100	0.00149500

Name	**TS3**		
charge	0	multiplicity	3
level of opt &freq	b3lyp/6-31G(d,p), PCM (Acetonitrile)		
electronic energy	-935.686043817 a.u.		
Temperature	298.15K		
Zero point energy (ZEP)	0.172981 a.u.		
Thermal correction to U	0.185962 a.u.		
Thermal correction to H	0.186906 a.u.		
Thermal correction to G	0.130534 a.u.		
level of sp	b3lyp/6-311g(d,p), SMD (Acetonitrile)		
Sum of electronic energy and thermal correction to G: -935.718848 a.u.			

Coordinate:

C	-1.20335500	0.09086000	0.41795600
C	0.11544100	0.24598400	0.99249600
O	0.71208300	-0.57756300	1.75677000
S	1.23417500	1.53665800	0.73818900
C	2.46232100	0.86227700	-0.96013800
C	2.38615300	-0.60262900	-0.90971000
C	3.51011600	-1.33808400	-0.21135700
H	3.28694800	-2.40493800	-0.17377500
H	3.63054900	-0.95365200	0.80716600
H	4.45992500	-1.17960500	-0.73311700
O	1.39429800	-1.18738400	-1.35406400
H	3.40991400	1.34102600	-0.72891700
H	1.88739900	1.32321700	-1.75840700
C	-1.70718200	1.01735100	-0.51230500
H	-1.09795100	1.86354100	-0.81454300
C	-2.97906100	0.84395300	-1.04451900
H	-3.36314100	1.55925300	-1.76425400
C	-3.76098300	-0.25005600	-0.65747500
H	-4.75350500	-0.38166800	-1.07583400
C	-3.26335300	-1.17413400	0.26716900
H	-3.86954300	-2.02278000	0.56701100
C	-1.99221200	-1.01049100	0.80466000

H -1.59575300 -1.72152700 1.52133000

Name	**Thioester**		
charge	0	multiplicity	1
level of opt &freq	b3lyp/6-31G(d,p), PCM (Acetonitrile)		
electronic energy	-935.792202992 a.u.		
Temperature	298.15K		
Zero point energy (ZEP)	0.177071 a.u.		
Thermal correction to U	0.189859 a.u.		
Thermal correction to H	0.190803 a.u.		
Thermal correction to G	0.135967 a.u.		
level of sp	b3lyp/6-311g(d,p), SMD (Acetonitrile)		
Sum of electronic energy and thermal correction to G: -935.819996 a.u.			

Coordinate:

C 0.70628700 0.84454600 0.02063000
C -0.58605300 1.56716800 -0.11697600
O -0.70767300 2.60688900 -0.73539900
S -2.08472600 0.87632600 0.63352900
C -1.72842100 -0.89753700 0.94896200
C -1.14961800 -1.70075300 -0.21617900
C -1.69615800 -1.45666800 -1.60172900
H -1.42791300 -2.29394100 -2.24633600
H -1.26222200 -0.54161400 -2.01732600
H -2.78010000 -1.31556500 -1.58177300
O -0.29876600 -2.54087600 0.01297900
H -2.72044100 -1.30902400 1.17044000
H -1.09785500 -1.03927200 1.82433900
C 1.15053500 0.34395800 1.25078600
H 0.54714200 0.48138600 2.14116000
C 2.38191600 -0.30088000 1.33159200
H 2.73121600 -0.67731500 2.28703800
C 3.16118400 -0.46637900 0.18433400
H 4.11226200 -0.98502400 0.24701500
C 2.72332500 0.03933700 -1.04215900
H 3.33228000 -0.08408700 -1.93143000
C 1.50596500 0.70991100 -1.12330500
H 1.15898600 1.11892200 -2.06596400

4. Characterization Data for the Products

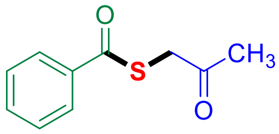

***S*-(2-Oxopropyl) benzothioate** (**4**); Colorless oil; 17.3 mg, 89% yield; ^{1}H NMR (600 MHz, $CDCl_3$) δ: 8.00–7.86 (m, 2H), 7.70–7.54 (m, 1H), 7.51–7.40 (m, 2H), 3.92 (s, 2H), 2.32 (s, 3H); ^{13}C NMR (151 MHz, $CDCl_3$) δ: 201.61, 190.10, 135.84, 133.60, 128.49, 127.14, 39.23, 28.64; HRMS (ESI) ($[M + H]^+$) Calcd For $C_{10}H_{11}O_2S^+$: 195.0474, Found: 195.0474. The NMR data are consistent with the reported values.[1]

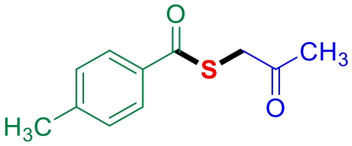

***S*-(2-Oxopropyl) 4-methylbenzothioate** (**5**); Colorless oil; 14.8 mg, 71% yield; ^{1}H NMR (600 MHz, $CDCl_3$) δ: 7.88–7.86 (m, 2H), 7.28–7.24 (m, 2H), 3.91 (s, 2H), 2.41 (s, 3H), 2.33 (s, 3H); ^{13}C NMR (151 MHz, $CDCl_3$) δ: 202.15, 189.85, 144.76, 133.49, 129.31, 127.42, 39.29, 28.77, 21.61; HRMS (ESI) ($[M + H]^+$) Calcd For $C_{11}H_{13}O_2S^+$: 209.0631, Found: 209.0631.

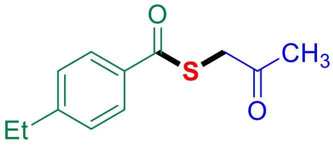

***S*-(2-Oxopropyl) 4-ethylbenzothioate** (**6**); Colorless oil; 15.1 mg, 68% yield; ^{1}H NMR (600 MHz, $CDCl_3$) δ: 7.90 (d, J = 8.3 Hz, 2H), 7.29 (d, J = 8.3 Hz, 2H), 3.92 (s, 2H), 2.71 (q, J = 7.6 Hz, 2H), 2.33 (s, 3H), 1.26 (t, J = 7.6 Hz, 3H); ^{13}C NMR (151 MHz, $CDCl_3$) δ: 202.23, 189.94, 150.99, 133.76, 128.20, 127.62, 39.35, 28.96, 28.82, 15.09; HRMS (ESI) ($[M + H]^+$) Calcd For $C_{12}H_{15}O_2S^+$: 223.0787, Found: 223.0785.

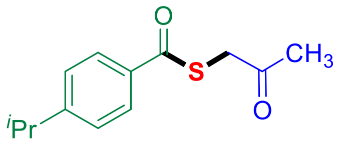

***S*-(2-Oxopropyl) 4-(*iso*-propyl)benzothioate** (**7**); Colorless oil; 17.0 mg, 72% yield; ^{1}H NMR (600 MHz, CDCl$_3$) δ: 7.92 (d, J = 8.4 Hz, 2H), 7.32 (d, J = 8.3 Hz, 2H), 3.92 (s, 2H), 2.97 (hept, J = 6.9 Hz, 1H), 2.33 (s, 3H), 1.27 (d, J = 6.9 Hz, 6H); ^{13}C NMR (151 MHz, CDCl$_3$) δ: 202.23, 189.92, 155.55, 133.89, 127.66, 126.81, 39.35, 34.29, 28.80, 23.59; HRMS (ESI) ([M + Na]$^+$) Calcd For $C_{13}H_{16}NaO_2S^+$: 259.0763, Found: 259.0766.

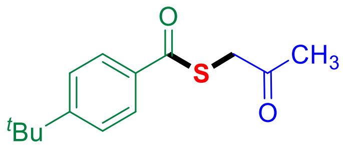

***S*-(2-Oxopropyl) 4-(*tert*-butyl)benzothioate** (**8**); Colorless oil; 18.3 mg, 73% yield; ^{1}H NMR (600 MHz, CDCl$_3$) δ: 7.93–7.91 (m, 2H), 7.49–7.47 (m, 2H), 3.92 (s, 2H), 2.32 (s, 3H), 1.33 (s, 9H); ^{13}C NMR (151 MHz, CDCl$_3$) δ: 202.04, 189.76, 157.63, 133.38, 127.26, 125.57, 39.23, 35.07, 30.90, 28.68; HRMS (ESI) ([M+H]$^+$) Calcd For $C_{14}H_{19}O_2S^+$: 251.1100, Found: 251.1096.

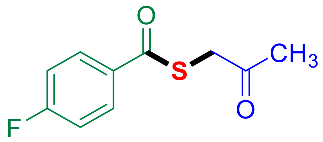

***S*-(2-Oxopropyl) 4-fluorobenzothioate** (**9**); Colorless oil; 13.8 mg, 65% yield; ^{1}H NMR (600 MHz, CDCl$_3$) δ: 8.02–8.00 (m, 2H), 7.16–7.13 (m, 2H), 3.94 (s, 2H), 2.35 (s, 3H); ^{13}C NMR (151 MHz, CDCl$_3$) δ: 201.67, 188.89, 166.14 (d, J = 255.7 Hz), 132.46 (d, J = 3.0 Hz), 130.02 (d, J = 9.4 Hz), 115.91 (d, J = 22.2 Hz), 39.56, 28.92; ^{19}F NMR (565 MHz, CDCl$_3$) δ: –103.73; HRMS (ESI) ([M + H]$^+$) Calcd For $C_{10}H_{10}FO_2S^+$: 213.0380, Found: 213.0378.

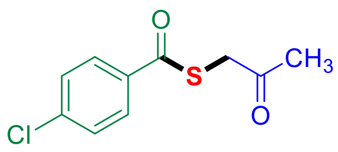

***S*-(2-Oxopropyl) 4-chlorobenzothioate** (**10**); Colorless oil; 16.4 mg, 72% yield; ^{1}H NMR (600 MHz, $CDCl_3$) *δ*: 7.92 (d, *J* = 8.6 Hz, 2H), 7.45 (d, *J* = 8.6 Hz, 2H), 3.94 (s, 2H), 2.35 (s, 3H); ^{13}C NMR (151 MHz, $CDCl_3$) *δ*: 201.54, 189.27, 140.29, 134.43, 129.05, 128.75, 39.59, 28.96; HRMS (ESI) ([M + H]$^+$) Calcd For $C_{10}H_{10}ClO_2S^+$: 229.0085, Found: 229.0085.

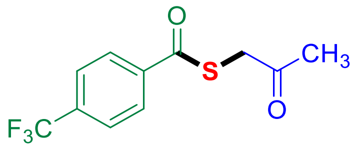

***S*-(2-Oxopropyl) 4-(trifluoromethyl)benzothioate** (**11**); Colorless oil; 19.7 mg, 75% yield; ^{1}H NMR (600 MHz, $CDCl_3$) *δ*: 8.08 (d, *J* = 8.1 Hz, 2H), 7.74 (d, *J* = 8.1 Hz, 2H), 3.99 (s, 2H), 2.36 (s, 3H); ^{13}C NMR (151 MHz, $CDCl_3$) *δ*: 201.10, 189.58, 138.81, 134.96 (q, *J* = 32.7 Hz), 127.71, 125.75 (q, *J* = 3.7 Hz), 123.35 (q, *J* = 272.9 Hz), 39.69, 28.93; ^{19}F NMR (565 MHz, $CDCl_3$) *δ*: –63.22; HRMS (ESI) ([M + H]$^+$) Calcd For $C_{11}H_{10}F_3O_2S^+$: 263.0348, Found: 263.0346.

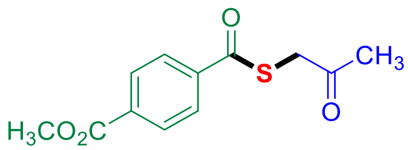

Methyl 4-(((2-oxopropyl)thio)carbonyl)benzoate (**12**); White solid; 18.1 mg, 72% yield; ^{1}H NMR (600 MHz, $CDCl_3$) *δ*: 8.13 (d, *J* = 8.5 Hz, 2H), 8.03 (d, *J* = 8.5 Hz, 2H), 3.97 (s, 2H), 3.95 (s, 3H), 2.36 (s, 3H); ^{13}C NMR (151 MHz, $CDCl_3$) *δ*: 201.36, 189.94, 165.91, 139.30, 134.52, 129.89, 127.32, 52.49, 39.68, 28.98; HRMS (ESI) ([M + H]$^+$) Calcd For $C_{12}H_{13}O_4S^+$: 253.0529, Found: 253.0526.

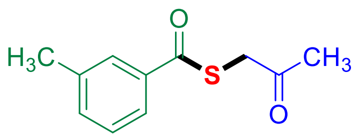

***S*-(2-Oxopropyl) 3-methylbenzothioate** (**13**); Colorless oil; 13.7 mg, 66% yield; ^{1}H NMR (600 MHz, $CDCl_3$) δ: 7.79–7.77 (m, 2H), 7.41–7.40 (m, 1H), 7.36–7.33 (m, 1H), 3.92 (s, 2H), 2.41 (s, 3H), 2.33 (s, 3H); ^{13}C NMR (151 MHz, $CDCl_3$) δ: 202.05, 190.48, 138.63, 136.09, 134.59, 128.58, 127.87, 124.63, 39.43, 28.84, 21.23; HRMS (ESI) ([M + H]$^+$) Calcd For $C_{11}H_{13}O_2S^+$: 209.0631, Found: 209.0630.

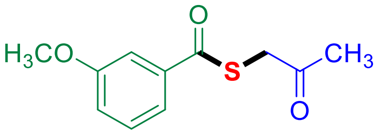

***S*-(2-Oxopropyl) 3-methoxybenzothioate** (**14**); Colorless oil; 14.3 mg, 64% yield; ^{1}H NMR (600 MHz, $CDCl_3$) δ: 7.60–7.58 (m, 1H), 7.46 (t, J = 2.1 Hz, 1H), 7.37 (t, J = 8.0 Hz, 1H), 7.14 (dd, J = 8.3, 2.6 Hz, 1H), 3.93 (s, 2H), 3.85 (s, 3H), 2.34 (s, 3H); ^{13}C NMR (151 MHz, $CDCl_3$) δ: 201.89, 190.28, 159.74, 137.34, 129.70, 120.27, 119.99, 111.56, 55.43, 39.51, 28.86; HRMS (ESI) ([M + Na]$^+$) Calcd For $C_{11}H_{12}NaO_3S^+$: 247.0399, Found: 247.0403.

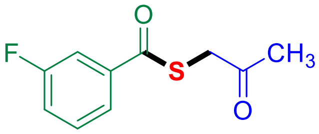

***S*-(2-Oxopropyl) 3-fluorobenzothioate** (**15**); Colorless oil; 16.1 mg, 76% yield; ^{1}H NMR (600 MHz, $CDCl_3$) δ: 7.78 (ddd, J = 7.8, 1.7, 1.0 Hz, 1H), 7.64 (ddd, J = 9.2, 2.6, 1.6 Hz, 1H), 7.45 (td, J = 8.0, 5.5 Hz, 1H), 7.31–7.28 (m, 1H), 3.96 (s, 2H), 2.35 (s, 3H); ^{13}C NMR (151 MHz, $CDCl_3$) δ: 201.28, 189.18 (d, J = 2.7 Hz), 162.50 (d, J = 248.6 Hz), 137.91 (d, J = 6.9 Hz), 130.34 (d, J = 7.7 Hz), 123.12 (d, J = 2.9 Hz), 120.65 (d, J = 21.3 Hz), 114.06 (d, J = 23.1 Hz), 39.52, 28.82; ^{19}F NMR (565 MHz, $CDCl_3$) δ: –111.28; HRMS (ESI) ([M + H]$^+$) Calcd For $C_{10}H_{10}FO_2S^+$: 213.0380, Found: 213.0378.

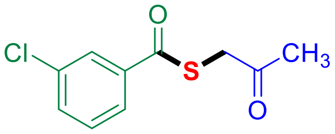

***S*-(2-Oxopropyl) 3-chlorobenzothioate** (**16**); Colorless oil; 16.0 mg, 70% yield; ^{1}H NMR (600 MHz, $CDCl_3$) δ: 7.94–7.93 (m, 1H), 7.86–7.85 (m, 1H), 7.57–7.56 (m, 1H), 7.41 (t, J = 7.9 Hz, 1H), 3.95 (s, 2H), 2.34 (s, 3H); ^{13}C NMR (151 MHz, $CDCl_3$) δ: 201.29, 189.21, 137.51, 134.94, 133.62, 129.97, 127.30, 125.47, 39.58, 28.89; HRMS (ESI) ($[M + Na]^+$) Calcd For $C_{10}H_9ClNaO_2S^+$: 250.9904, Found: 250.9909.

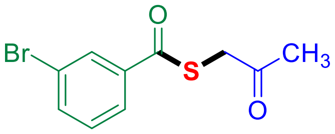

***S*-(2-Oxopropyl) 3-bromobenzothioate** (**17**); Colorless oil; 18.5 mg, 68% yield; ^{1}H NMR (600 MHz, $CDCl_3$) δ: 8.09–8.08 (m, 1H), 7.90–7.89 (m, 1H), 7.72–7.70 (m, 1H), 7.34 (t, J = 7.9 Hz, 1H), 3.95 (s, 2H), 2.34 (s, 3H); ^{13}C NMR (151 MHz, $CDCl_3$) δ: 201.23, 189.06, 137.65, 136.51, 130.19, 130.17, 125.89, 122.83, 39.57, 28.88; HRMS (ESI) ($[M + H]^+$) Calcd For $C_{10}H_{10}BrO_2S^+$: 272.9579, Found: 272.9578.

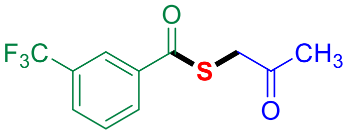

***S*-(2-Oxopropyl) 3-(trifluoromethyl)benzothioate** (**18**); Colorless oil; 18.1 mg, 69% yield; ^{1}H NMR (600 MHz, $CDCl_3$) δ: 8.23–8.22 (m, 1H), 8.17–8.16 (m, 1H), 7.87–7.85 (m, 1H), 7.63 (t, J = 7.8 Hz, 1H), 3.99 (s, 2H), 2.37 (s, 3H); ^{13}C NMR (151 MHz, $CDCl_3$) δ: 201.15, 189.35, 136.68, 131.40 (q, J = 33.2 Hz), 130.56, 130.16 (q, J = 3.4 Hz), 129.45, 124.24 (q, J = 3.8 Hz), 123.42 (q, J = 272.7 Hz), 39.70, 28.99; ^{19}F NMR (565 MHz, $CDCl_3$) δ: –62.89; HRMS (ESI) ($[M + H]^+$) Calcd For $C_{11}H_{10}F_3O_2S^+$: 263.0348, Found: 263.0347.

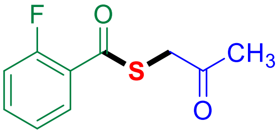

***S*-(2-Oxopropyl) 2-fluorobenzothioate** (**19**); Colorless oil; 15.3 mg, 72% yield; ^{1}H NMR (600 MHz, $CDCl_3$) δ: 7.90 (td, J = 7.5, 1.8 Hz, 1H), 7.57–7.53 (m, 1H), 7.26–

7.23 (m, 1H), 7.19–7.16 (m, 1H), 3.93 (s, 2H), 2.35 (s, 3H); ^{13}C NMR (151 MHz, $CDCl_3$) δ: 201.64, 187.21 (d, J = 5.3 Hz), 160.62 (d, J = 258.7 Hz), 134.91 (d, J = 8.8 Hz), 129.82, 124.40 (d, J = 11.0 Hz), 124.32 (d, J = 3.8 Hz), 116.92 (d, J = 22.3 Hz), 39.77 (d, J = 3.8 Hz), 28.94; ^{19}F NMR (565 MHz, $CDCl_3$) δ: –109.77; HRMS (ESI) ([M + H]$^+$) Calcd For $C_{10}H_{10}FO_2S^+$: 213.0380, Found: 213.0374.

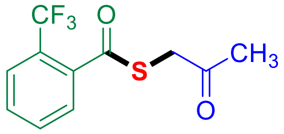

***S*-(2-Oxopropyl) 2-(trifluoromethyl)benzothioate** (**20**); Colorless oil; 18.6 mg, 71% yield; ^{1}H NMR (600 MHz, $CDCl_3$) δ: 7.80–7.76 (m, 2H), 7.67–7.63 (m, 2H), 3.96 (s, 2H), 2.35 (s, 3H); ^{13}C NMR (151 MHz, $CDCl_3$) δ: 201.22, 191.44, 136.78, 131.87, 131.56, 128.80, 127.32 (q, J = 32.9 Hz), 127.07 (q, J = 5.2 Hz), 123.09 (q, J = 273.9 Hz), 40.40, 28.79; ^{19}F NMR (565 MHz, $CDCl_3$) δ: –58.72; HRMS (ESI) ([M + H]$^+$) Calcd For $C_{11}H_{10}F_3O_2S^+$: 263.0348, Found: 263.0349.

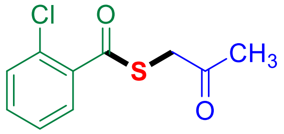

***S*-(2-Oxopropyl) 2-chlorobenzothioate** (**21**); Colorless oil; 18.5 mg, 81% yield; ^{1}H NMR (600 MHz, $CDCl_3$) δ: 7.74–7.73 (m, 1H), 7.47–7.42 (m, 2H), 7.36–7.33 (m, 1H), 3.95 (s, 2H), 2.35 (s, 3H); ^{13}C NMR (151 MHz, $CDCl_3$) δ: 201.32, 190.25, 136.23, 132.67, 130.95, 130.91, 129.39, 126.75, 40.24, 28.85; HRMS (ESI) ([M + H]$^+$) Calcd For $C_{10}H_{10}ClO_2S^+$: 229.0085, Found: 229.0081.

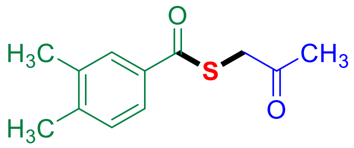

***S*-(2-Oxopropyl) 3,4-dimethylbenzothioate** (**22**); White solid; 15.8 mg, 71% yield; ^{1}H NMR (600 MHz, $CDCl_3$) δ: 7.74 (s, 1H), 7.73–7.71 (m, 1H), 7.21 (d, J = 7.9 Hz, 1H), 3.91 (s, 2H), 2.33 (s, 3H), 2.31 (s, 6H); ^{13}C NMR (151 MHz, $CDCl_3$) δ: 202.26,

190.01, 143.51, 137.13, 133.88, 129.87, 128.40, 125.06, 39.31, 28.77, 20.02, 19.64; HRMS (ESI) ([M + Na]$^+$) Calcd For $C_{12}H_{14}NaO_2S^+$: 245.0607, Found: 245.0611.

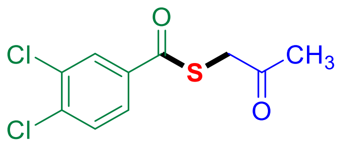

***S*-(2-Oxopropyl) 3,4-dichlorobenzothioate** (**23**); White solid; 14.4 mg, 55% yield; ^{1}H NMR (600 MHz, $CDCl_3$) δ: 8.05 (d, J = 2.1 Hz, 1H), 7.81 (dd, J = 8.4, 2.1 Hz, 1H), 7.56–7.50 (m, 1H), 3.96 (s, 2H), 2.35 (s, 3H); ^{13}C NMR (151 MHz, $CDCl_3$) δ: 201.07, 188.40, 138.39, 135.60, 133.48, 130.81, 129.22, 126.38, 39.75, 29.01; HRMS (ESI) ([M + Na]$^+$) Calcd For $C_{10}H_8Cl_2NaO_2S^+$: 284.9514, Found: 284.9513.

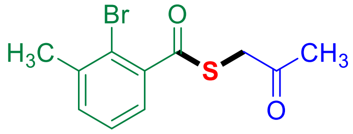

***S*-(2-Oxopropyl) 2-bromo-3-methylbenzothioate** (**24**); White solid; 17.7 mg, 62% yield; ^{1}H NMR (600 MHz, $CDCl_3$) δ: 7.79 (s, 1H), 7.41 (d, J = 7.6 Hz, 1H), 7.35 (t, J = 7.9 Hz, 1H), 3.93 (s, 2H), 2.42 (s, 3H), 2.35 (s, 3H); ^{13}C NMR (151 MHz, $CDCl_3$) δ: 202.16, 190.52, 138.63, 136.01, 134.62, 128.58, 127.87, 124.62, 39.45, 28.92, 21.26; HRMS (ESI) ([M + Na]$^+$) Calcd For $C_{11}H_{11}BrNaO_2S^+$: 305.9555, Found: 308.9559.

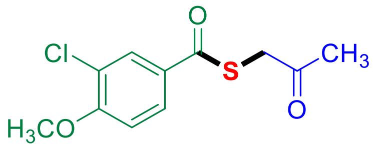

***S*-(2-Oxopropyl) 3-chloro-4-methoxybenzothioate** (**25**); White solid; 17.8 mg, 69% yield; ^{1}H NMR (600 MHz, $CDCl_3$) δ: 8.00 (s, 1H), 7.90 (d, J = 8.7 Hz, 1H), 6.97 (d, J = 8.7 Hz, 1H), 3.97 (d, J = 1.4 Hz, 3H), 3.93 (s, 2H), 2.34 (d, J = 1.5 Hz, 3H); ^{13}C NMR (151 MHz, $CDCl_3$) δ: 201.79, 187.96, 159.28, 129.49, 129.38, 127.84, 123.01, 111.30, 56.40, 39.45, 28.87; HRMS (ESI) ([M + H]$^+$) Calcd For $C_{11}H_{12}ClO_2S^+$: 259.0190, Found: 259.0195.

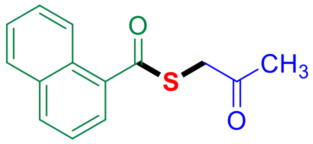

***S*-(2-Oxopropyl) naphthalene-1-carbothioate** (**26**); Colorless oil; 17.1 mg, 70% yield; ^{1}H NMR (600 MHz, $CDCl_3$) δ: 8.54–8.45 (m, 1H), 8.17–8.10 (m, 1H), 8.05–7.99 (m, 1H), 7.91–7.84 (m, 1H), 7.69–7.43 (m, 3H), 3.99 (s, 2H), 2.39 (s, 3H); ^{13}C NMR (151 MHz, $CDCl_3$) δ: 201.99, 192.50, 134.21, 133.74, 133.49, 129.19, 128.37, 128.26, 128.22, 126.75, 125.10, 124.43, 40.34, 28.95; HRMS (ESI) ([M + Na]$^+$) Calcd For $C_{14}H_{12}NaO_2S^+$: 267.0450, Found: 267.0452.

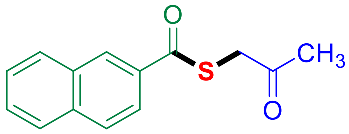

***S*-(2-Oxopropyl) naphthalene-2-carbothioate** (**27**); Colorless oil; 15.0 mg, 61% yield; ^{1}H NMR (600 MHz, $CDCl_3$) δ: 8.56 (s, 1H), 7.99–7.96 (m, 2H), 7.90–7.87 (m, 2H), 7.62–7.55 (m, 2H), 3.99 (s, 2H), 2.37 (s, 3H); ^{13}C NMR (151 MHz, $CDCl_3$) δ: 202.01, 190.28, 135.90, 133.37, 132.34, 129.57, 129.09, 128.73, 128.62, 127.80, 127.04, 123.03, 39.59, 28.92; HRMS (ESI) ([M + H]$^+$) Calcd For $C_{14}H_{13}O_2S^+$: 245.0631, Found: 245.0635.

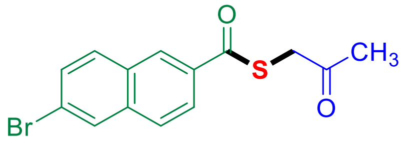

***S*-(2-Oxopropyl) 6-bromonaphthalene-2-carbothioate** (**28**); Colorless oil; 21.3 mg, 66% yield; ^{1}H NMR (600 MHz, $CDCl_3$) δ: 8.51 (d, J = 1.8 Hz, 1H), 8.05 (s, 1H), 8.01–7.99 (m, 1H), 7.82 (dd, J = 15.8, 8.7 Hz, 2H), 7.65–7.63 (m, 1H), 4.00 (s, 2H), 2.37 (s, 3H); ^{13}C NMR (151 MHz, $CDCl_3$) δ: 201.75, 190.02, 136.75, 133.73, 131.06, 130.79, 130.62, 130.00, 128.89, 127.71, 124.21, 123.21, 39.67, 28.99; HRMS (ESI) ([M + H]$^+$) Calcd For $C_{14}H_{12}BrO_2S^+$: 322.9736, Found: 322.9738.

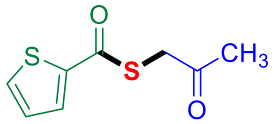

***S*-(2-Oxopropyl) thiophene-2-carbothioate** (**29**); Colorless oil; 14.2 mg, 71% yield; ^{1}H NMR (600 MHz, $CDCl_3$) δ: 7.86–7.85 (m, 1H), 7.67–7.66 (m, 1H), 7.14 (dd, J = 4.9, 3.9 Hz, 1H), 3.93 (s, 2H), 2.34 (s, 3H); ^{13}C NMR (151 MHz, $CDCl_3$) δ: 201.73, 182.30, 140.92, 133.43, 131.84, 128.06, 39.45, 28.86; HRMS (ESI) ([M + H]$^+$) Calcd For $C_8H_9O_2S_2^+$: 201.0038, Found: 201.0042.

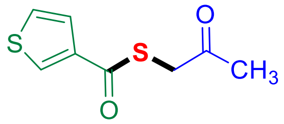

***S*-(2-Oxopropyl) thiophene-3-carbothioate** (**30**); Colorless oil; 10.6 mg, 53% yield; ^{1}H NMR (600 MHz, $CDCl_3$) δ: 8.17–8.16 (m, 1H), 7.55–7.54 (m, 1H), 7.36 (dd, J = 5.1, 2.9 Hz, 1H), 3.92 (s, 2H), 2.33 (s, 3H); ^{13}C NMR (151 MHz, $CDCl_3$) δ: 201.88, 183.76, 139.87, 131.27, 126.71, 125.96, 39.28, 28.84; HRMS (ESI) ([M + H]$^+$) Calcd For $C_8H_9O_2S_2^+$: 201.0038, Found: 201.0039.

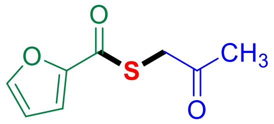

***S*-(2-Oxopropyl) furan-2-carbothioate** (**31**); Colorless oil; 13.6 mg, 74% yield; ^{1}H NMR (600 MHz, $CDCl_3$) δ: 7.61–7.61 (m, 1H), 7.24 (d, J = 4.3 Hz, 1H), 6.57 (dd, J = 3.6, 1.7 Hz, 1H), 3.91 (s, 2H), 2.33 (s, 3H); ^{13}C NMR (151 MHz, $CDCl_3$) δ: 201.65, 178.81, 150.13, 146.65, 116.33, 112.44, 38.52, 28.81; HRMS (ESI) ([M + Na]$^+$) Calcd For $C_8H_8NaO_3S^+$: 207.0086, Found: 207.0087.

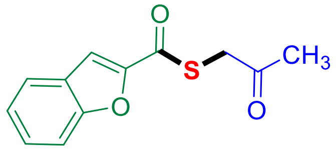

***S*-(2-Oxopropyl) benzofuran-2-carbothioate** (**32**); White solid; 17.8 mg, 76% yield; ^{1}H NMR (600 MHz, $CDCl_3$) δ: 7.66 (d, J = 8.0 Hz, 1H), 7.55–7.53 (m, 2H), 7.45 (t, J = 8.4 Hz, 1H), 7.29 (t, J = 7.6 Hz, 1H), 3.97 (s, 2H), 2.34 (s, 3H); ^{13}C NMR (151 MHz, $CDCl_3$) δ: 201.11, 180.47, 155.29, 150.10, 128.20, 126.52, 123.91, 123.06, 112.13, 111.73, 38.67, 28.70; HRMS (ESI) ([M + H]$^+$) Calcd For $C_{12}H_{11}O_3S^+$: 235.0423, Found: 235.0427.

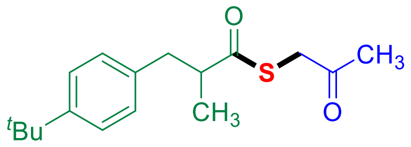

***S*-(2-Oxopropyl) 3-(4-(*tert*-butyl)phenyl)-2-methylpropanethioate** (**33**); White solid; 21.3 mg, 73% yield; ^{1}H NMR (600 MHz, $CDCl_3$) δ: 7.29 (d, J = 6.2 Hz, 2H), 7.08 (d, J = 10.4 Hz, 2H), 3.70 (qd, J = 16.0, 2.1 Hz, 2H), 3.09–3.02 (m, 1H), 3.01–2.95 (m, 1H), 2.68–2.62 (m, 1H), 2.17 (s, 3H), 1.30 (s, 9H), 1.20 (dd, J = 6.9, 2.1 Hz, 3H); ^{13}C NMR (151 MHz, $CDCl_3$) δ: 202.12, 201.60, 149.26, 135.43, 128.63, 125.26, 49.96, 39.16, 39.13, 34.32, 31.30, 28.45, 17.16; HRMS (ESI) ([M + Na]$^+$) Calcd For $C_{17}H_{24}NaO_2S^+$: 315.1389, Found: 315.1390.

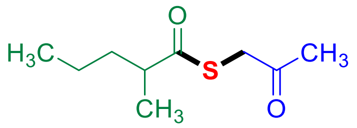

***S*-(2-Oxopropyl) 2-methylpentanethioate** (**34**); Colorless oil; 12.4 mg, 66% yield; ^{1}H NMR (600 MHz, $CDCl_3$) δ: 3.73 (s, 2H), 2.72 (h, J = 6.9 Hz, 1H), 2.27 (s, 3H), 1.74–1.70 (m, 1H), 1.45–1.41 (m, 1H), 1.40–1.33 (m, 2H), 1.19 (d, J = 6.9 Hz, 3H), 0.91 (t, J = 7.3 Hz, 3H); ^{13}C NMR (151 MHz, $CDCl_3$) δ: 202.35, 202.07, 48.09, 39.14, 36.15, 28.68, 20.22, 17.41, 13.89; HRMS (ESI) ([M + Na]$^+$) Calcd For $C_9H_{16}NaO_2S^+$: 211.0763, Found: 211.0768.

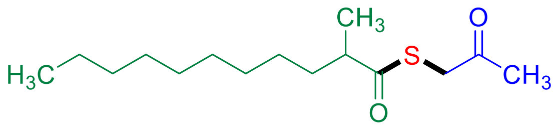

***S*-(2-Oxopropyl) 2-methylundecanethioate** (**35**); Colorless oil; 21.5 mg, 79% yield; ^{1}H NMR (600 MHz, $CDCl_3$) δ: 3.73 (s, 2H), 2.69 (h, J = 6.9 Hz, 1H), 2.26 (s, 3H), 1.74–1.70 (m, 1H), 1.46–1.42 (m, 1H), 1.31–1.26 (m, 14H), 1.19 (d, J = 6.9 Hz, 3H), 0.88 (t, J = 7.0 Hz, 3H); ^{13}C NMR (151 MHz, $CDCl_3$) δ: 202.38, 202.09, 48.38, 39.17, 34.08, 31.86, 29.52, 29.49, 29.41, 29.27, 28.68, 27.06, 22.66, 17.47, 14.09; HRMS (ESI) ($[M + H]^+$) Calcd For $C_{15}H_{29}O_2S^+$: 273.1883, Found: 273.1884.

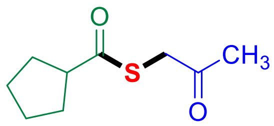

***S*-(2-Oxopropyl) cyclopentanecarbothioate** (**36**); Colorless oil; 15.3 mg, 82% yield; ^{1}H NMR (600 MHz, $CDCl_3$) δ: 3.73 (s, 2H), 3.09–2.99 (m, 1H), 2.27 (s, 3H), 1.96–1.90 (m, 2H), 1.87–1.81 (m, 2H), 1.75–1.70 (m, 2H), 1.64–1.59 (m, 2H); ^{13}C NMR (151 MHz, $CDCl_3$) δ: 202.17, 201.45, 52.81, 39.32, 30.47, 28.72, 25.82; HRMS (ESI) ($[M + Na]^+$) Calcd For $C_9H_{14}NaO_2S^+$: 209.0607, Found: 209.0614.

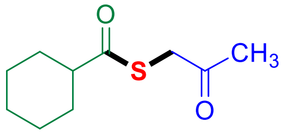

***S*-(2-Oxopropyl) cyclohexanecarbothioate** (**37**); Colorless oil; 14.2 mg, 71% yield; ^{1}H NMR (600 MHz, $CDCl_3$) δ: 3.72 (s, 2H), 2.55 (tt, J = 11.5, 3.6 Hz, 1H), 2.26 (s, 3H), 1.97–1.94 (m, 2H), 1.82–1.77 (m, 2H), 1.68–1.65 (m, 1H), 1.48 (qd, J = 12.1, 3.5 Hz, 2H), 1.31–1.21 (m, 3H); ^{13}C NMR (151 MHz, $CDCl_3$) δ: 202.23, 201.49, 52.26, 39.04, 29.40, 28.70, 25.52, 25.37; HRMS (ESI) ($[M + Na]^+$) Calcd For $C_{10}H_{16}NaO_2S^+$: 223.0763, Found: 223.0766.

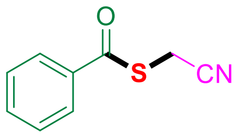

***S*-(Cyanomethyl) benzothioate** (**38**); Colorless oil; 12.9 mg, 73% yield; ^{1}H NMR (600 MHz, $CDCl_3$) δ: 7.95–7.93 (m, 2H), 7.66–7.63 (m, 1H), 7.52–7.48 (m, 2H), 3.87

(s, 2H); ^{13}C NMR (151 MHz, $CDCl_3$) δ: 187.89, 135.18, 134.52, 128.99, 127.51, 115.83, 14.33; HRMS (ESI) ([M + Na]$^+$) Calcd For $C_9H_7NNaOS^+$: 200.0141, Found: 200.0145; The NMR data are consistent with the reported values.[2]

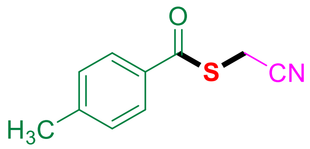

***S*-(Cyanomethyl) 4-methylbenzothioate** (**39**); Colorless oil; 12.0 mg, 63% yield; ^{1}H NMR (600 MHz, $CDCl_3$) δ: 7.84 (d, J = 8.2 Hz, 2H), 7.29 (d, J = 8.0 Hz, 2H), 3.85 (s, 2H), 2.44 (s, 3H); ^{13}C NMR (151 MHz, $CDCl_3$) δ: 187.39, 145.72, 132.68, 129.66, 127.61, 115.96, 21.78, 14.24; HRMS (ESI) ([M + Na]$^+$) Calcd For $C_{10}H_9NNaOS^+$: 214.0297, Found: 214.0298; The NMR data are consistent with the reported values.[3]

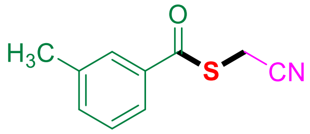

***S*-(Cyanomethyl) 3-methylbenzothioate** (**40**); Colorless oil; 13.2 mg, 69% yield; ^{1}H NMR (600 MHz, $CDCl_3$) δ: 7.74–7.73 (m, 2H), 7.46–7.44 (m, 1H), 7.39–7.36 (m, 1H), 3.85 (s, 2H), 2.42 (s, 3H); ^{13}C NMR (151 MHz, $CDCl_3$) δ: 187.98, 138.99, 135.28, 135.17, 128.82, 127.88, 124.70, 115.88, 21.21, 14.28; HRMS (ESI) ([M + Na]$^+$) Calcd For $C_{10}H_9NNaOS^+$: 214.0297, Found: 214.0300; The NMR data are consistent with the reported values.[3]

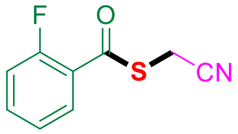

***S*-(Cyanomethyl) 2-fluorobenzothioate** (**41**); Colorless oil; 14.0 mg, 72% yield; ^{1}H NMR (600 MHz, $CDCl_3$) δ: 7.93–7.91 (m, 1H), 7.63–7.59 (m, 1H), 7.29 (t, J = 7.6 Hz, 1H), 7.24–7.18 (m, 1H), 3.86 (s, 2H); ^{13}C NMR (151 MHz, $CDCl_3$) δ: 184.62 (d, J = 5.5 Hz), 161.04 (d, J = 258.8 Hz), 135.78 (d, J = 9.2 Hz), 129.90, 124.66 (d, J = 3.6 Hz), 123.25 (d, J = 11.0 Hz), 117.03 (d, J = 22.2 Hz), 115.67, 14.62 (d, J = 6.6 Hz);

^{19}F NMR (565 MHz, $CDCl_3$) δ: –108.71; HRMS (ESI) ([M + Na]$^+$) Calcd For $C_9H_6FNNaOS^+$: 218.0046, Found: 218.0048.

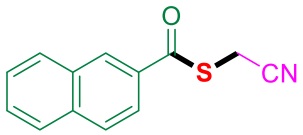

***S*-(Cyanomethyl) naphthalene-2-carbothioate** (**42**); Colorless oil; 17.3 mg, 76% yield; ^1H NMR (600 MHz, $CDCl_3$) δ: 8.48 (s, 1H), 7.96 (d, J = 8.2 Hz, 1H), 7.93–7.87 (m, 3H), 7.65–7.62 (m, 1H), 7.60–7.57 (m, 1H), 3.91 (s, 2H); ^{13}C NMR (151 MHz, $CDCl_3$) δ: 187.77, 136.13, 132.42, 132.25, 129.62, 129.36, 129.16, 128.96, 127.87, 127.30, 122.75, 115.90, 14.43; HRMS (ESI) ([M + Na]$^+$) Calcd For $C_{13}H_9NNaOS^+$: 250.0297, Found: 250.0301.

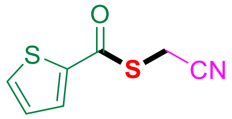

***S*-(Cyanomethyl) thiophene-2-carbothioate** (**43**); Colorless oil; 11.1 mg, 61% yield; ^1H NMR (600 MHz, $CDCl_3$) δ: 7.84–7.83 (m, 1H), 7.75–7.74 (m, 1H), 7.18–7.17 (m, 1H), 3.87 (s, 2H); ^{13}C NMR (151 MHz, $CDCl_3$) δ: 179.58, 139.64, 134.56, 132.44, 128.31, 115.68, 14.36; HRMS (ESI) ([M + Na]$^+$) Calcd For $C_7H_5NNaOS_2^+$: 205.9705, Found: 205.9709. The NMR data are consistent with the reported values.[2]

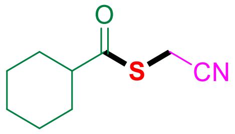

***S*-(Cyanomethyl) cyclohexanecarbothioate** (**44**); Colorless oil; 11.7 mg, 64% yield; ^1H NMR (600 MHz, $CDCl_3$) δ: 3.66 (s, 2H), 2.55 (tt, J = 11.4, 3.6 Hz, 1H), 1.96 (dd, J = 13.1, 3.7 Hz, 2H), 1.82–1.79 (m, 2H), 1.70–1.66 (m, 1H), 1.50 (qd, J = 12.0, 3.5 Hz, 2H), 1.32–1.24 (m, 3H); ^{13}C NMR (151 MHz, $CDCl_3$) δ: 198.80, 115.98, 52.20, 29.18, 25.39, 25.24, 13.77; HRMS (ESI) ([M + H]$^+$) Calcd For $C_9H_{14}NOS^+$: 184.0791, Found: 184.0791.

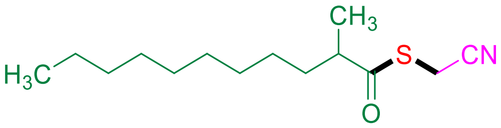

***S*-(Cyanomethyl) 2-methylundecanethioate** (**45**); Colorless oil; 17.1 mg, 67% yield; ^{1}H NMR (600 MHz, $CDCl_3$) δ: 3.67 (s, 2H), 2.69 (h, J = 6.9 Hz, 1H), 1.76–1.71 (m, 1H), 1.49–1.44 (m, 1H), 1.31–1.21 (m, 17H), 0.88 (t, J = 7.0 Hz, 3H); ^{13}C NMR (151 MHz, $CDCl_3$) δ: 199.68, 115.89, 48.44, 33.84, 31.80, 29.44, 29.37, 29.32, 29.21, 26.92, 22.61, 17.16, 14.04, 13.87; HRMS (ESI) ([M + Na]$^+$) Calcd For $C_{14}H_{25}NNaOS^+$: 278.1549, Found: 278.1547.

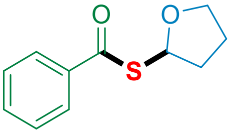

***S*-(Tetrahydrofuran-2-yl) benzothioate** (**46**); Colorless oil; 15.4 mg, 74% yield; ^{1}H NMR (600 MHz, $CDCl_3$) δ: 7.93 (d, J = 7.8 Hz, 2H), 7.56–7.54 (m, 1H), 7.43 (t, J = 7.6 Hz, 2H), 6.20 (dd, J = 7.2, 3.3 Hz, 1H), 3.97 (h, J = 8.2 Hz, 2H), 2.49–2.43 (m, 1H), 2.18–2.13 (m, 1H), 2.07–1.96 (m, 2H); ^{13}C NMR (151 MHz, $CDCl_3$) δ: 191.37, 136.98, 133.30, 128.43, 127.21, 83.53, 68.28, 32.65, 24.52; The NMR data are consistent with the reported values.[4]

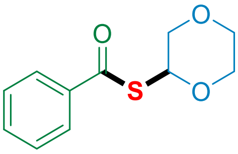

***S*-(1,4-Dioxan-2-yl) benzothioate** (**47**); Colorless oil; 18.2 mg, 81% yield; ^{1}H NMR (600 MHz, $CDCl_3$) δ: 7.98 (dd, J = 8.2, 1.3 Hz, 2H), 7.59 (t, J = 7.4 Hz, 1H), 7.46 (t, J = 7.8 Hz, 2H), 5.92 (t, J = 3.3 Hz, 1H), 4.10–4.06 (m, 2H), 3.87 (dd, J = 12.0, 3.7 Hz, 1H), 3.80–3.78 (m, 2H), 3.77–3.74 (m, 1H); ^{13}C NMR (151 MHz, $CDCl_3$) δ: 189.52, 136.62, 133.68, 128.60, 127.43, 78.79, 70.28, 66.58, 63.77; The NMR data are consistent with the reported values.[4]

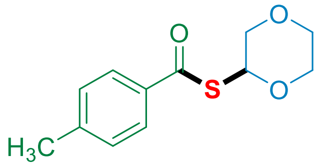

***S*-(1,4-Dioxan-2-yl) 4-methylbenzothioate** (**48**); Colorless oil; 18.1 mg, 76% yield; ^{1}H NMR (600 MHz, $CDCl_3$) δ: 7.88 (d, J = 7.9 Hz, 2H), 7.25 (d, J = 8.0 Hz, 2H), 5.91–5.90 (m, 1H), 4.10–4.05 (m, 2H), 3.86 (dd, J = 11.9, 3.8 Hz, 1H), 3.79–3.78 (m, 2H), 3.77–3.73 (m, 1H), 2.41 (s, 3H); ^{13}C NMR (151 MHz, $CDCl_3$) δ: 189.11, 144.72, 134.14, 129.31, 127.57, 78.69, 70.35, 66.63, 63.88, 21.64; HRMS (ESI) ([M + H]$^+$) Calcd For $C_{12}H_{15}O_3S^+$: 239.0736, Found: 239.0739.

5. References

[1] Ramazani, A.; Nasrabadi, F. Z. *Phosphorus, Sulfur Silicon Relat. Elem.* **2013**, *188*, 1214–1219.

[2] Tiefenbrunner, I.; Brutiu, B. R.; Stopka, T.; Maulide, N. *J. Org. Chem.* **2023**, *88*, 3941–3944.

[3] Wepplo, P. *Synth. Commun.* **1989**, *19*, 1533–1538.

[4] Liu, G.; Zheng, N.; Duan, X.; Sun, X.; Song, W. *Green Chem.* **2023**, *25*, 5035–5040.

6. Copies of ^{1}H, ^{13}C NMR and ^{19}F NMR Spectra

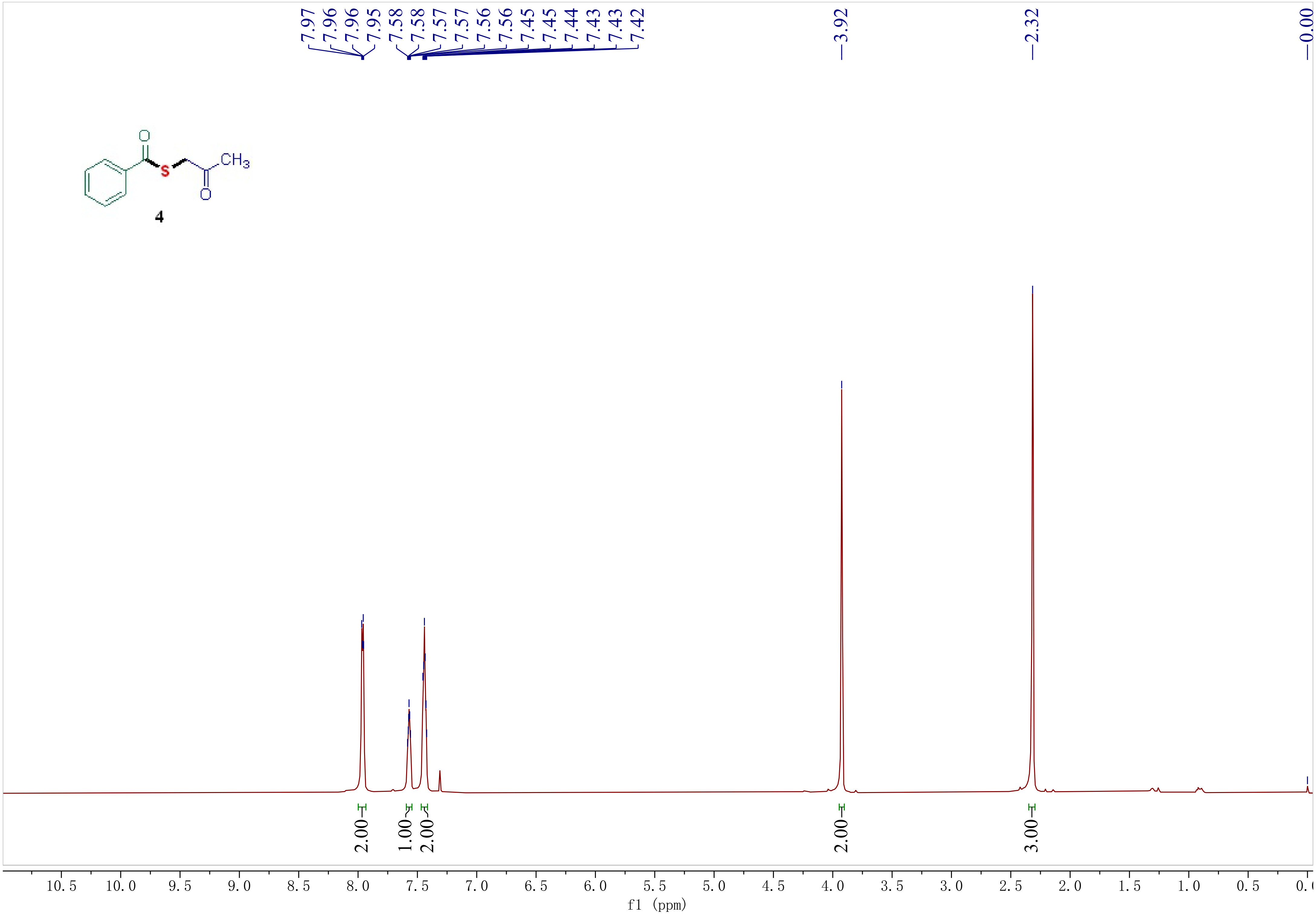

7.97
7.96
7.96
7.95
7.58
7.58
7.57
7.57
7.56
7.56
7.45
7.45
7.44
7.43
7.43
7.42
3.92
2.32
0.00
4
2.00
1.00
2.00
2.00
3.00
10.5
10.0
9.5
9.0
8.5
8.0
7.5
7.0
6.5
6.0
5.5
5.0
4.5
4.0
3.5
3.0
2.5
2.0
1.5
1.0
0.5
f1 (ppm)

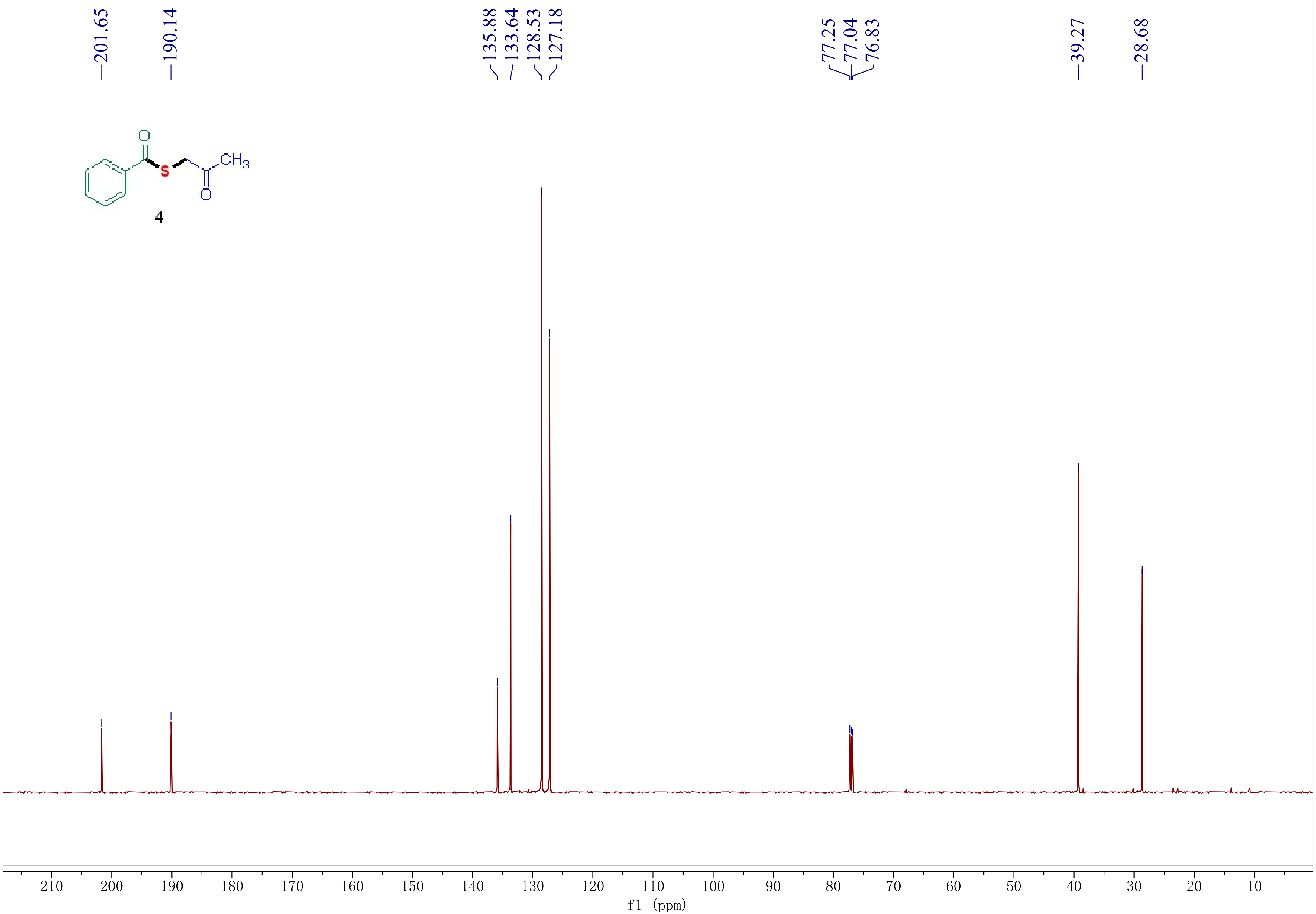

201.65
190.14
135.88
133.64
128.53
127.18
77.25
77.04
76.83
39.27
28.68
O
S
CH3
O
4
210 200 190 180 170 160 150 140 130 120 110 100 90 80 70 60 50 40 30 20 10
f1 (ppm)

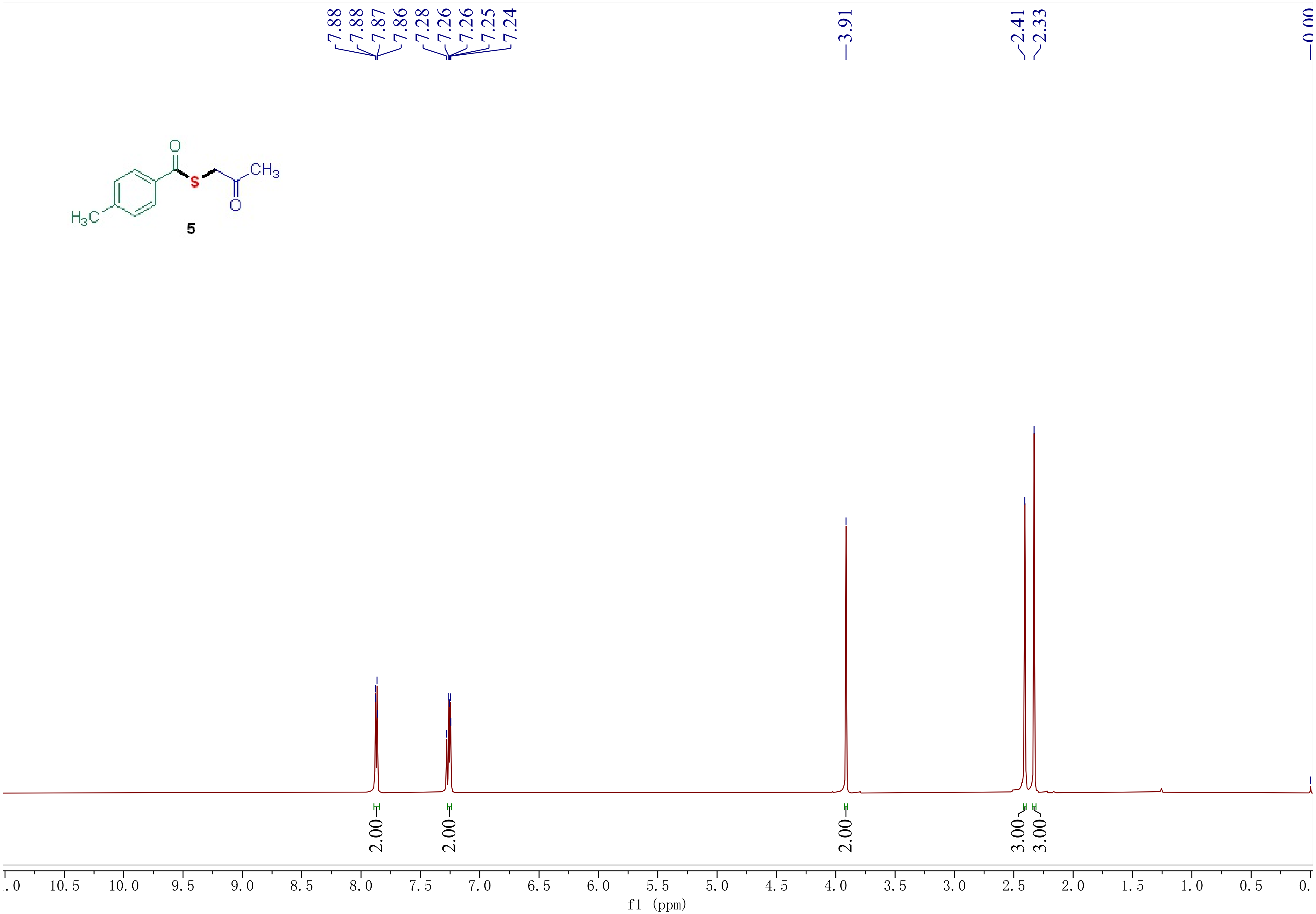
7.88
7.88
7.87
7.86
7.28
7.26
7.26
7.25
7.24
3.91
2.41
2.33
0.00
O
S
CH3
O
H3C
5
2.00
2.00
2.00
3.00
3.00
10.5
10.0
9.5
9.0
8.5
8.0
7.5
7.0
6.5
6.0
5.5
5.0
4.5
4.0
3.5
3.0
2.5
2.0
1.5
1.0
0.5
f1 (ppm)

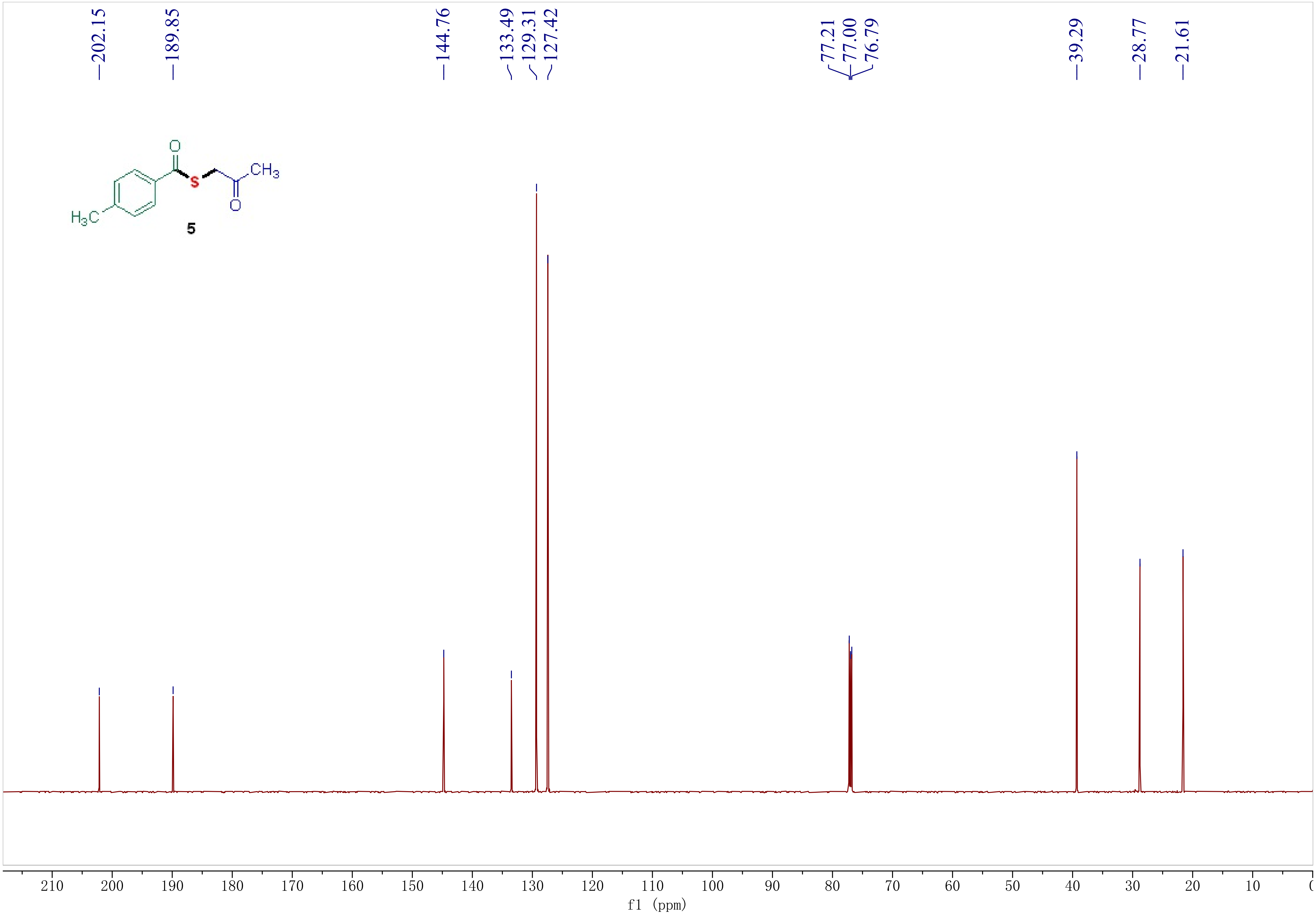

202.15
189.85
144.76
133.49
129.31
127.42
77.21
77.00
76.79
39.29
28.77
21.61
O
S
CH3
O
H3C
5
210
200
190
180
170
160
150
140
130
120
110
100
90
80
70
60
50
40
30
20
10
f1 (ppm)

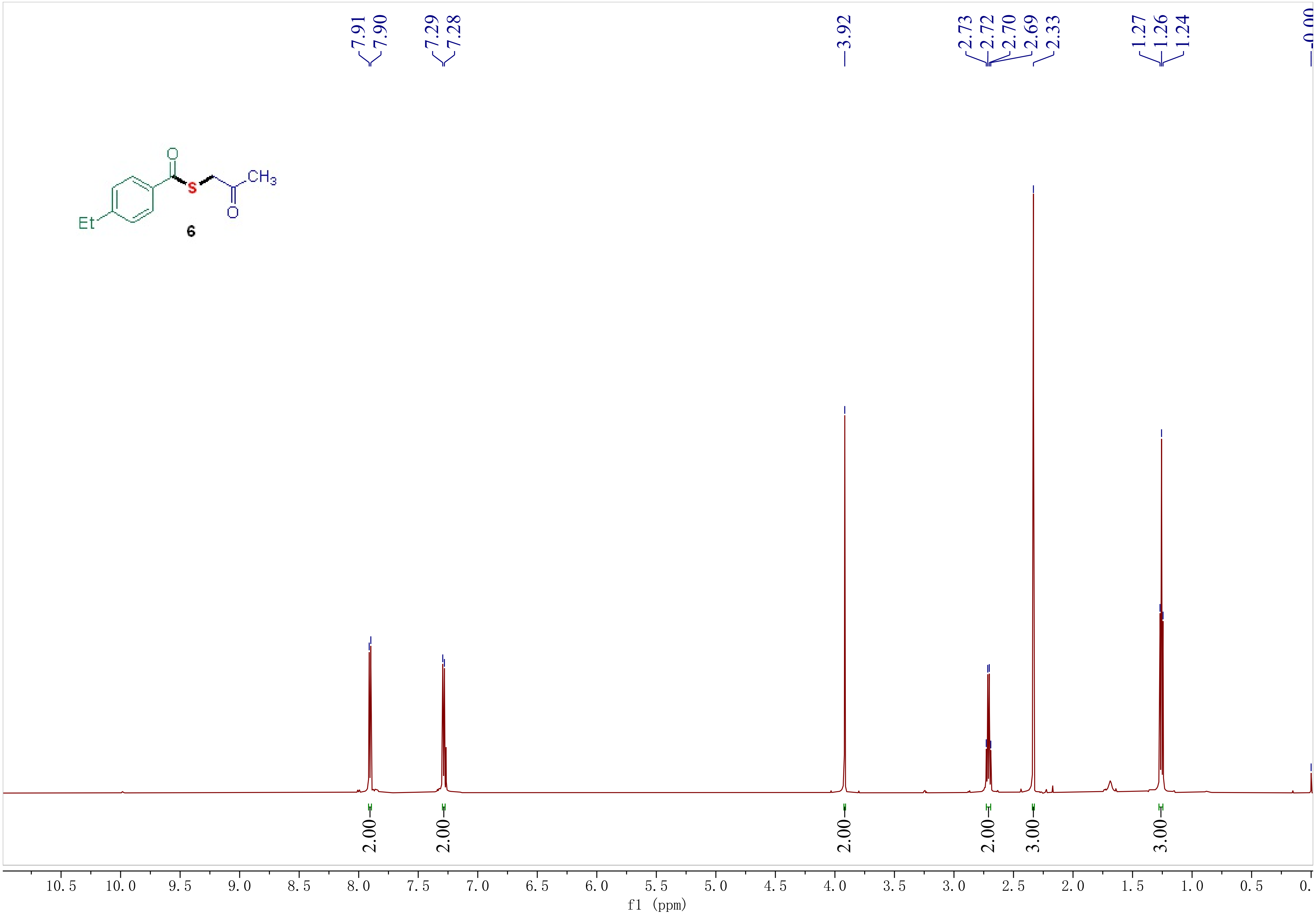

7.91
7.90
7.29
7.28
3.92
2.73
2.72
2.70
2.69
2.33
1.27
1.26
1.24
0.00
Et
S
O
CH3
6
2.00
2.00
2.00
2.00
3.00
3.00
10.5 10.0 9.5 9.0 8.5 8.0 7.5 7.0 6.5 6.0 5.5 5.0 4.5 4.0 3.5 3.0 2.5 2.0 1.5 1.0 0.5 0.
f1 (ppm)

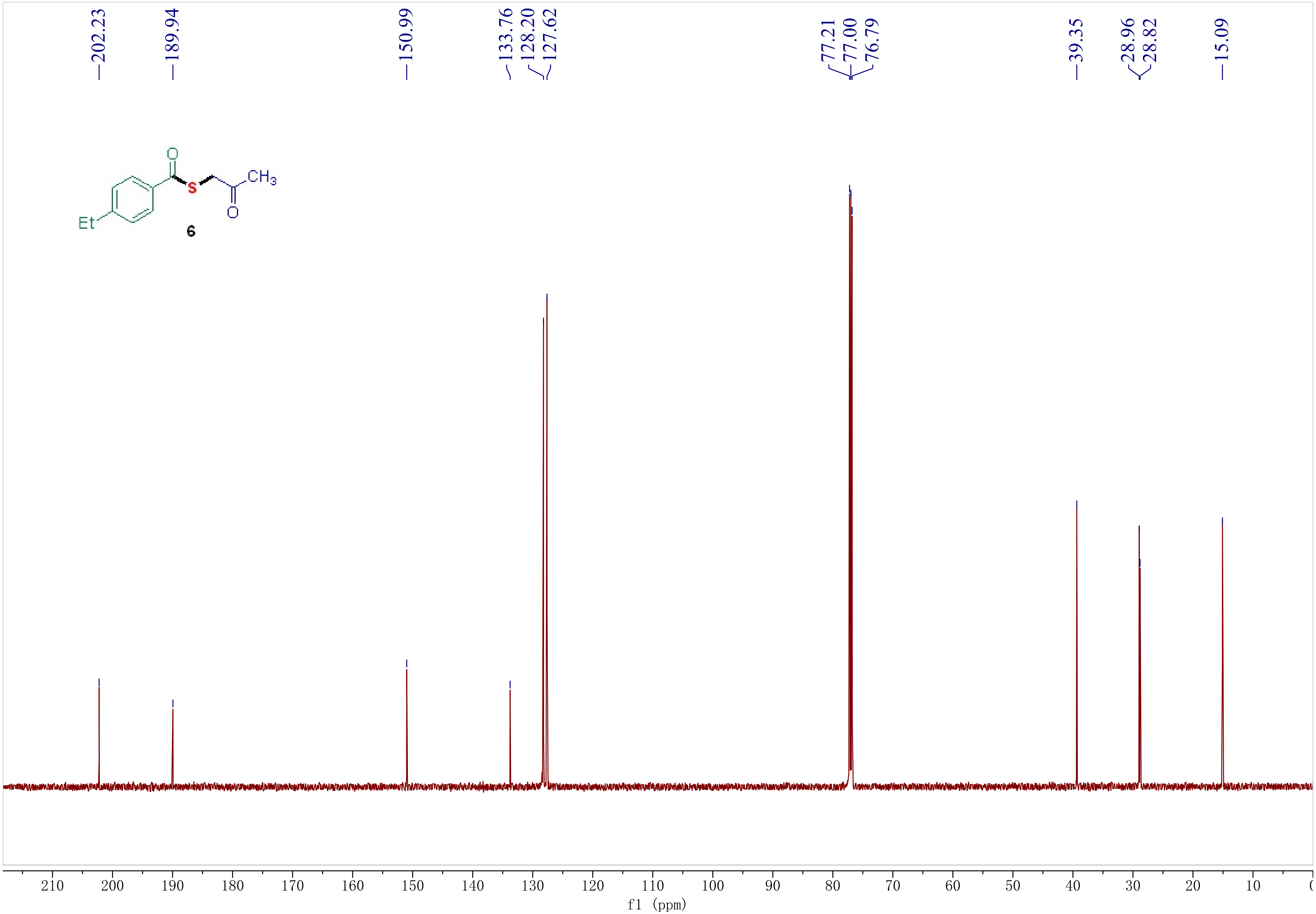

202.23
189.94
150.99
133.76
128.20
127.62
77.21
77.00
76.79
39.35
28.96
28.82
15.09
CH3
Et
S
O
6
210
200
190
180
170
160
150
140
130
120
110
100
90
80
70
60
50
40
30
20
10
f1 (ppm)

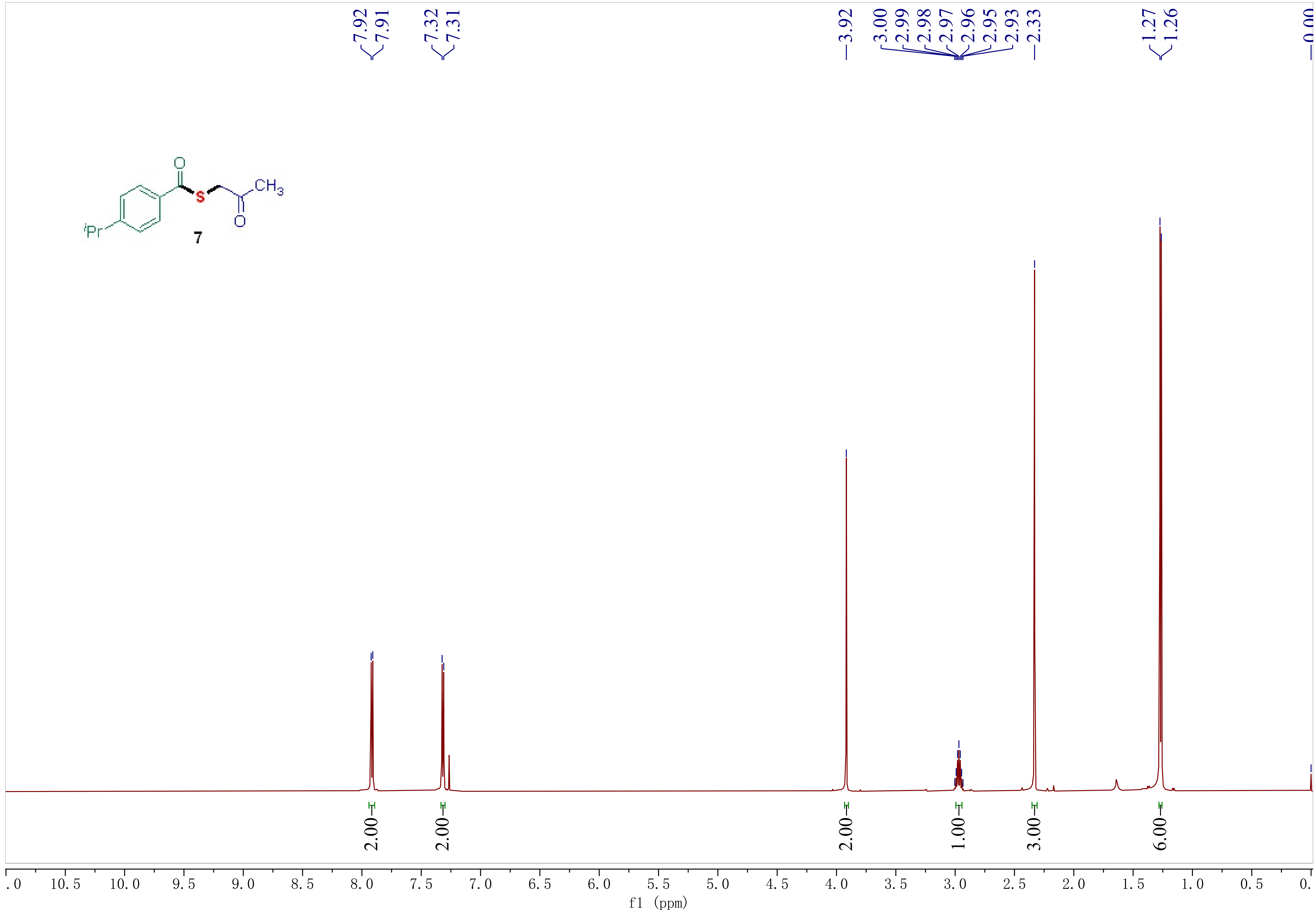
7.92
7.91
7.32
7.31
3.92
3.00
2.99
2.98
2.97
2.96
2.95
2.93
2.33
1.27
1.26
0.00
O
S
CH3
O
iPr
7
2.00
2.00
2.00
1.00
3.00
6.00
10.5
10.0
9.5
9.0
8.5
8.0
7.5
7.0
6.5
6.0
5.5
5.0
4.5
4.0
3.5
3.0
2.5
2.0
1.5
1.0
0.5
f1 (ppm)

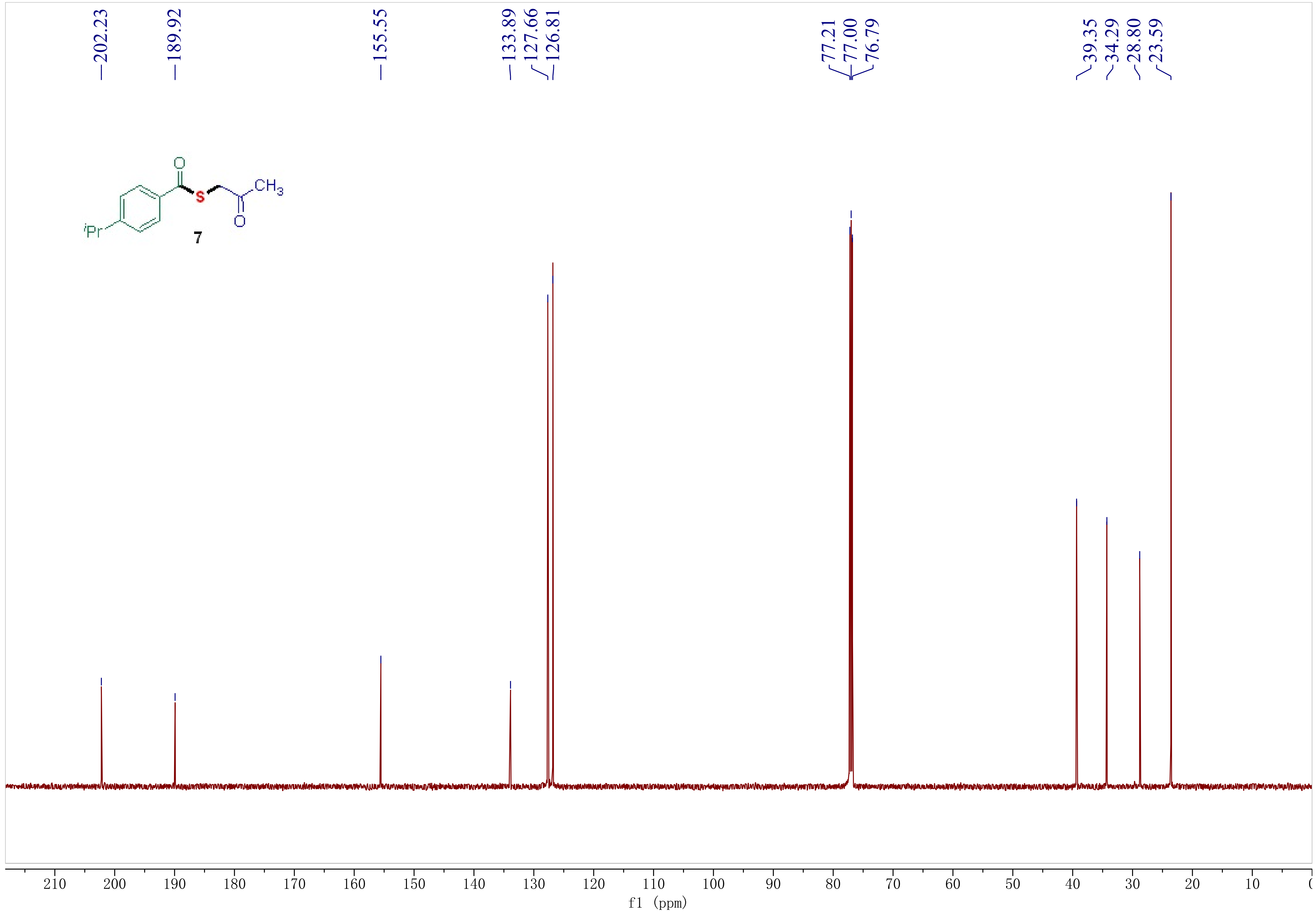

202.23
189.92
155.55
133.89
127.66
126.81
77.21
77.00
76.79
39.35
34.29
28.80
23.59
O
S
CH3
O
iPr
7
210
200
190
180
170
160
150
140
130
120
110
100
90
80
70
60
50
40
30
20
10
f1 (ppm)

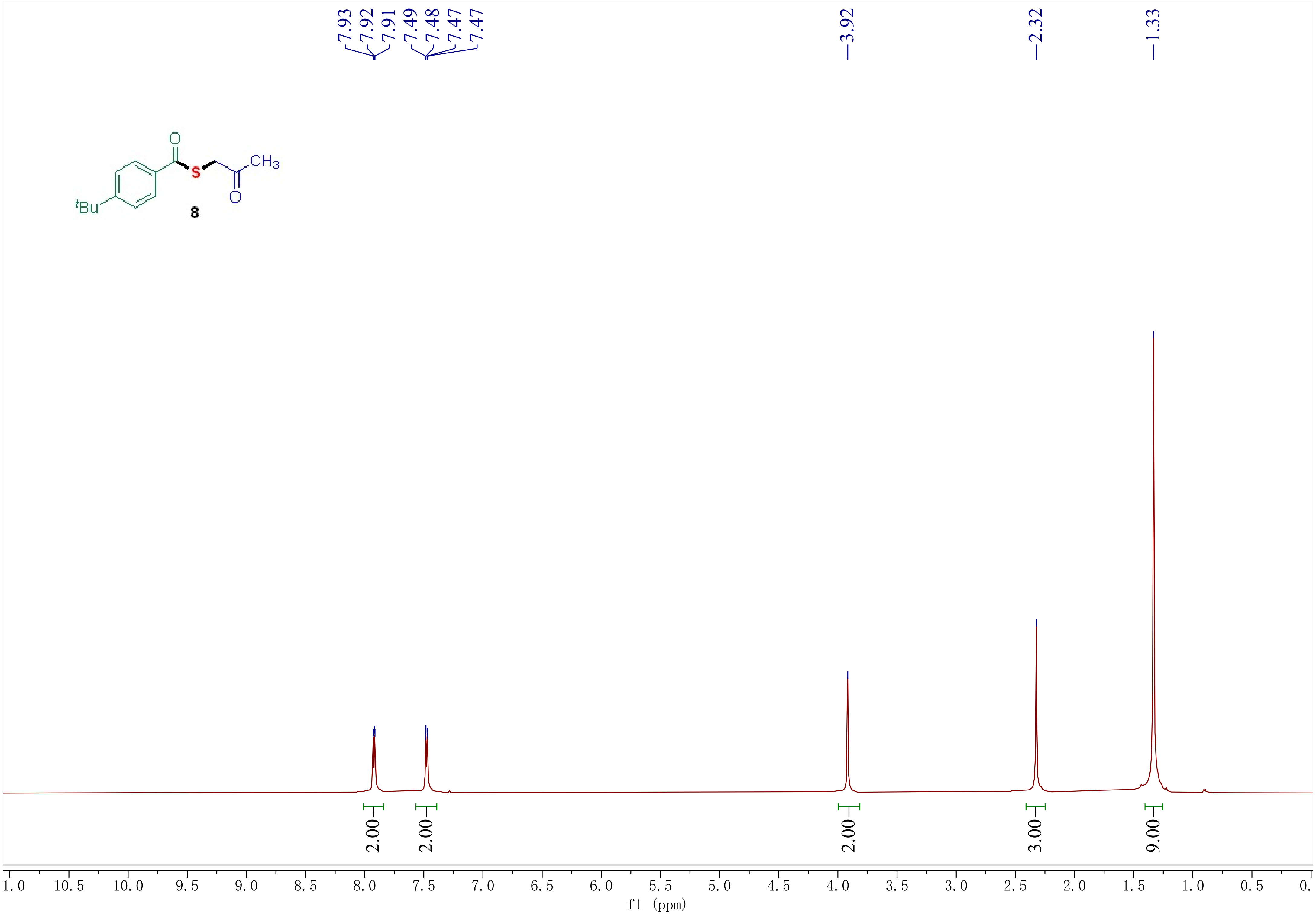
7.93
7.92
7.91
7.49
7.48
7.47
7.47
3.92
2.32
1.33
O
S
CH3
O
tBu
8
2.00
2.00
2.00
3.00
9.00
1.0
10.5
10.0
9.5
9.0
8.5
8.0
7.5
7.0
6.5
6.0
5.5
5.0
4.5
4.0
3.5
3.0
2.5
2.0
1.5
1.0
0.5
0.
f1 (ppm)

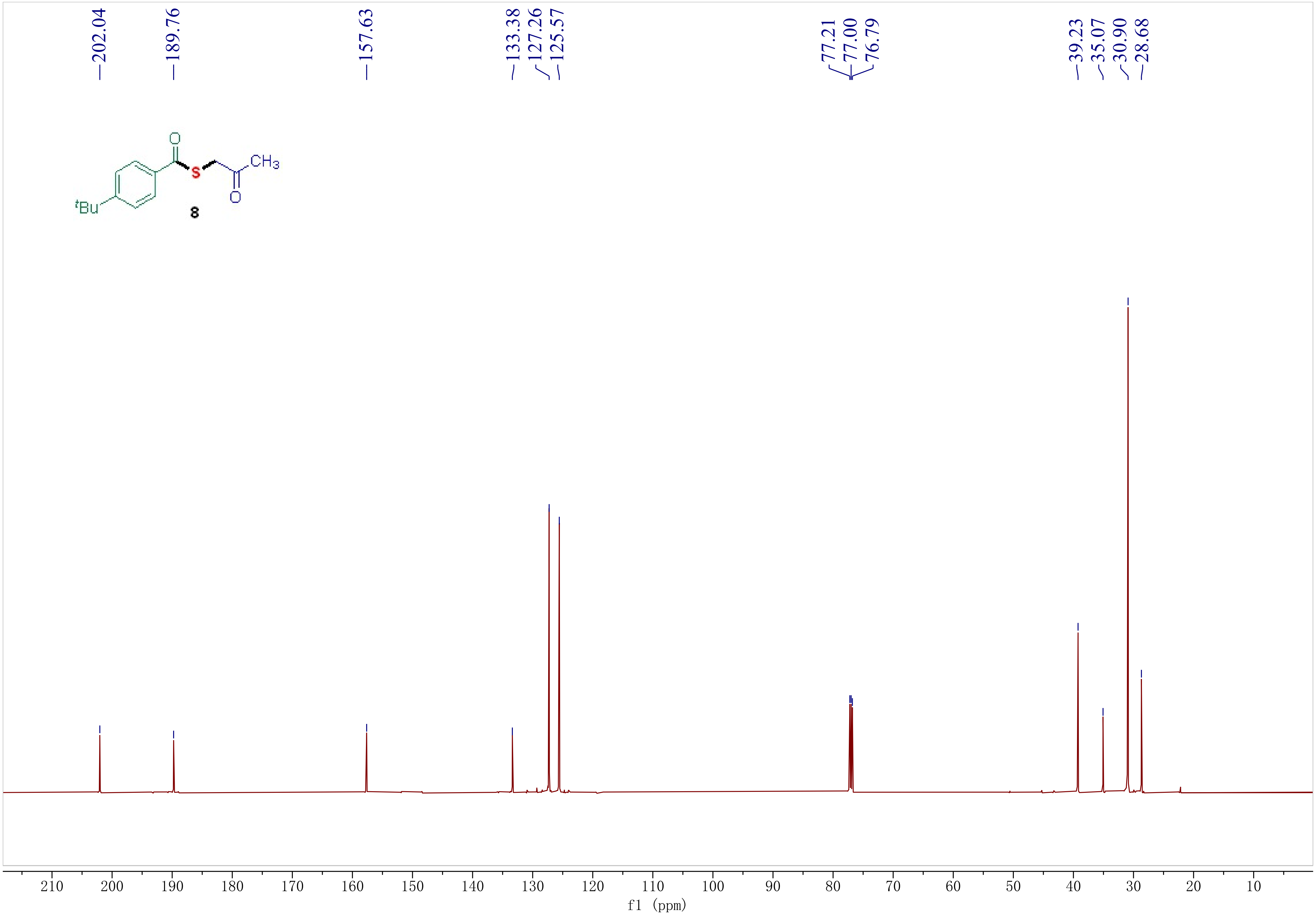

202.04
189.76
157.63
133.38
127.26
125.57
77.21
77.00
76.79
39.23
35.07
30.90
28.68
O
S
CH3
O
tBu
8
210 200 190 180 170 160 150 140 130 120 110 100 90 80 70 60 50 40 30 20 10
f1 (ppm)

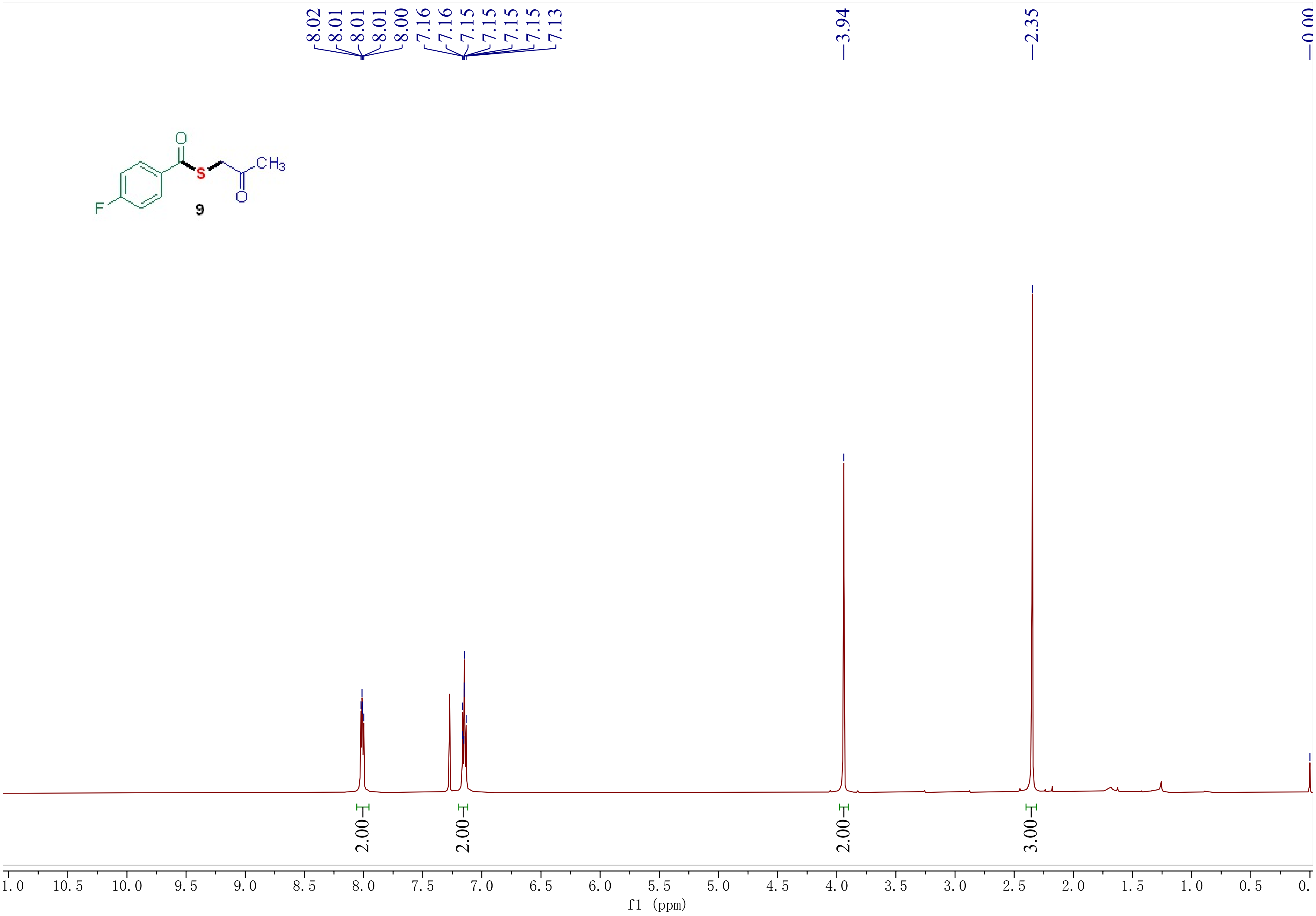
8.02
8.01
8.01
8.01
8.00
7.16
7.16
7.15
7.15
7.15
7.15
7.13
3.94
2.35
0.00
O
S
CH3
O
F
9
2.00
2.00
2.00
3.00
1.0
10.5
10.0
9.5
9.0
8.5
8.0
7.5
7.0
6.5
6.0
5.5
5.0
4.5
4.0
3.5
3.0
2.5
2.0
1.5
1.0
0.5
0.
f1 (ppm)

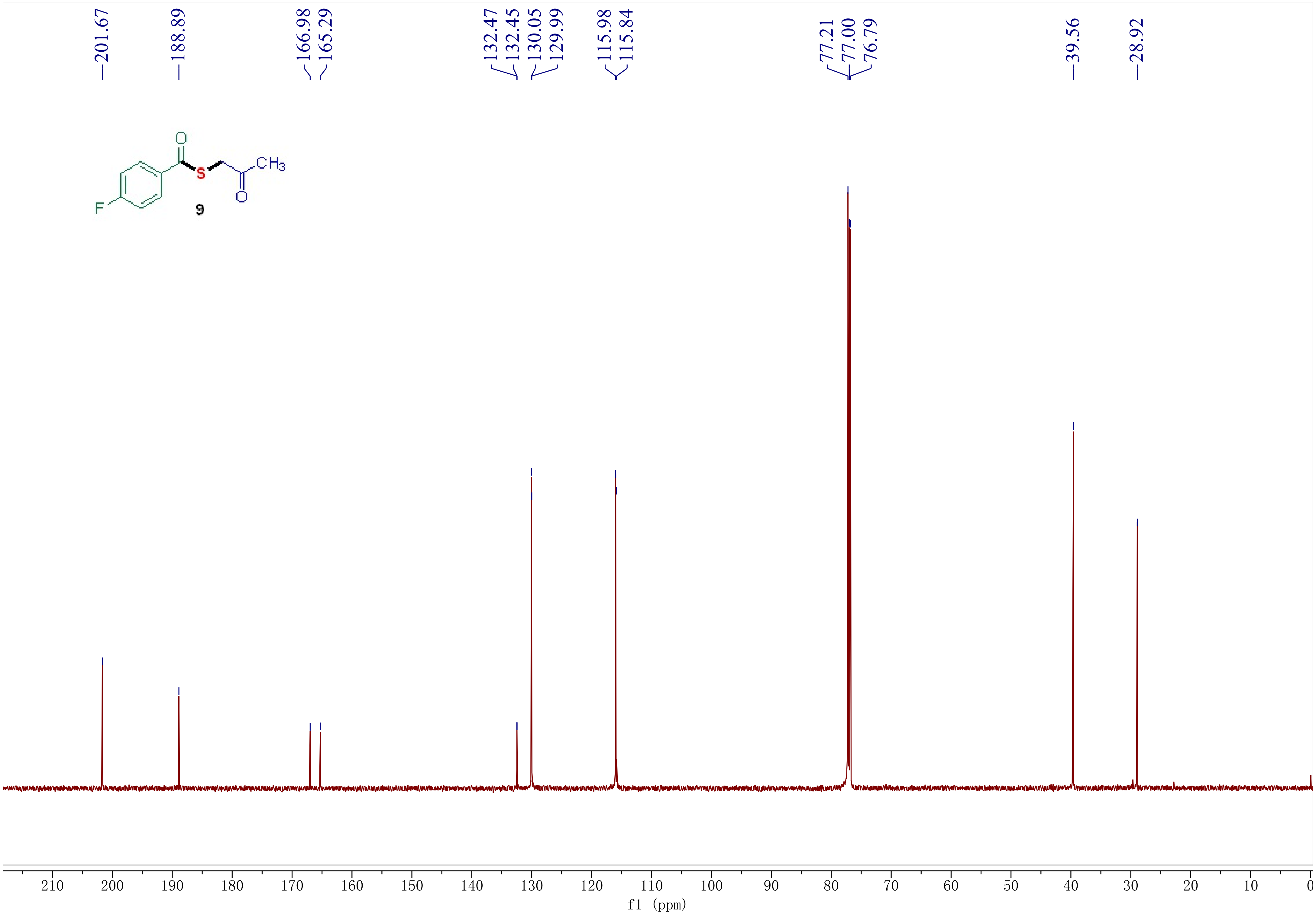

201.67
188.89
166.98
165.29
132.47
132.45
130.05
129.99
115.98
115.84
77.21
77.00
76.79
39.56
28.92
O
S
CH3
O
F
9
210
200
190
180
170
160
150
140
130
120
110
100
90
80
70
60
50
40
30
20
10
0
f1 (ppm)

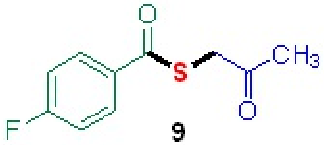

9

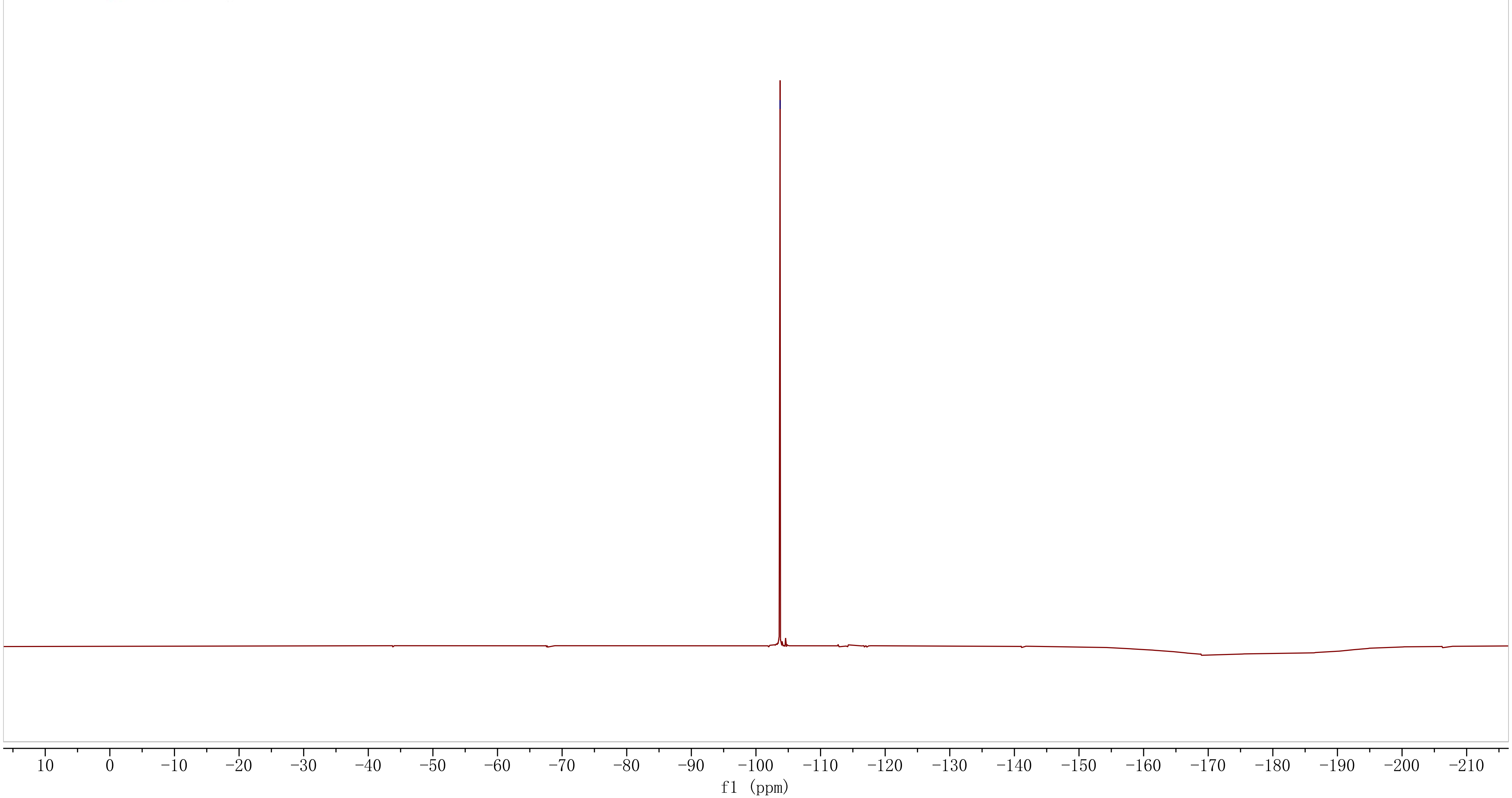

-103.73
10
0
−10
−20
−30
−40
−50
−60
−70
−80
−90
−100
−110
−120
−130
−140
−150
−160
−170
−180
−190
−200
−210
f1 (ppm)

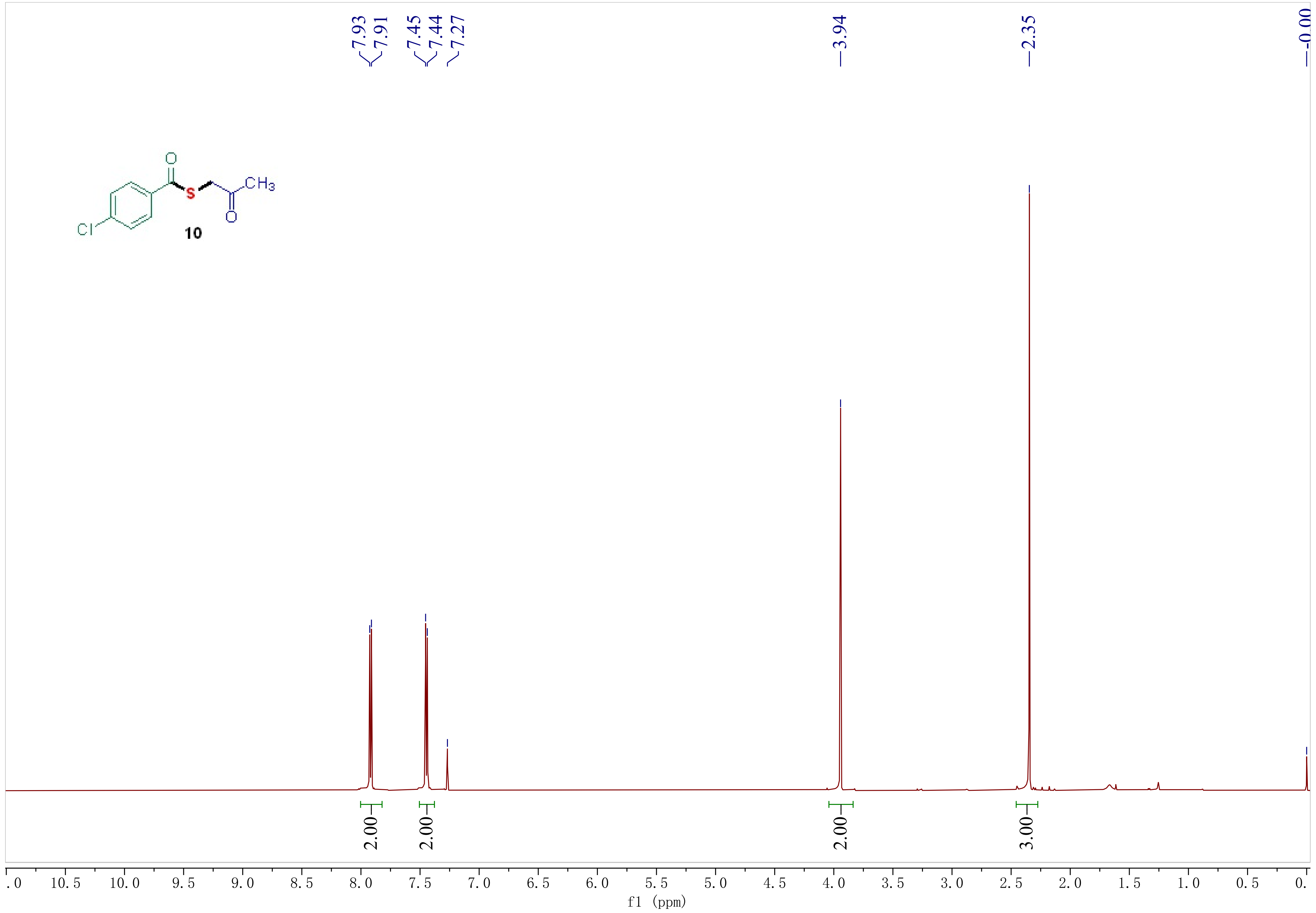
7.93
7.91
7.45
7.44
7.27
3.94
2.35
-0.00
O
S
CH3
O
Cl
10
2.00
2.00
2.00
3.00
.0
10.5
10.0
9.5
9.0
8.5
8.0
7.5
7.0
6.5
6.0
5.5
5.0
4.5
4.0
3.5
3.0
2.5
2.0
1.5
1.0
0.5
0.
f1 (ppm)

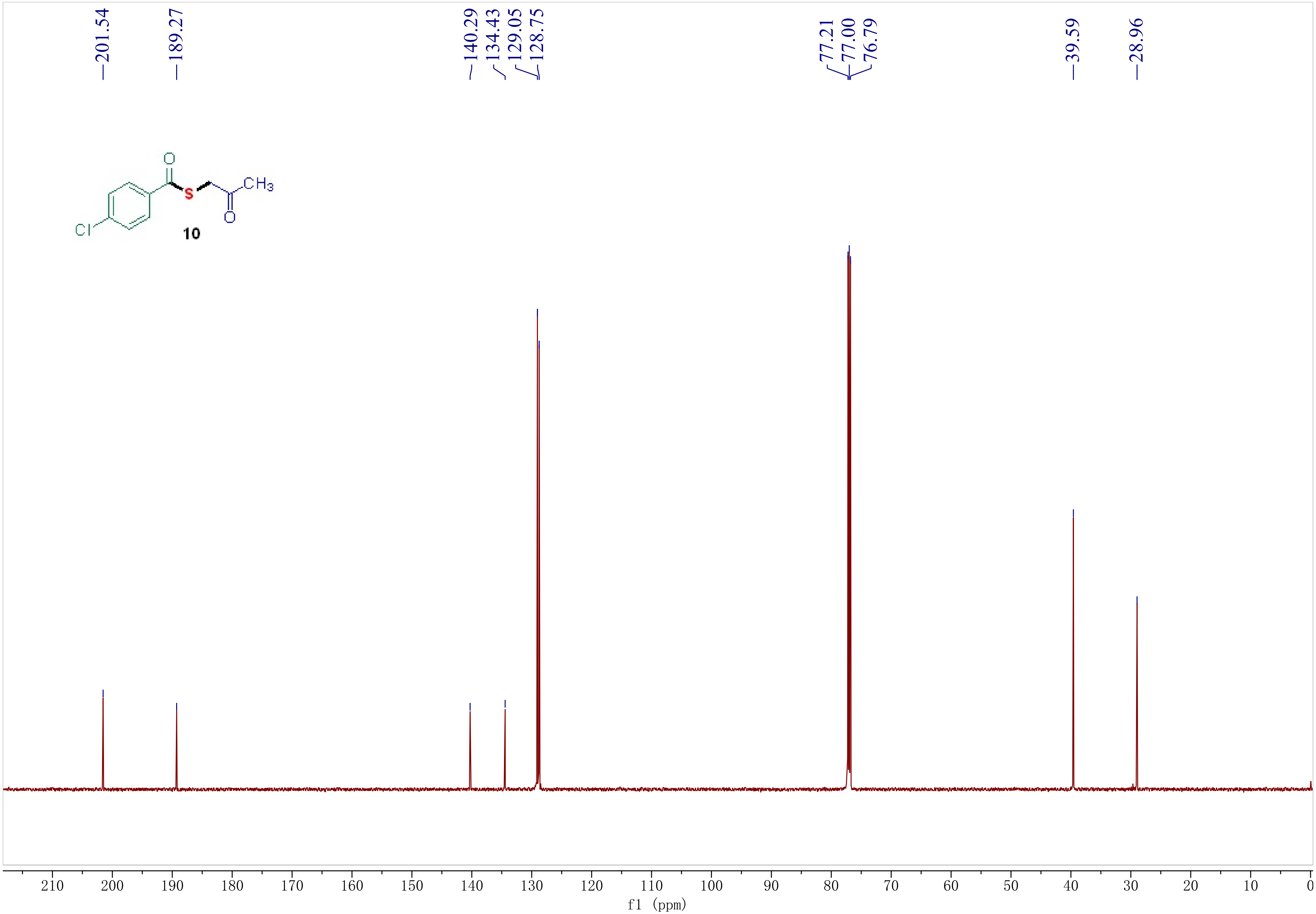

201.54
189.27
140.29
134.43
129.05
128.75
77.21
77.00
76.79
39.59
28.96
O
S
CH3
O
Cl
10
210 200 190 180 170 160 150 140 130 120 110 100 90 80 70 60 50 40 30 20 10 0
f1 (ppm)

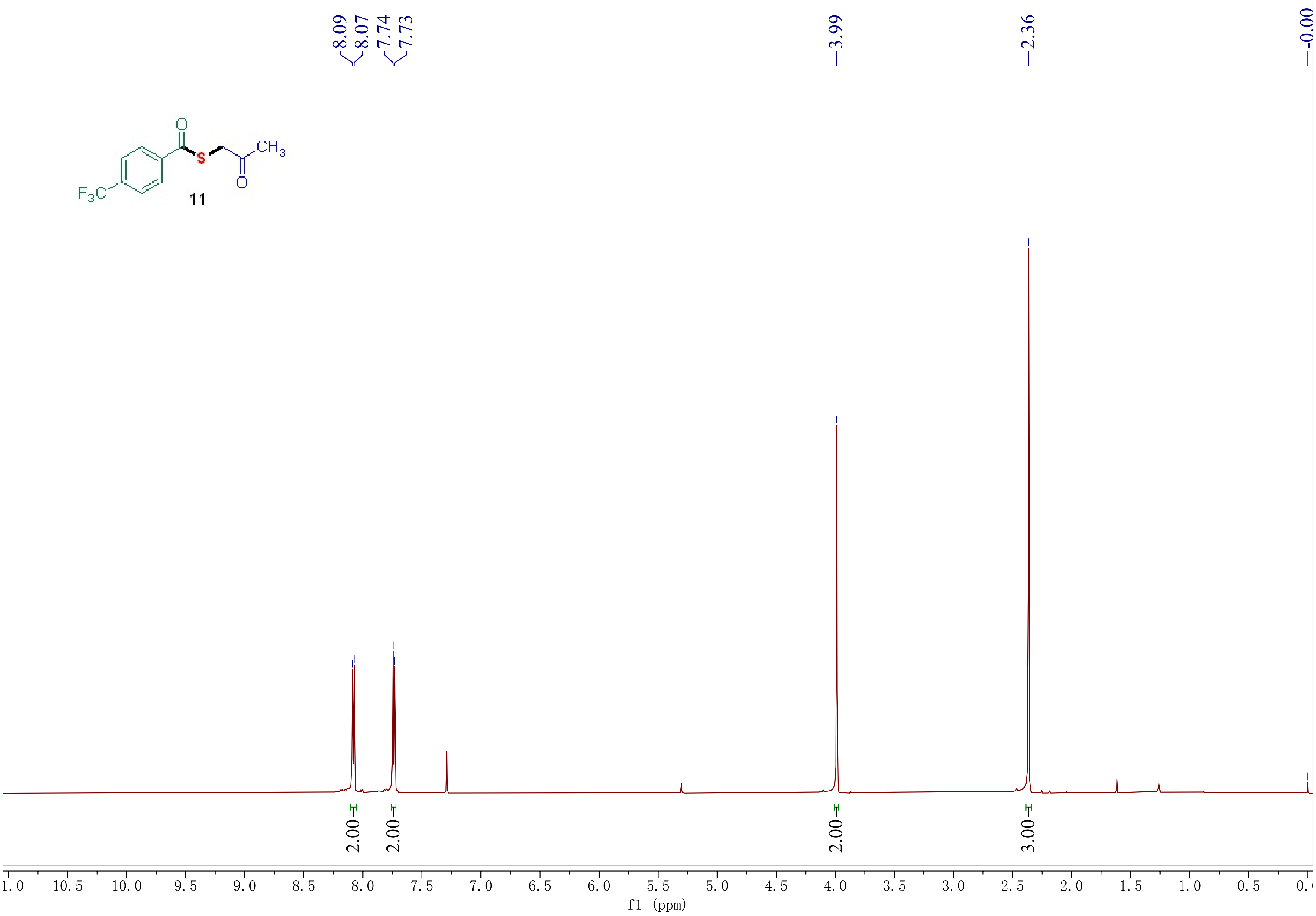
8.09
8.07
7.74
7.73
3.99
2.36
-0.00
O
S
CH3
F3C
O
11
2.00
2.00
2.00
3.00
1.0
10.5
10.0
9.5
9.0
8.5
8.0
7.5
7.0
6.5
6.0
5.5
5.0
4.5
4.0
3.5
3.0
2.5
2.0
1.5
1.0
0.5
0.0
f1 (ppm)

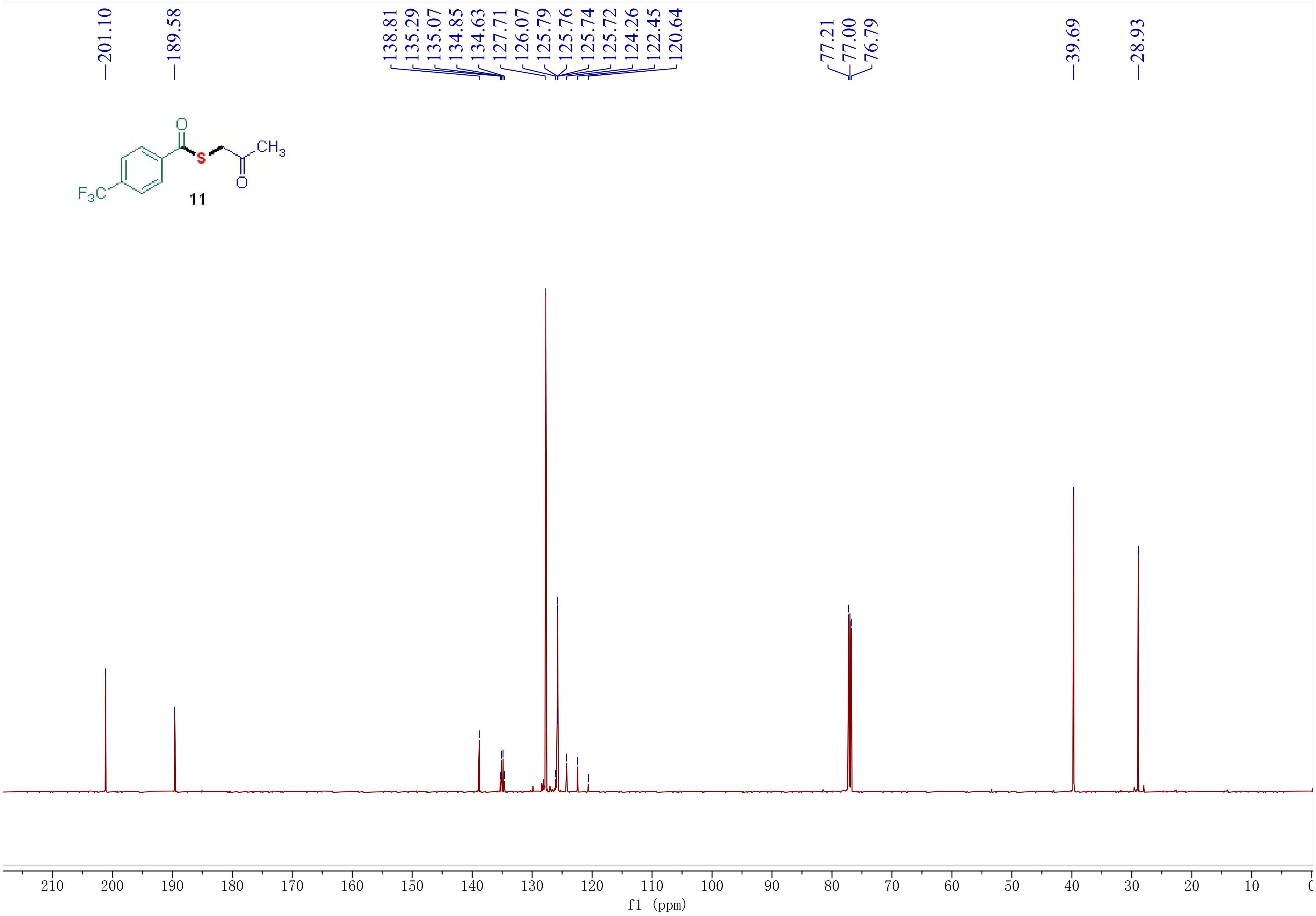

201.10
189.58
138.81
135.29
135.07
134.85
134.63
127.71
126.07
125.79
125.76
125.74
125.72
124.26
122.45
120.64
77.21
77.00
76.79
39.69
28.93
O
S
CH3
O
F3C
11
210 200 190 180 170 160 150 140 130 120 110 100 90 80 70 60 50 40 30 20 10 0
f1 (ppm)

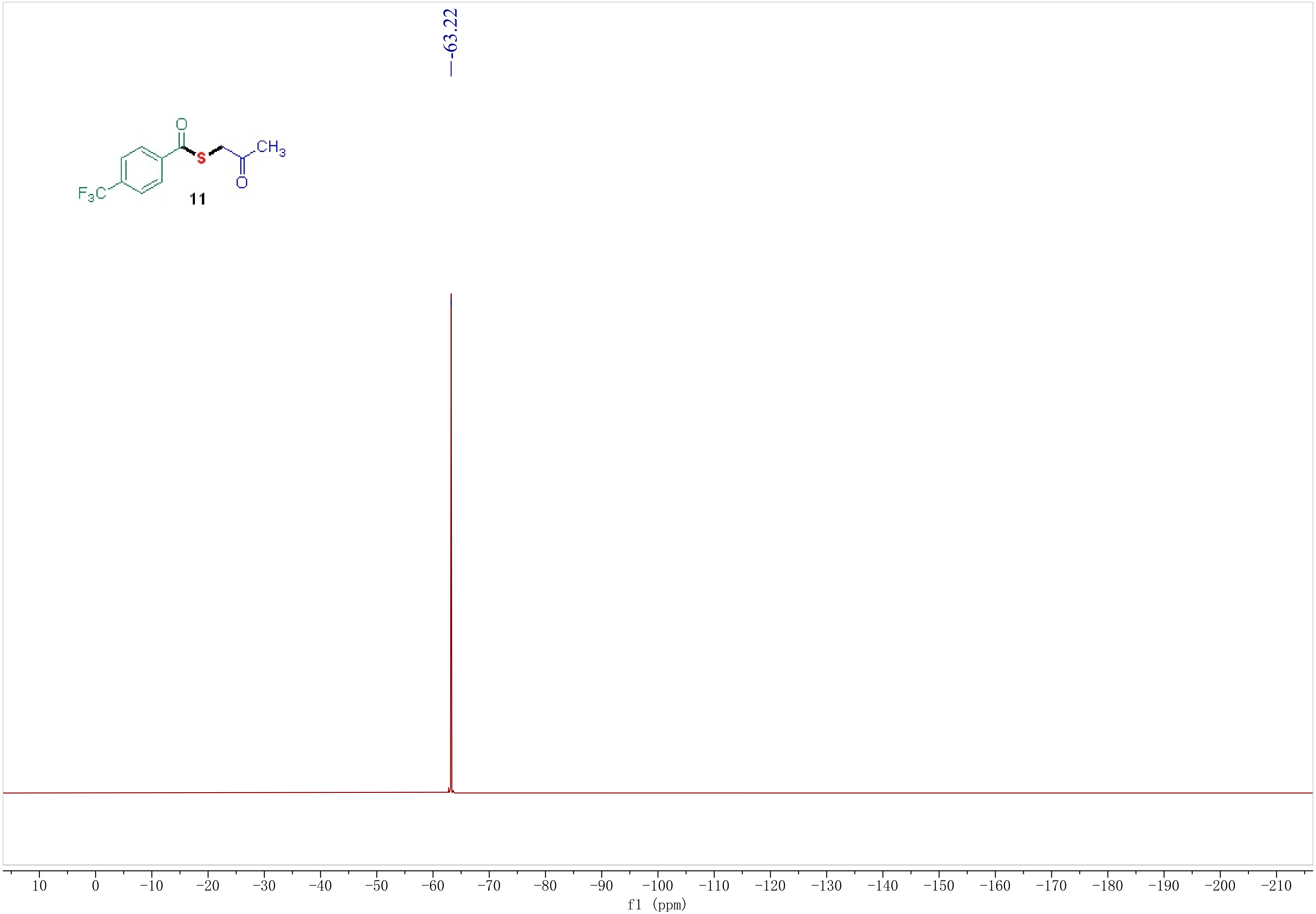

-63.22
F3C
O
S
CH3
O
11
10 0 −10 −20 −30 −40 −50 −60 −70 −80 −90 −100 −110 −120 −130 −140 −150 −160 −170 −180 −190 −200 −210
f1 (ppm)

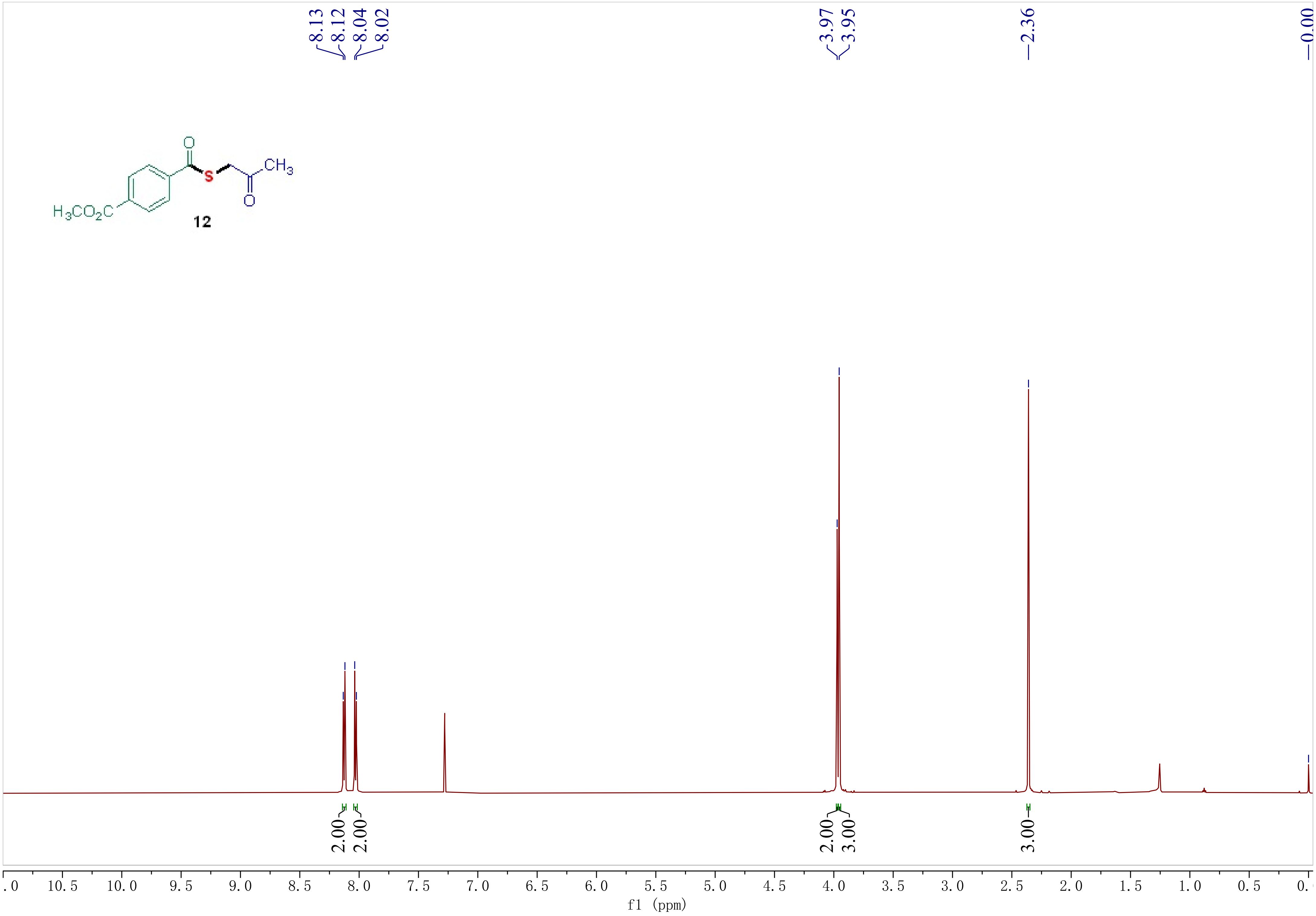

8.13
8.12
8.04
8.02
3.97
3.95
2.36
0.00
O
S
CH3
O
H3CO2C
12
2.00
2.00
2.00
3.00
3.00
10.5
10.0
9.5
9.0
8.5
8.0
7.5
7.0
6.5
6.0
5.5
5.0
4.5
4.0
3.5
3.0
2.5
2.0
1.5
1.0
0.5
f1 (ppm)

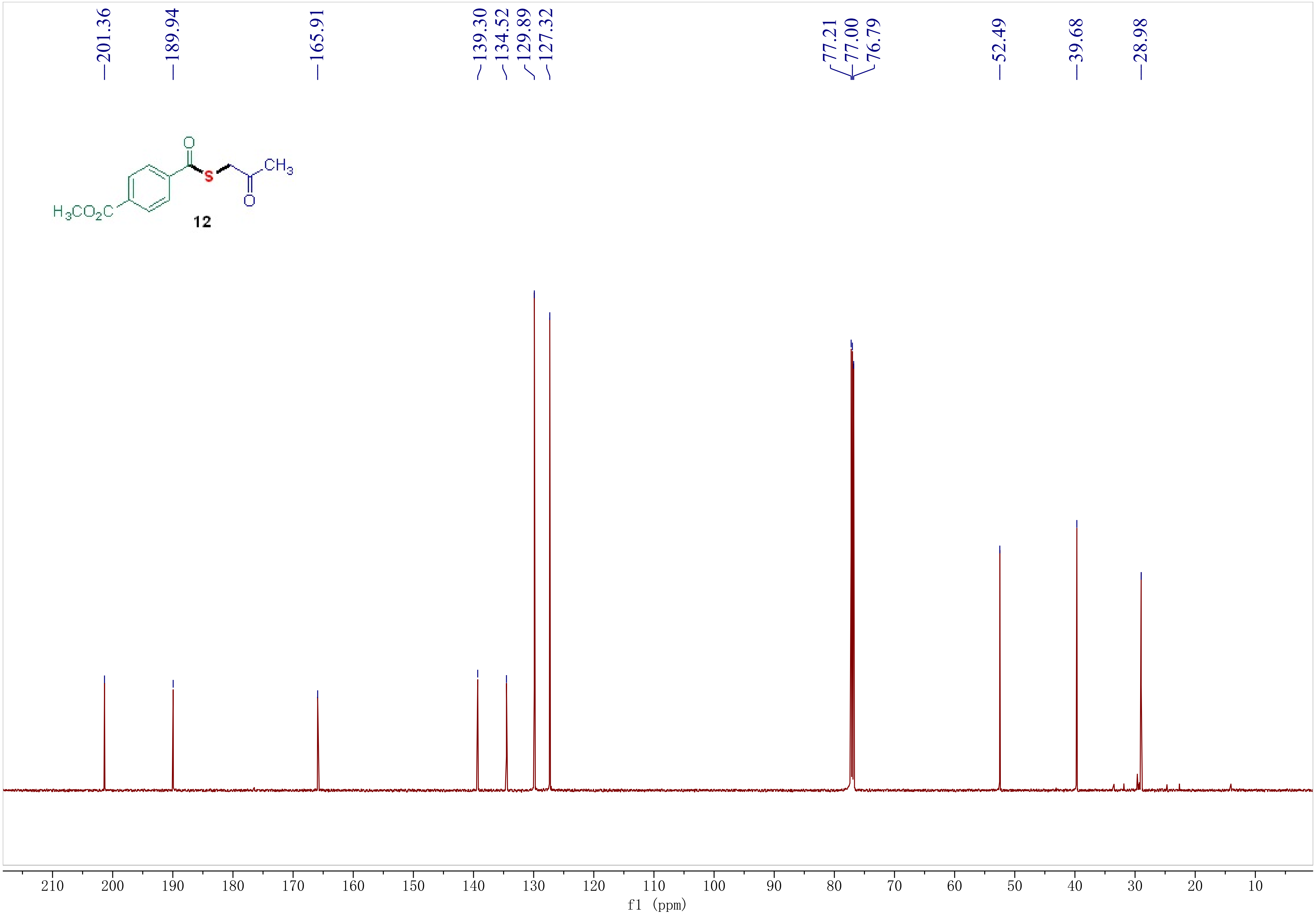

201.36
189.94
165.91
139.30
134.52
129.89
127.32
77.21
77.00
76.79
52.49
39.68
28.98
H_3CO_2C
S
O
CH_3
12
210
200
190
180
170
160
150
140
130
120
110
100
90
80
70
60
50
40
30
20
10
f1 (ppm)

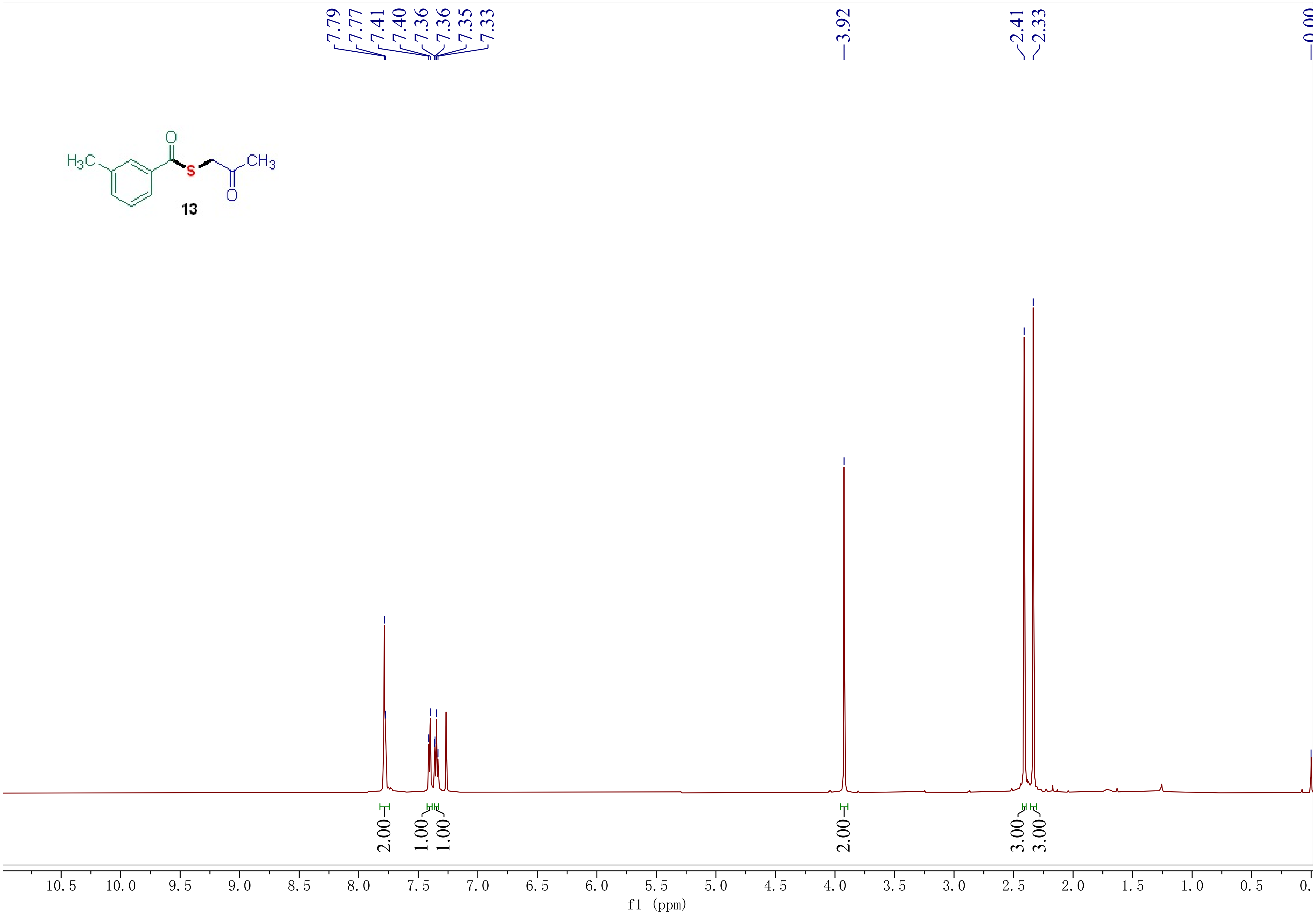
7.79
7.77
7.41
7.40
7.36
7.36
7.35
7.33
3.92
2.41
2.33
0.00
H3C
O
S
CH3
O
13
2.00
1.00
1.00
2.00
3.00
3.00
10.5
10.0
9.5
9.0
8.5
8.0
7.5
7.0
6.5
6.0
5.5
5.0
4.5
4.0
3.5
3.0
2.5
2.0
1.5
1.0
0.5
0.
f1 (ppm)

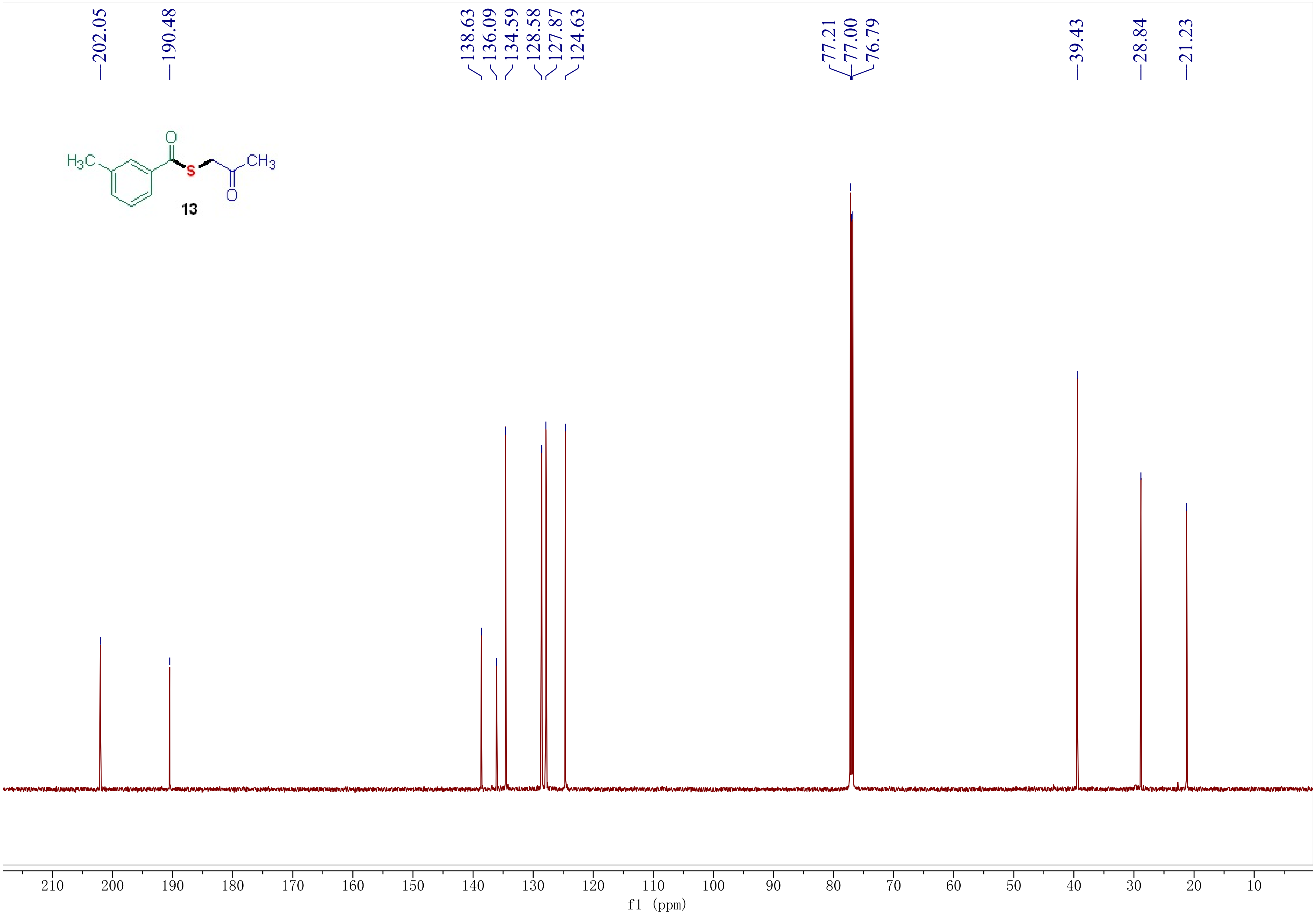

202.05
190.48
138.63
136.09
134.59
128.58
127.87
124.63
77.21
77.00
76.79
39.43
28.84
21.23
H3C
O
S
CH3
O
13
210 200 190 180 170 160 150 140 130 120 110 100 90 80 70 60 50 40 30 20 10
f1 (ppm)

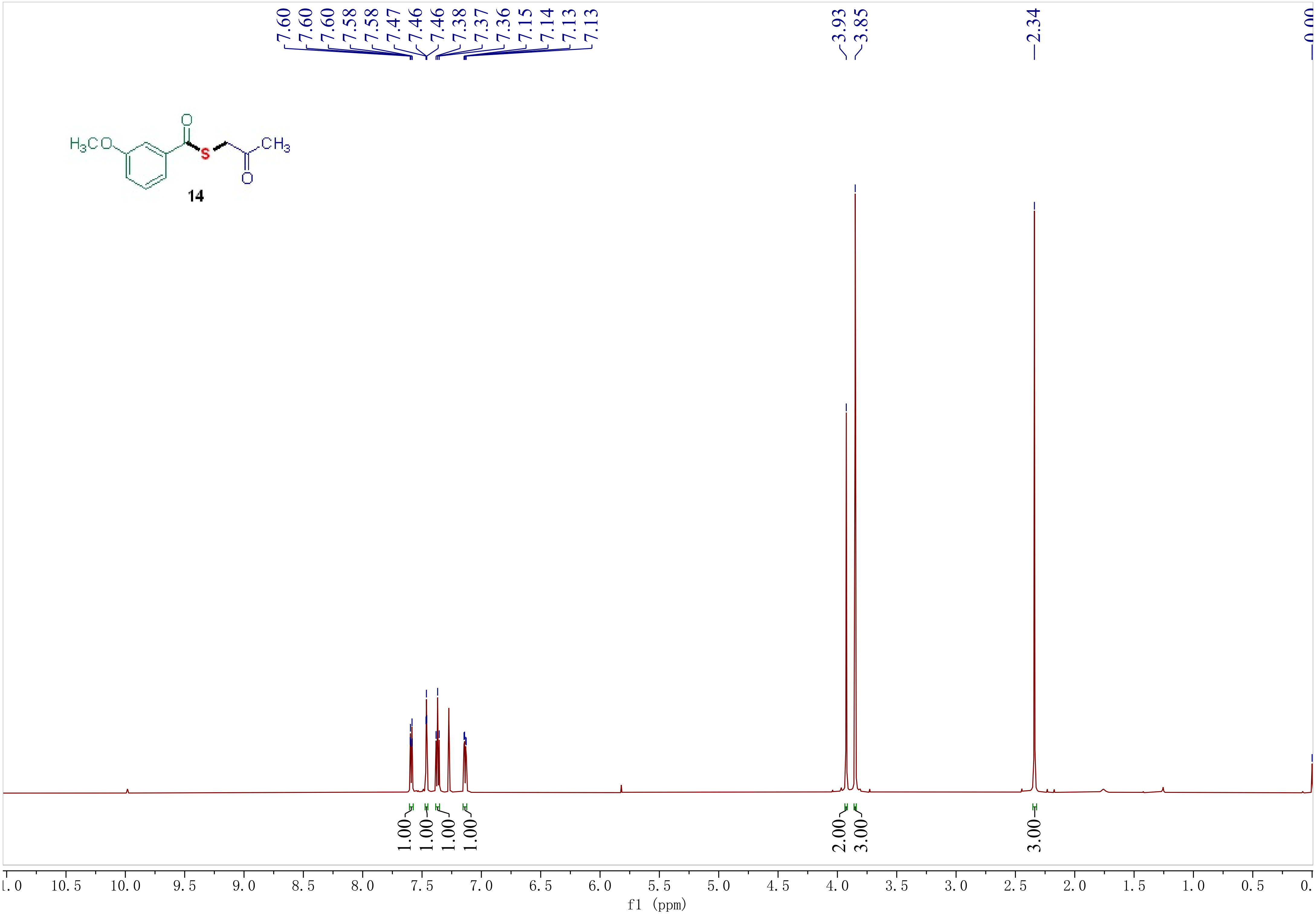

7.60
7.60
7.60
7.58
7.58
7.47
7.46
7.46
7.38
7.37
7.36
7.15
7.14
7.13
7.13
3.93
3.85
2.34
0.00
H3CO
O
S
CH3
O
14
1.00
1.00
1.00
1.00
2.00
3.00
3.00
10.5
10.0
9.5
9.0
8.5
8.0
7.5
7.0
6.5
6.0
5.5
5.0
4.5
4.0
3.5
3.0
2.5
2.0
1.5
1.0
0.5
f1 (ppm)

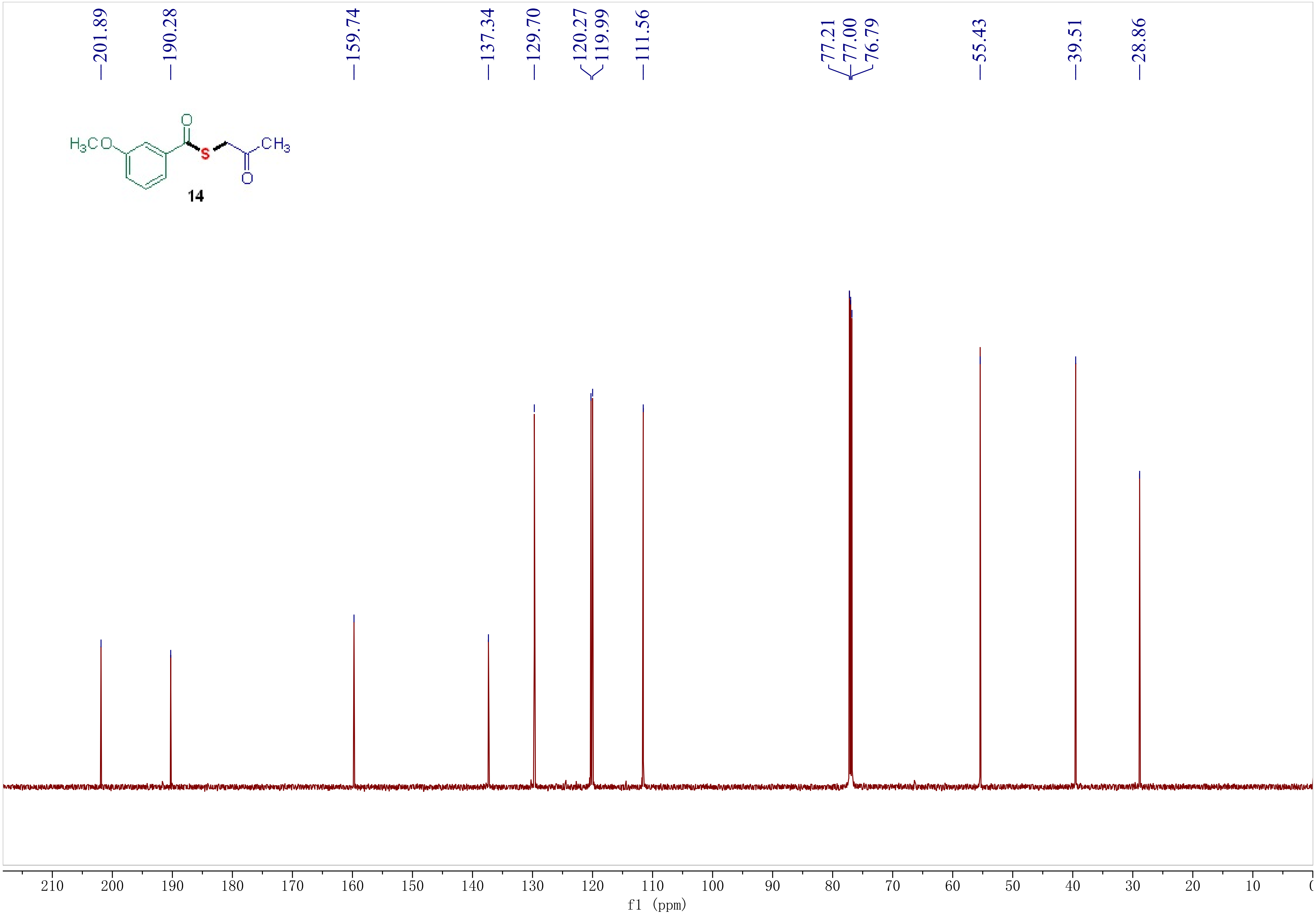

201.89
190.28
159.74
137.34
129.70
120.27
119.99
111.56
77.21
77.00
76.79
55.43
39.51
28.86
H3CO
O
S
CH3
O
14
210
200
190
180
170
160
150
140
130
120
110
100
90
80
70
60
50
40
30
20
10
f1 (ppm)

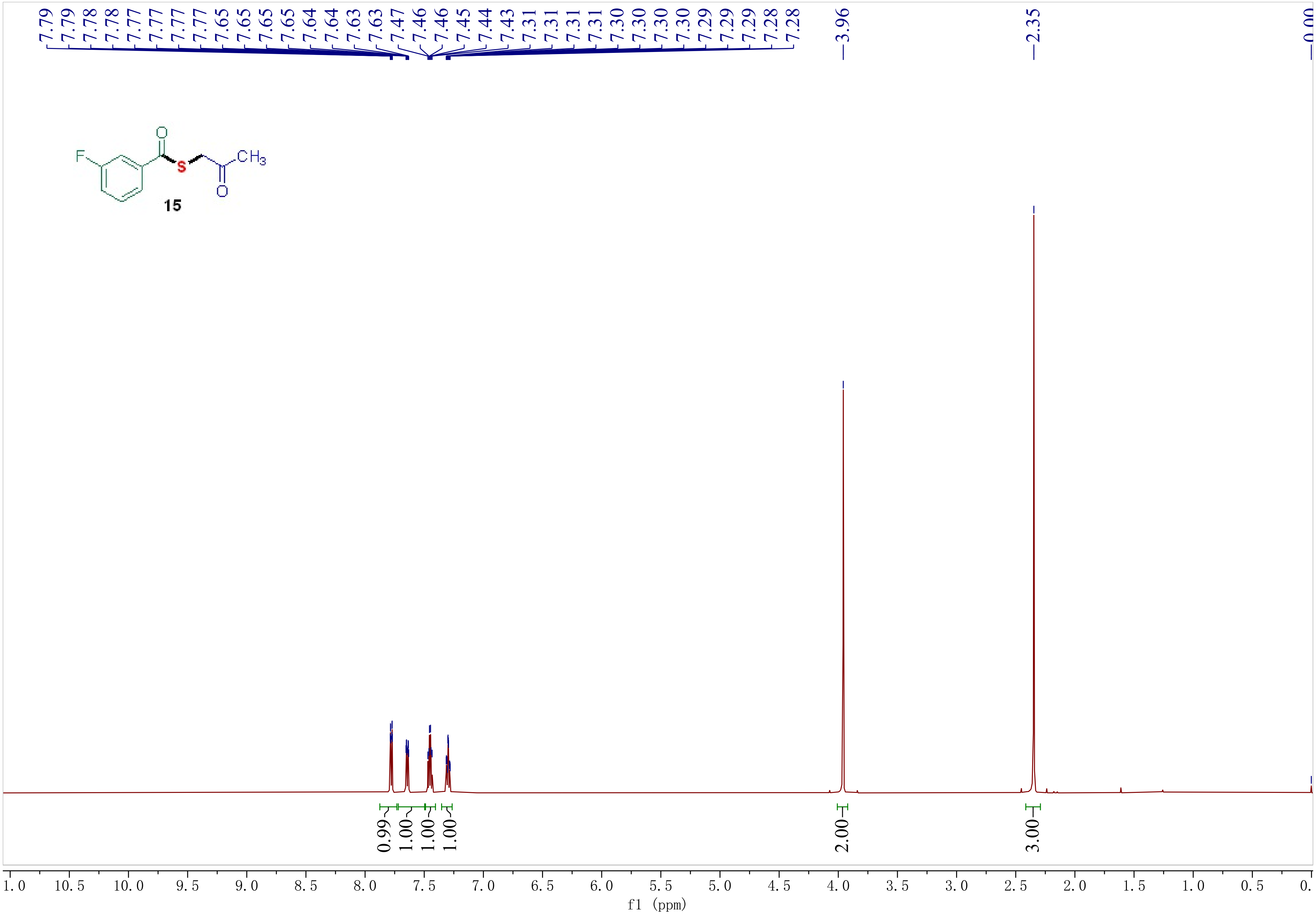
7.79 7.79 7.78 7.78 7.77 7.77 7.77 7.77 7.65 7.65 7.65 7.65 7.64 7.64 7.63 7.63 7.47 7.46 7.46 7.45 7.44 7.43 7.31 7.31 7.31 7.31 7.30 7.30 7.30 7.30 7.29 7.29 7.29 7.28 7.28
3.96
2.35
0.00
F
O
S
CH3
15
0.99
1.00
1.00
1.00
2.00
3.00
f1 (ppm)

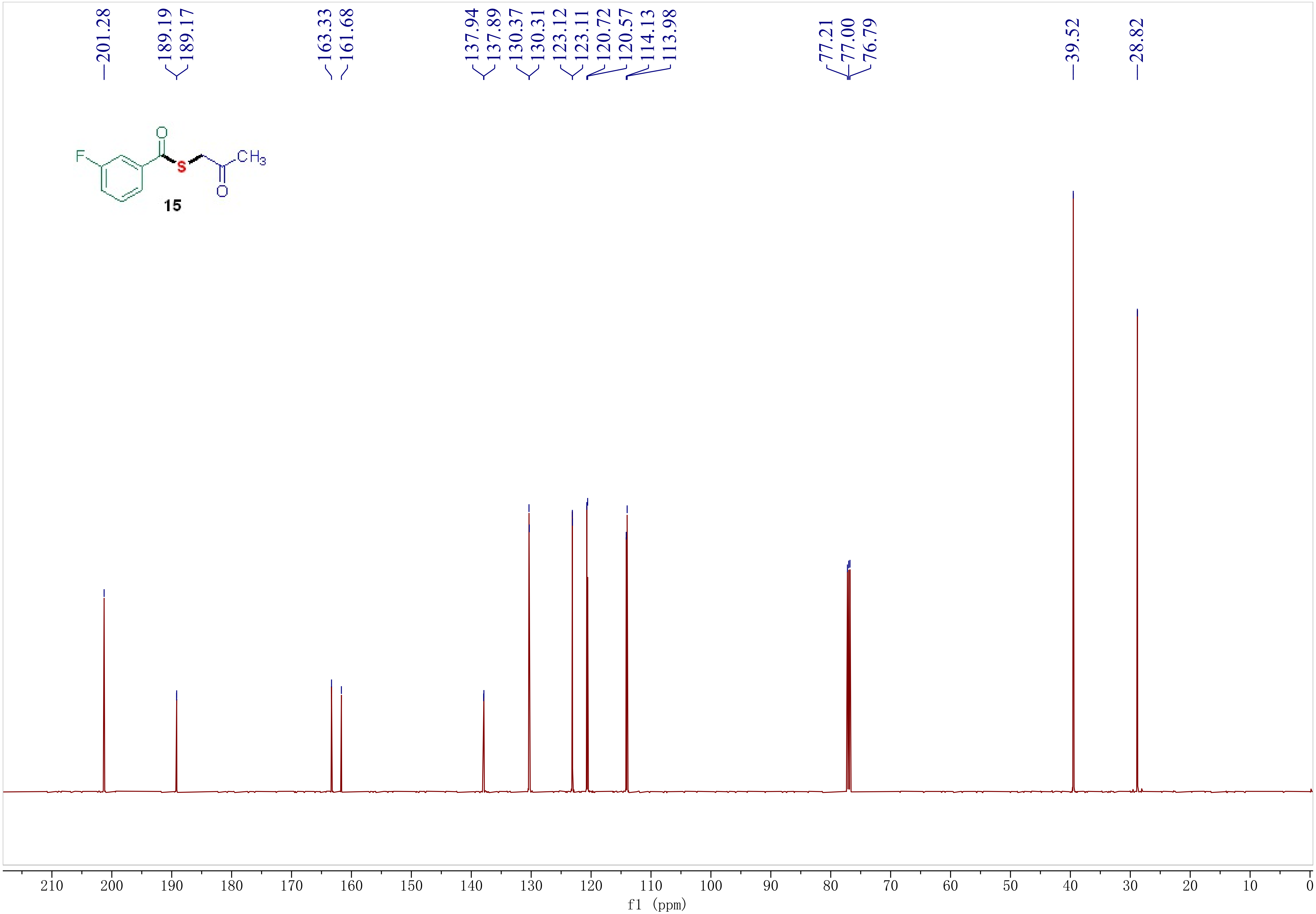
201.28
189.19
189.17
163.33
161.68
137.94
137.89
130.37
130.31
123.12
123.11
120.72
120.57
114.13
113.98
77.21
77.00
76.79
39.52
28.82
F
O
S
O
CH3
15
210
200
190
180
170
160
150
140
130
120
110
100
90
80
70
60
50
40
30
20
10
0
f1 (ppm)

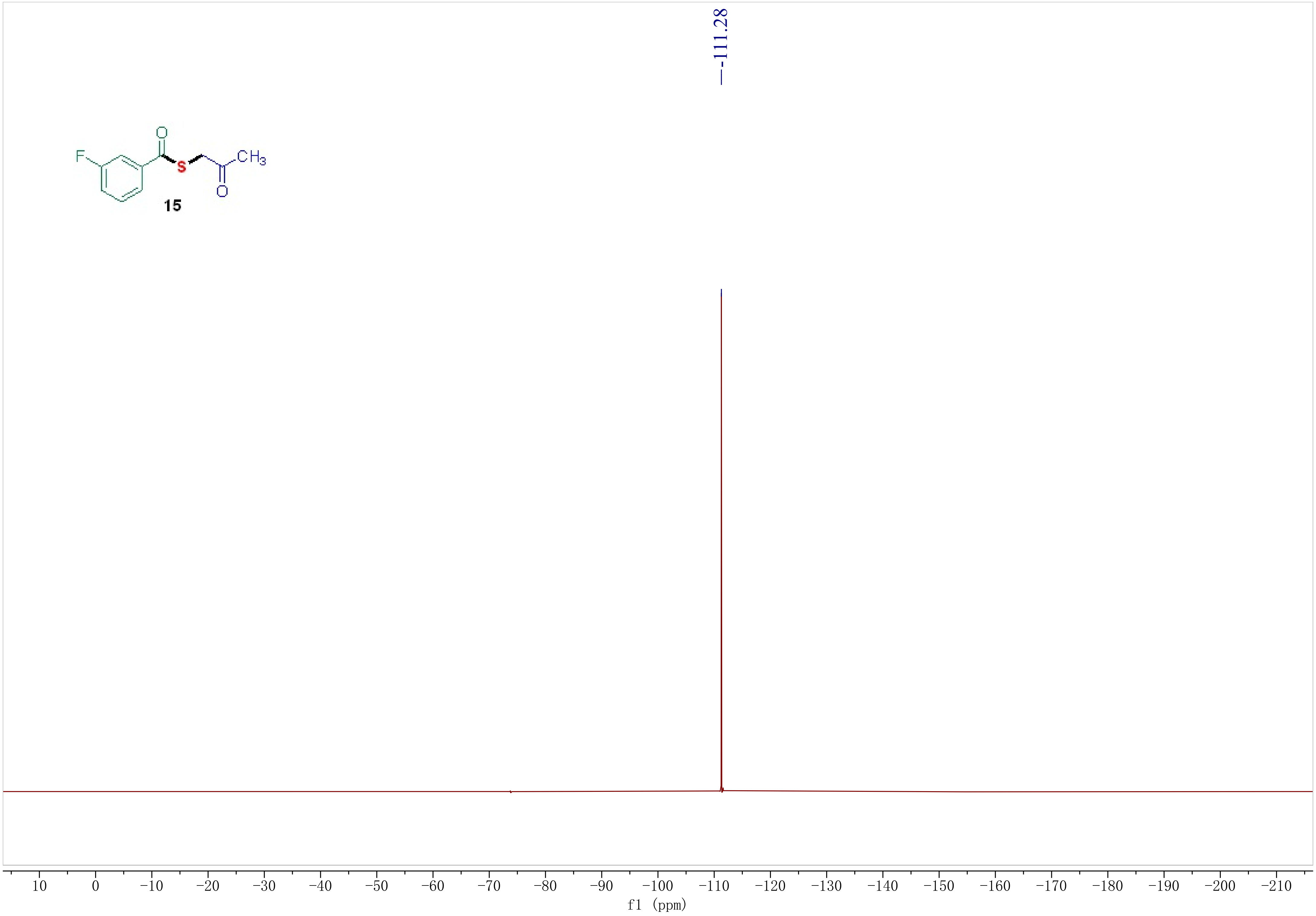

-111.28
15
10
0
−10
−20
−30
−40
−50
−60
−70
−80
−90
−100
−110
−120
−130
−140
−150
−160
−170
−180
−190
−200
−210
f1 (ppm)

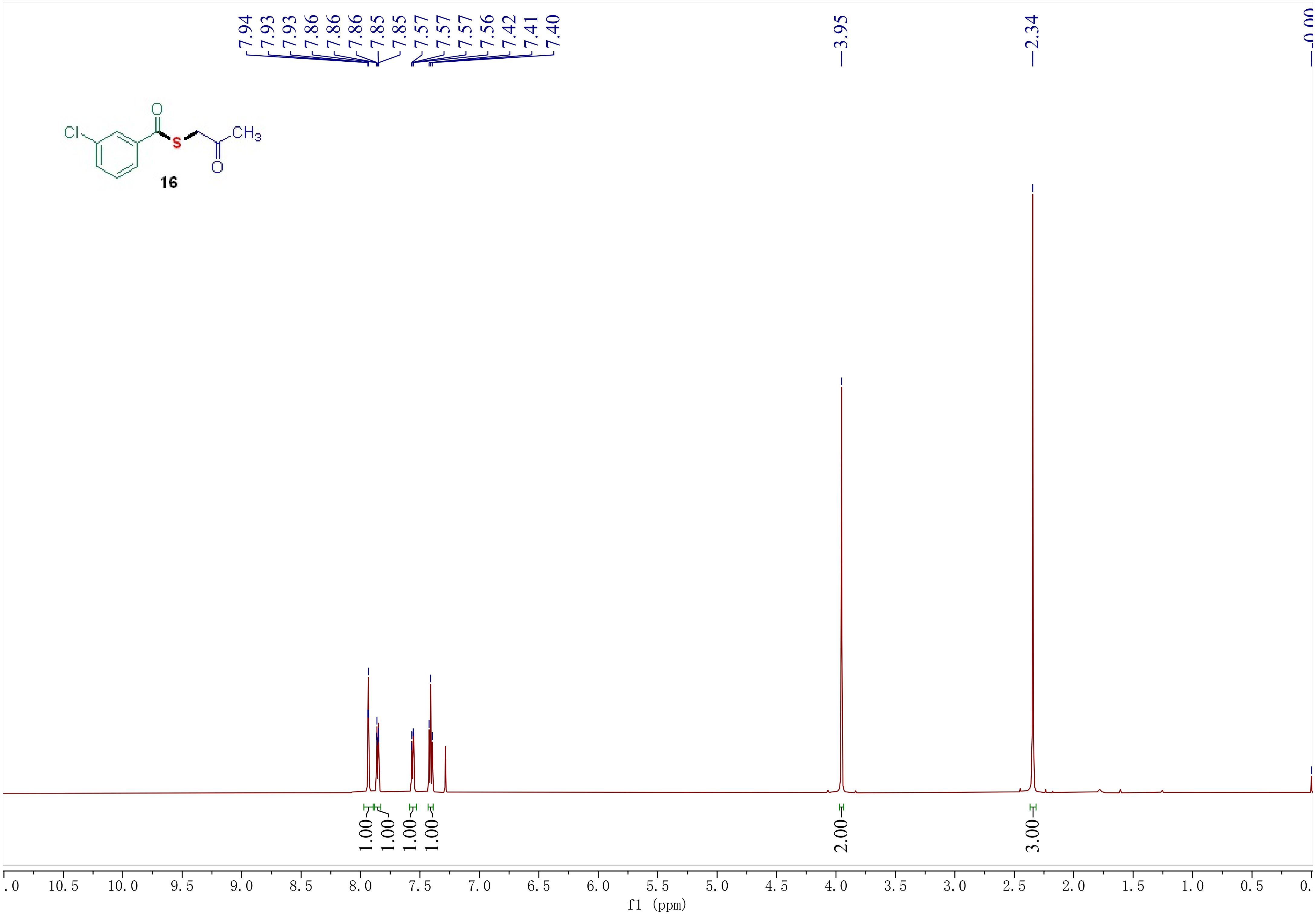

7.94
7.93
7.93
7.86
7.86
7.86
7.85
7.85
7.57
7.57
7.57
7.56
7.42
7.41
7.40
3.95
2.34
0.00
Cl
O
S
CH3
O
16
1.00
1.00
1.00
1.00
2.00
3.00
f1 (ppm)

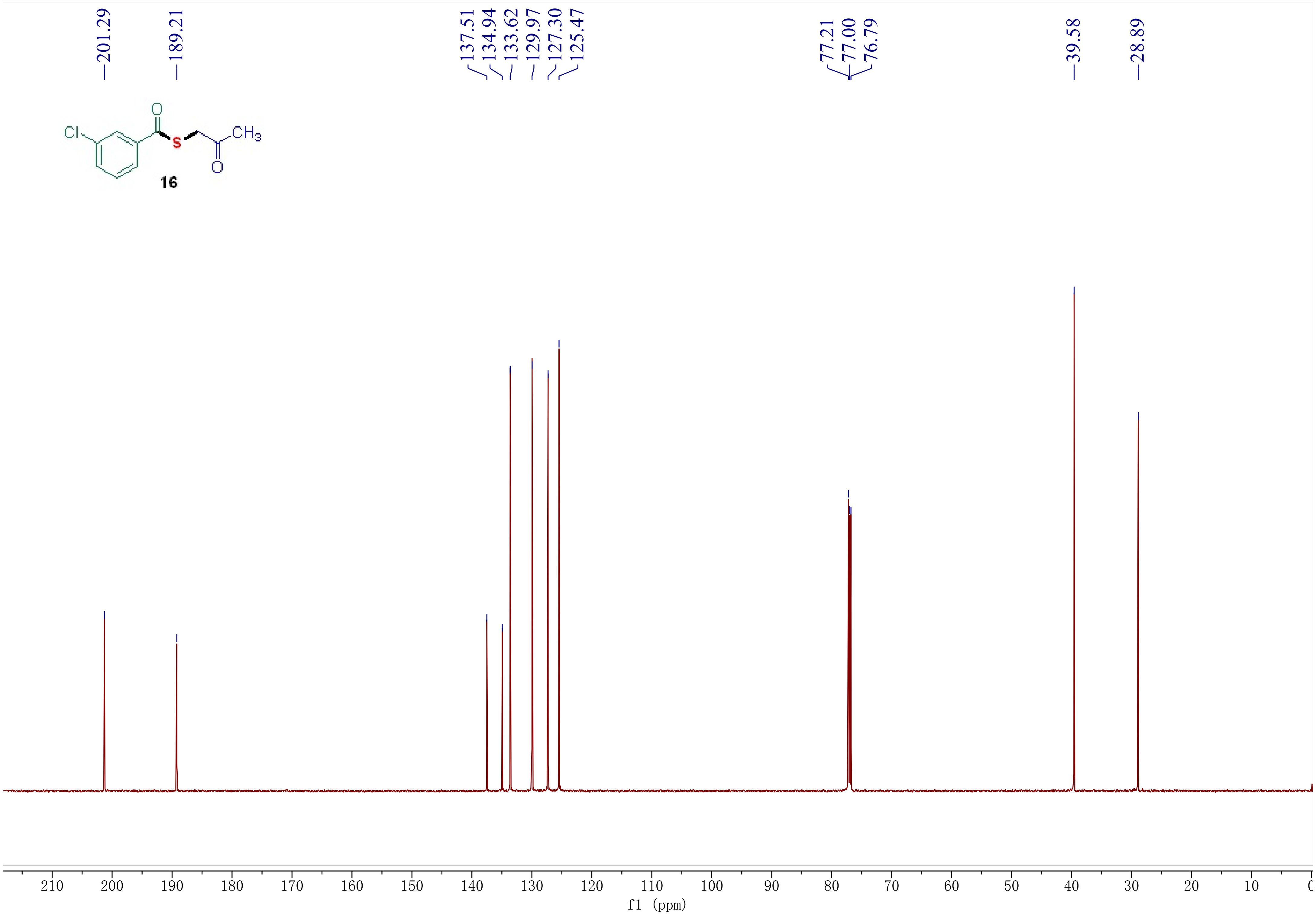
201.29
189.21
137.51
134.94
133.62
129.97
127.30
125.47
77.21
77.00
76.79
39.58
28.89
Cl
O
S
CH3
O
16
210
200
190
180
170
160
150
140
130
120
110
100
90
80
70
60
50
40
30
20
10
f1 (ppm)

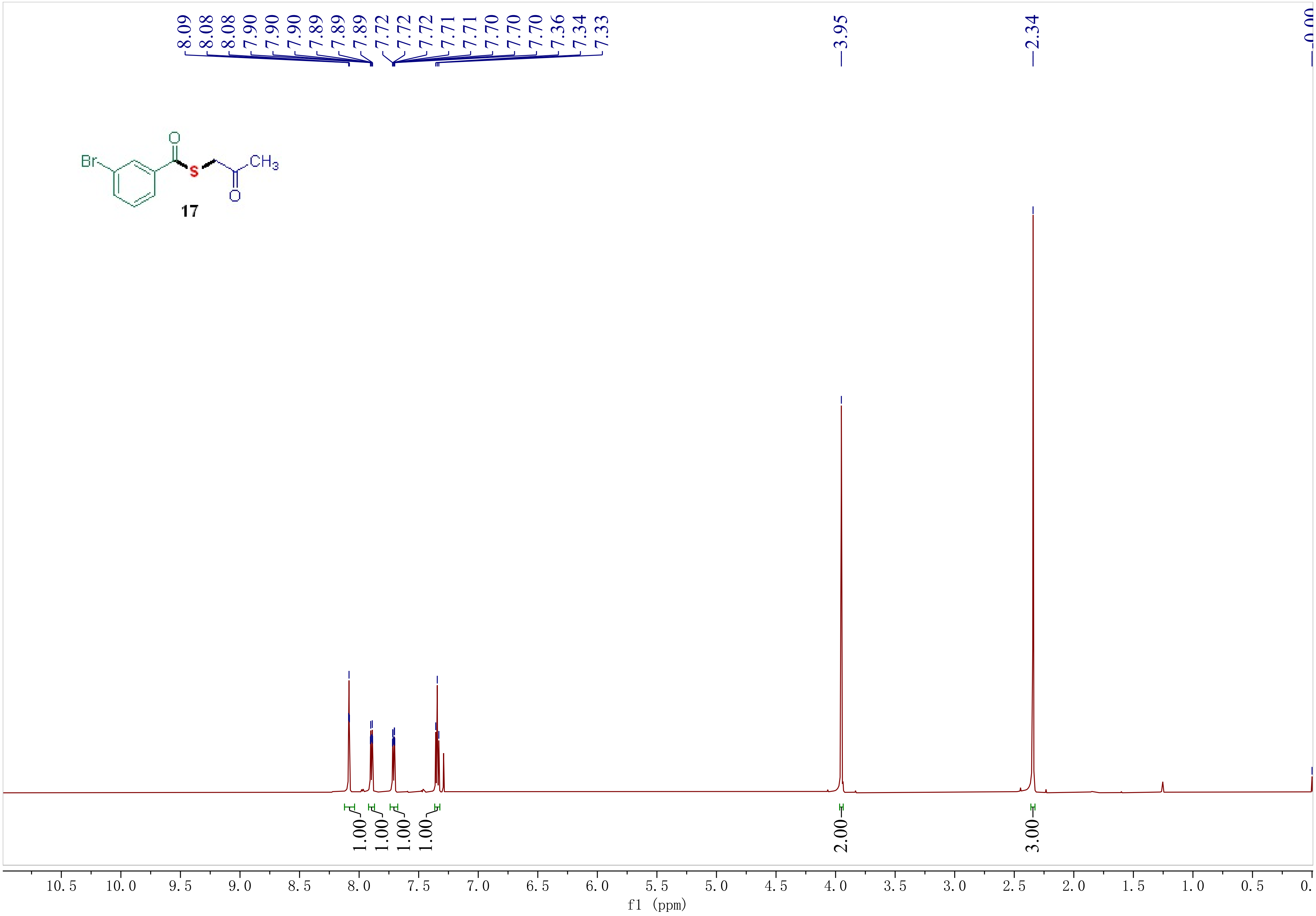
8.09
8.08
8.08
7.90
7.90
7.90
7.89
7.89
7.89
7.72
7.72
7.72
7.71
7.71
7.70
7.70
7.70
7.36
7.34
7.33
3.95
2.34
Br
O
S
O
CH3
17
1.00
1.00
1.00
1.00
2.00
3.00
10.5
10.0
9.5
9.0
8.5
8.0
7.5
7.0
6.5
6.0
5.5
5.0
4.5
4.0
3.5
3.0
2.5
2.0
1.5
1.0
0.5
f1 (ppm)

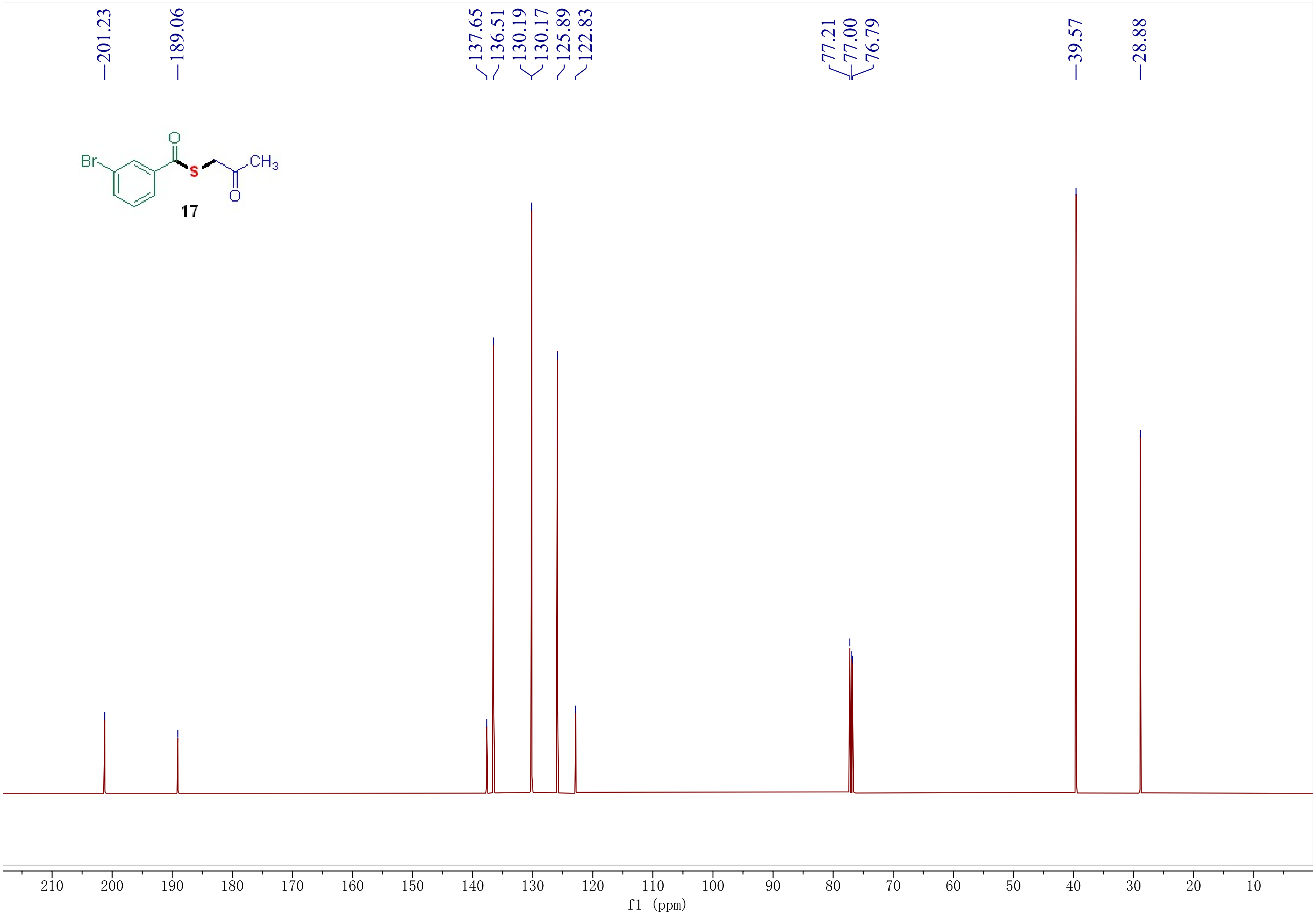
201.23
189.06
137.65
136.51
130.19
130.17
125.89
122.83
77.21
77.00
76.79
39.57
28.88
Br
O
S
CH3
O
17
210
200
190
180
170
160
150
140
130
120
110
100
90
80
70
60
50
40
30
20
10
f1 (ppm)

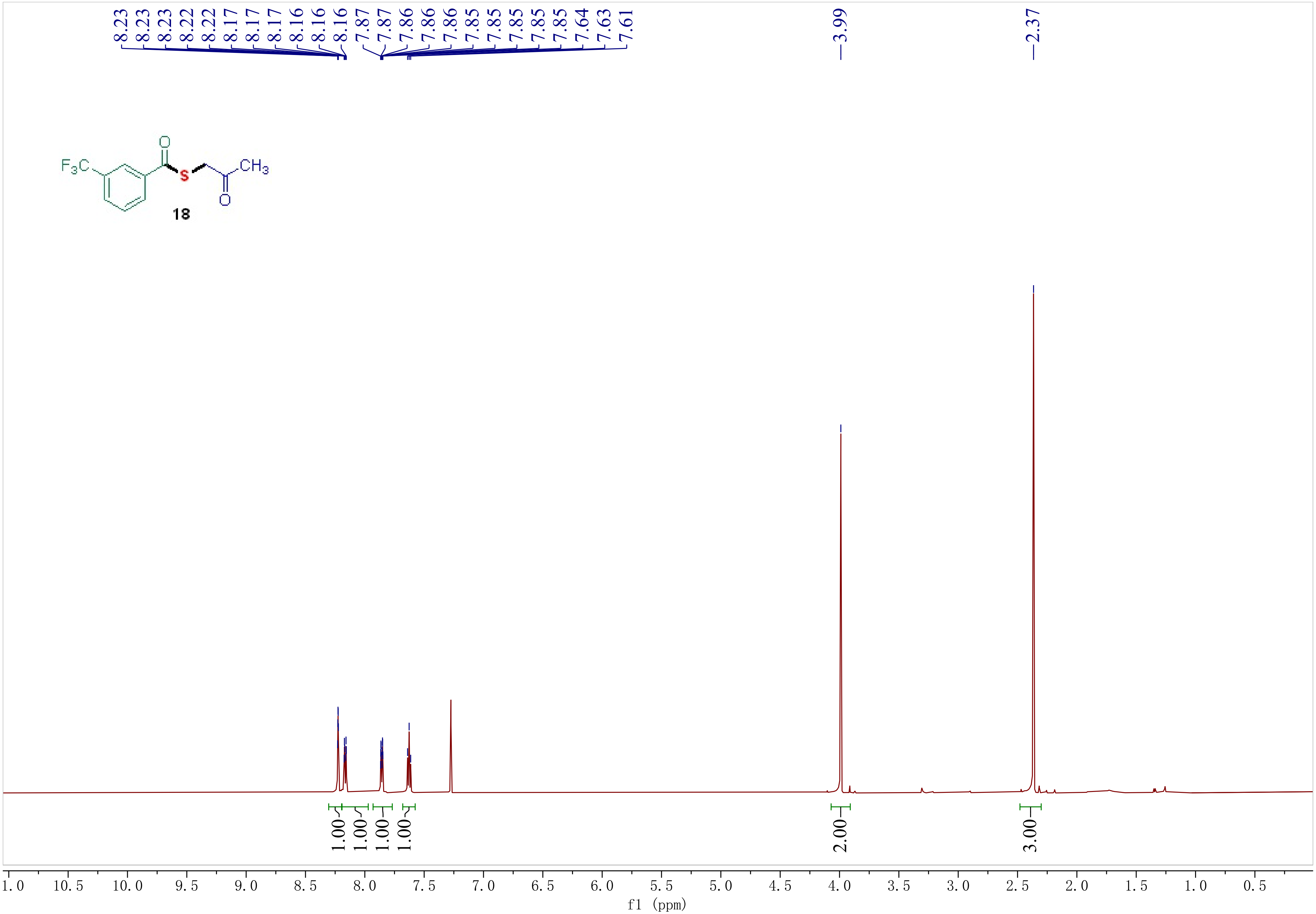
8.23 8.23 8.23 8.22 8.22 8.17 8.17 8.17 8.16 8.16 8.16 7.87 7.87 7.86 7.86 7.86 7.85 7.85 7.85 7.85 7.85 7.64 7.63 7.61
3.99
2.37
F3C
O
S
CH3
O
18
1.00 1.00 1.00 1.00
2.00
3.00
f1 (ppm)

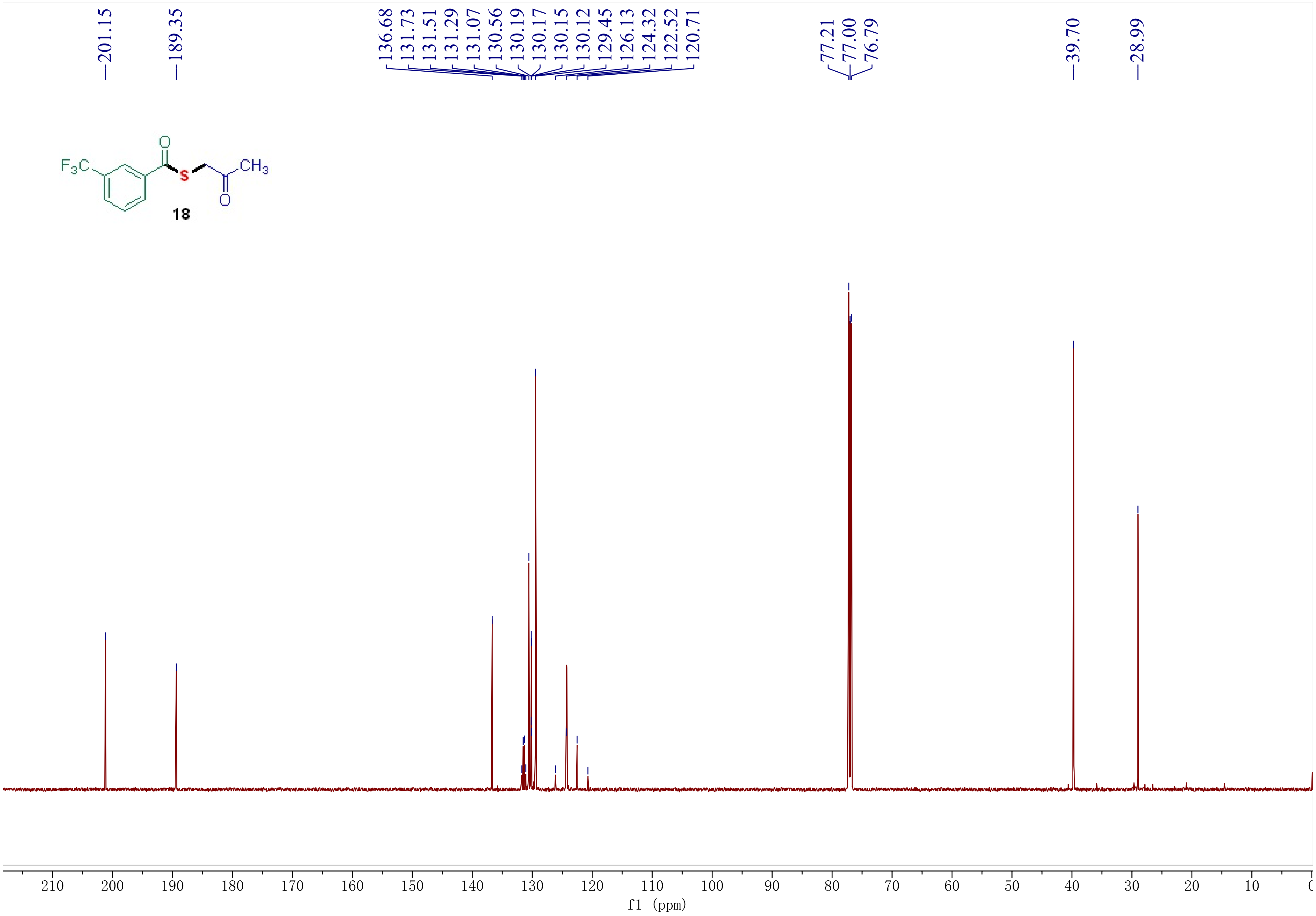

201.15
189.35
136.68
131.73
131.51
131.29
131.07
130.56
130.19
130.17
130.15
130.12
129.45
126.13
124.32
122.52
120.71
77.21
77.00
76.79
39.70
28.99
F3C
O
S
CH3
O
18
210
200
190
180
170
160
150
140
130
120
110
100
90
80
70
60
50
40
30
20
10
f1 (ppm)

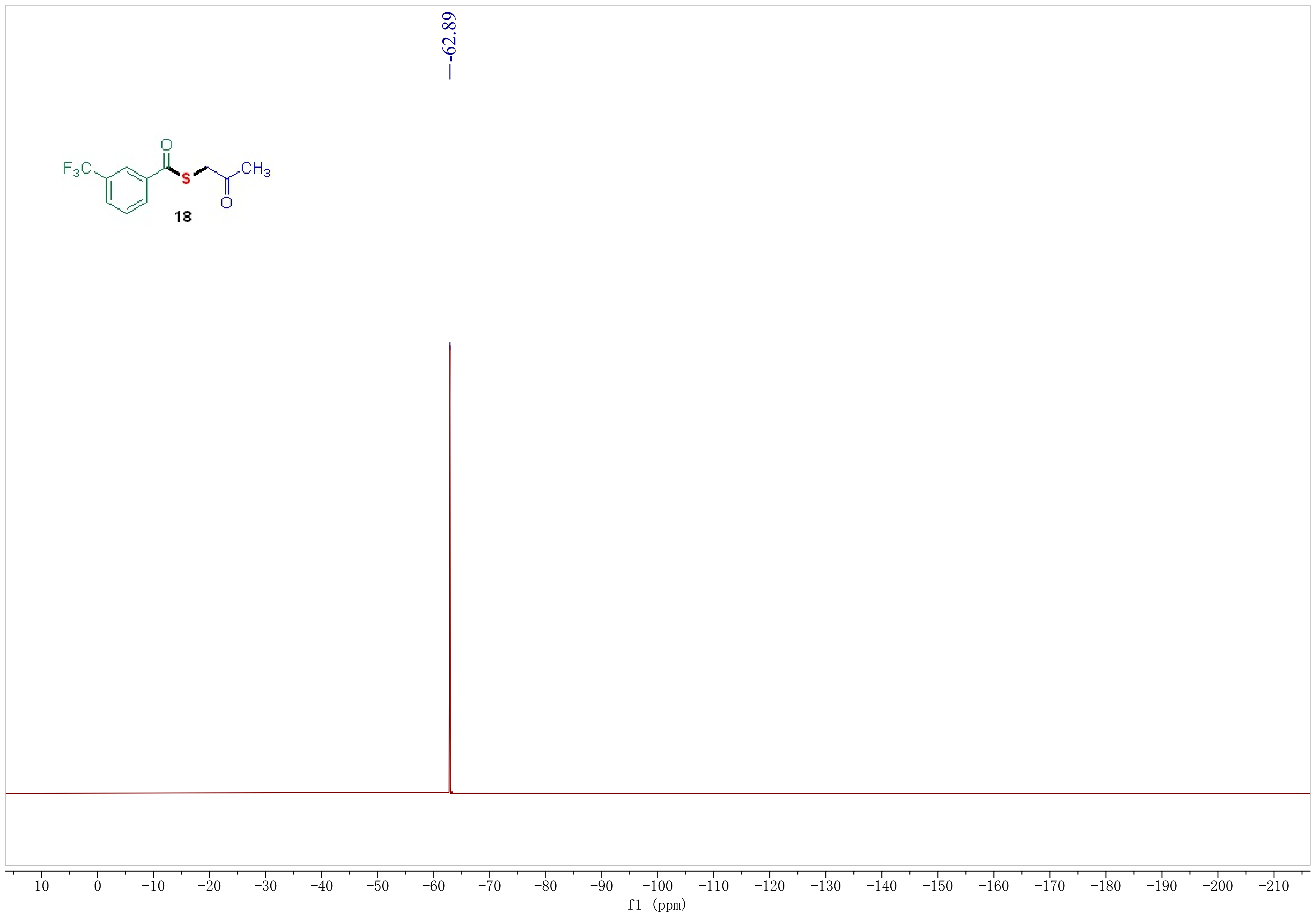

-62.89
F3C
O
S
CH3
O
18
10
0
−10
−20
−30
−40
−50
−60
−70
−80
−90
−100
−110
−120
−130
−140
−150
−160
−170
−180
−190
−200
−210
f1 (ppm)

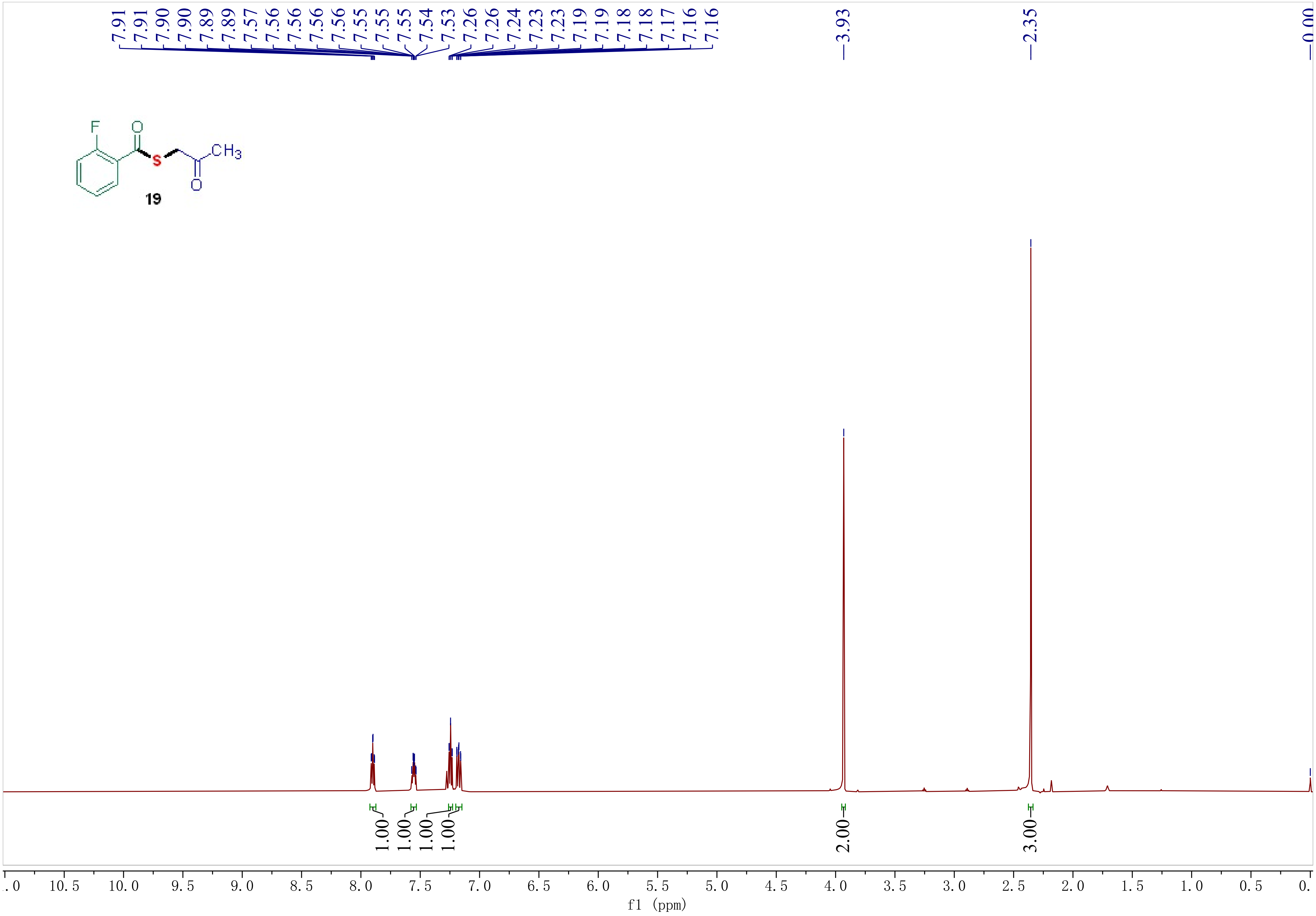
7.91 7.91 7.90 7.90 7.89 7.89 7.57 7.56 7.56 7.56 7.56 7.55 7.55 7.55 7.54 7.53 7.26 7.26 7.24 7.23 7.23 7.19 7.19 7.18 7.18 7.17 7.16 7.16
3.93
2.35
0.00
F
O
S
CH3
O
19
1.00
1.00
1.00
1.00
2.00
3.00
11.0 10.5 10.0 9.5 9.0 8.5 8.0 7.5 7.0 6.5 6.0 5.5 5.0 4.5 4.0 3.5 3.0 2.5 2.0 1.5 1.0 0.5 0.
f1 (ppm)

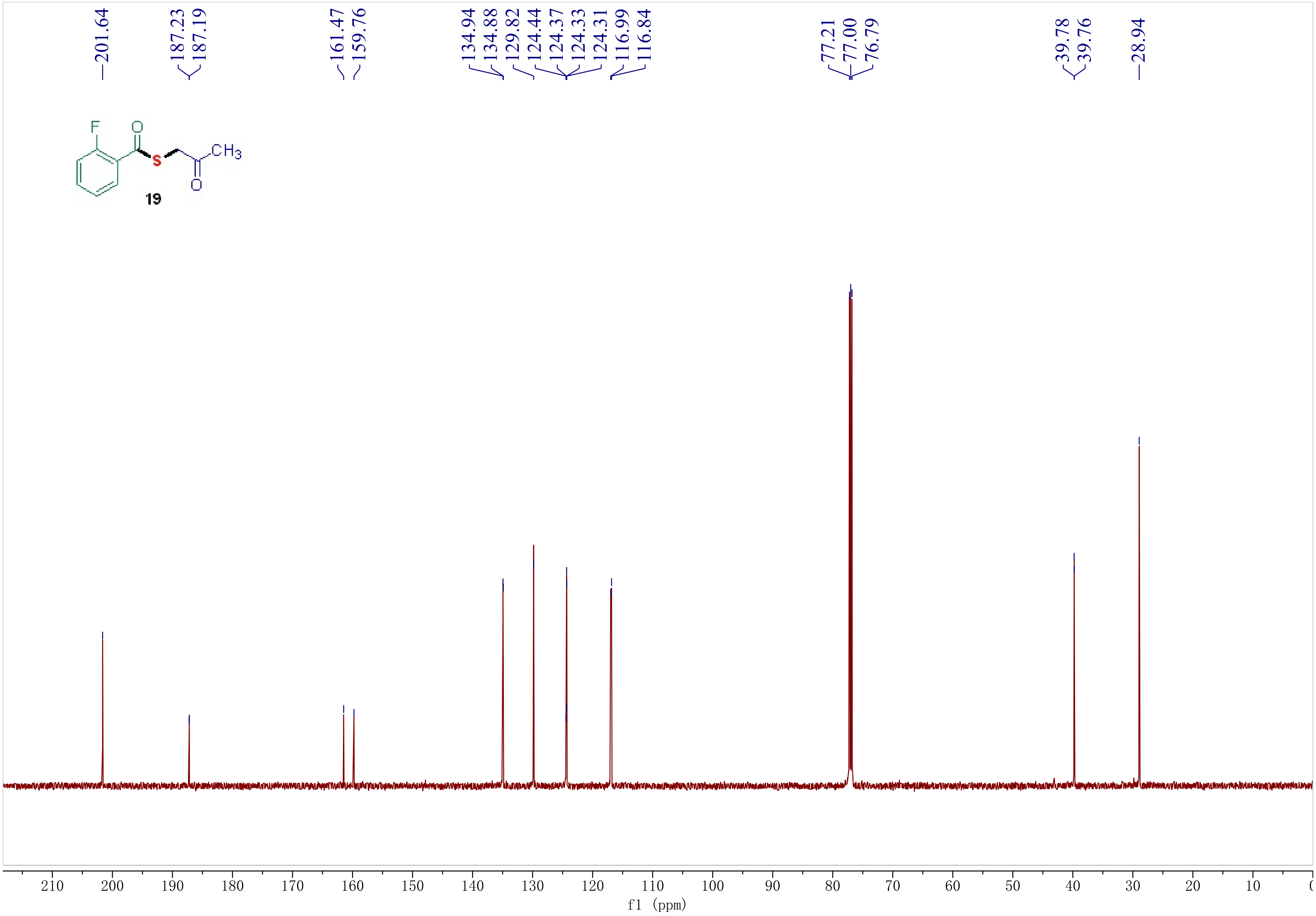

201.64
187.23
187.19
161.47
159.76
134.94
134.88
129.82
124.44
124.37
124.33
124.31
116.99
116.84
77.21
77.00
76.79
39.78
39.76
28.94
F
O
S
CH3
O
19
210 200 190 180 170 160 150 140 130 120 110 100 90 80 70 60 50 40 30 20 10
f1 (ppm)

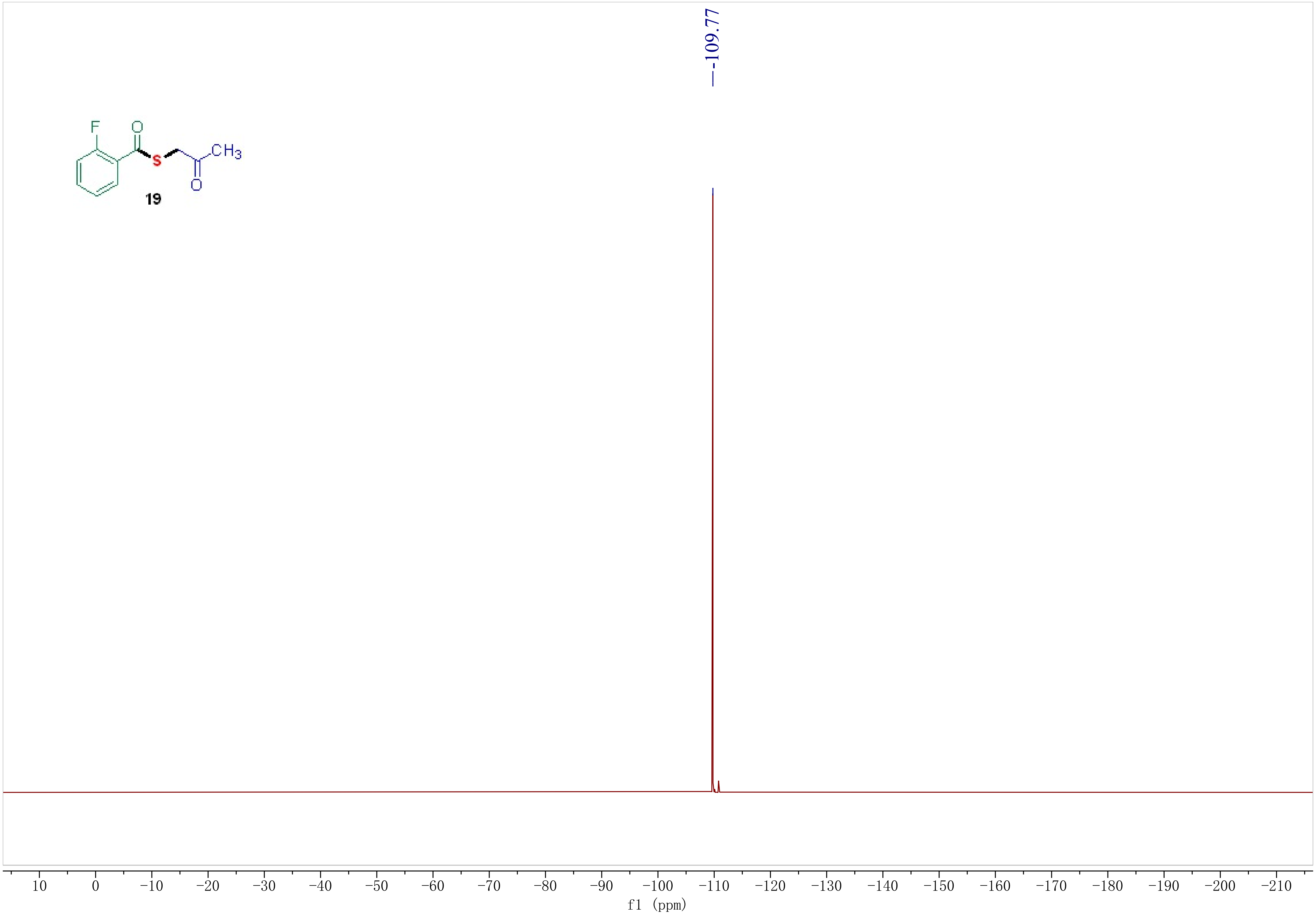

--109.77
F
O
S
CH3
O
19
10 0 −10 −20 −30 −40 −50 −60 −70 −80 −90 −100 −110 −120 −130 −140 −150 −160 −170 −180 −190 −200 −210
f1 (ppm)

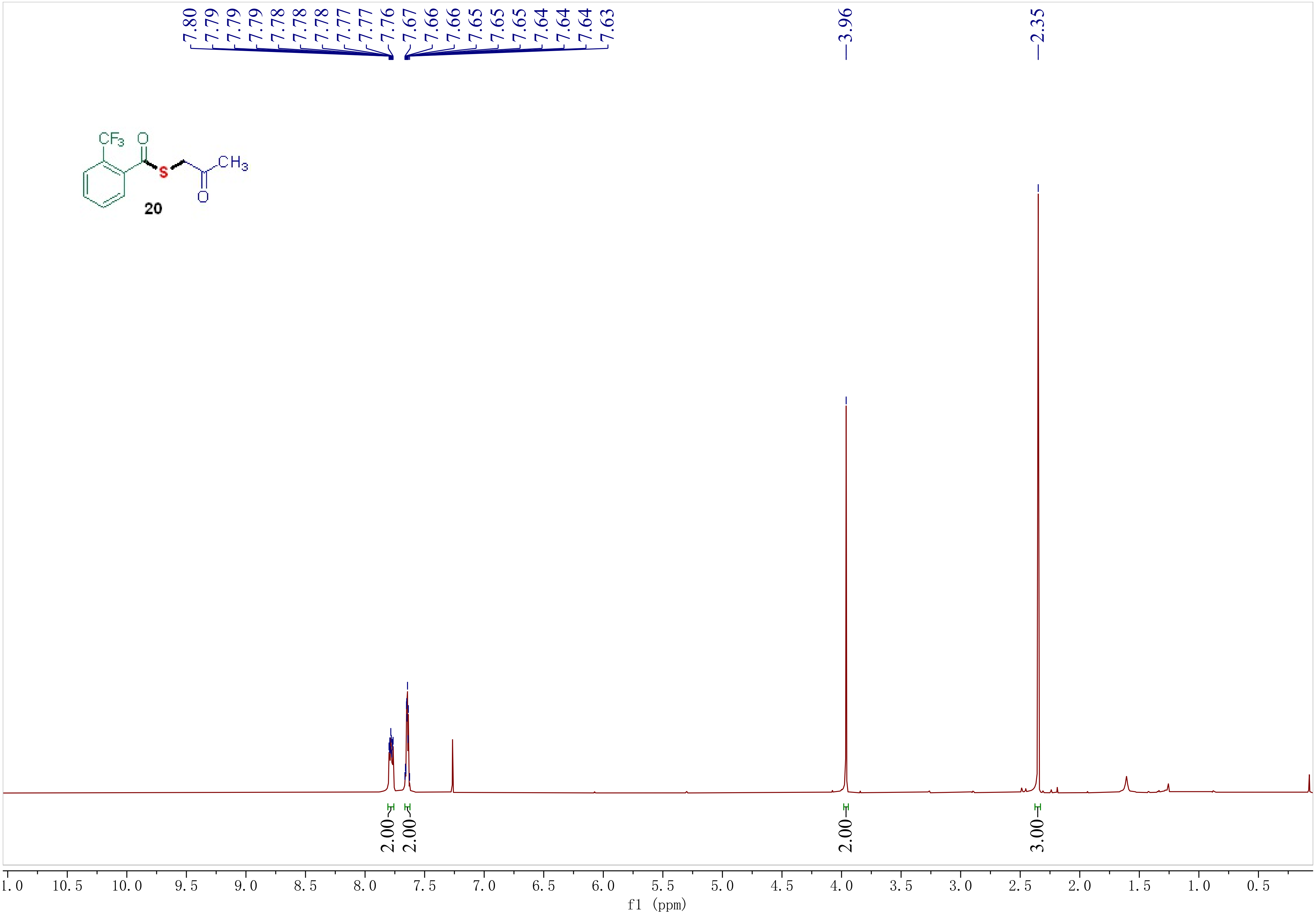
7.80
7.79
7.79
7.79
7.78
7.78
7.78
7.77
7.77
7.76
7.67
7.66
7.66
7.65
7.65
7.65
7.64
7.64
7.64
7.63
3.96
2.35
CF3
O
S
CH3
O
20
2.00
2.00
2.00
3.00
1.0
10.5
10.0
9.5
9.0
8.5
8.0
7.5
7.0
6.5
6.0
5.5
5.0
4.5
4.0
3.5
3.0
2.5
2.0
1.5
1.0
0.5
f1 (ppm)

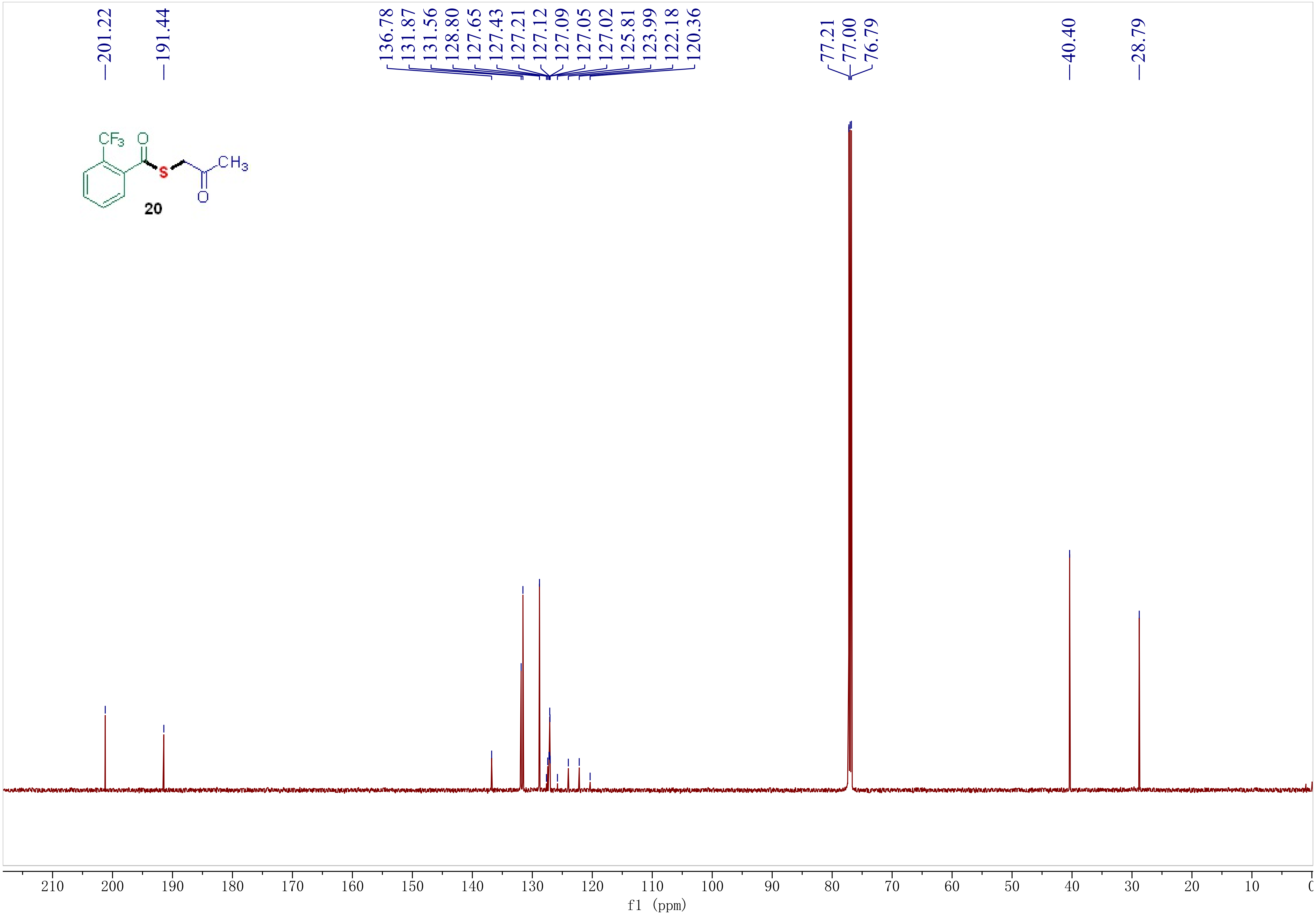

201.22
191.44
136.78
131.87
131.56
128.80
127.65
127.43
127.21
127.12
127.09
127.05
127.02
125.81
123.99
122.18
120.36
77.21
77.00
76.79
40.40
28.79
CF3
O
S
CH3
O
20
210
200
190
180
170
160
150
140
130
120
110
100
90
80
70
60
50
40
30
20
10
f1 (ppm)

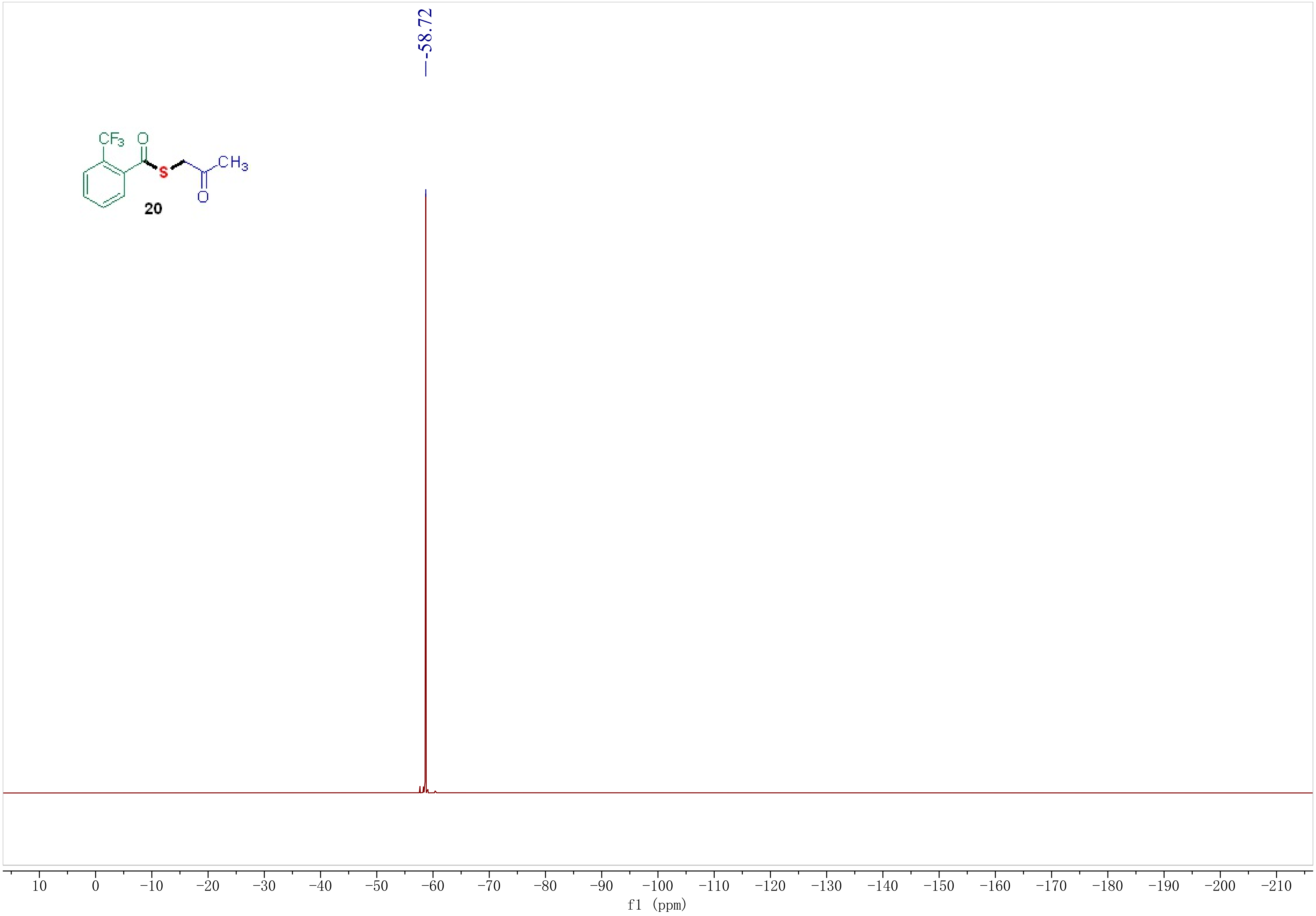
-58.72
CF3
O
S
CH3
O
20
10
0
−10
−20
−30
−40
−50
−60
−70
−80
−90
−100
−110
−120
−130
−140
−150
−160
−170
−180
−190
−200
−210
f1 (ppm)

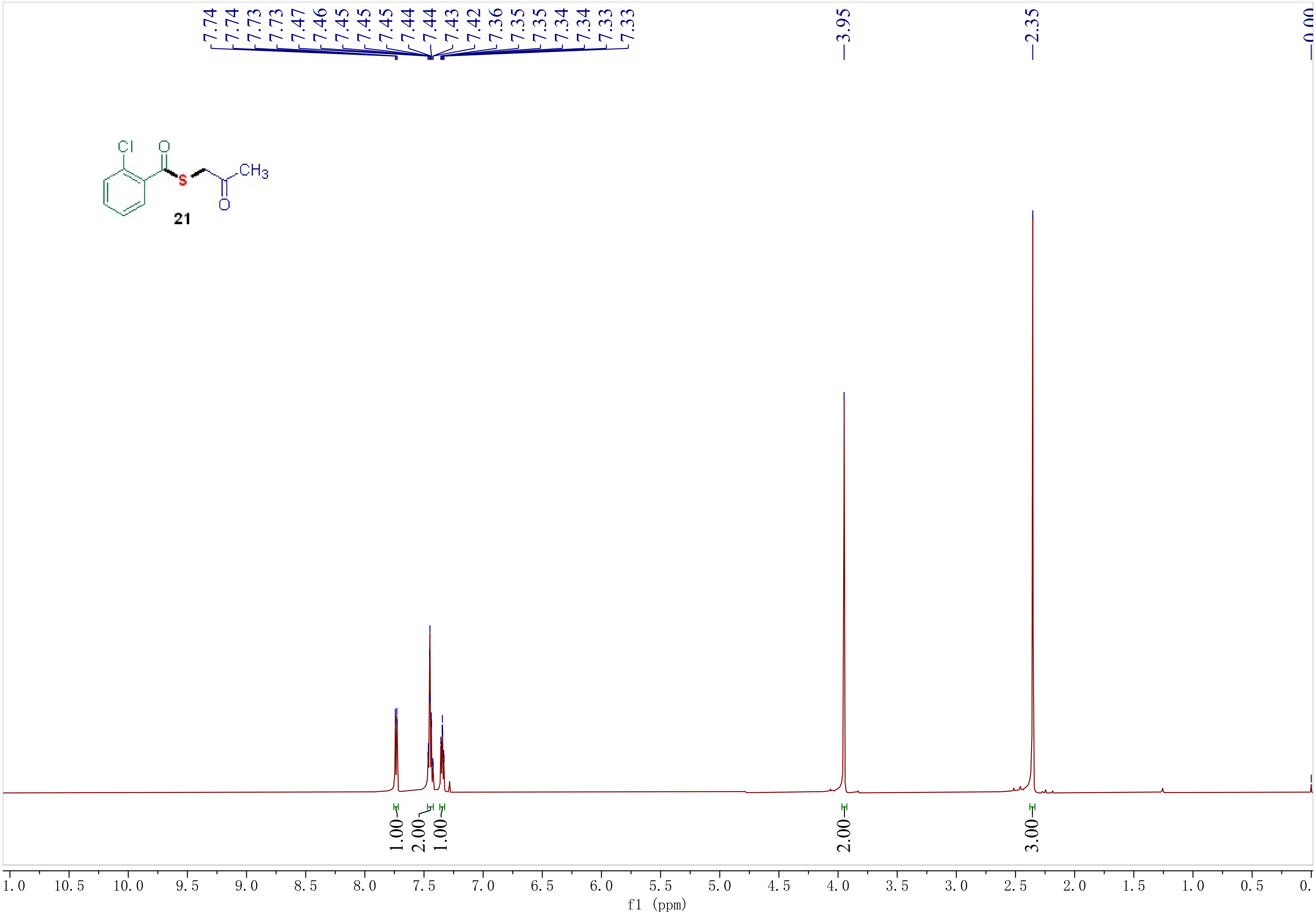
7.74
7.74
7.73
7.73
7.47
7.46
7.45
7.45
7.45
7.44
7.44
7.43
7.42
7.36
7.35
7.35
7.34
7.34
7.33
7.33
3.95
2.35
0.00
Cl
O
S
O
CH3
21
1.00
2.00
1.00
2.00
3.00
f1 (ppm)

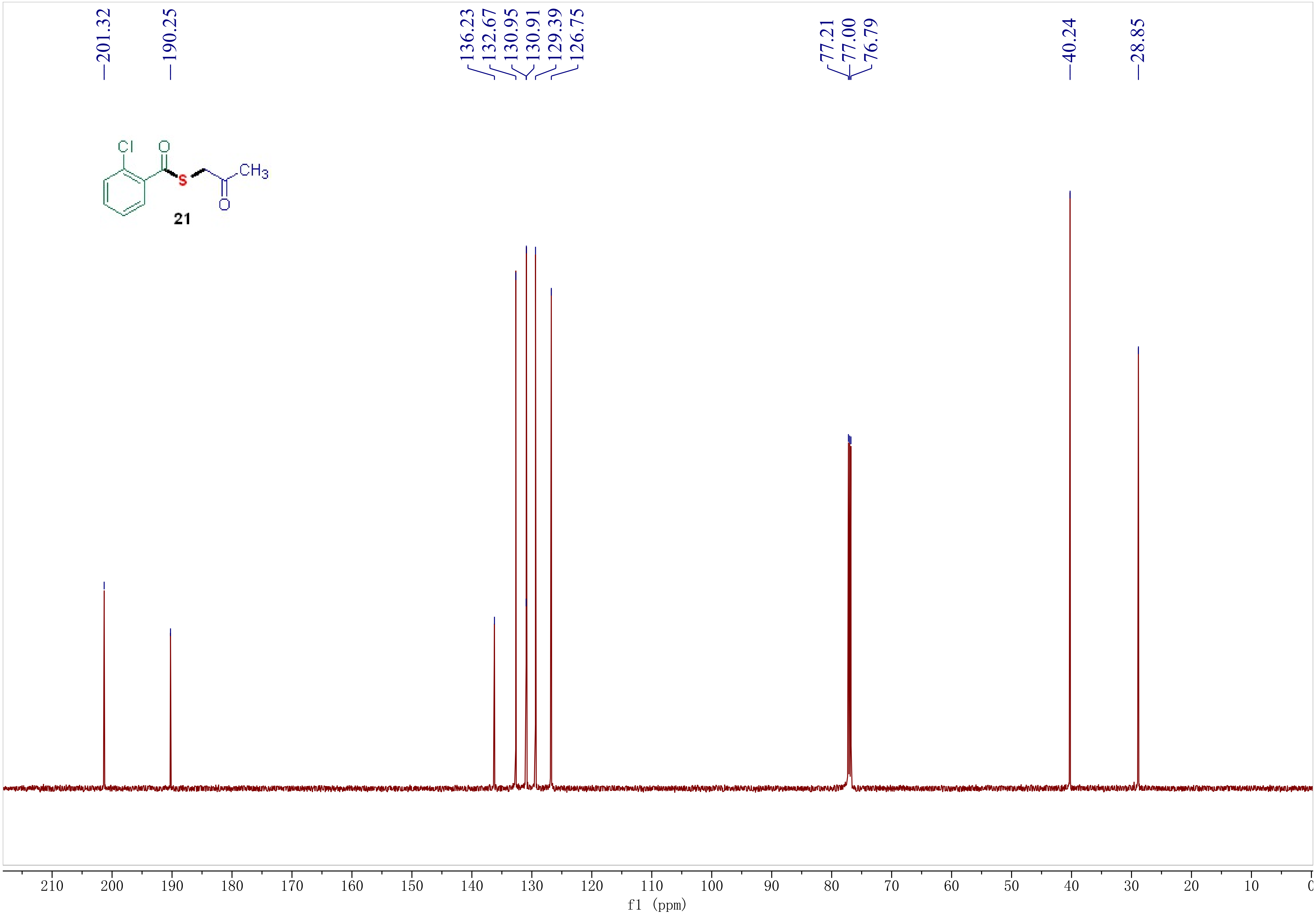

201.32
190.25
136.23
132.67
130.95
130.91
129.39
126.75
77.21
77.00
76.79
40.24
28.85
Cl
O
S
CH3
O
21
210
200
190
180
170
160
150
140
130
120
110
100
90
80
70
60
50
40
30
20
10
0
f1 (ppm)

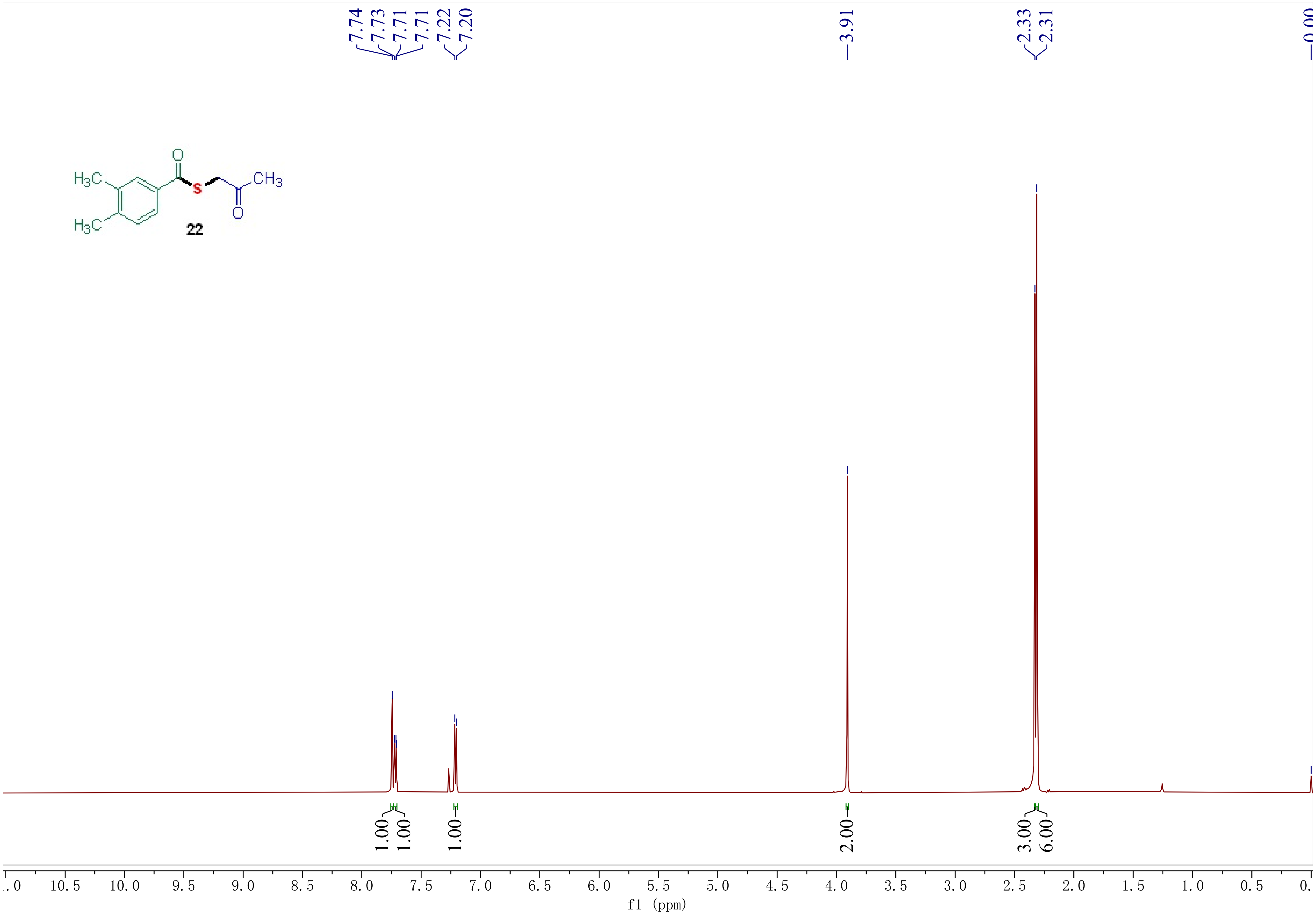

7.74
7.73
7.71
7.71
7.22
7.20
3.91
2.33
2.31
0.00
22
1.00
1.00
1.00
2.00
3.00
6.00
f1 (ppm)

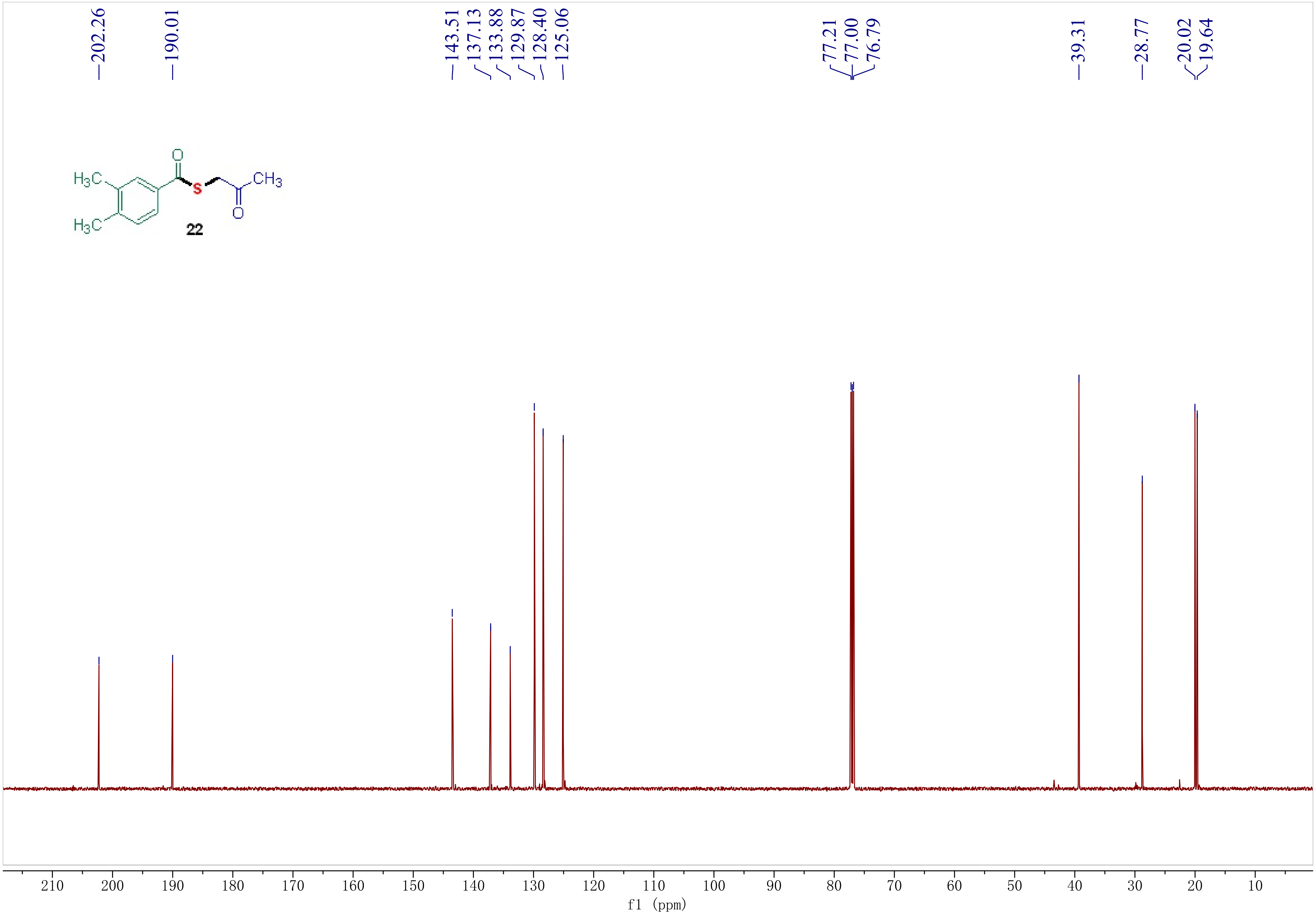
202.26
190.01
143.51
137.13
133.88
129.87
128.40
125.06
77.21
77.00
76.79
39.31
28.77
20.02
19.64
H_3C
H_3C
O
S
CH_3
O
22
210
200
190
180
170
160
150
140
130
120
110
100
90
80
70
60
50
40
30
20
10
f1 (ppm)

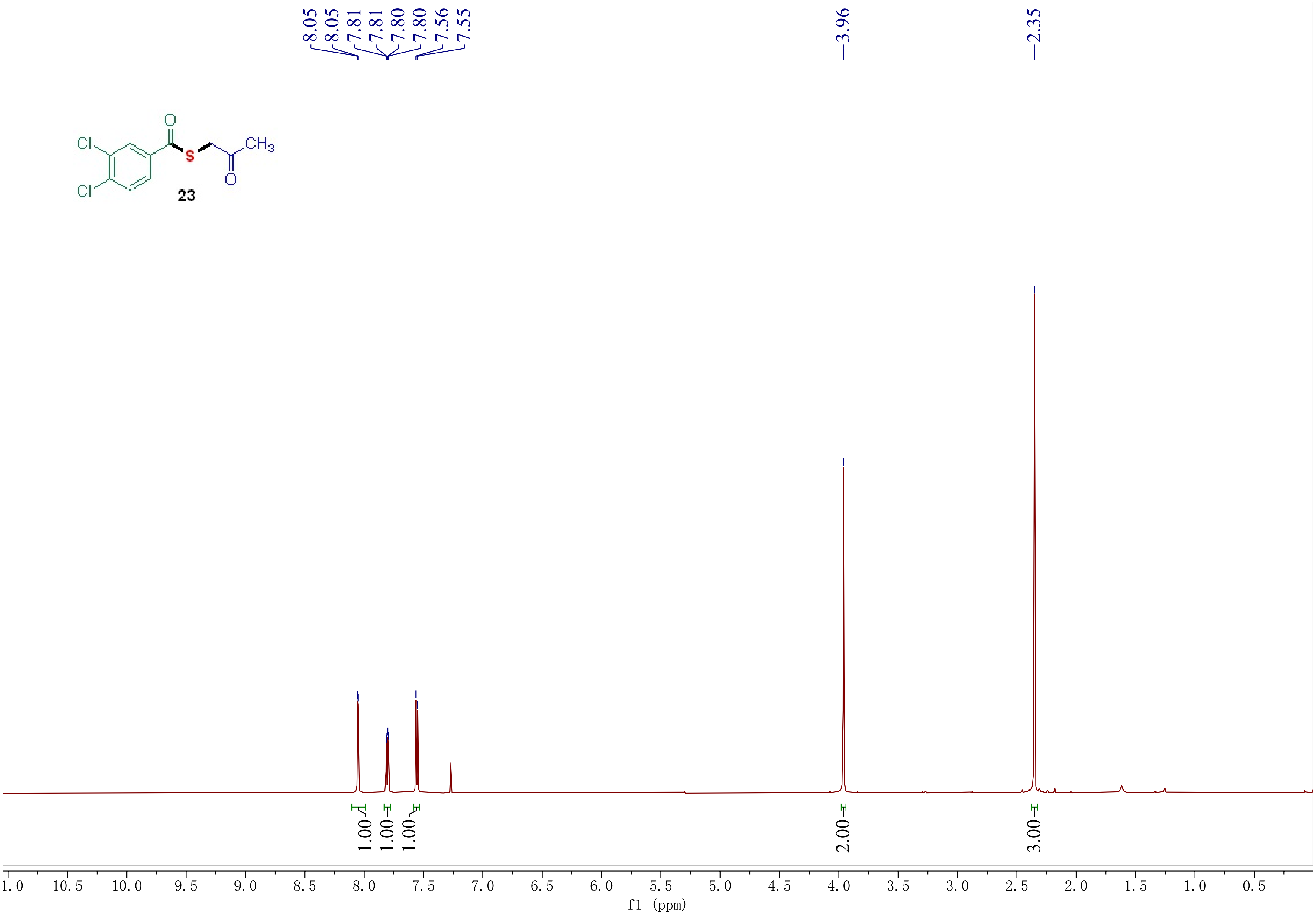

8.05
8.05
7.81
7.81
7.80
7.80
7.56
7.55
3.96
2.35
Cl
Cl
O
S
CH3
O
23
1.00
1.00
1.00
2.00
3.00
1.0
10.5
10.0
9.5
9.0
8.5
8.0
7.5
7.0
6.5
6.0
5.5
5.0
4.5
4.0
3.5
3.0
2.5
2.0
1.5
1.0
0.5
f1 (ppm)

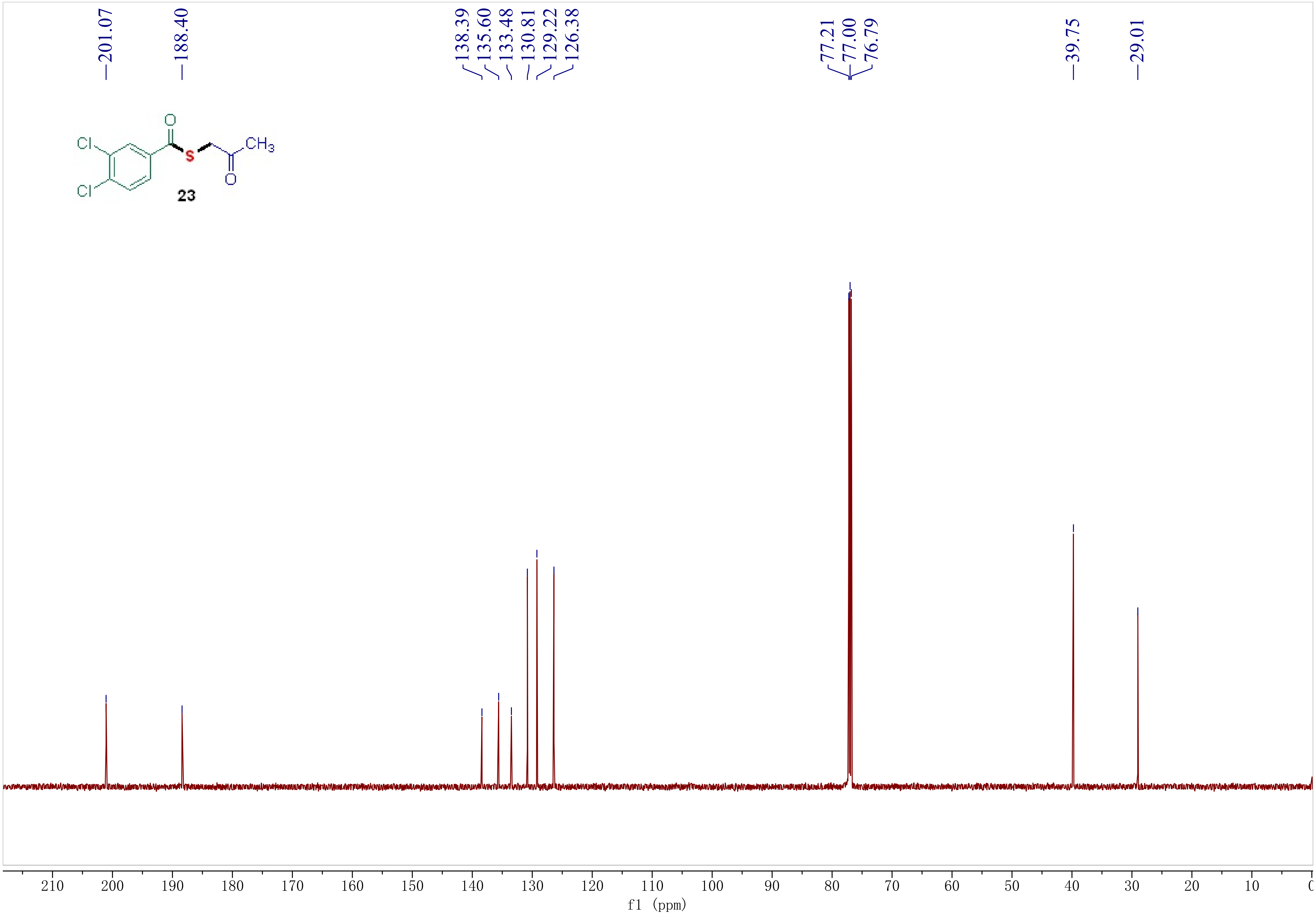

201.07
188.40
138.39
135.60
133.48
130.81
129.22
126.38
77.21
77.00
76.79
39.75
29.01
Cl
Cl
O
S
CH3
O
23
210
200
190
180
170
160
150
140
130
120
110
100
90
80
70
60
50
40
30
20
10
f1 (ppm)

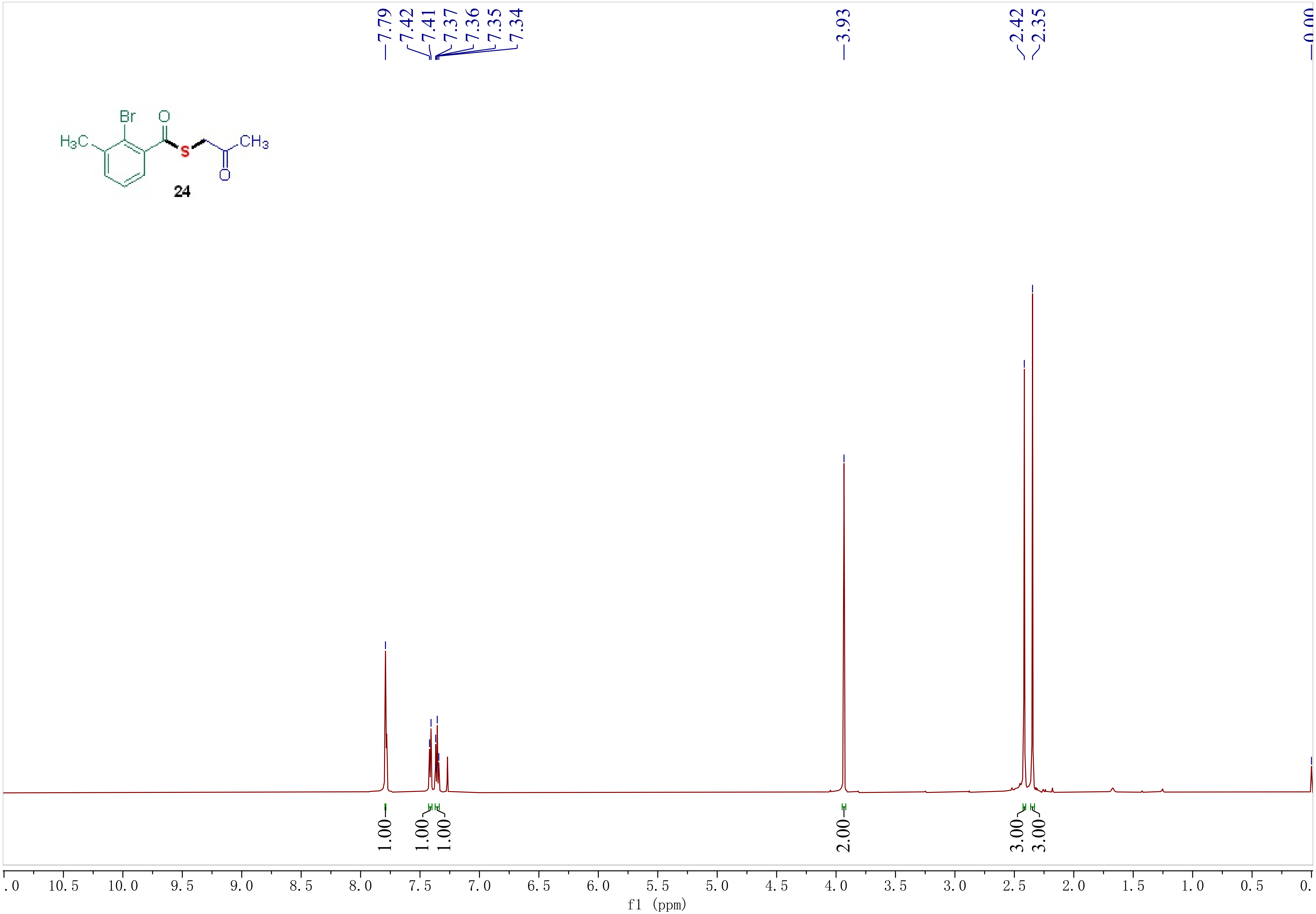
7.79
7.42
7.41
7.37
7.36
7.35
7.34
3.93
2.42
2.35
0.00
Br
O
H_3C
S
CH_3
O
24
1.00
1.00
1.00
2.00
3.00
3.00
.0
10.5
10.0
9.5
9.0
8.5
8.0
7.5
7.0
6.5
6.0
5.5
5.0
4.5
4.0
3.5
3.0
2.5
2.0
1.5
1.0
0.5
0.
f1 (ppm)

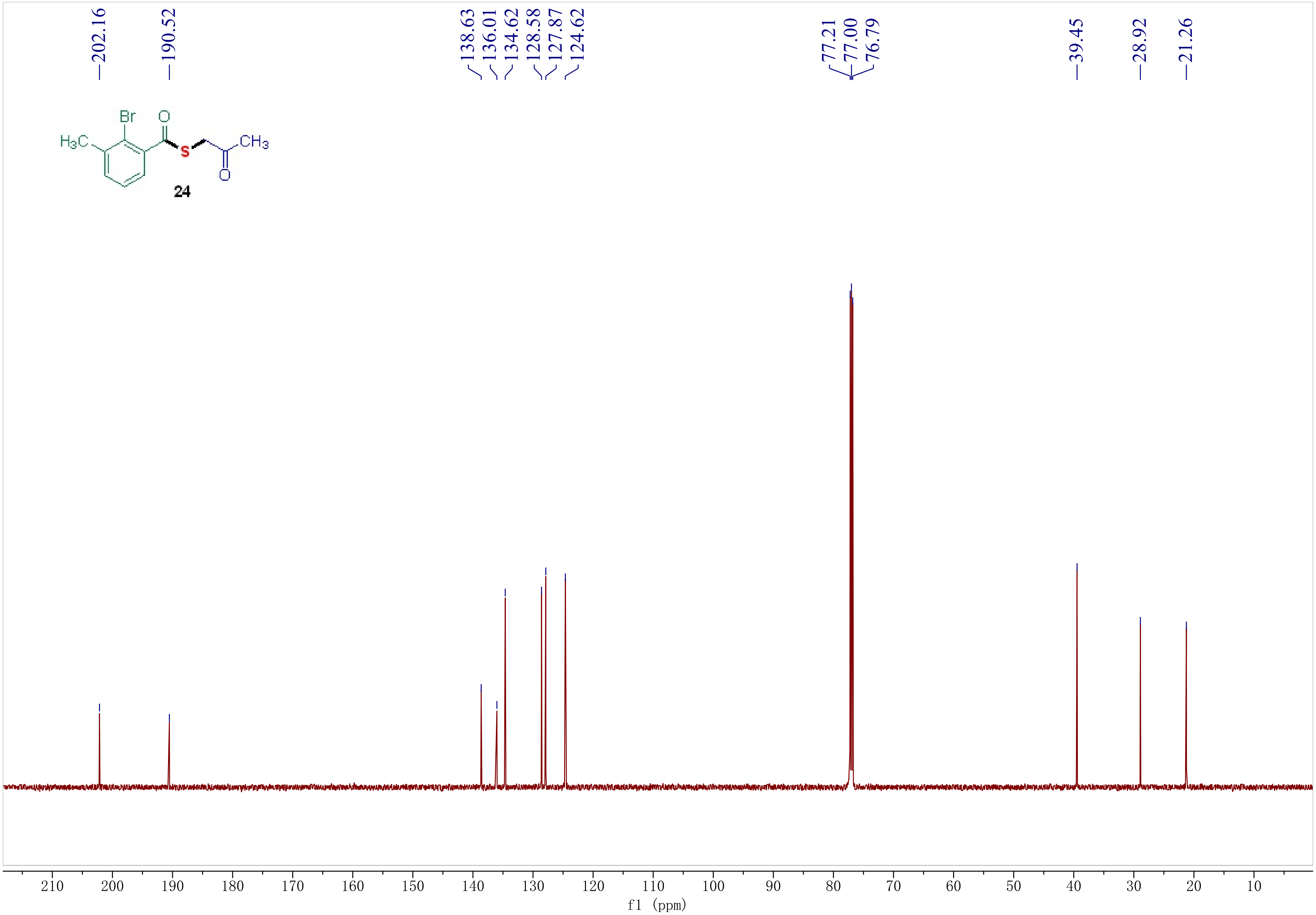
202.16
190.52
138.63
136.01
134.62
128.58
127.87
124.62
77.21
77.00
76.79
39.45
28.92
21.26
Br
O
H3C
S
CH3
O
24
210
200
190
180
170
160
150
140
130
120
110
100
90
80
70
60
50
40
30
20
10
f1 (ppm)

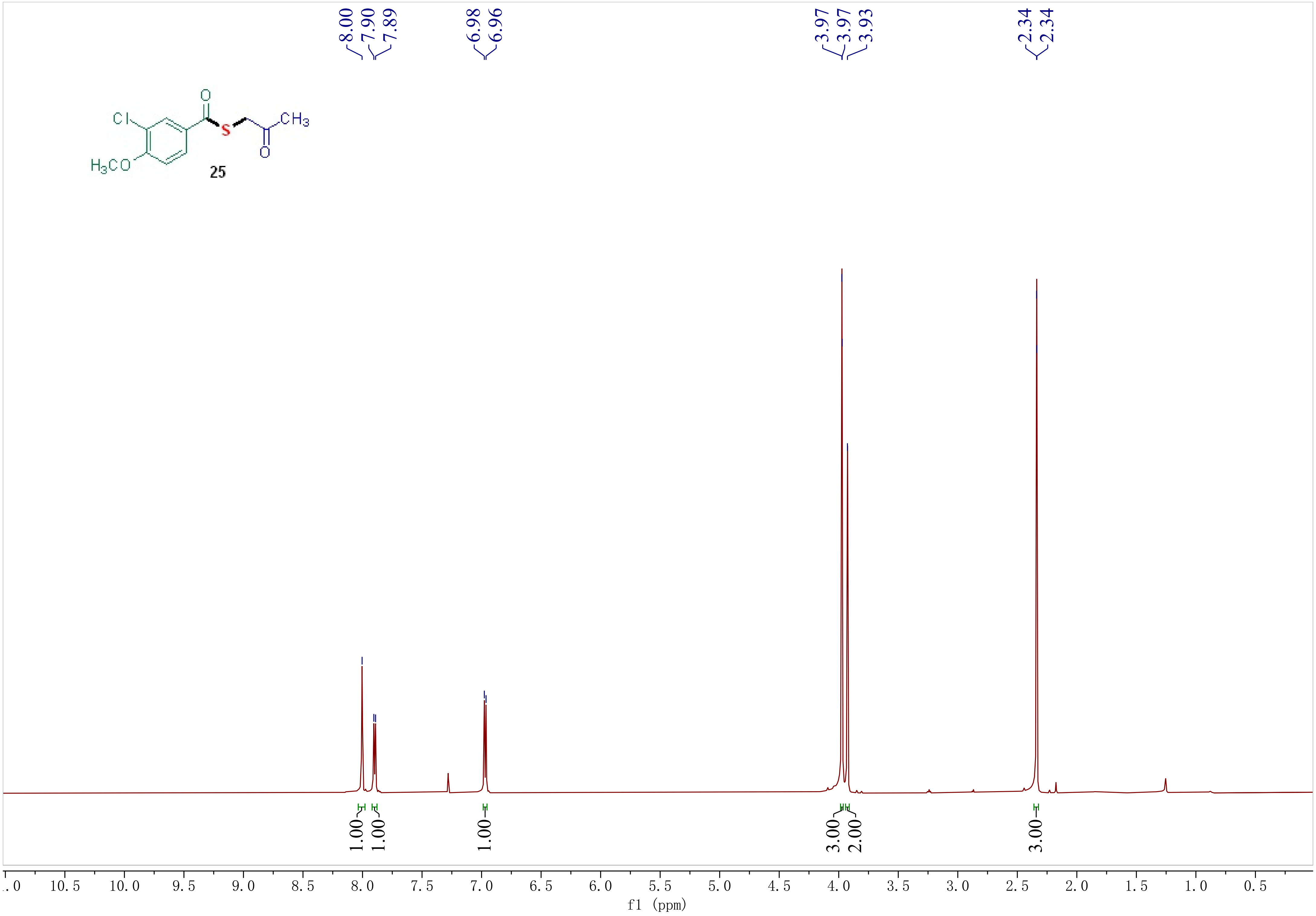

8.00
7.90
7.89
6.98
6.96
3.97
3.97
3.93
2.34
2.34
Cl
O
S
CH3
O
H3CO
25
1.00
1.00
1.00
3.00
2.00
3.00
11.0
10.5
10.0
9.5
9.0
8.5
8.0
7.5
7.0
6.5
6.0
5.5
5.0
4.5
4.0
3.5
3.0
2.5
2.0
1.5
1.0
0.5
f1 (ppm)

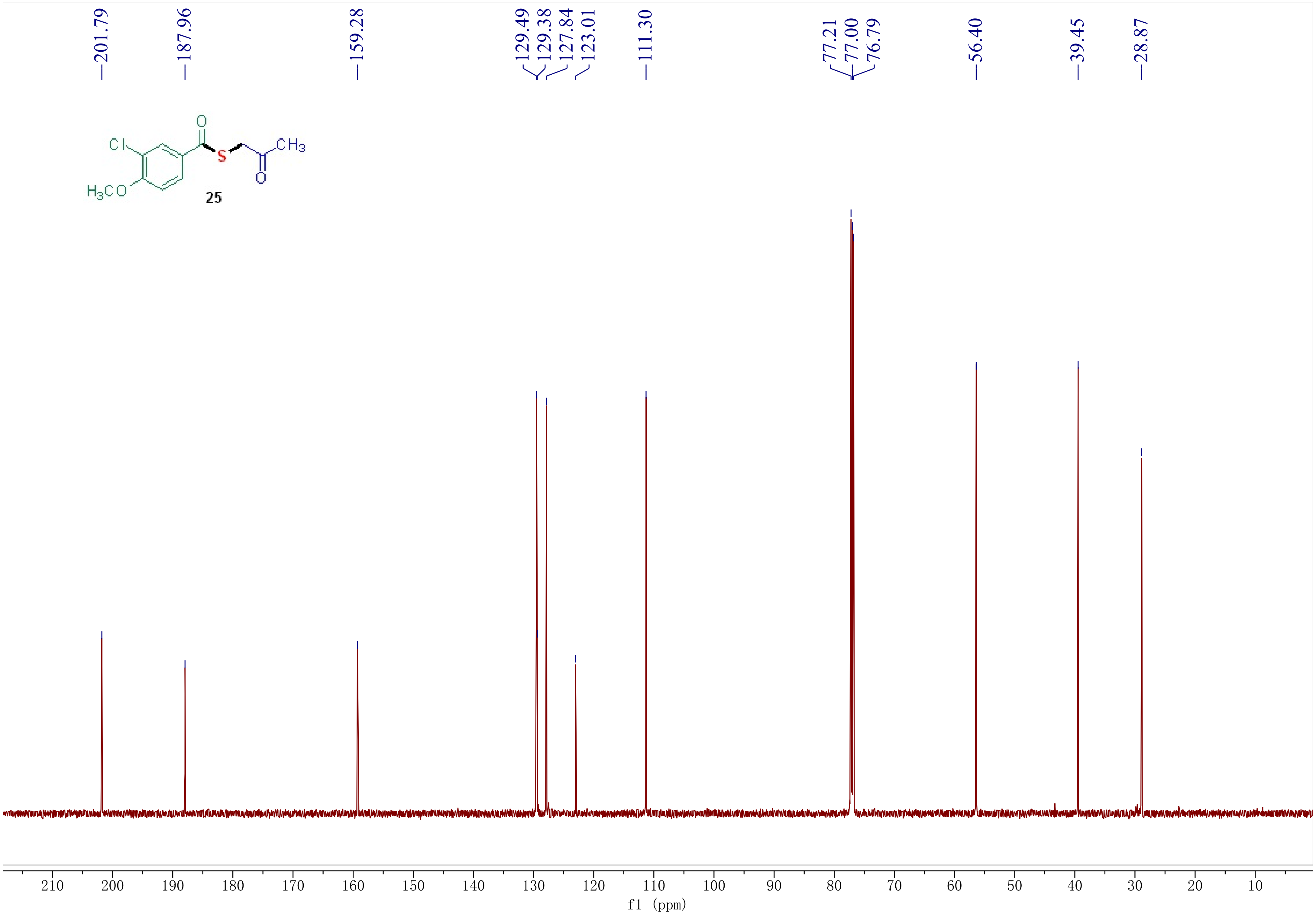

201.79
187.96
159.28
129.49
129.38
127.84
123.01
111.30
77.21
77.00
76.79
56.40
39.45
28.87
Cl
H3CO
O
S
CH3
25
210
200
190
180
170
160
150
140
130
120
110
100
90
80
70
60
50
40
30
20
10
f1 (ppm)

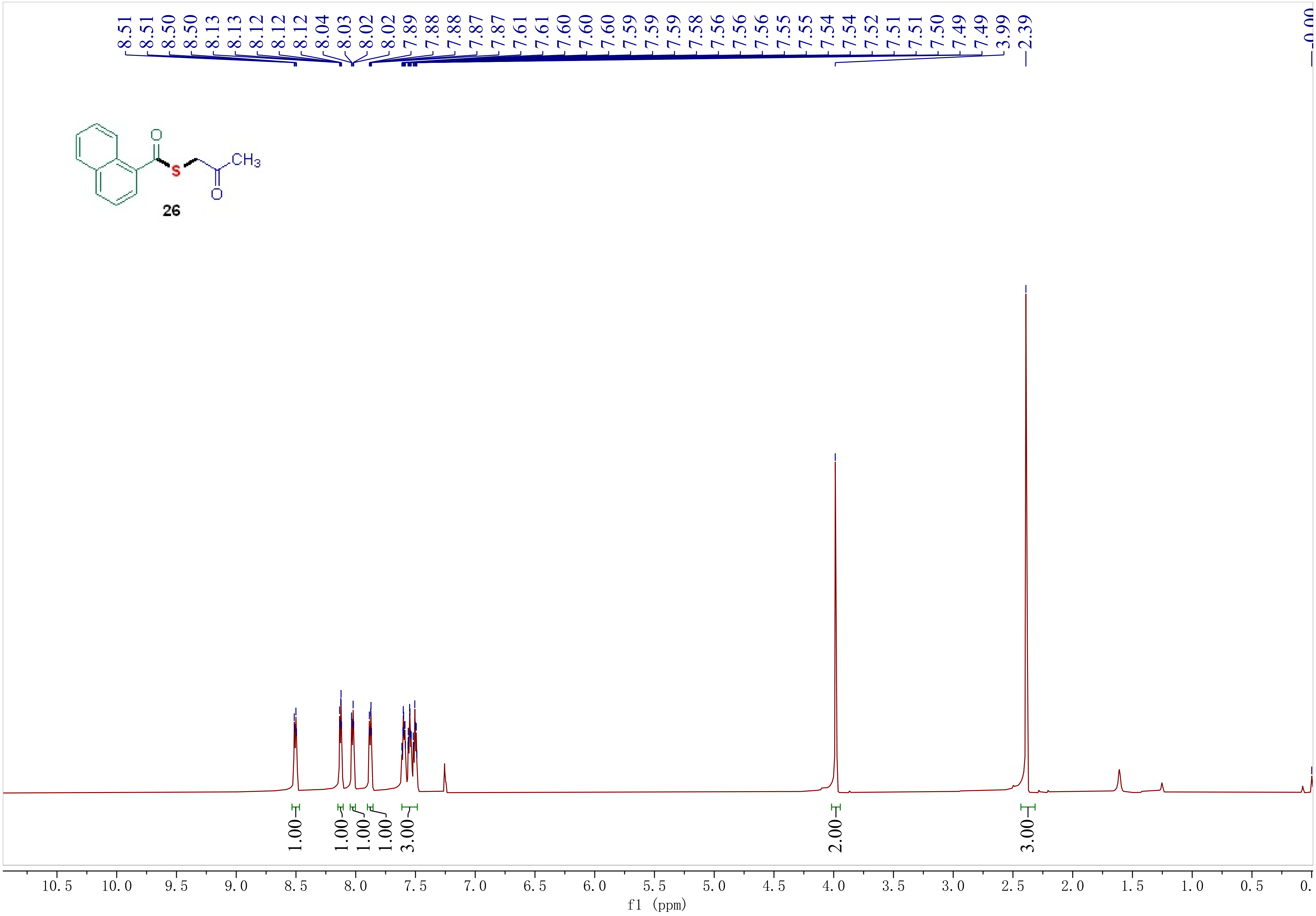
8.51 8.51 8.50 8.50 8.13 8.13 8.12 8.12 8.12 8.04 8.03 8.02 8.02 7.89 7.88 7.88 7.87 7.87 7.61 7.61 7.60 7.60 7.60 7.59 7.59 7.59 7.58 7.56 7.56 7.56 7.55 7.55 7.54 7.54 7.52 7.51 7.51 7.50 7.49 7.49 3.99 2.39 0.00
O
S
CH3
O
26
1.00
1.00
1.00
1.00
3.00
2.00
3.00
10.5 10.0 9.5 9.0 8.5 8.0 7.5 7.0 6.5 6.0 5.5 5.0 4.5 4.0 3.5 3.0 2.5 2.0 1.5 1.0 0.5 0.
f1 (ppm)

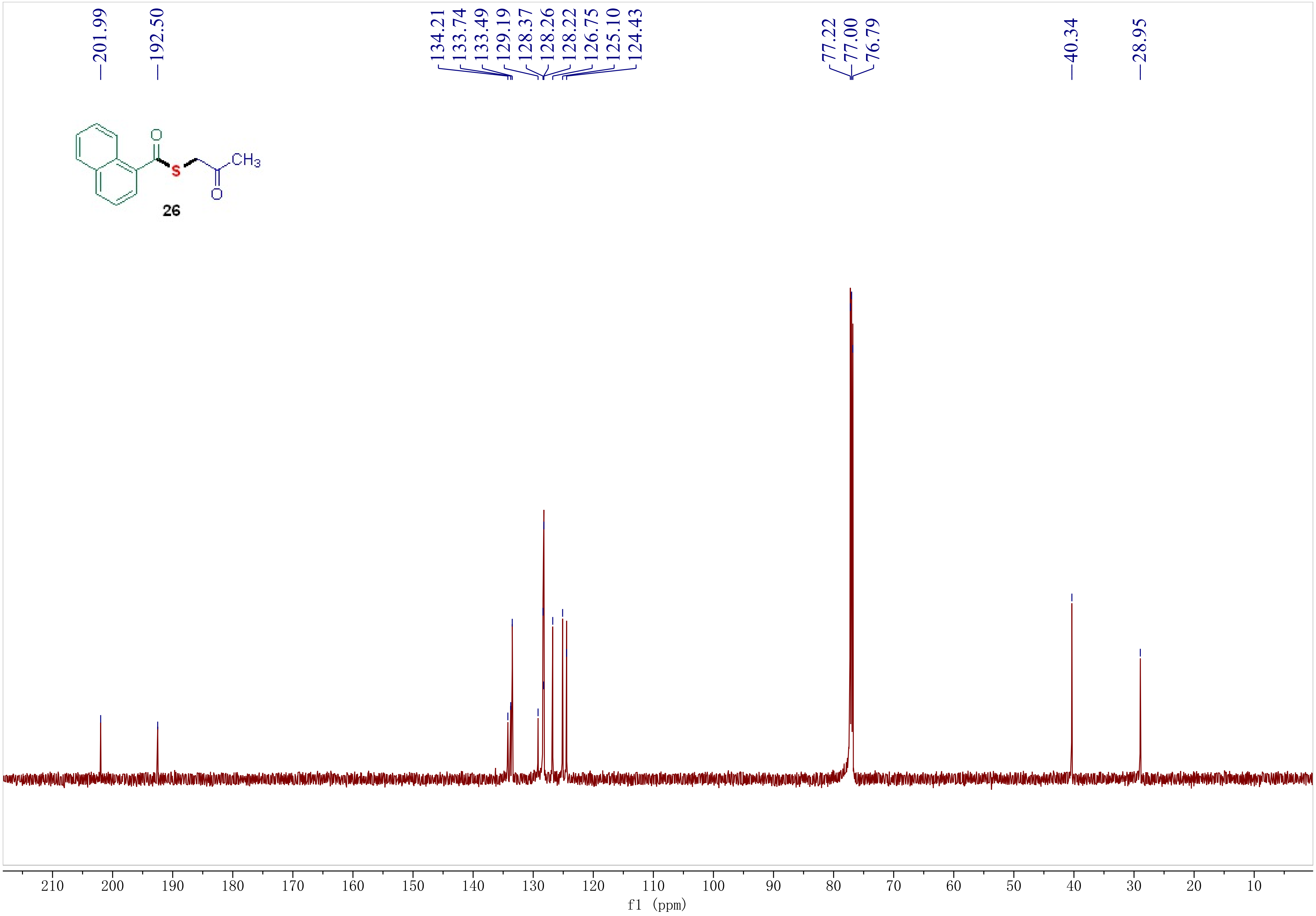

201.99
192.50
134.21
133.74
133.49
129.19
128.37
128.26
128.22
126.75
125.10
124.43
77.22
77.00
76.79
40.34
28.95
O
S
CH3
O
26
210
200
190
180
170
160
150
140
130
120
110
100
90
80
70
60
50
40
30
20
10
f1 (ppm)

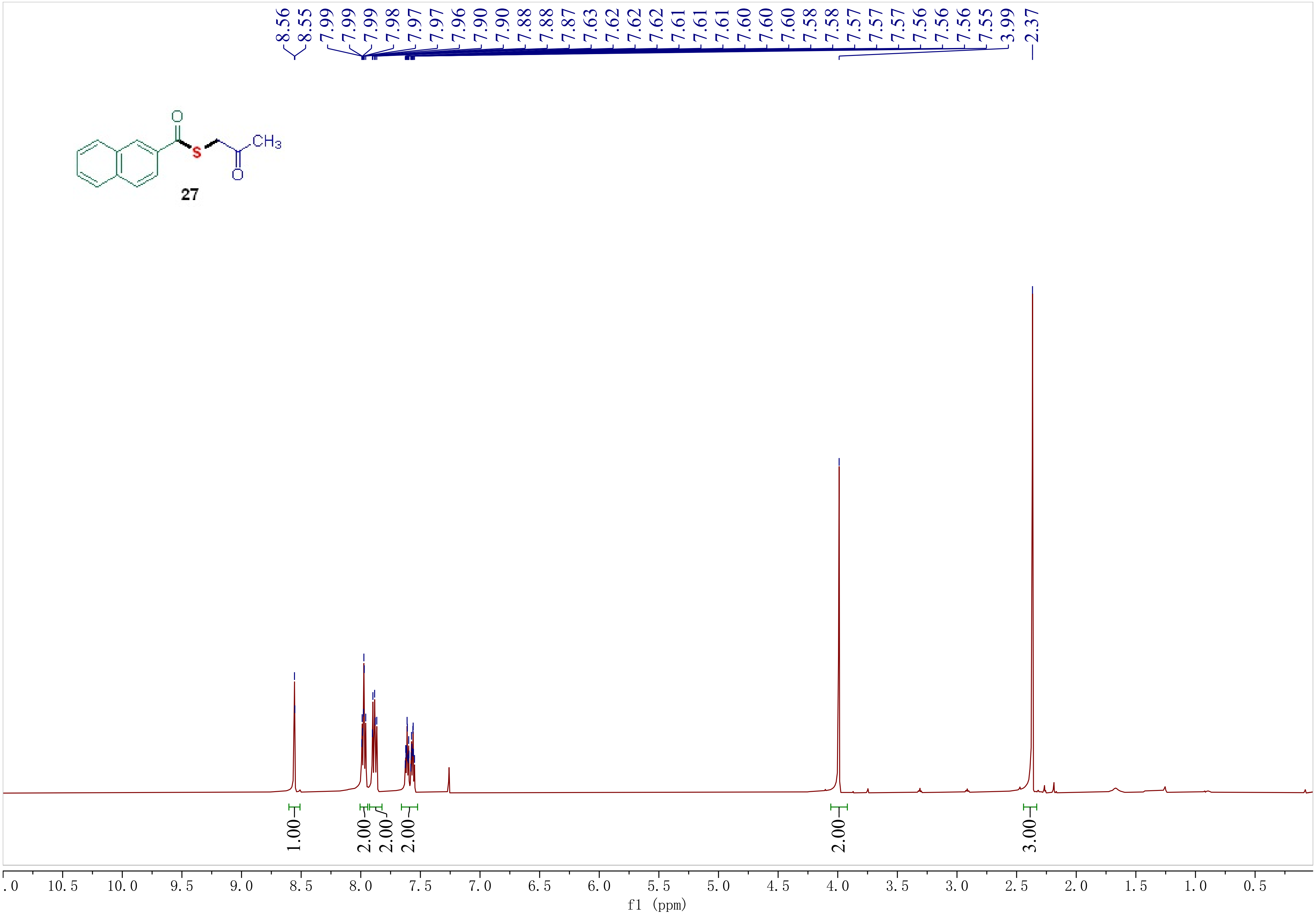

8.56
8.55
7.99
7.99
7.99
7.98
7.97
7.97
7.96
7.90
7.90
7.88
7.88
7.87
7.63
7.62
7.62
7.62
7.61
7.61
7.61
7.60
7.60
7.60
7.58
7.58
7.57
7.57
7.57
7.56
7.56
7.56
7.55
3.99
2.37
O
S
CH3
O
27
1.00
2.00
2.00
2.00
2.00
3.00
.0
10.5
10.0
9.5
9.0
8.5
8.0
7.5
7.0
6.5
6.0
5.5
5.0
4.5
4.0
3.5
3.0
2.5
2.0
1.5
1.0
0.5
f1 (ppm)

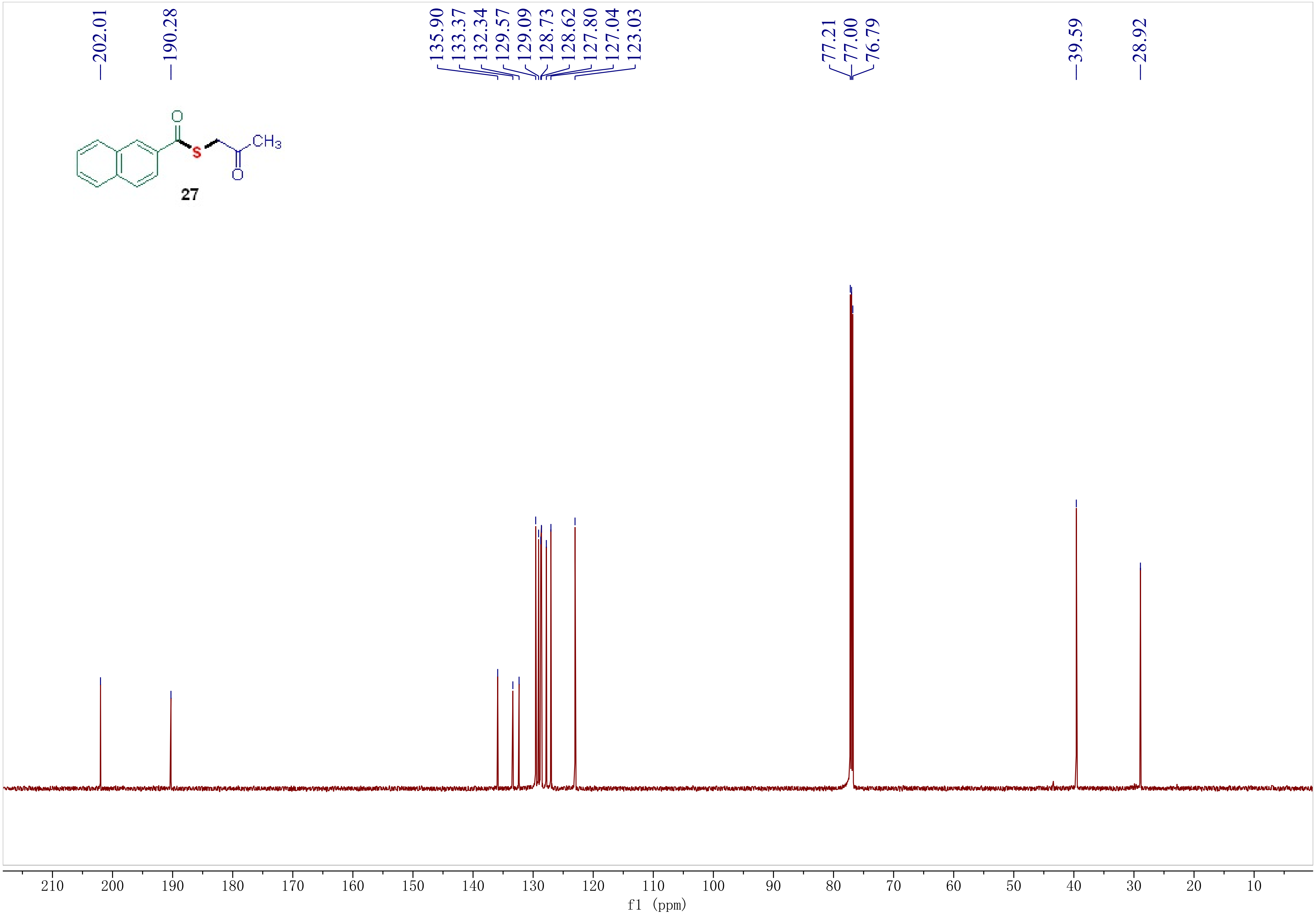
202.01
190.28
135.90
133.37
132.34
129.57
129.09
128.73
128.62
127.80
127.04
123.03
77.21
77.00
76.79
39.59
28.92
O
S
CH3
O
27
210
200
190
180
170
160
150
140
130
120
110
100
90
80
70
60
50
40
30
20
10
f1 (ppm)

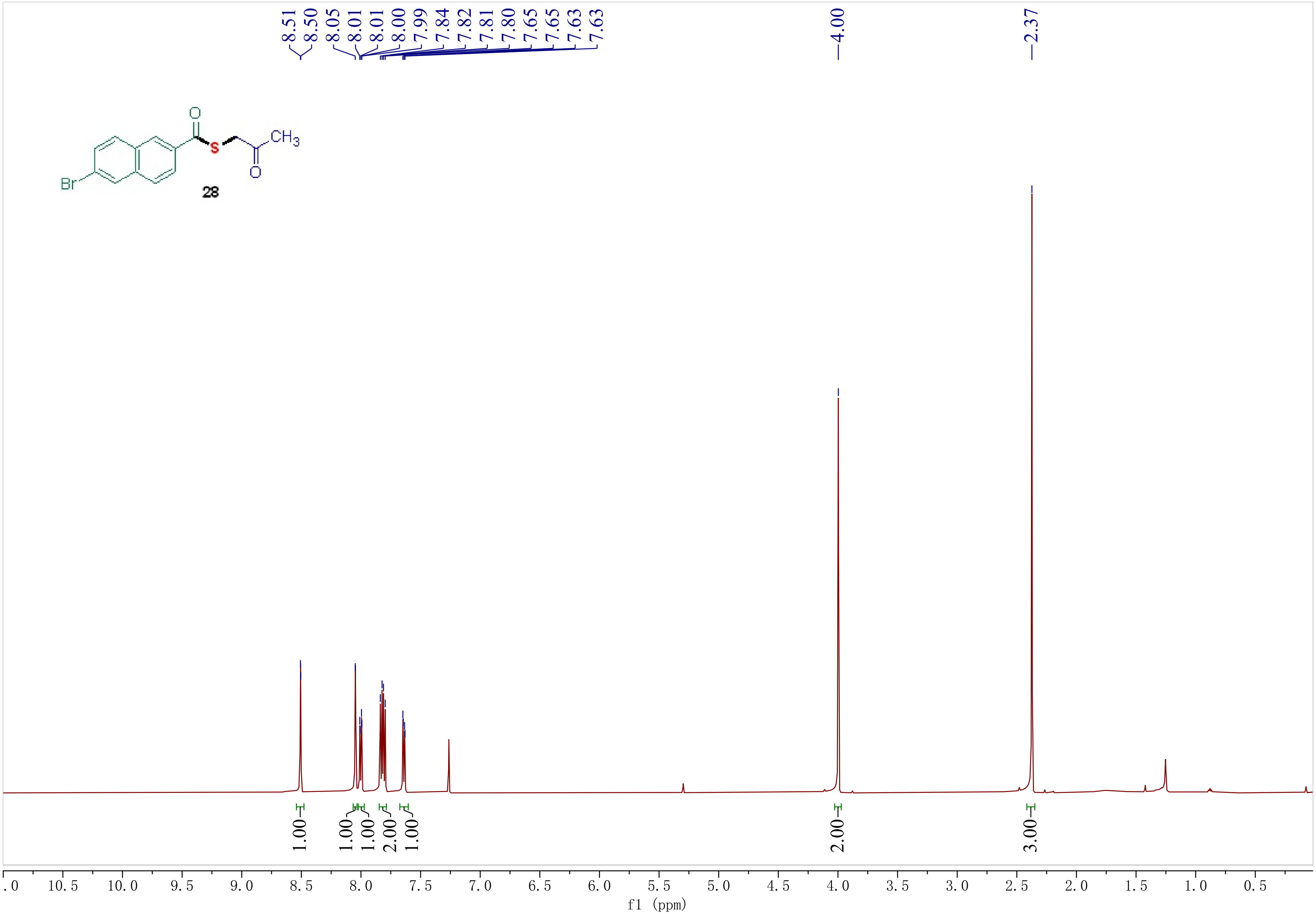

8.51
8.50
8.05
8.01
8.01
8.00
7.99
7.84
7.82
7.81
7.80
7.65
7.65
7.63
7.63
4.00
2.37
O
S
CH3
O
Br
28
1.00
1.00
1.00
2.00
1.00
2.00
3.00
.0
10.5
10.0
9.5
9.0
8.5
8.0
7.5
7.0
6.5
6.0
5.5
5.0
4.5
4.0
3.5
3.0
2.5
2.0
1.5
1.0
0.5
f1 (ppm)

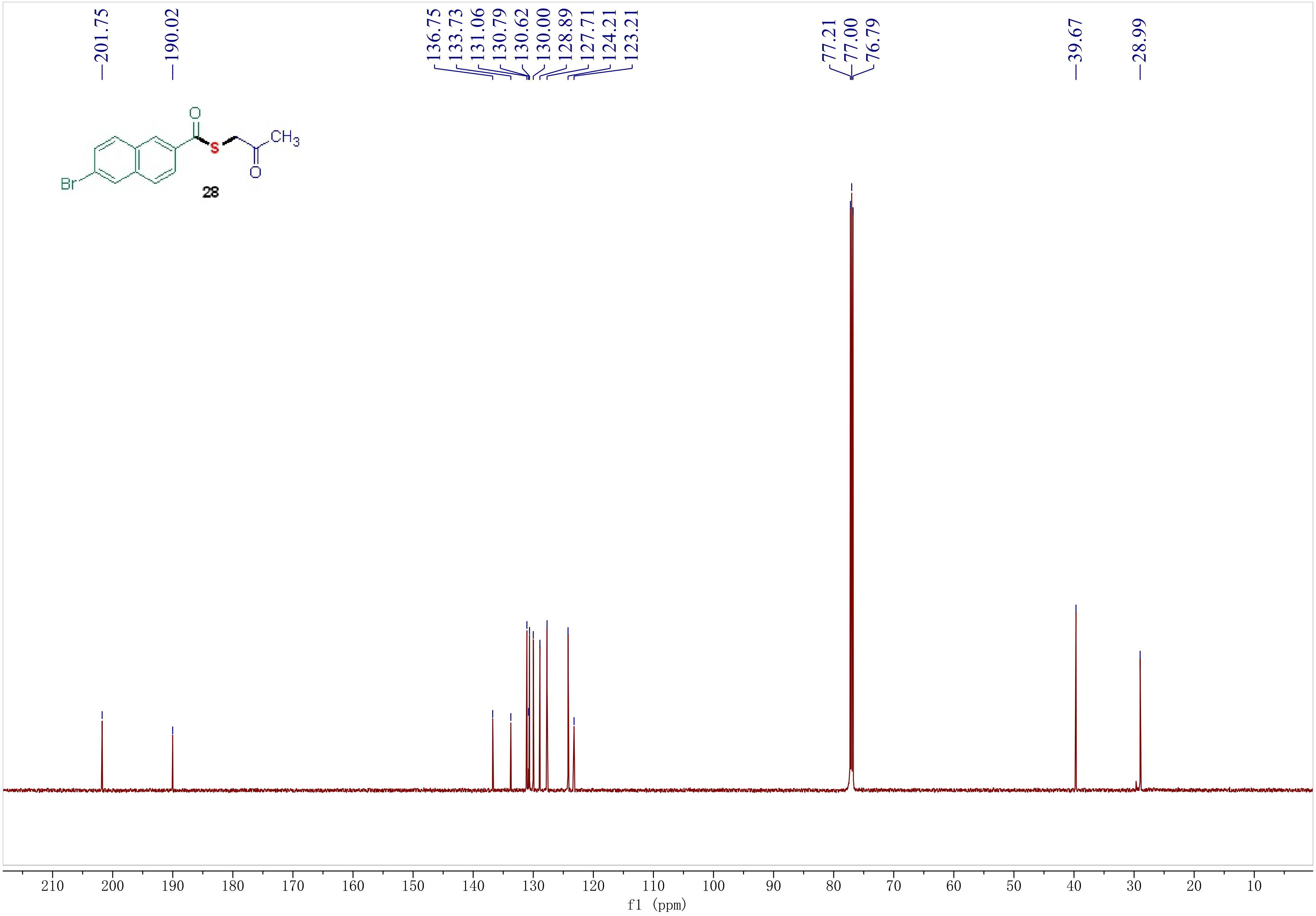

201.75
190.02
136.75
133.73
131.06
130.79
130.62
130.00
128.89
127.71
124.21
123.21
77.21
77.00
76.79
39.67
28.99
Br
S
O
CH3
28
210
200
190
180
170
160
150
140
130
120
110
100
90
80
70
60
50
40
30
20
10
f1 (ppm)

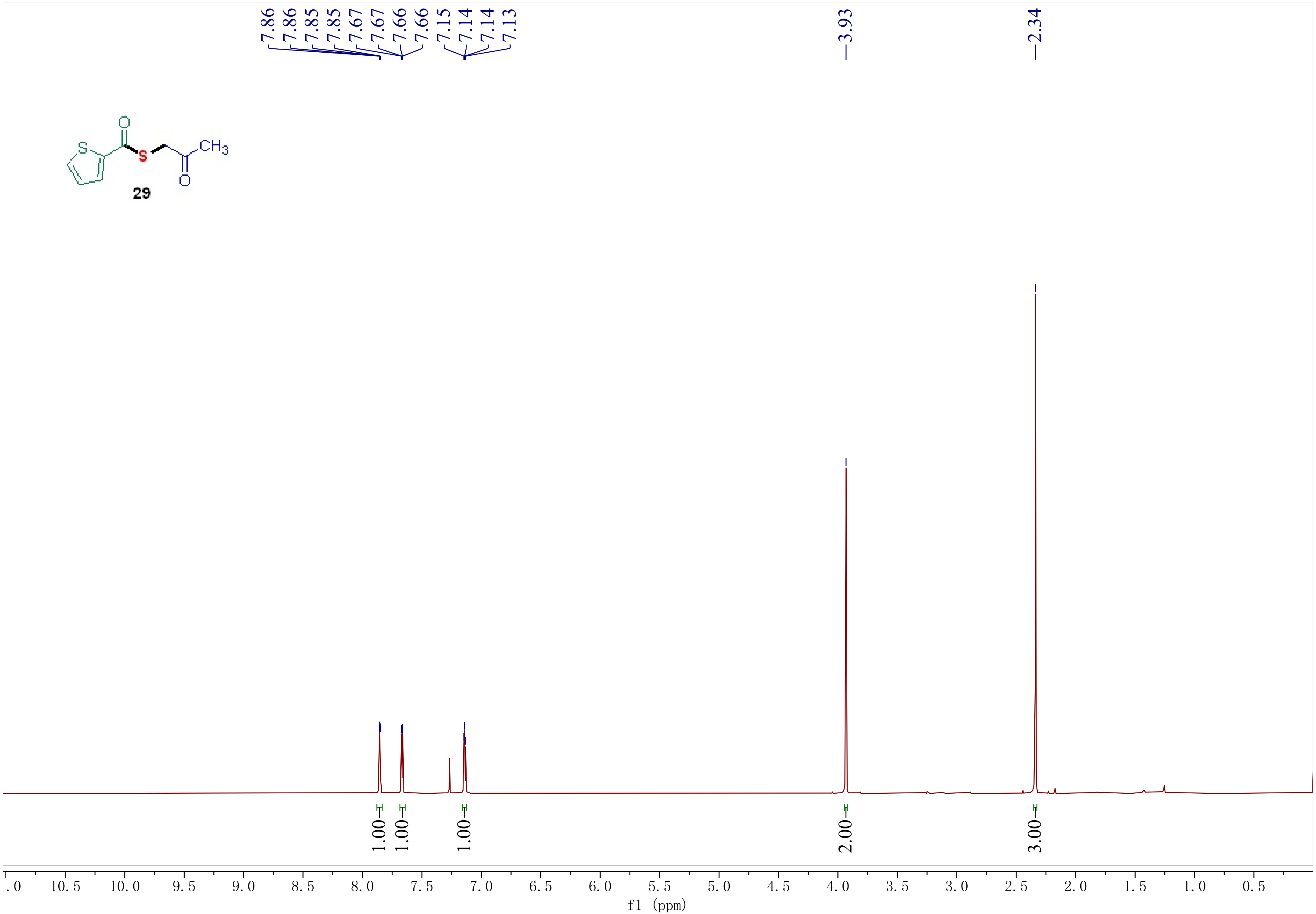

7.86
7.86
7.85
7.85
7.67
7.67
7.66
7.66
7.15
7.14
7.14
7.13
3.93
2.34
O
S
S
CH3
O
29
1.00
1.00
1.00
2.00
3.00
11.0
10.5
10.0
9.5
9.0
8.5
8.0
7.5
7.0
6.5
6.0
5.5
5.0
4.5
4.0
3.5
3.0
2.5
2.0
1.5
1.0
0.5
f1 (ppm)

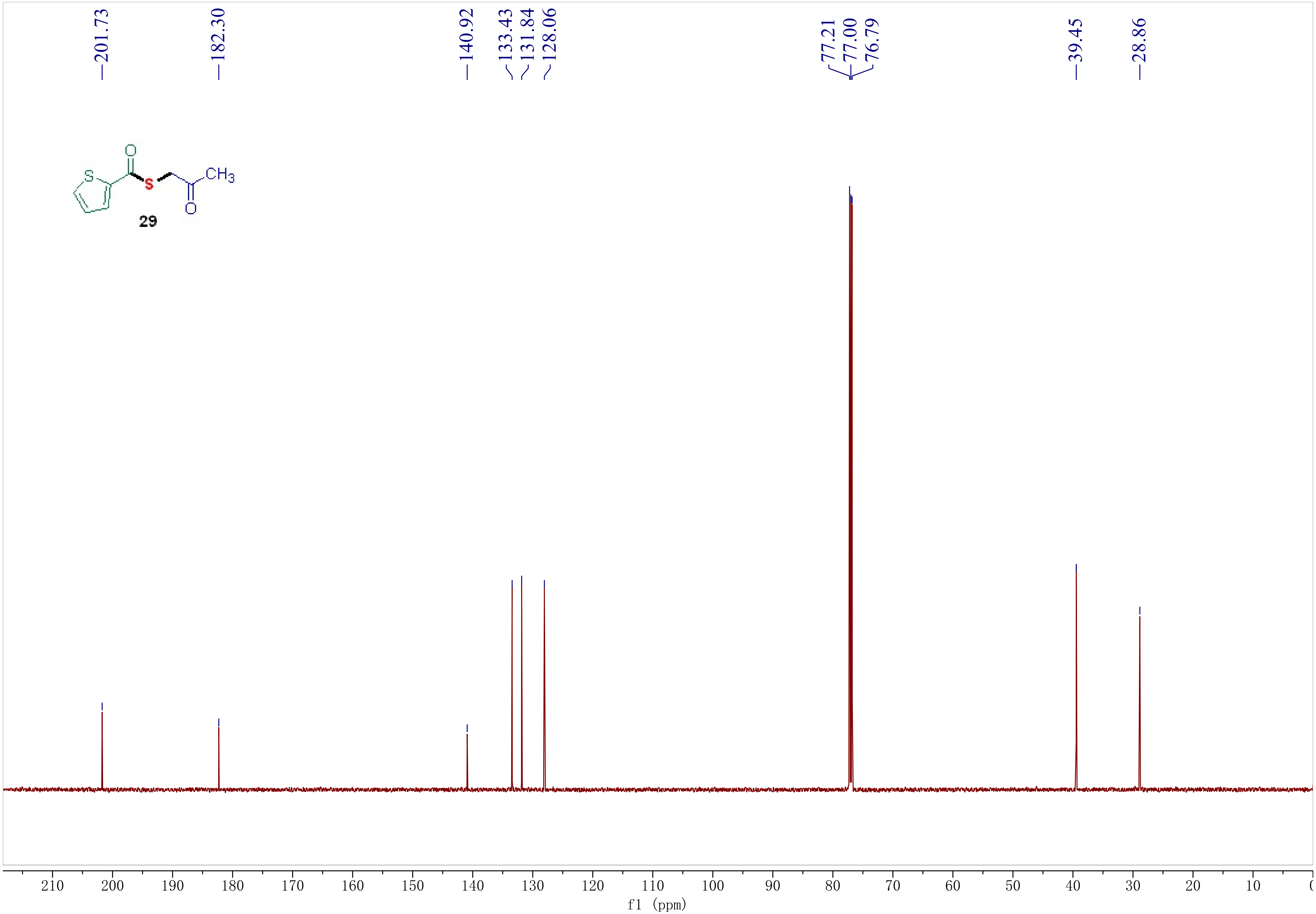
201.73
182.30
140.92
133.43
131.84
128.06
77.21
77.00
76.79
39.45
28.86
CH3
29
210 200 190 180 170 160 150 140 130 120 110 100 90 80 70 60 50 40 30 20 10
f1 (ppm)

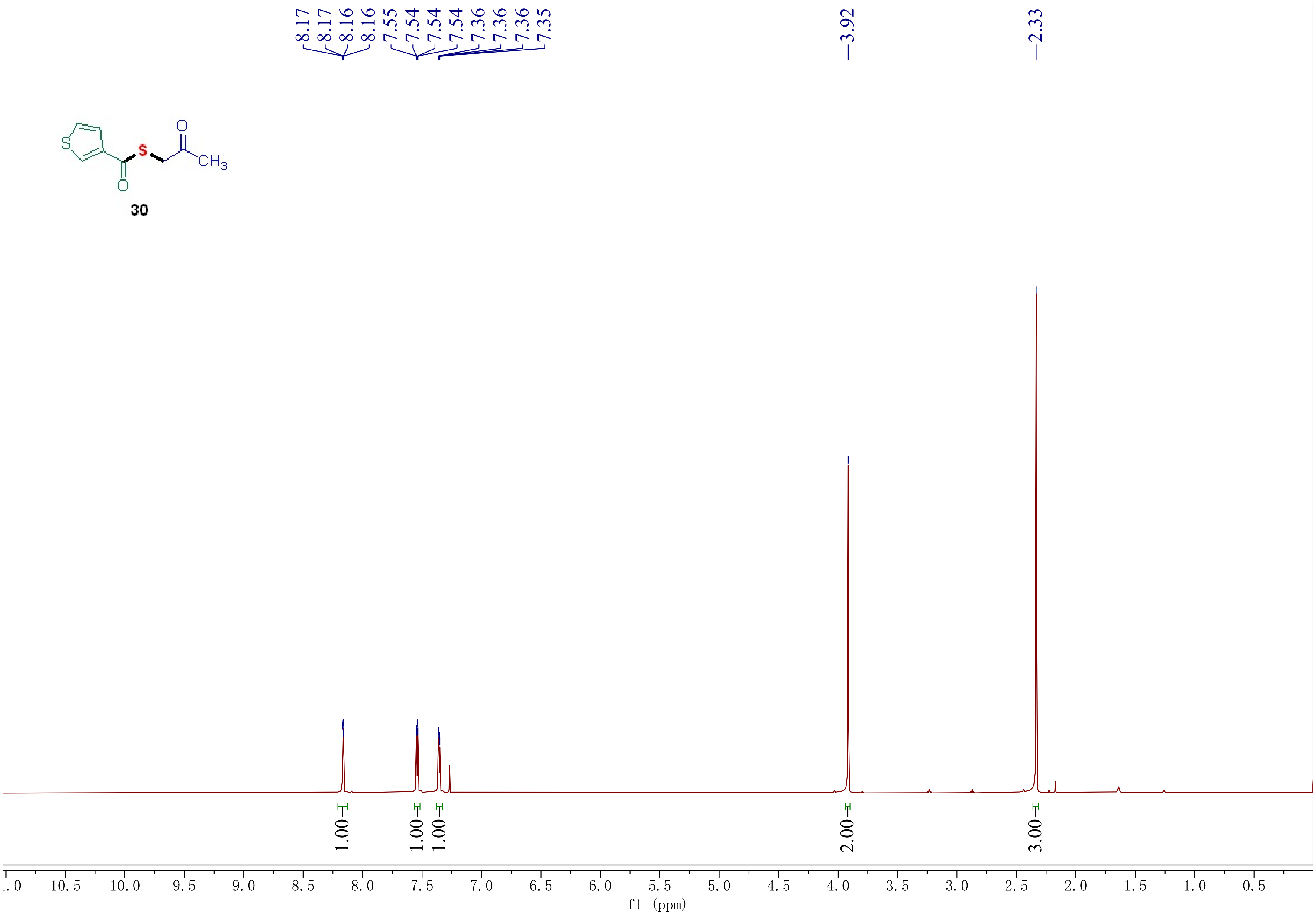

8.17
8.17
8.16
8.16
7.55
7.54
7.54
7.54
7.36
7.36
7.36
7.35
3.92
2.33
S
S
O
O
CH3
30
1.00
1.00
1.00
2.00
3.00
10.5
10.0
9.5
9.0
8.5
8.0
7.5
7.0
6.5
6.0
5.5
5.0
4.5
4.0
3.5
3.0
2.5
2.0
1.5
1.0
0.5
f1 (ppm)

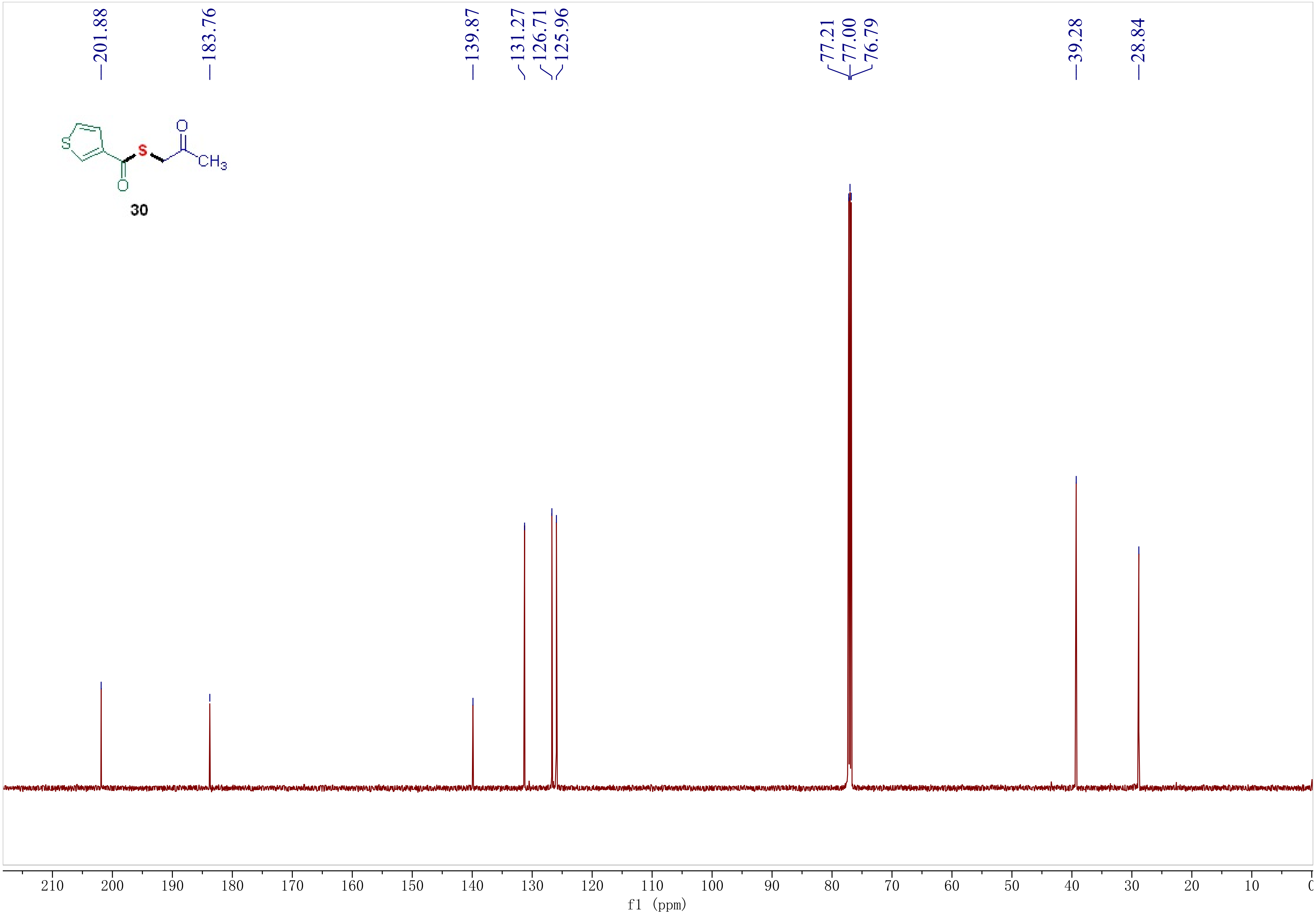

201.88
183.76
139.87
131.27
126.71
125.96
77.21
77.00
76.79
39.28
28.84
S
S
O
O
CH3
30
210
200
190
180
170
160
150
140
130
120
110
100
90
80
70
60
50
40
30
20
10
f1 (ppm)

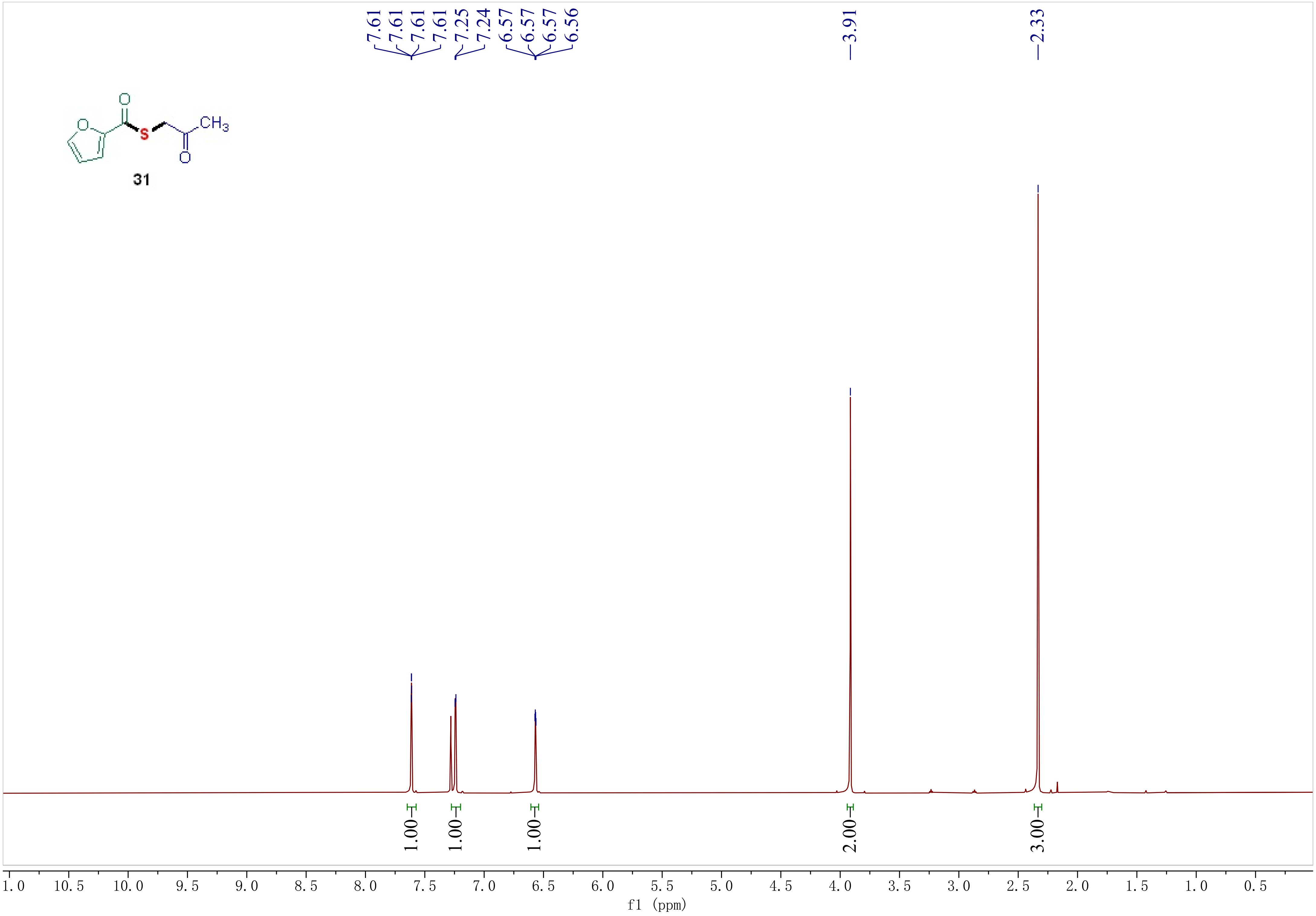

7.61
7.61
7.61
7.61
7.25
7.24
6.57
6.57
6.57
6.56
3.91
2.33
CH3
S
O
31
1.00
1.00
1.00
2.00
3.00
f1 (ppm)

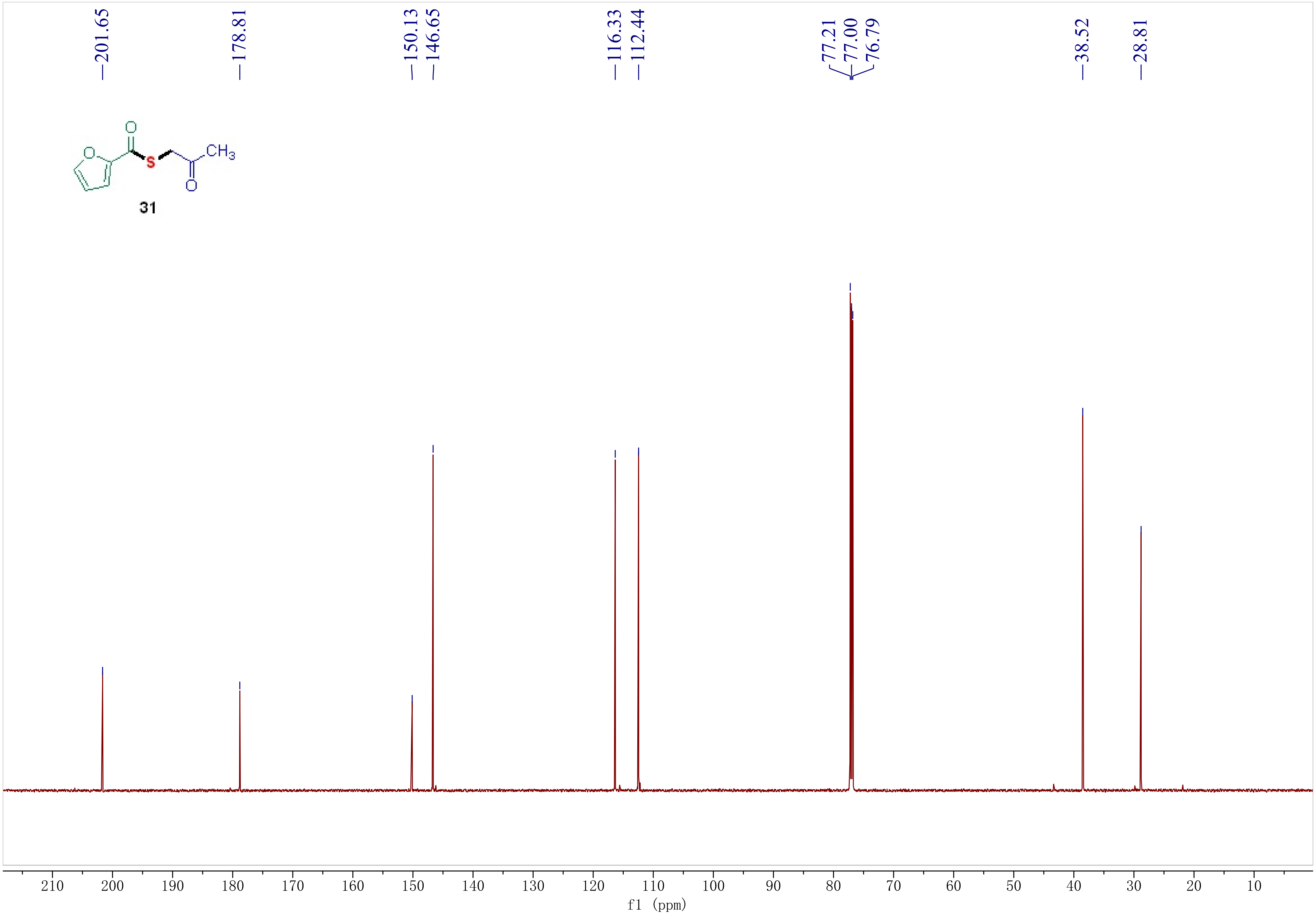

201.65
178.81
150.13
146.65
116.33
112.44
77.21
77.00
76.79
38.52
28.81
CH3
O
S
31
210 200 190 180 170 160 150 140 130 120 110 100 90 80 70 60 50 40 30 20 10
f1 (ppm)

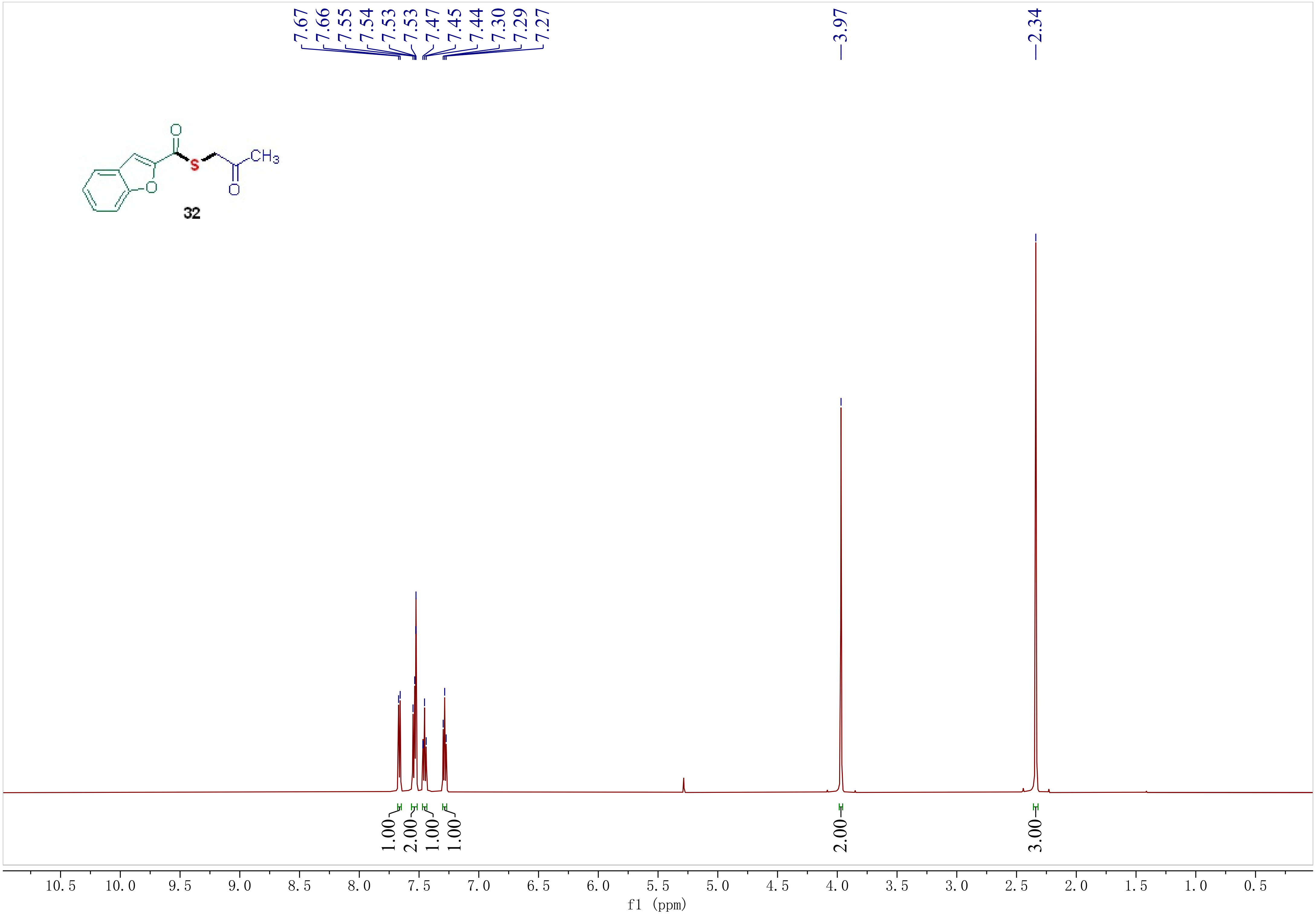
7.67
7.66
7.55
7.54
7.53
7.53
7.47
7.45
7.44
7.30
7.29
7.27
3.97
2.34
CH3
O
S
32
1.00
2.00
1.00
1.00
2.00
3.00
10.5 10.0 9.5 9.0 8.5 8.0 7.5 7.0 6.5 6.0 5.5 5.0 4.5 4.0 3.5 3.0 2.5 2.0 1.5 1.0 0.5
f1 (ppm)

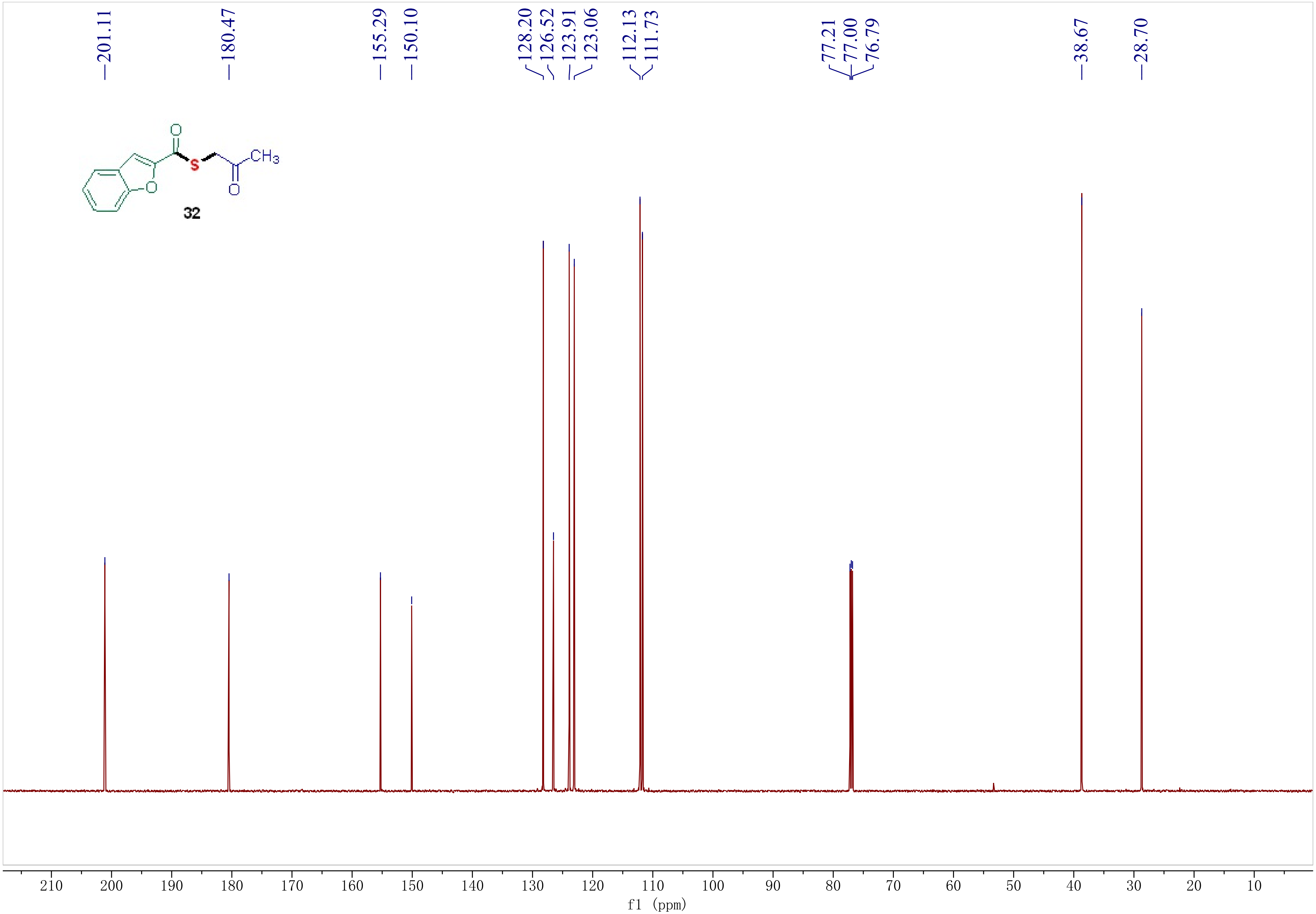

201.11
180.47
155.29
150.10
128.20
126.52
123.91
123.06
112.13
111.73
77.21
77.00
76.79
38.67
28.70
O
S
CH3
O
O
32
210 200 190 180 170 160 150 140 130 120 110 100 90 80 70 60 50 40 30 20 10
f1 (ppm)

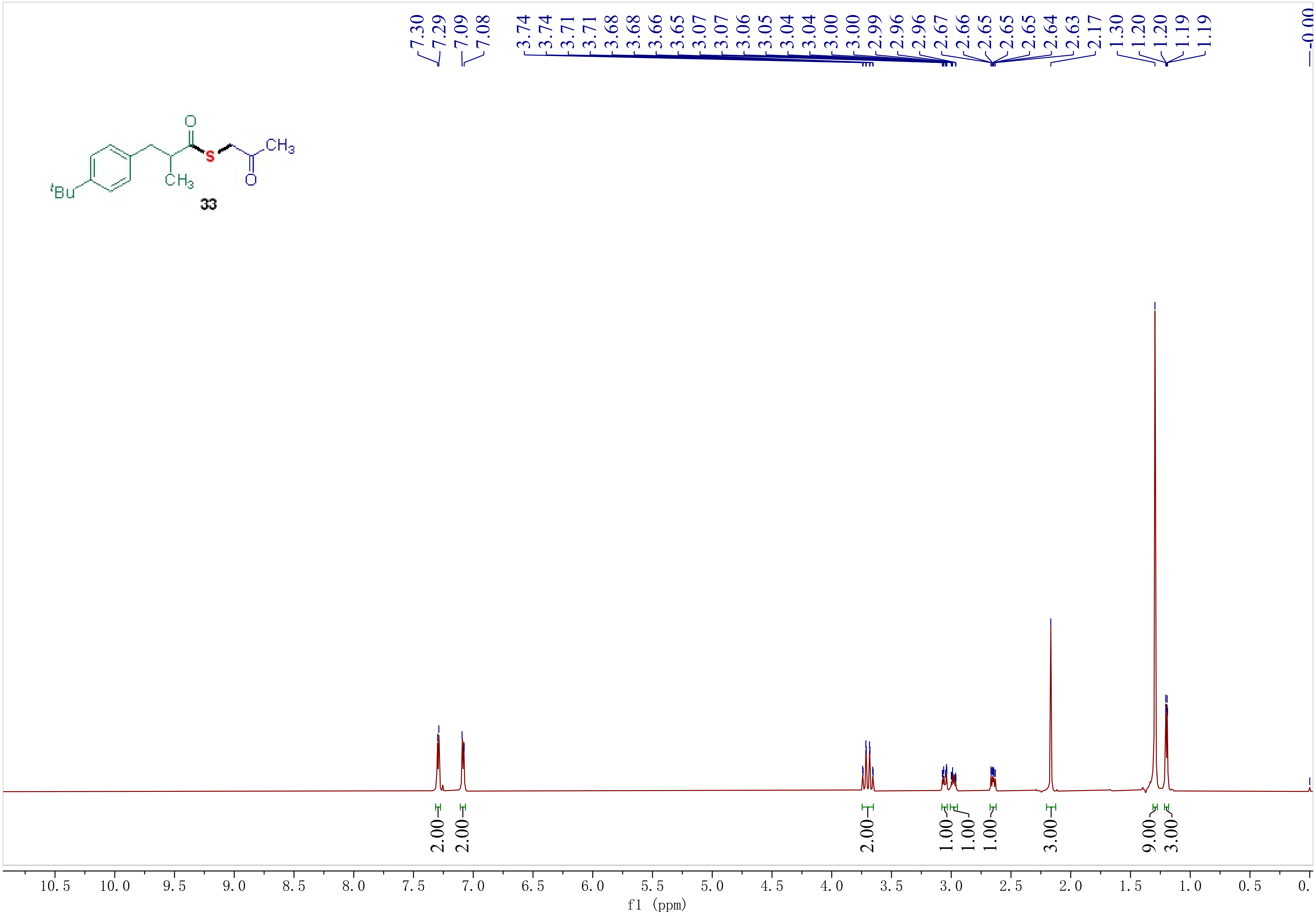

7.30
7.29
7.09
7.08
3.74
3.74
3.71
3.71
3.68
3.68
3.66
3.65
3.07
3.07
3.06
3.05
3.04
3.04
3.00
3.00
2.99
2.96
2.96
2.67
2.66
2.65
2.65
2.65
2.64
2.63
2.17
1.30
1.20
1.20
1.19
1.19
-0.00
tBu
O
S
CH3
CH3
O
33
2.00
2.00
2.00
1.00
1.00
1.00
3.00
9.00
3.00
10.5
10.0
9.5
9.0
8.5
8.0
7.5
7.0
6.5
6.0
5.5
5.0
4.5
4.0
3.5
3.0
2.5
2.0
1.5
1.0
0.5
0.
f1 (ppm)

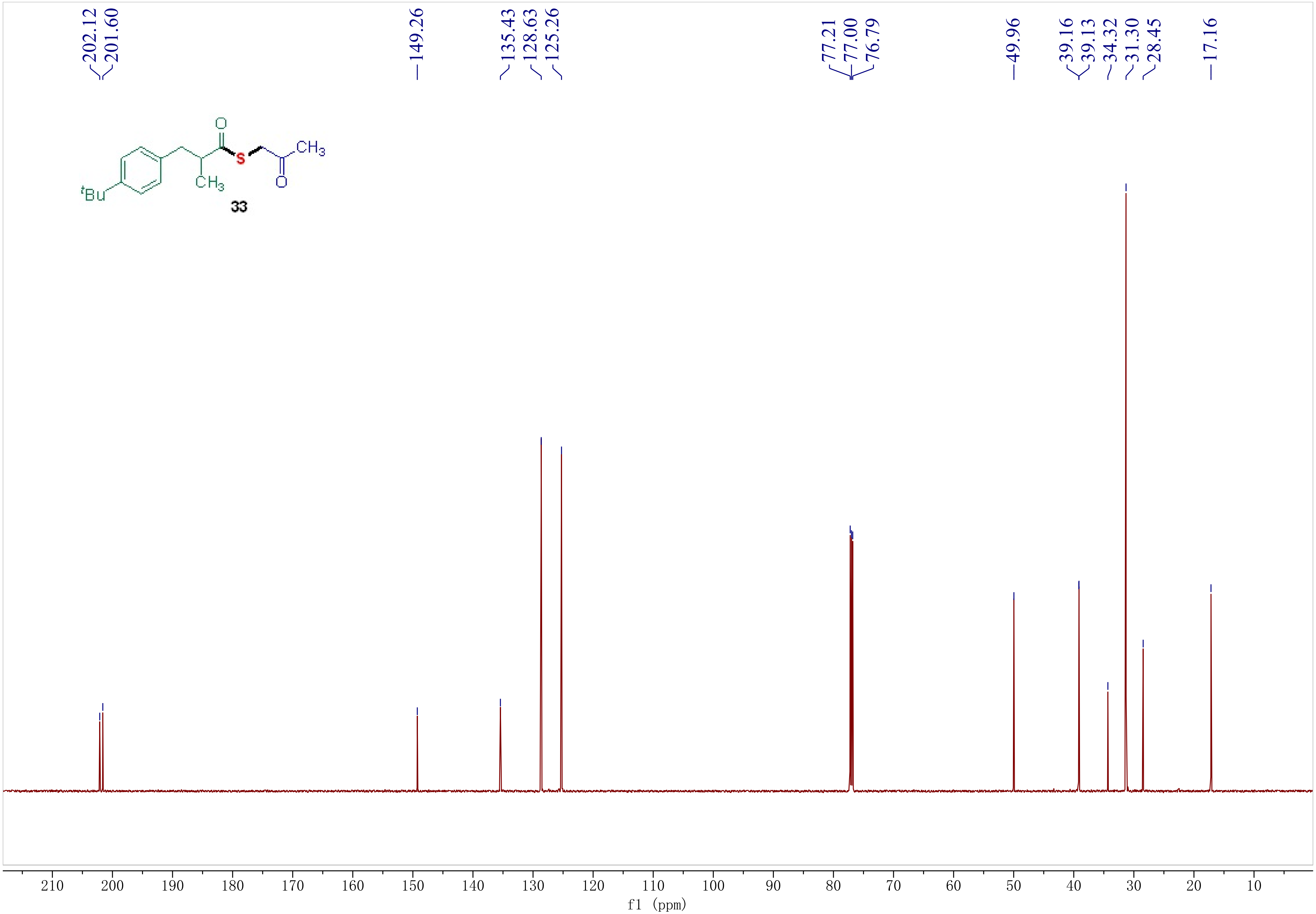

202.12
201.60
149.26
135.43
128.63
125.26
77.21
77.00
76.79
49.96
39.16
39.13
34.32
31.30
28.45
17.16
O
S
CH3
tBu
CH3
O
33
210 200 190 180 170 160 150 140 130 120 110 100 90 80 70 60 50 40 30 20 10
f1 (ppm)

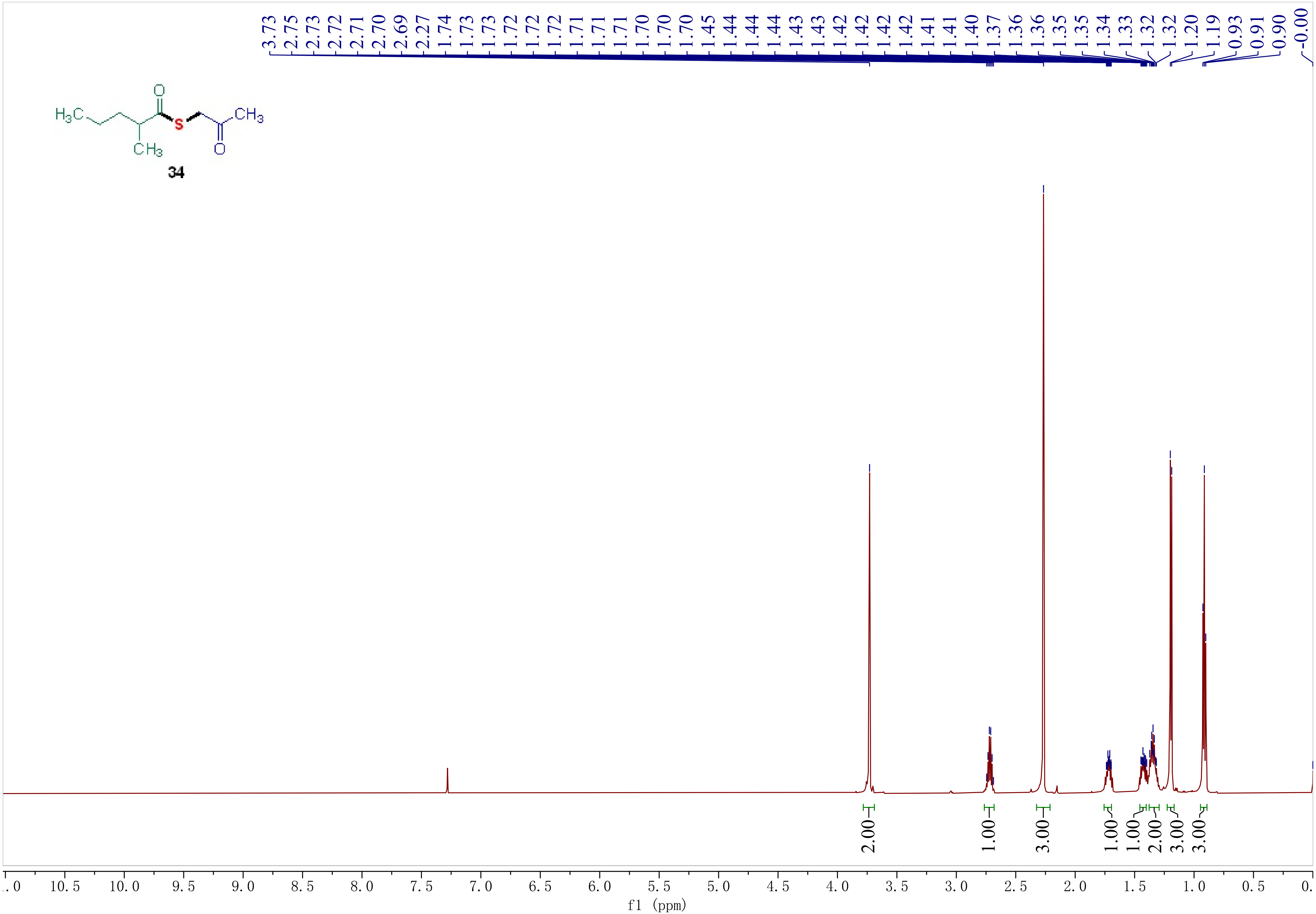

3.73
2.75
2.73
2.72
2.71
2.70
2.69
2.27
1.74
1.73
1.73
1.72
1.72
1.72
1.71
1.71
1.71
1.70
1.70
1.70
1.45
1.44
1.44
1.44
1.43
1.43
1.42
1.42
1.42
1.42
1.41
1.41
1.40
1.37
1.36
1.36
1.35
1.35
1.34
1.33
1.32
1.32
1.20
1.19
0.93
0.91
0.90
-0.00
34
2.00
1.00
3.00
1.00
1.00
2.00
3.00
3.00
10.5
10.0
9.5
9.0
8.5
8.0
7.5
7.0
6.5
6.0
5.5
5.0
4.5
4.0
3.5
3.0
2.5
2.0
1.5
1.0
0.5
f1 (ppm)

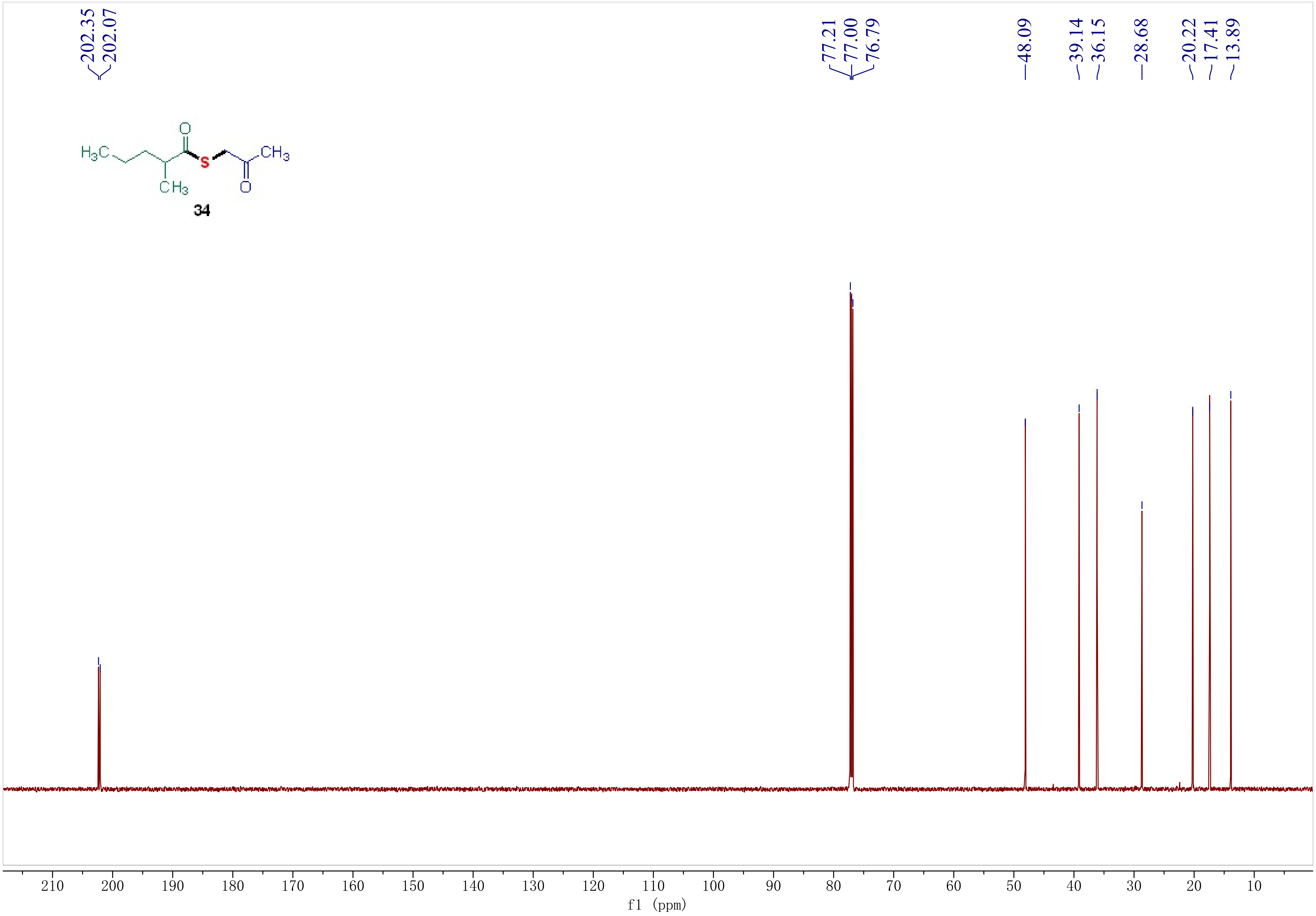

202.35
202.07
77.21
77.00
76.79
48.09
39.14
36.15
28.68
20.22
17.41
13.89
34
210
200
190
180
170
160
150
140
130
120
110
100
90
80
70
60
50
40
30
20
10
f1 (ppm)

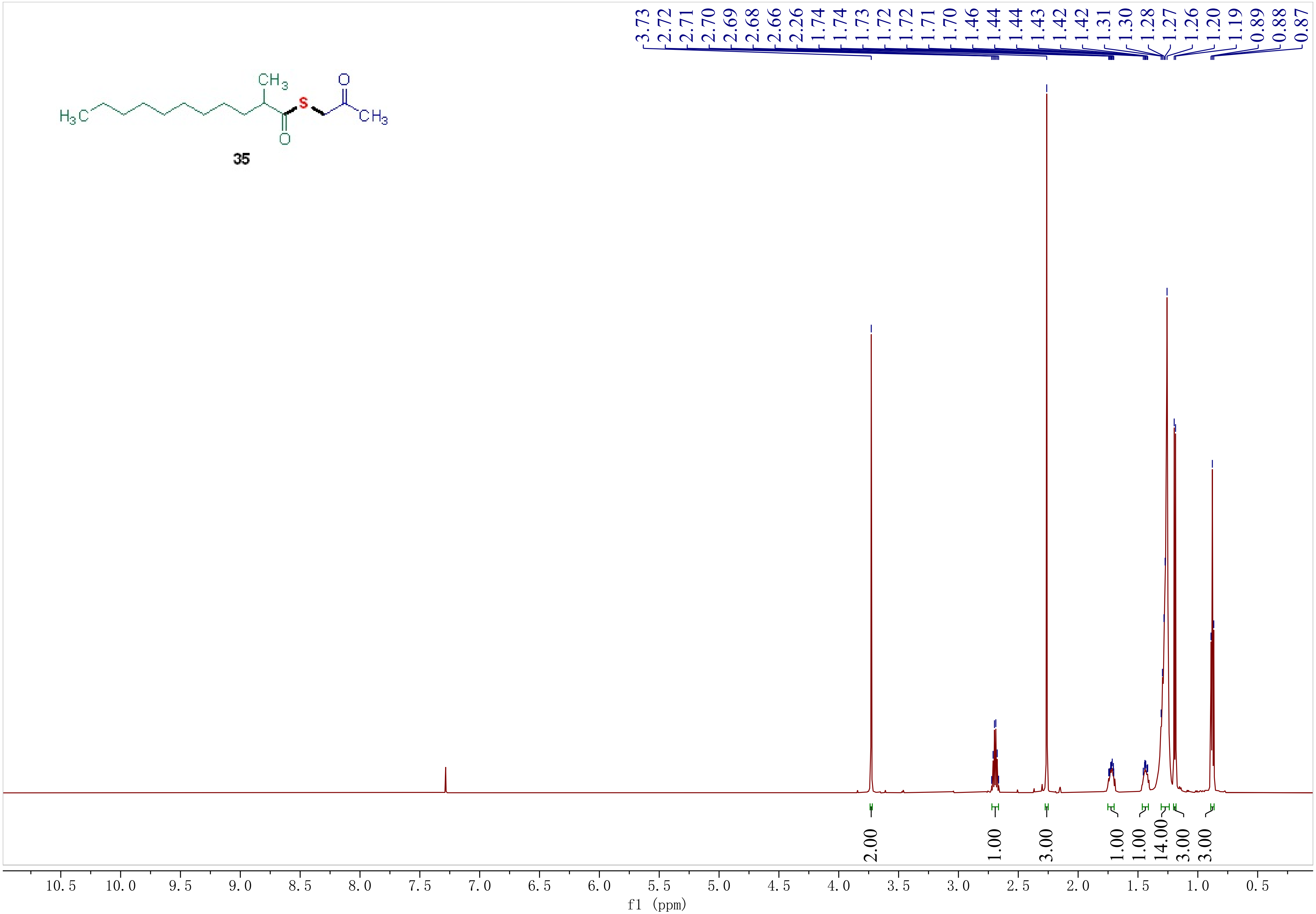
3.73 2.72 2.71 2.70 2.69 2.68 2.66 2.26 1.74 1.74 1.73 1.72 1.72 1.71 1.70 1.46 1.44 1.44 1.43 1.42 1.42 1.31 1.30 1.28 1.27 1.26 1.20 1.19 0.89 0.88 0.87
CH3
H3C
S
O
O
CH3
35
2.00
1.00
3.00
1.00
1.00
14.00
3.00
3.00
10.5 10.0 9.5 9.0 8.5 8.0 7.5 7.0 6.5 6.0 5.5 5.0 4.5 4.0 3.5 3.0 2.5 2.0 1.5 1.0 0.5
f1 (ppm)

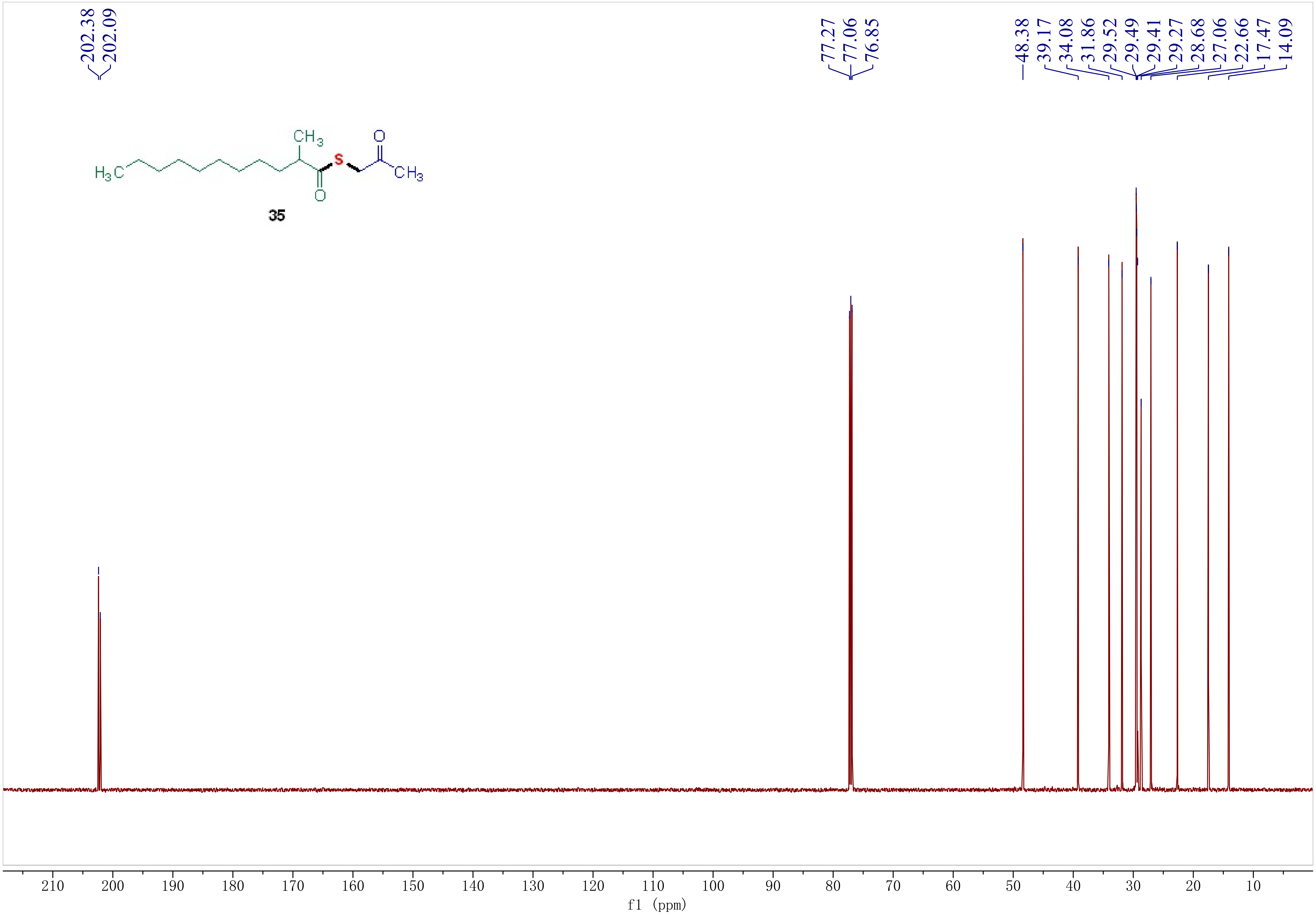
202.38
202.09
77.27
77.06
76.85
48.38
39.17
34.08
31.86
29.52
29.49
29.41
29.27
28.68
27.06
22.66
17.47
14.09
35
210
200
190
180
170
160
150
140
130
120
110
100
90
80
70
60
50
40
30
20
10
f1 (ppm)

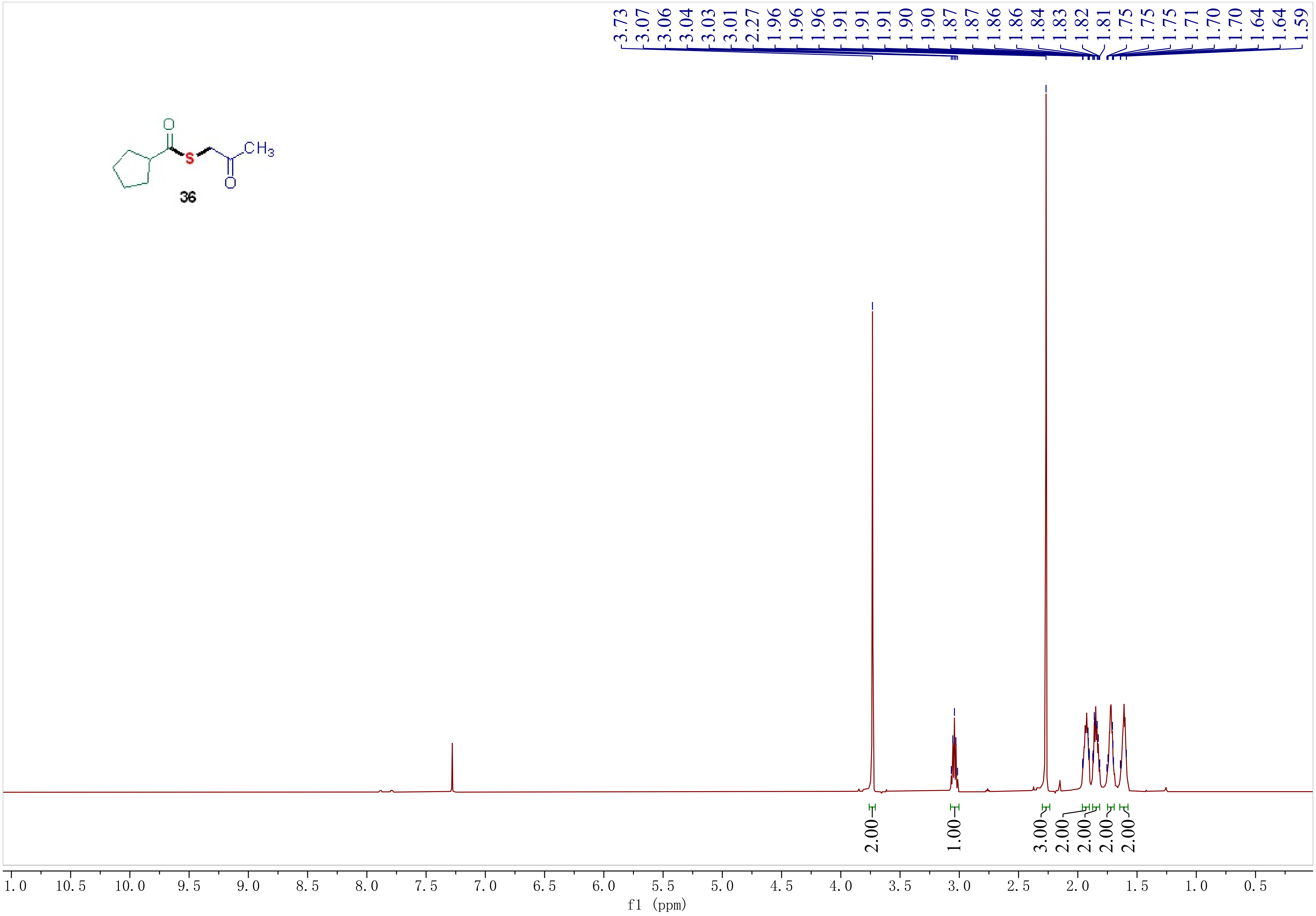

3.73
3.07
3.06
3.04
3.03
3.01
2.27
1.96
1.96
1.96
1.91
1.91
1.91
1.90
1.90
1.87
1.87
1.86
1.86
1.84
1.83
1.82
1.81
1.75
1.75
1.75
1.71
1.70
1.70
1.64
1.64
1.59
CH3
S
O
O
36
2.00
1.00
3.00
2.00
2.00
2.00
2.00
1.0
10.5
10.0
9.5
9.0
8.5
8.0
7.5
7.0
6.5
6.0
5.5
5.0
4.5
4.0
3.5
3.0
2.5
2.0
1.5
1.0
0.5
f1 (ppm)

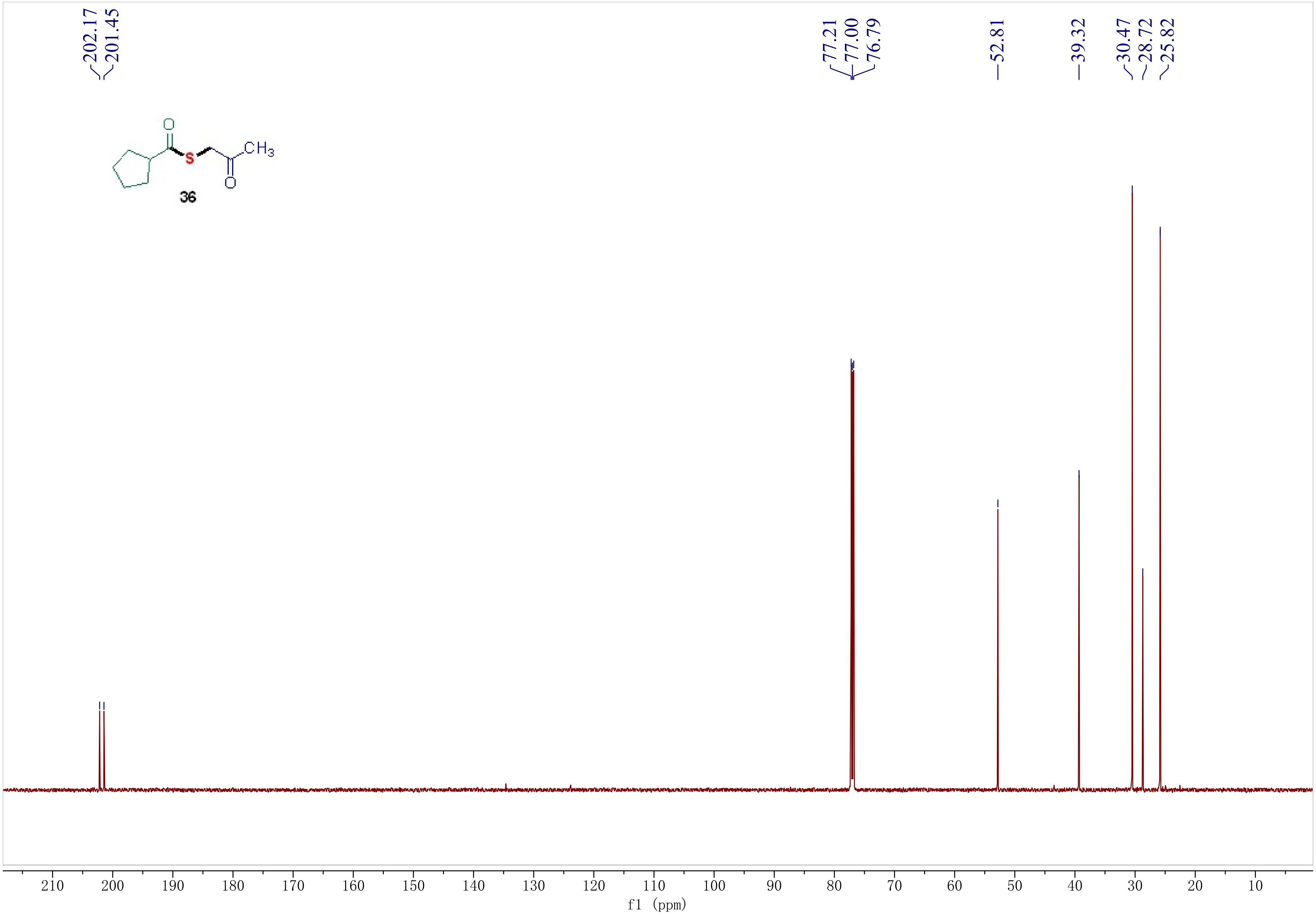
202.17
201.45
77.21
77.00
76.79
52.81
39.32
30.47
28.72
25.82
CH3
S
O
36
210
200
190
180
170
160
150
140
130
120
110
100
90
80
70
60
50
40
30
20
10
f1 (ppm)

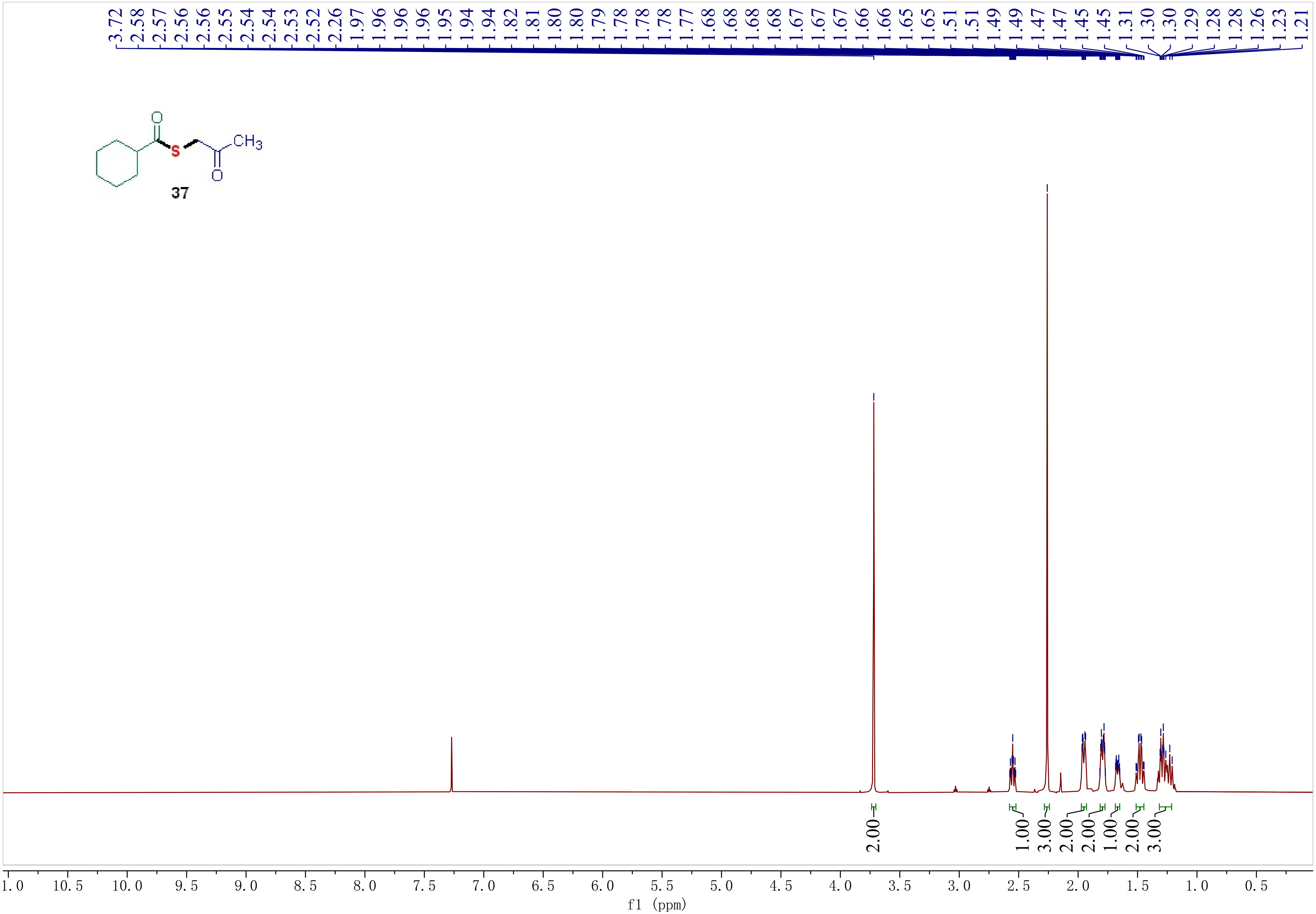
3.72 2.58 2.57 2.56 2.56 2.55 2.54 2.54 2.53 2.52 2.26 1.97 1.96 1.96 1.96 1.95 1.94 1.94 1.82 1.81 1.80 1.80 1.79 1.78 1.78 1.78 1.77 1.68 1.68 1.68 1.68 1.67 1.67 1.67 1.66 1.66 1.65 1.65 1.51 1.51 1.49 1.49 1.47 1.47 1.45 1.45 1.31 1.30 1.30 1.29 1.28 1.28 1.26 1.23 1.21
O
S
CH3
O
37
2.00
1.00
3.00
2.00
2.00
1.00
2.00
3.00
1.0 10.5 10.0 9.5 9.0 8.5 8.0 7.5 7.0 6.5 6.0 5.5 5.0 4.5 4.0 3.5 3.0 2.5 2.0 1.5 1.0 0.5
f1 (ppm)

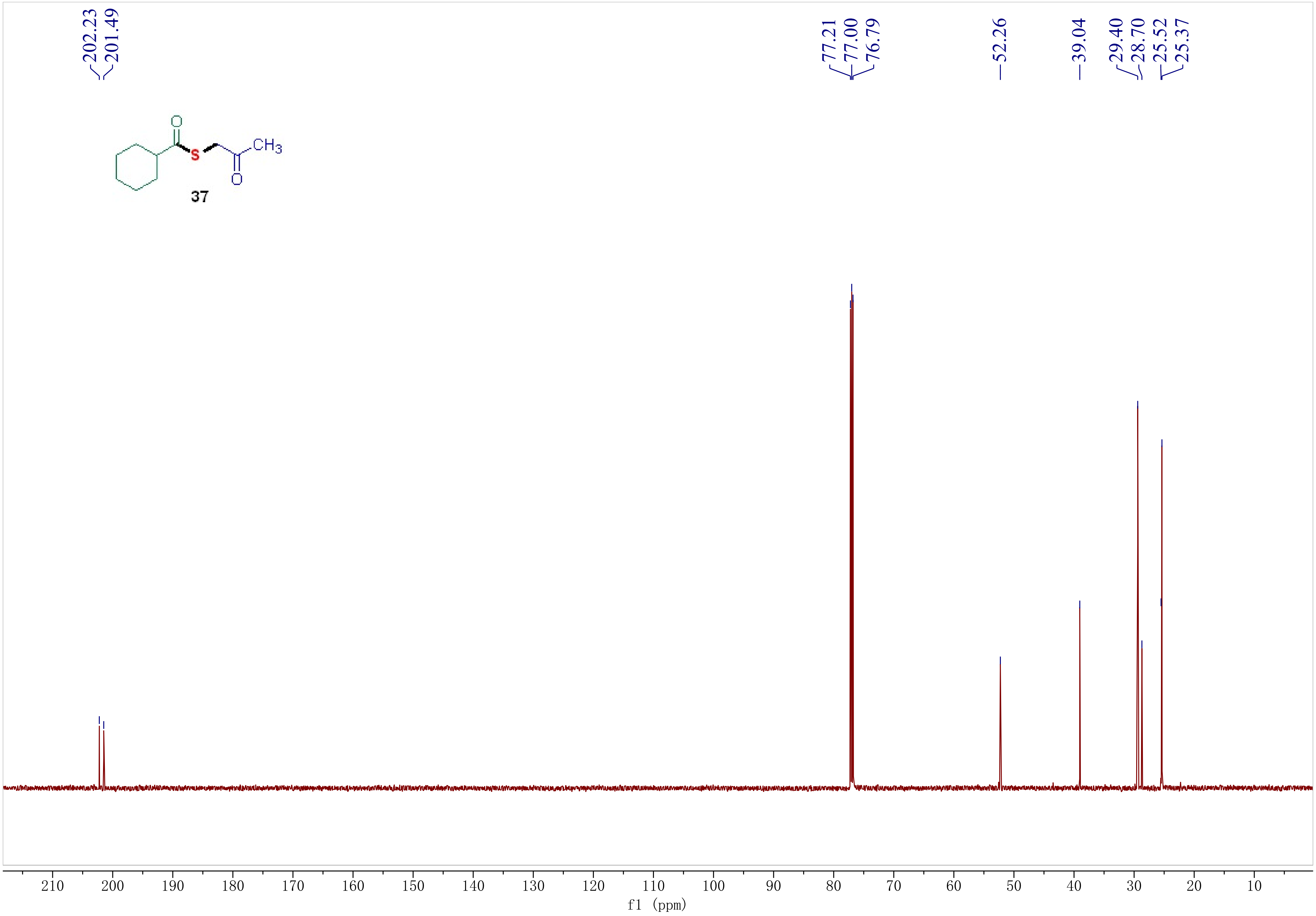
202.23
201.49
77.21
77.00
76.79
52.26
39.04
29.40
28.70
25.52
25.37
37
210 200 190 180 170 160 150 140 130 120 110 100 90 80 70 60 50 40 30 20 10
f1 (ppm)

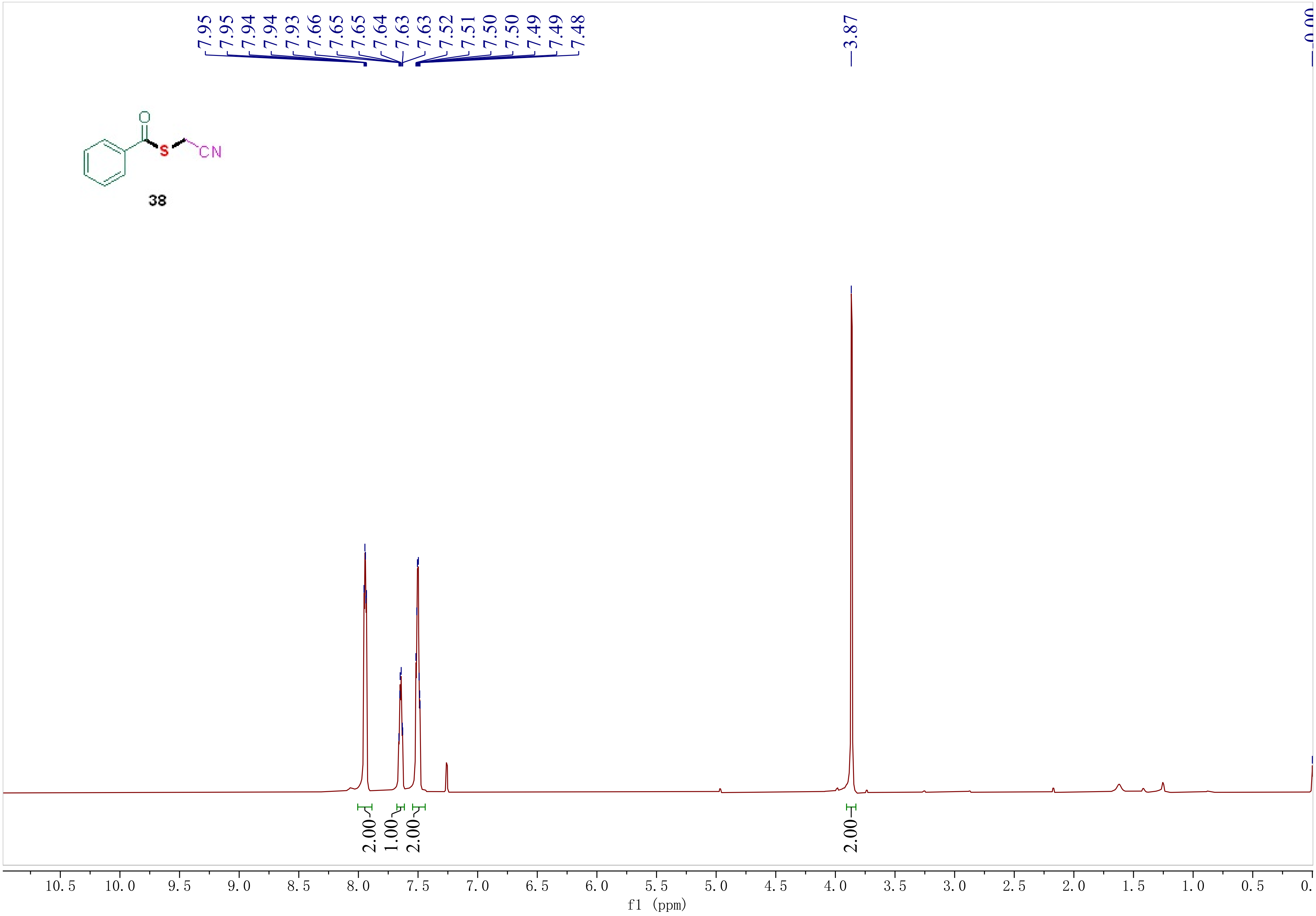
7.95
7.95
7.94
7.94
7.93
7.66
7.65
7.65
7.64
7.63
7.63
7.52
7.51
7.50
7.50
7.49
7.49
7.48
3.87
0.00
O
S
CN
38
2.00
1.00
2.00
2.00
10.5
10.0
9.5
9.0
8.5
8.0
7.5
7.0
6.5
6.0
5.5
5.0
4.5
4.0
3.5
3.0
2.5
2.0
1.5
1.0
0.5
0.
f1 (ppm)

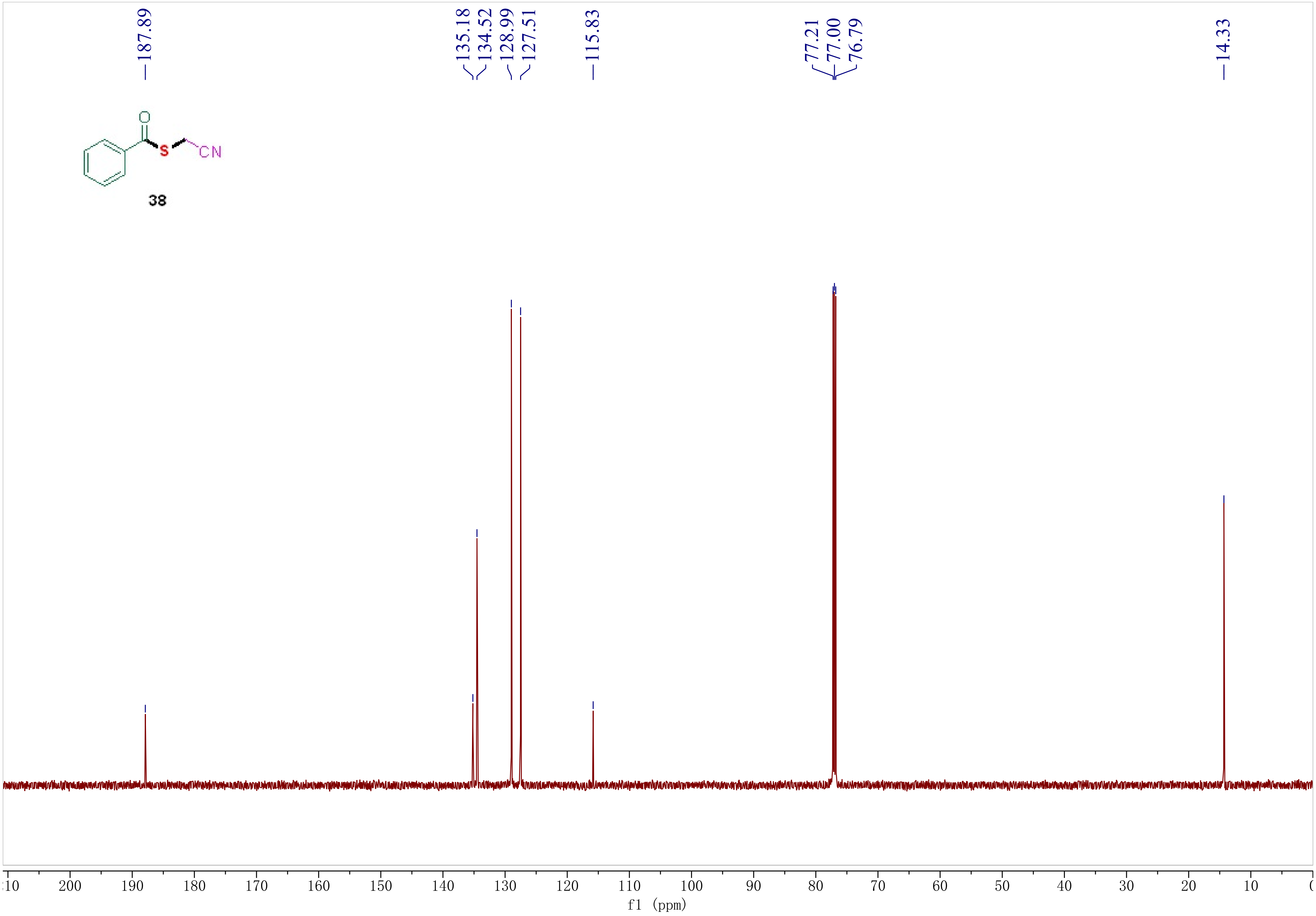
187.89
135.18
134.52
128.99
127.51
115.83
77.21
77.00
76.79
14.33
O
S
CN
38
210
200
190
180
170
160
150
140
130
120
110
100
90
80
70
60
50
40
30
20
10
0
f1 (ppm)

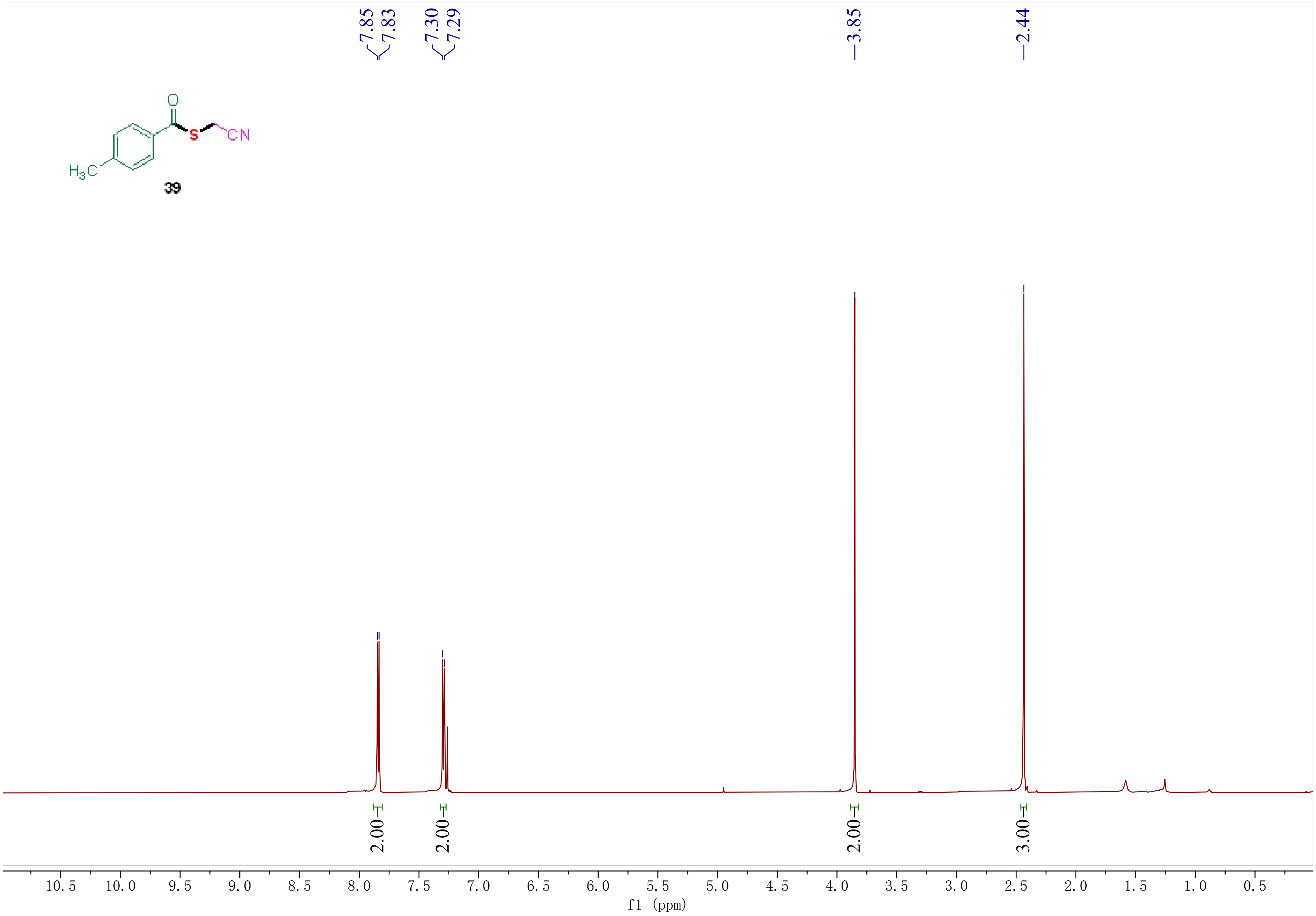
7.85
7.83
7.30
7.29
3.85
2.44
O
S
CN
H3C
39
2.00
2.00
2.00
3.00
10.5
10.0
9.5
9.0
8.5
8.0
7.5
7.0
6.5
6.0
5.5
5.0
4.5
4.0
3.5
3.0
2.5
2.0
1.5
1.0
0.5
f1 (ppm)

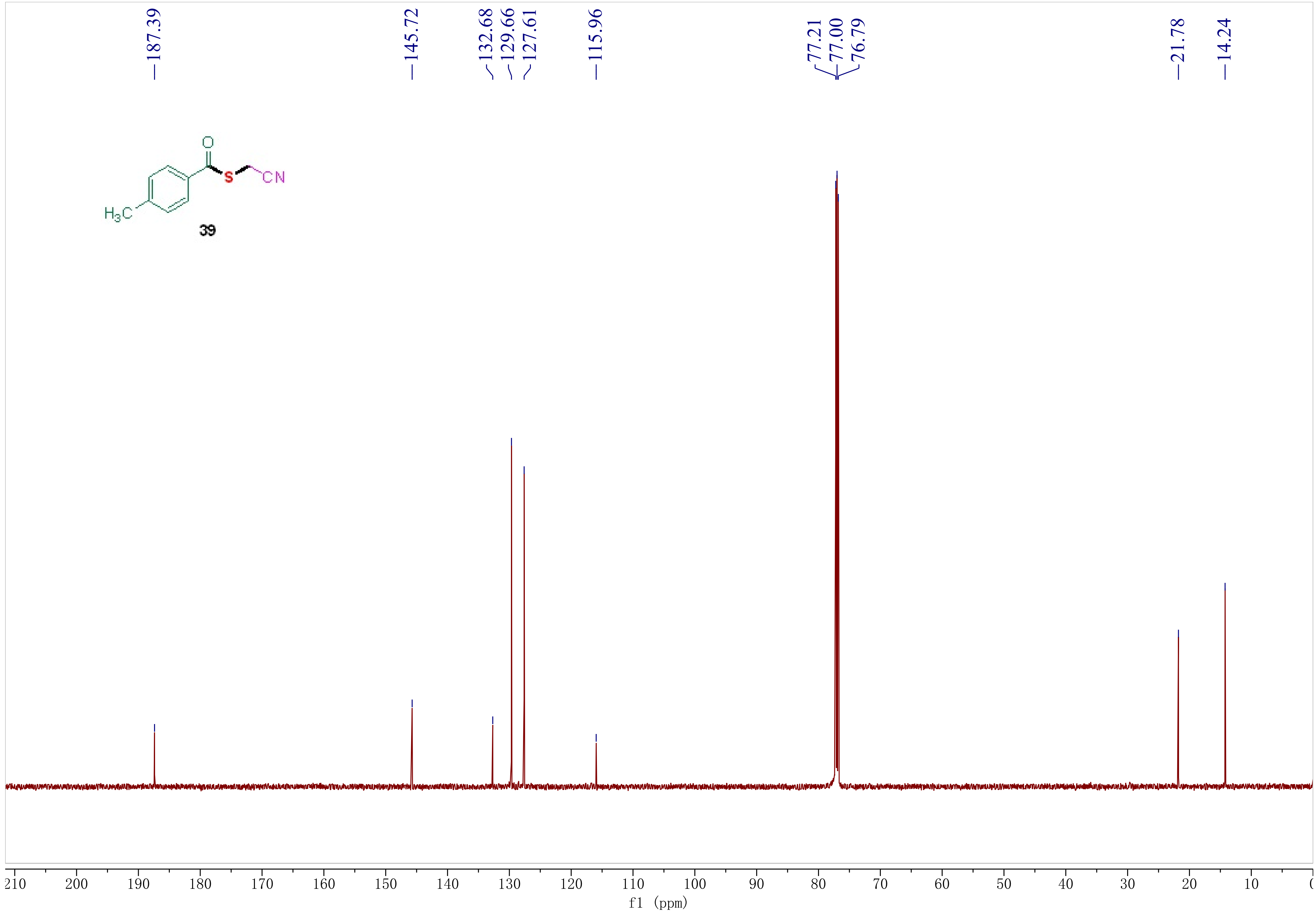

187.39
145.72
132.68
129.66
127.61
115.96
77.21
77.00
76.79
21.78
14.24
O
S
CN
H3C
39
210
200
190
180
170
160
150
140
130
120
110
100
90
80
70
60
50
40
30
20
10
f1 (ppm)

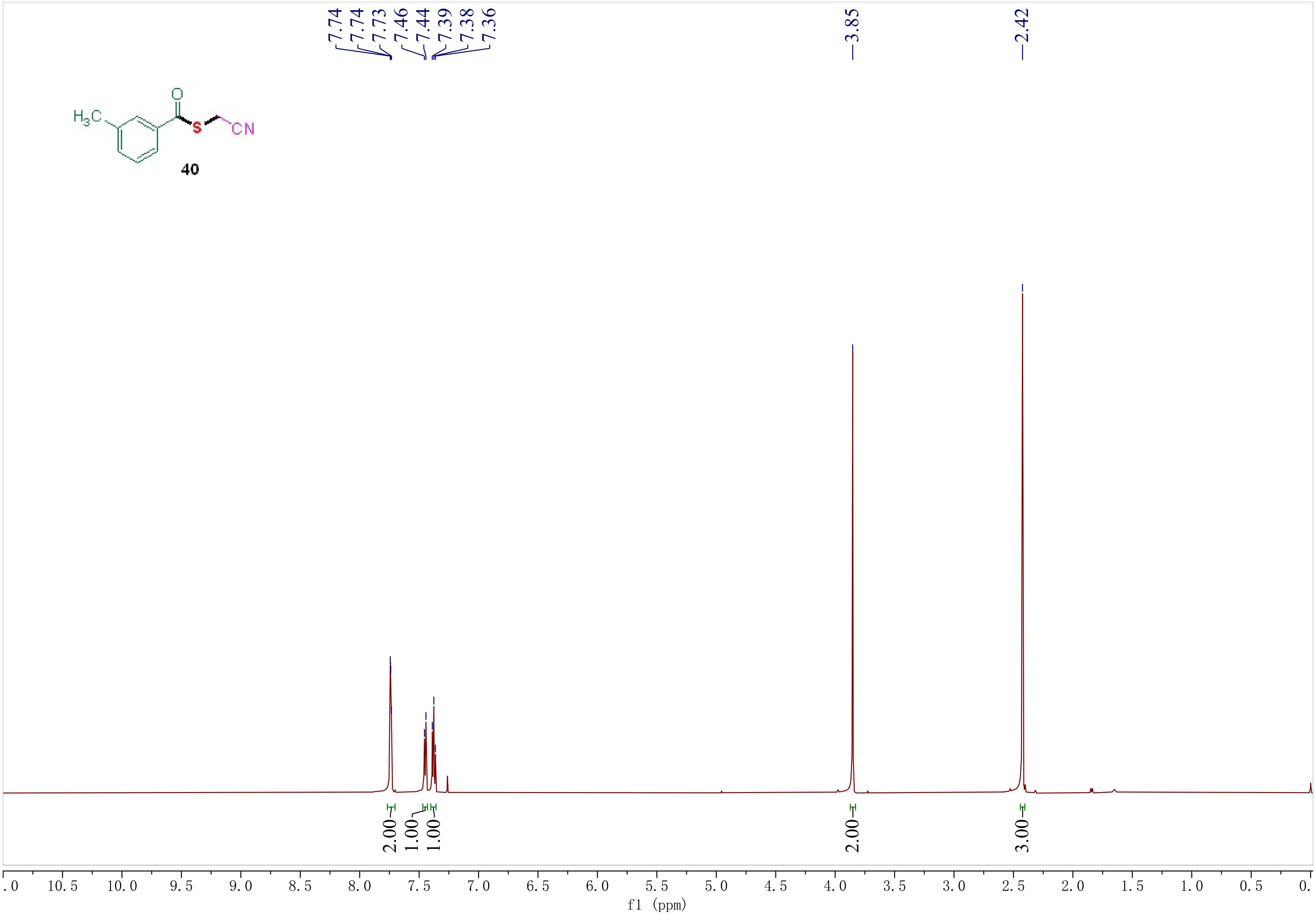
7.74
7.74
7.73
7.46
7.44
7.39
7.38
7.36
3.85
2.42
H3C
O
S
CN
40
2.00
1.00
1.00
2.00
3.00
.0
10.5
10.0
9.5
9.0
8.5
8.0
7.5
7.0
6.5
6.0
5.5
5.0
4.5
4.0
3.5
3.0
2.5
2.0
1.5
1.0
0.5
0.
f1 (ppm)

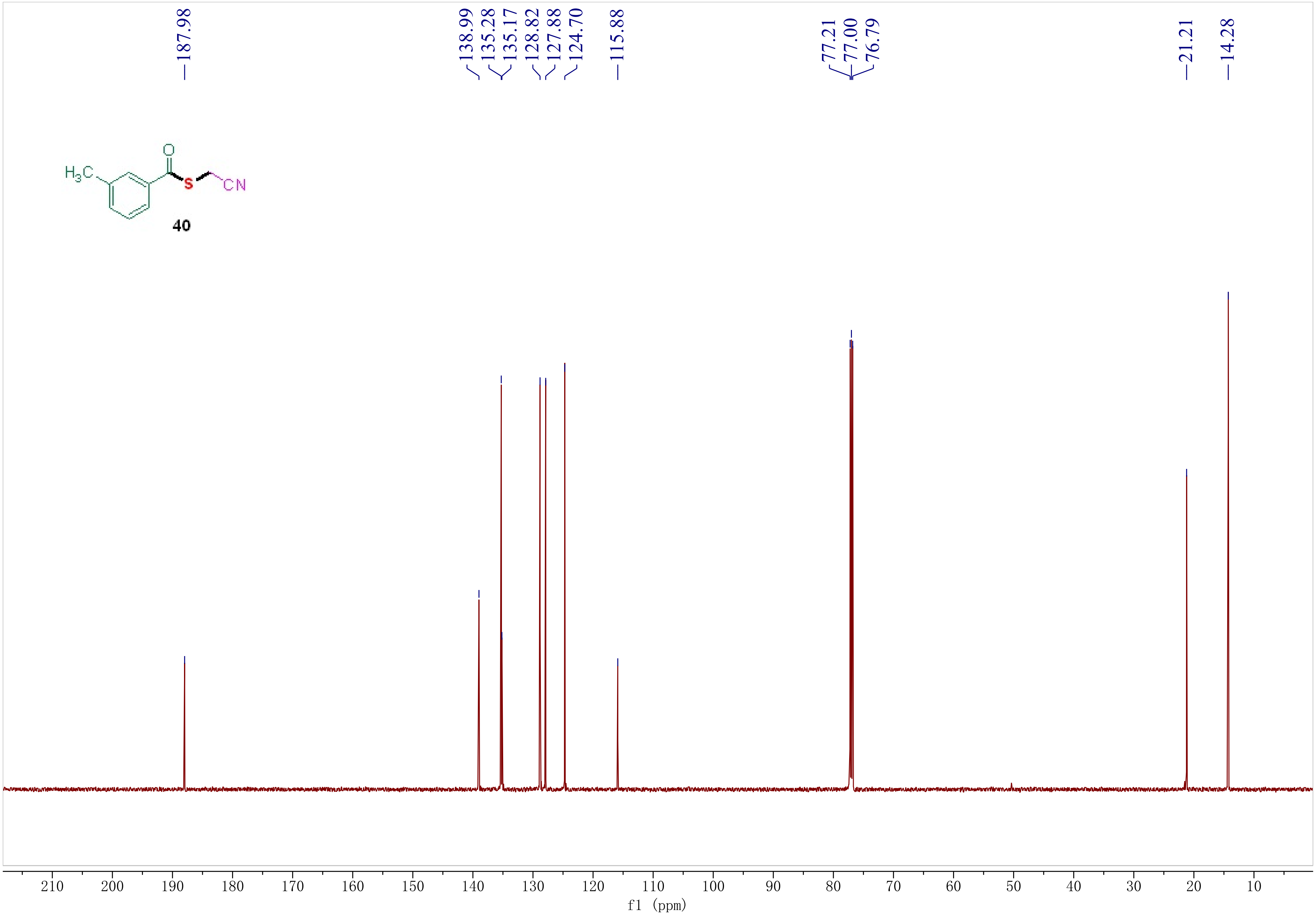

187.98
138.99
135.28
135.17
128.82
127.88
124.70
115.88
77.21
77.00
76.79
21.21
14.28
H_3C
O
S
CN
40
210 200 190 180 170 160 150 140 130 120 110 100 90 80 70 60 50 40 30 20 10
f1 (ppm)

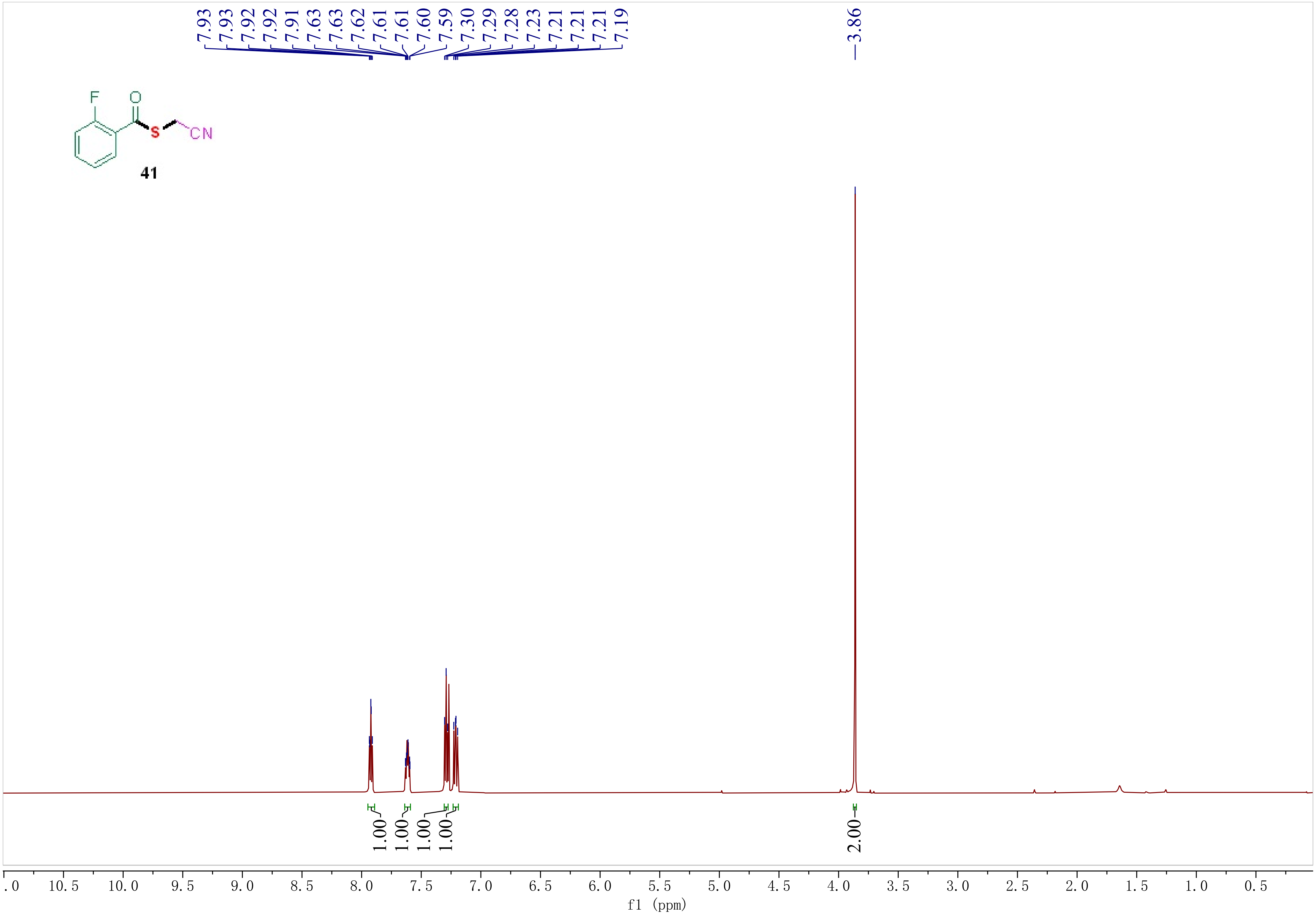
7.93
7.93
7.92
7.92
7.91
7.63
7.63
7.62
7.61
7.61
7.60
7.59
7.30
7.29
7.28
7.23
7.21
7.21
7.21
7.19
3.86
F
O
S
CN
41
1.00
1.00
1.00
1.00
2.00
.0
10.5
10.0
9.5
9.0
8.5
8.0
7.5
7.0
6.5
6.0
5.5
5.0
4.5
4.0
3.5
3.0
2.5
2.0
1.5
1.0
0.5
f1 (ppm)

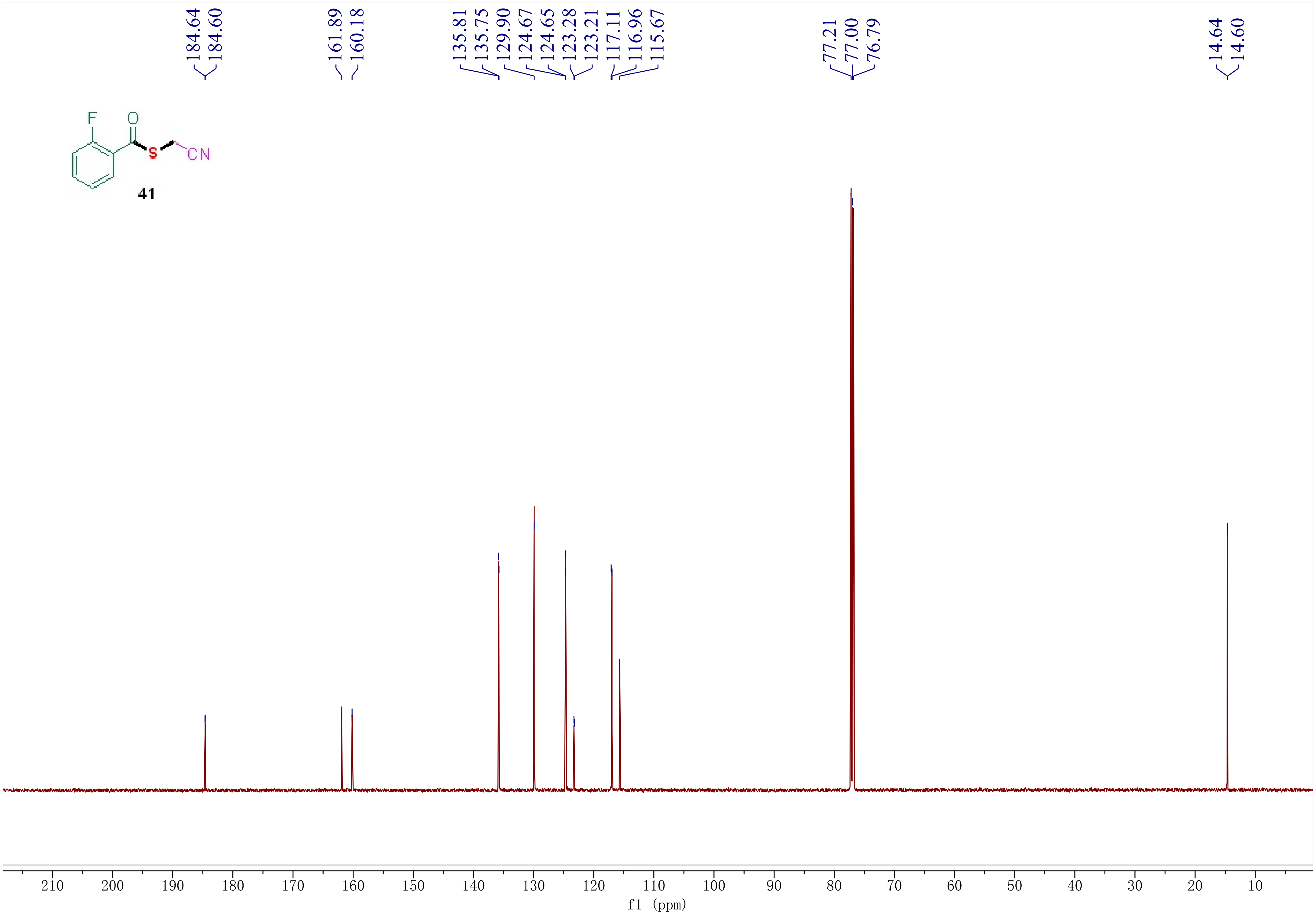
184.64
184.60
161.89
160.18
135.81
135.75
129.90
124.67
124.65
123.28
123.21
117.11
116.96
115.67
77.21
77.00
76.79
14.64
14.60
F
O
S
CN
41
210 200 190 180 170 160 150 140 130 120 110 100 90 80 70 60 50 40 30 20 10
f1 (ppm)

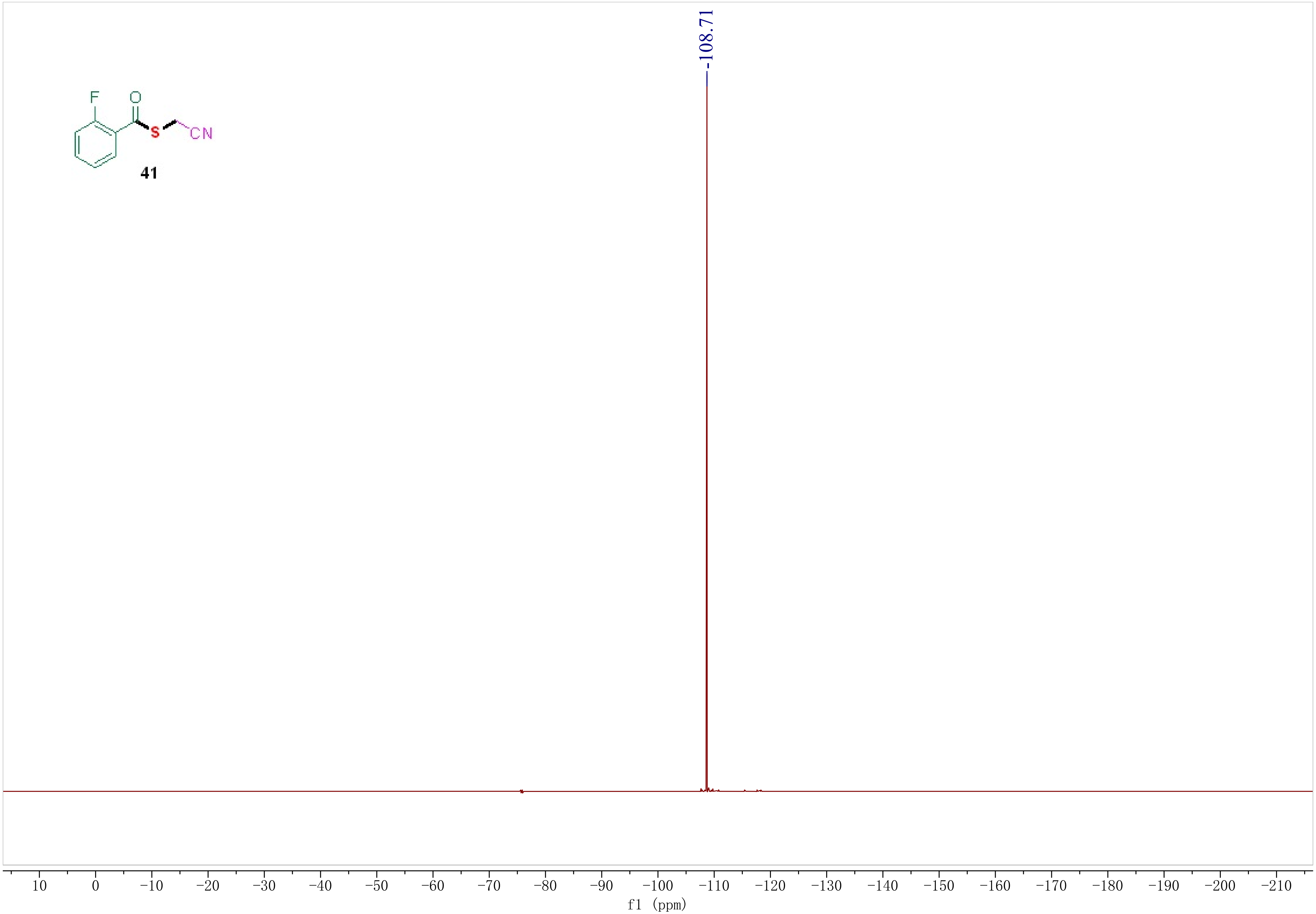

F
O
S
CN
41
-108.71
10
0
−10
−20
−30
−40
−50
−60
−70
−80
−90
−100
−110
−120
−130
−140
−150
−160
−170
−180
−190
−200
−210
f1 (ppm)

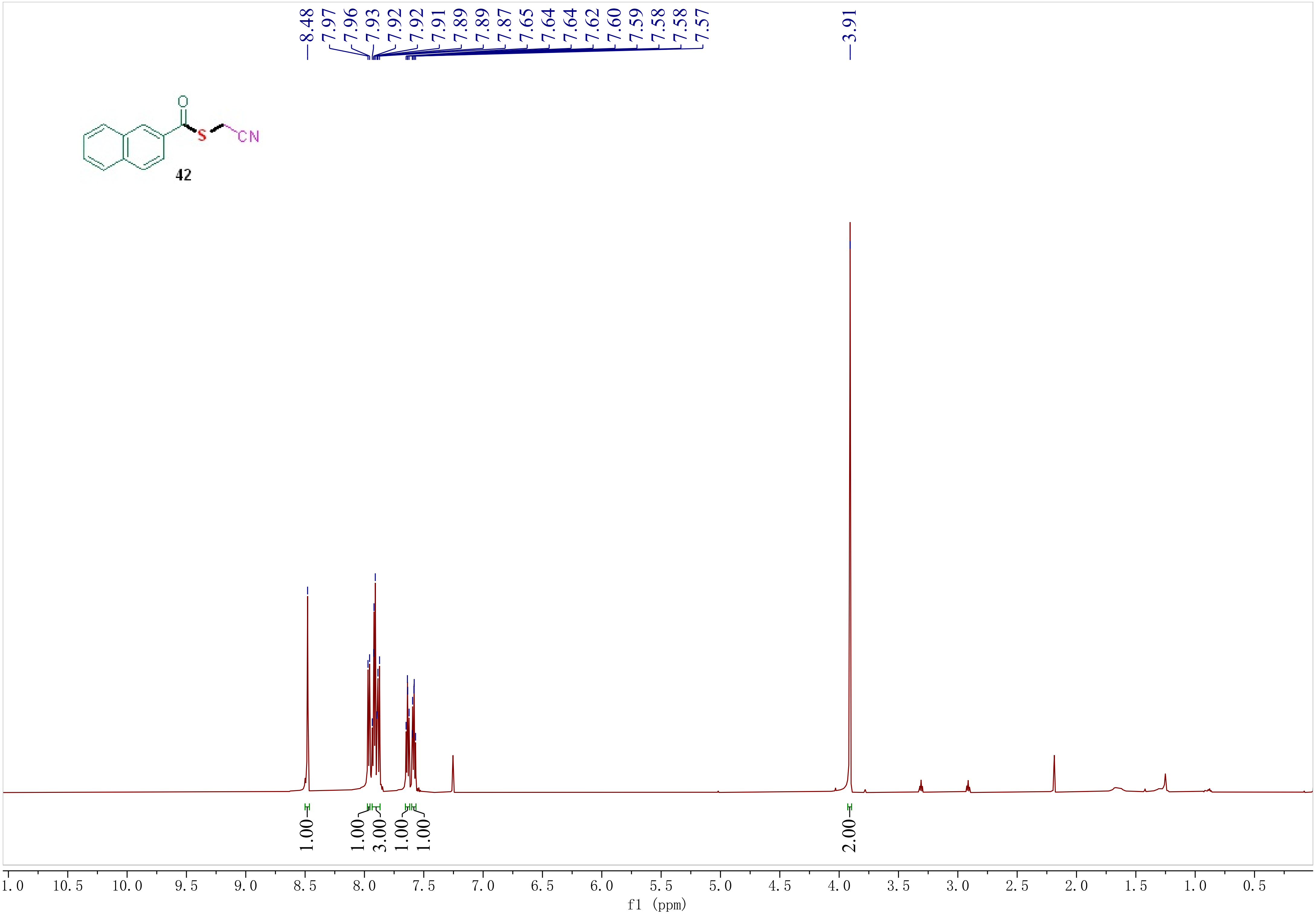

8.48
7.97 7.96 7.93 7.92 7.92 7.91 7.89 7.89 7.87 7.65 7.64 7.64 7.62 7.60 7.59 7.58 7.58 7.57
3.91
O
S
CN
42
1.00
1.00
3.00
1.00
1.00
2.00
1.0 10.5 10.0 9.5 9.0 8.5 8.0 7.5 7.0 6.5 6.0 5.5 5.0 4.5 4.0 3.5 3.0 2.5 2.0 1.5 1.0 0.5
f1 (ppm)

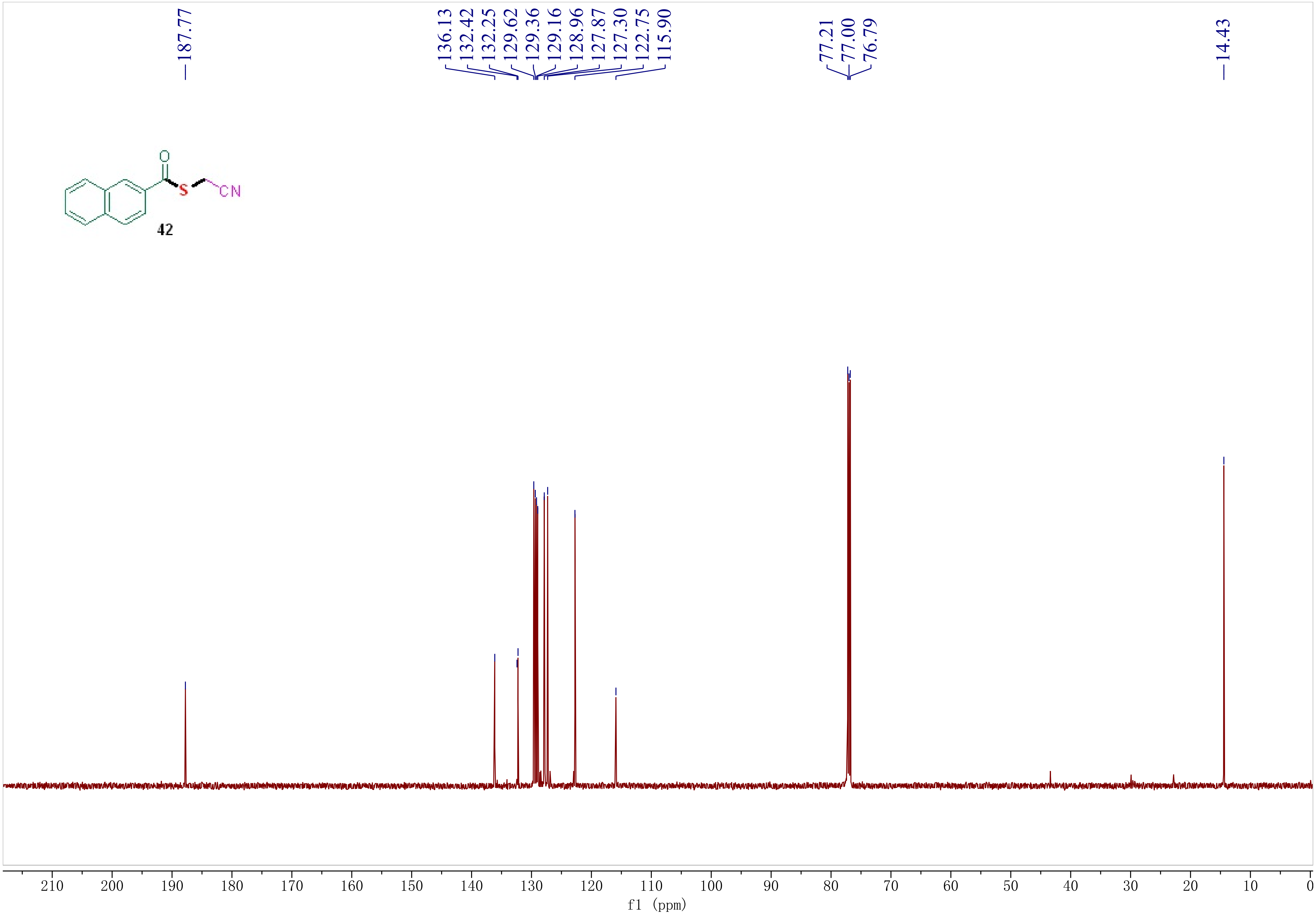

187.77
136.13
132.42
132.25
129.62
129.36
129.16
128.96
127.87
127.30
122.75
115.90
77.21
77.00
76.79
14.43
O
S
CN
42
210 200 190 180 170 160 150 140 130 120 110 100 90 80 70 60 50 40 30 20 10 0
f1 (ppm)

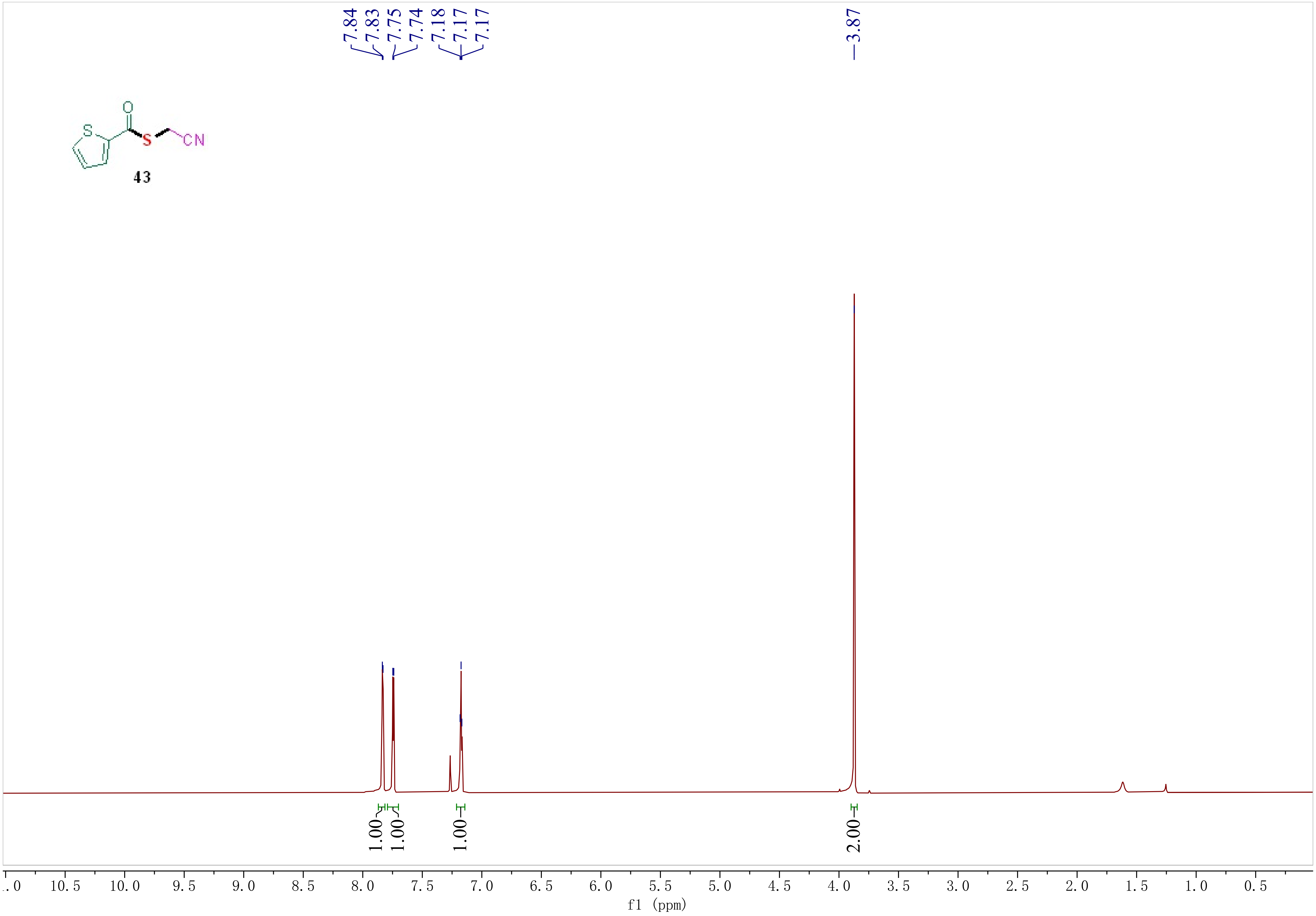

7.84
7.83
7.75
7.74
7.18
7.17
7.17
3.87
O
S
S
CN
43
1.00
1.00
1.00
2.00
11.0 10.5 10.0 9.5 9.0 8.5 8.0 7.5 7.0 6.5 6.0 5.5 5.0 4.5 4.0 3.5 3.0 2.5 2.0 1.5 1.0 0.5
f1 (ppm)

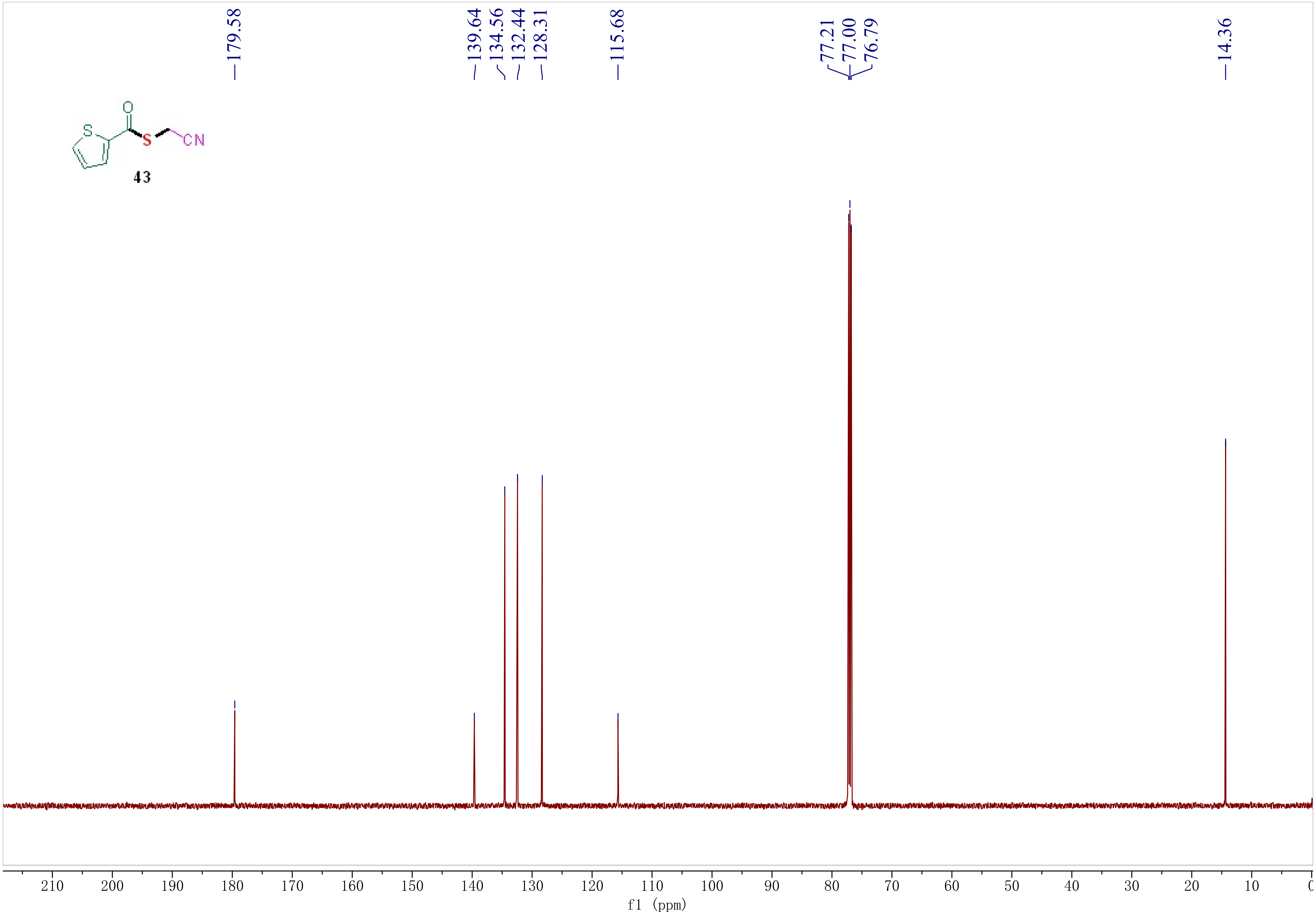

179.58
139.64
134.56
132.44
128.31
115.68
77.21
77.00
76.79
14.36
O
S
S
CN
43
210
200
190
180
170
160
150
140
130
120
110
100
90
80
70
60
50
40
30
20
10
f1 (ppm)

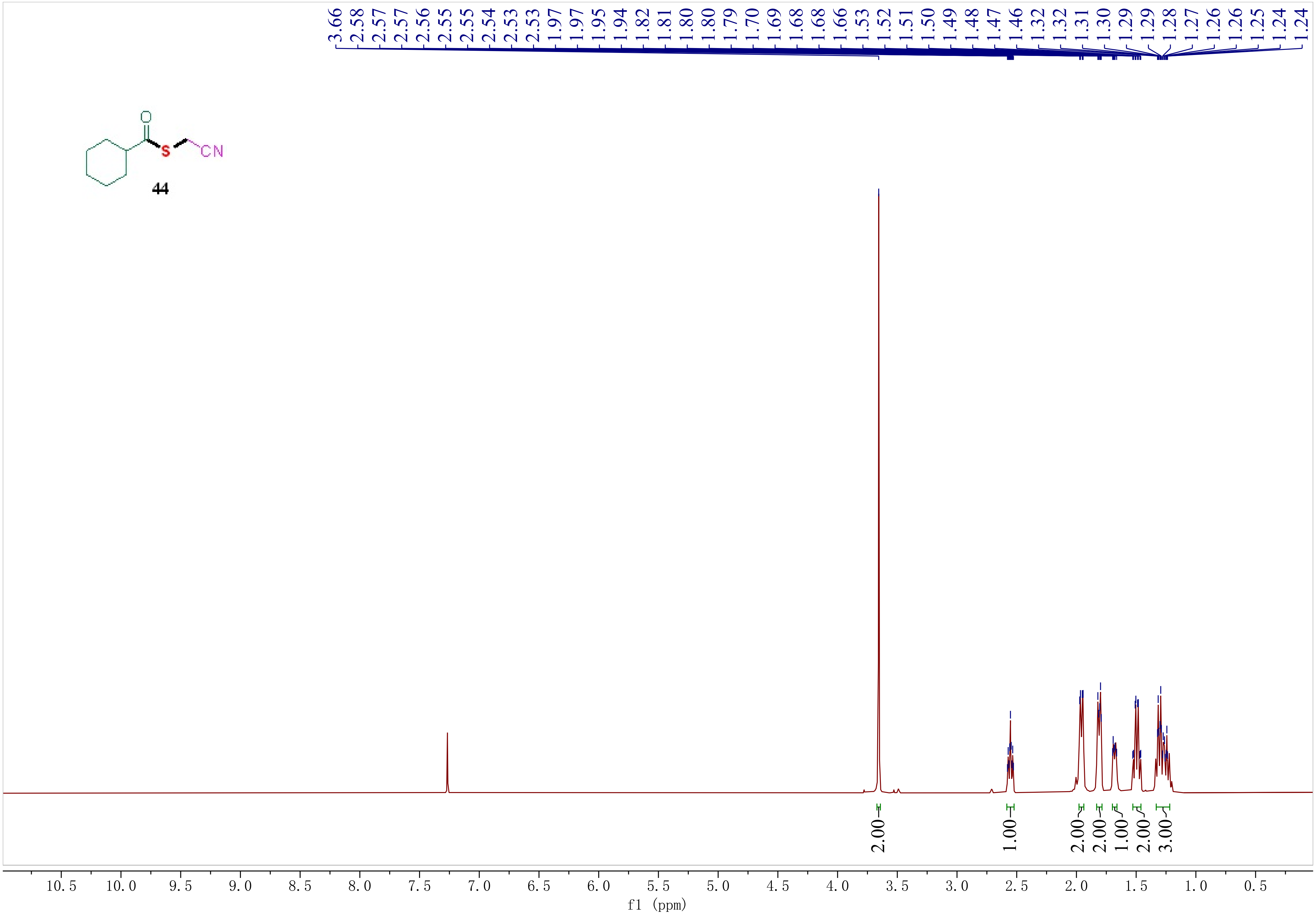

3.66 2.58 2.57 2.57 2.56 2.55 2.55 2.54 2.53 2.53 1.97 1.97 1.95 1.94 1.82 1.81 1.80 1.80 1.79 1.70 1.69 1.68 1.68 1.66 1.53 1.52 1.51 1.50 1.49 1.48 1.47 1.46 1.32 1.32 1.31 1.30 1.29 1.29 1.28 1.27 1.26 1.26 1.25 1.24 1.24
O
S
CN
44
2.00
1.00
2.00
2.00
1.00
2.00
3.00
10.5 10.0 9.5 9.0 8.5 8.0 7.5 7.0 6.5 6.0 5.5 5.0 4.5 4.0 3.5 3.0 2.5 2.0 1.5 1.0 0.5
f1 (ppm)

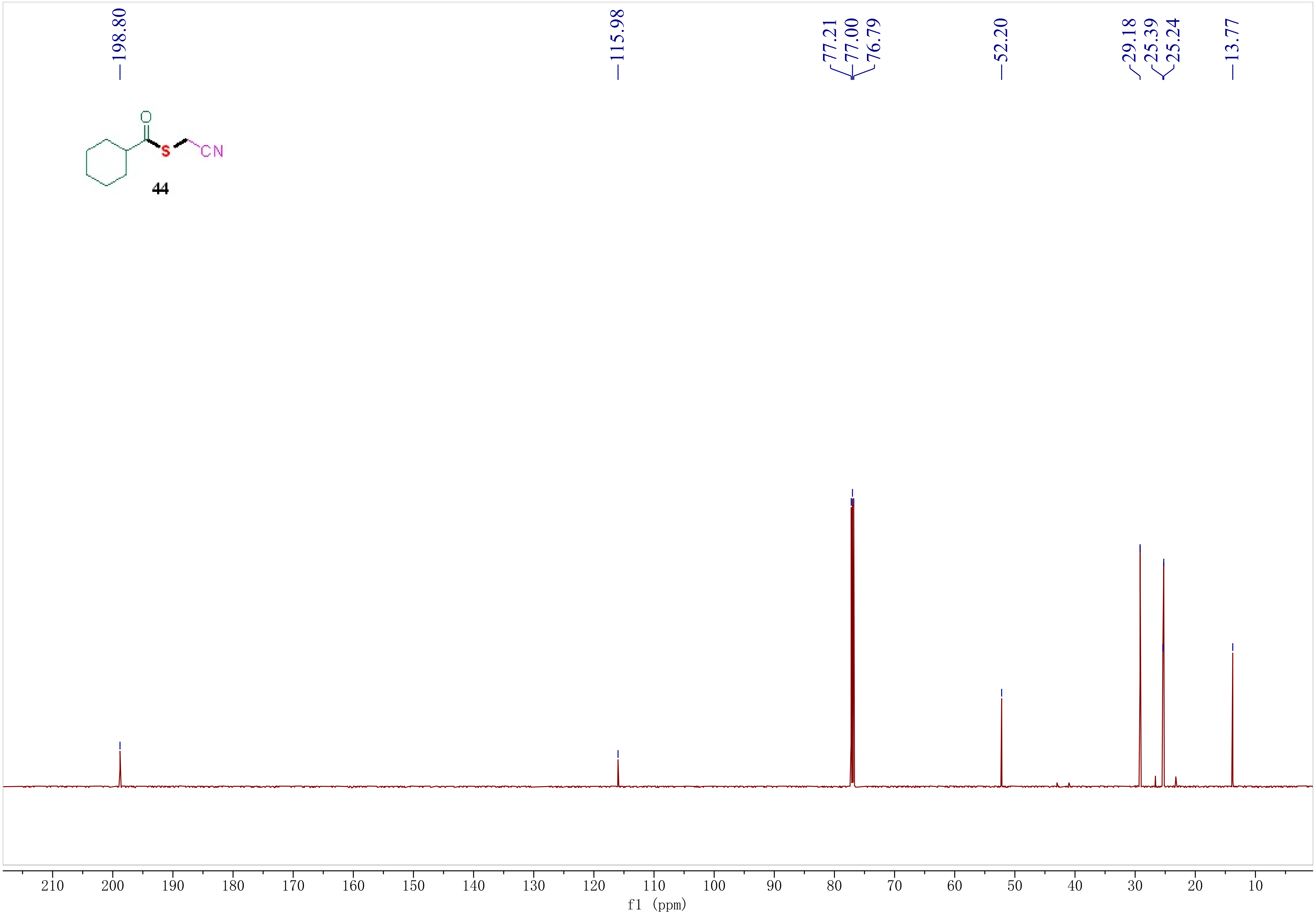
198.80
115.98
77.21
77.00
76.79
52.20
29.18
25.39
25.24
13.77
O
S
CN
44
210 200 190 180 170 160 150 140 130 120 110 100 90 80 70 60 50 40 30 20 10
f1 (ppm)

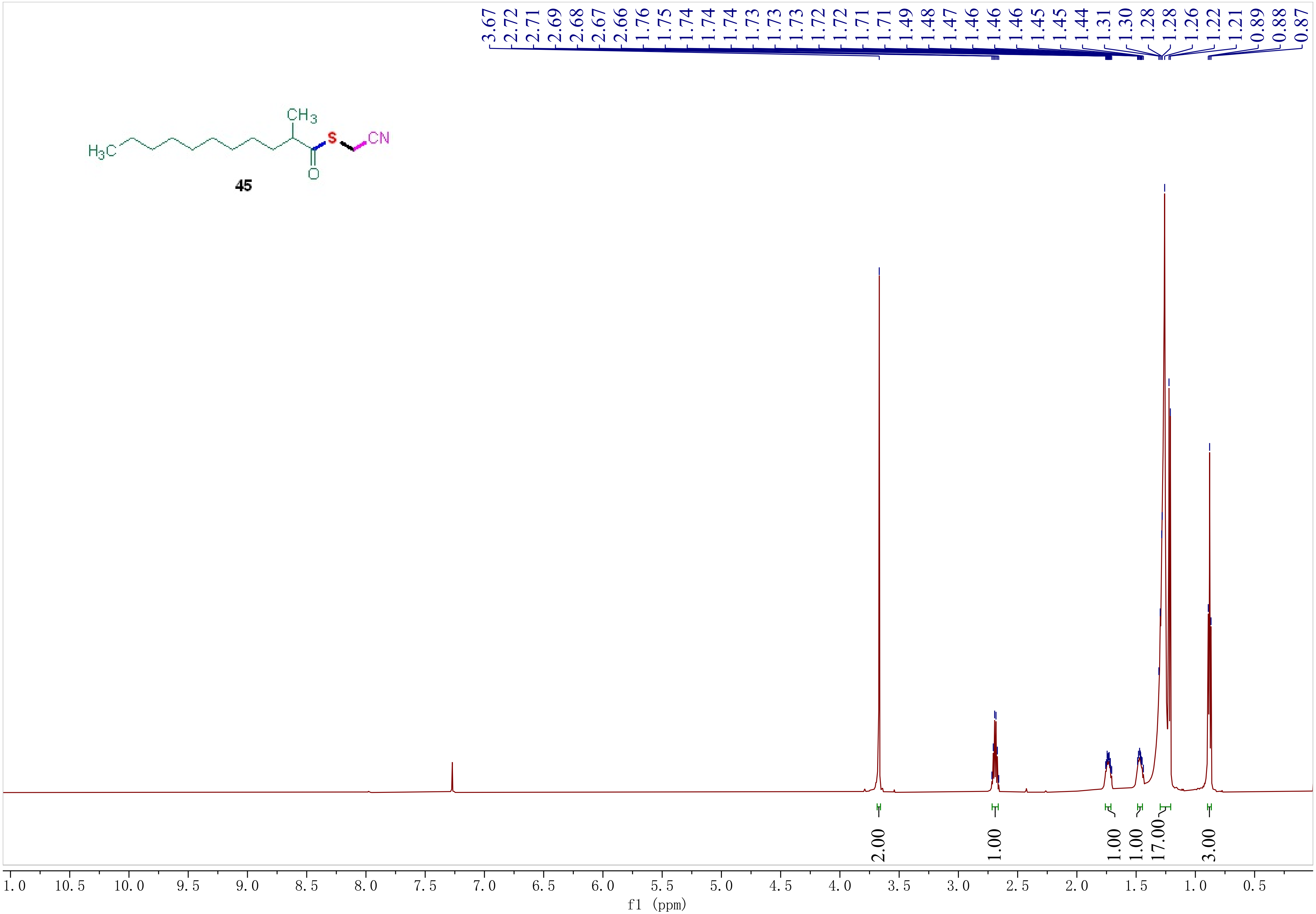
3.67 2.72 2.71 2.69 2.68 2.67 2.66 1.76 1.75 1.74 1.74 1.74 1.73 1.73 1.73 1.72 1.72 1.71 1.71 1.49 1.48 1.47 1.46 1.46 1.46 1.45 1.45 1.44 1.31 1.30 1.28 1.28 1.26 1.22 1.21 0.89 0.88 0.87
CH3
H3C
S
CN
O
45
2.00
1.00
1.00
1.00
17.00
3.00
1.0 10.5 10.0 9.5 9.0 8.5 8.0 7.5 7.0 6.5 6.0 5.5 5.0 4.5 4.0 3.5 3.0 2.5 2.0 1.5 1.0 0.5
f1 (ppm)

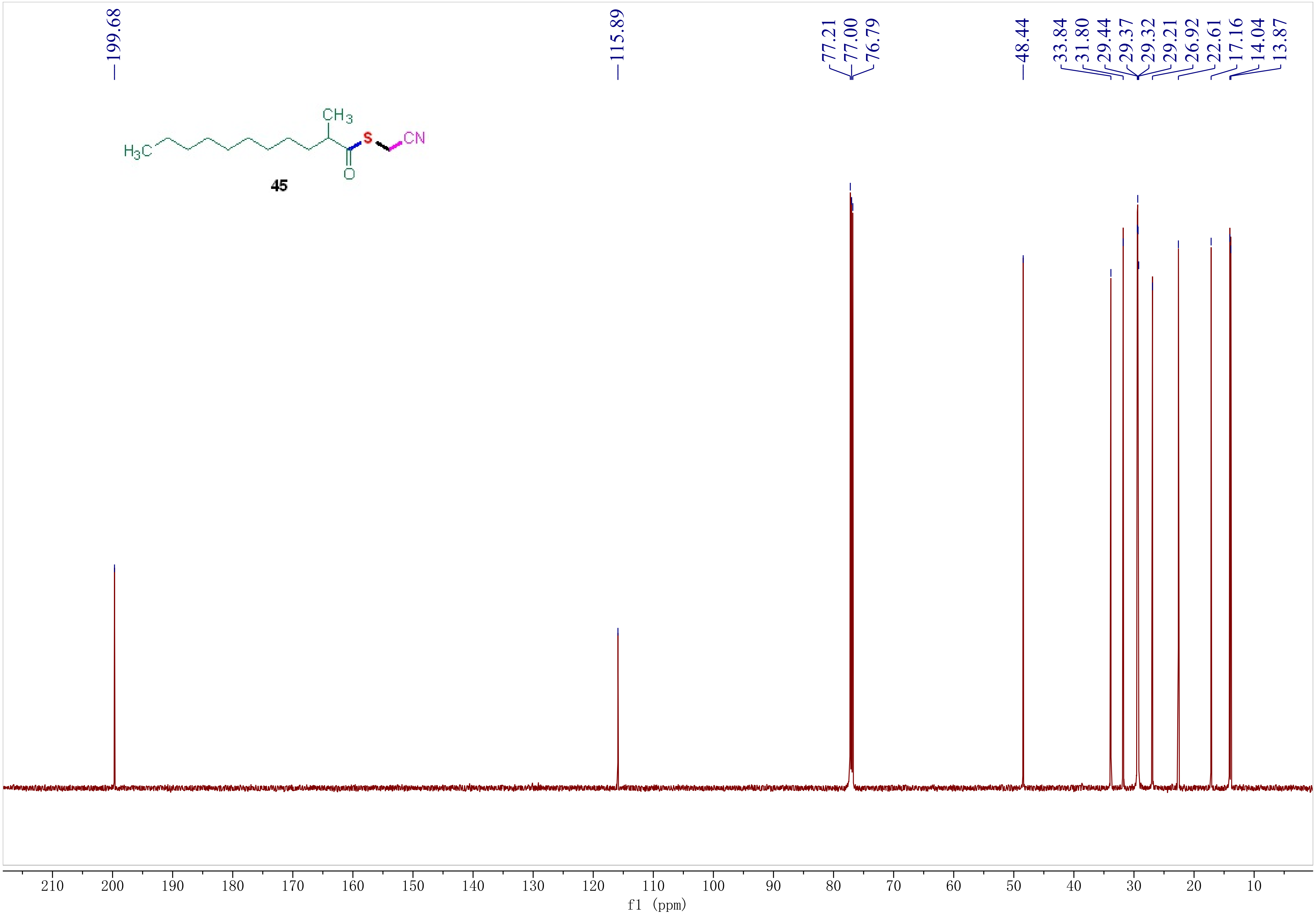

199.68
115.89
77.21
77.00
76.79
48.44
33.84
31.80
29.44
29.37
29.32
29.21
26.92
22.61
17.16
14.04
13.87
CH3
H3C
S
CN
O
45
210 200 190 180 170 160 150 140 130 120 110 100 90 80 70 60 50 40 30 20 10
f1 (ppm)

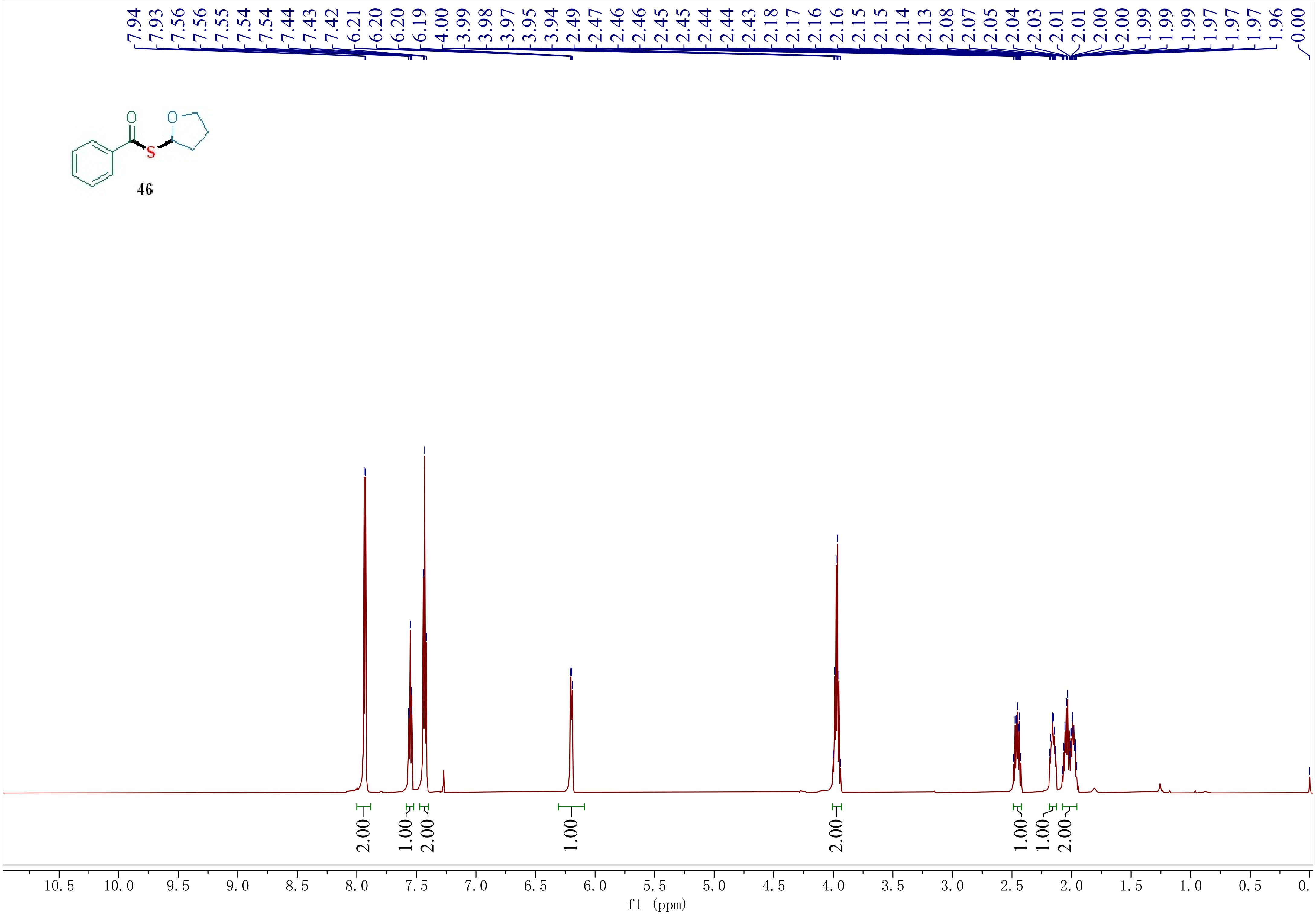
7.94 7.93 7.56 7.56 7.55 7.54 7.54 7.44 7.43 7.42 6.21 6.20 6.20 6.19 4.00 3.99 3.98 3.97 3.95 3.94 2.49 2.47 2.46 2.46 2.45 2.45 2.44 2.44 2.43 2.18 2.17 2.16 2.16 2.15 2.15 2.14 2.13 2.08 2.07 2.05 2.04 2.03 2.01 2.01 2.00 2.00 1.99 1.99 1.99 1.97 1.97 1.97 1.96 0.00
O
S
O
46
2.00
1.00
2.00
1.00
2.00
1.00
1.00
2.00
10.5 10.0 9.5 9.0 8.5 8.0 7.5 7.0 6.5 6.0 5.5 5.0 4.5 4.0 3.5 3.0 2.5 2.0 1.5 1.0 0.5 0.
f1 (ppm)

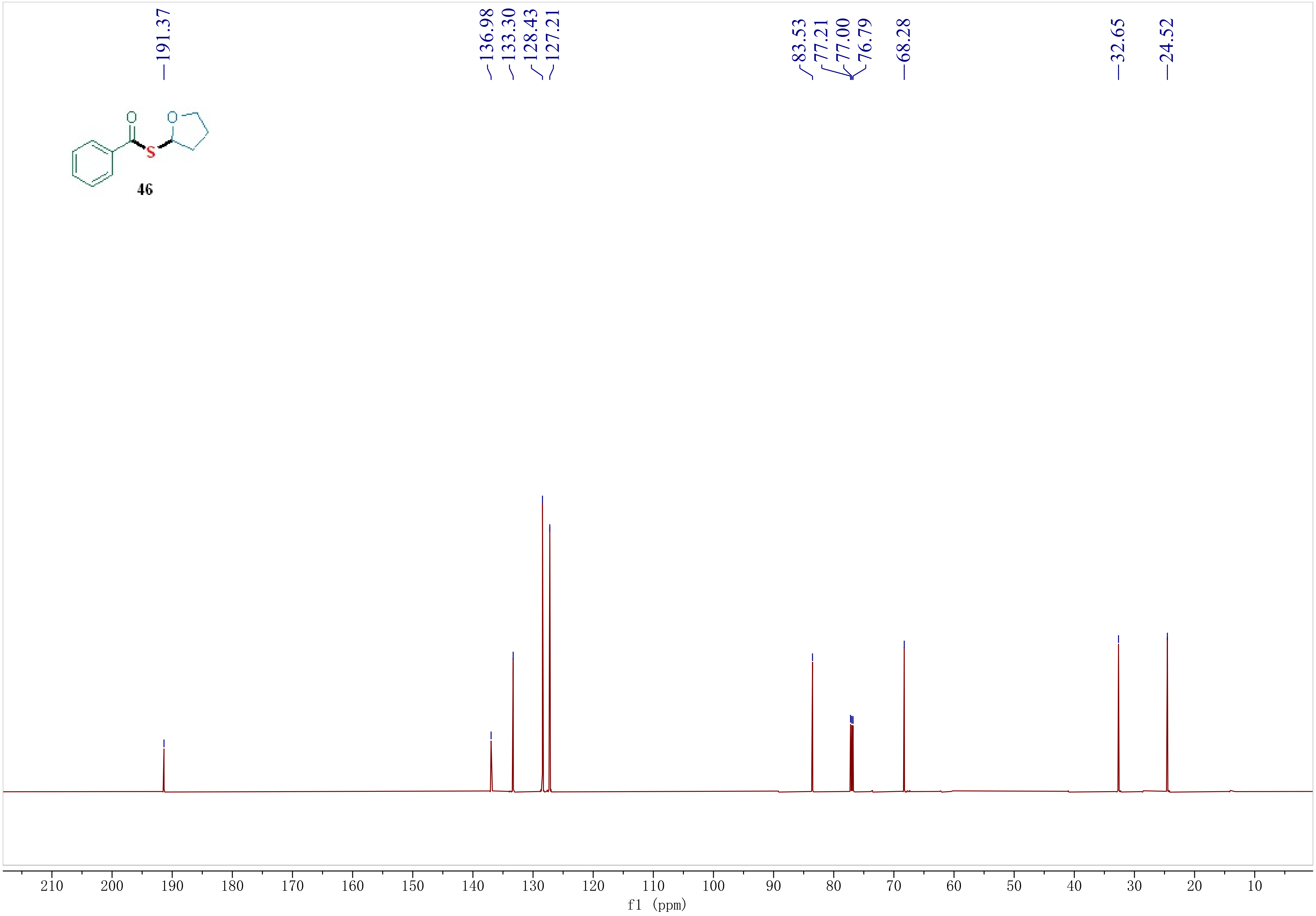

191.37
136.98
133.30
128.43
127.21
83.53
77.21
77.00
76.79
68.28
32.65
24.52
O
O
S
46
210
200
190
180
170
160
150
140
130
120
110
100
90
80
70
60
50
40
30
20
10
f1 (ppm)

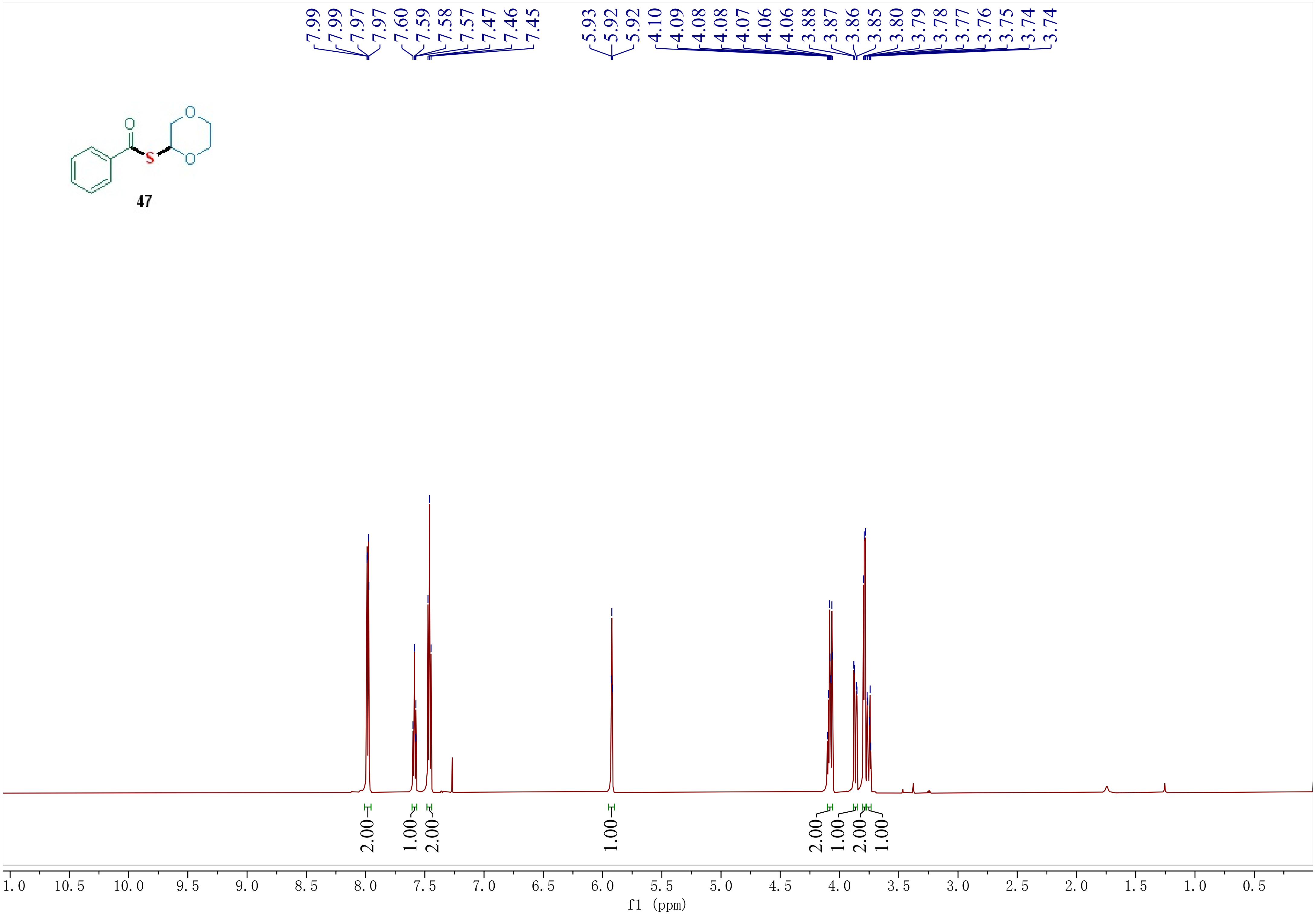
7.99
7.99
7.97
7.97
7.60
7.59
7.58
7.57
7.47
7.46
7.45
5.93
5.92
5.92
4.10
4.09
4.08
4.08
4.07
4.06
4.06
3.88
3.87
3.86
3.85
3.80
3.79
3.78
3.77
3.76
3.75
3.74
3.74
O
S
O
O
47
2.00
1.00
2.00
1.00
2.00
1.00
2.00
1.00
1.0
10.5
10.0
9.5
9.0
8.5
8.0
7.5
7.0
6.5
6.0
5.5
5.0
4.5
4.0
3.5
3.0
2.5
2.0
1.5
1.0
0.5
f1 (ppm)

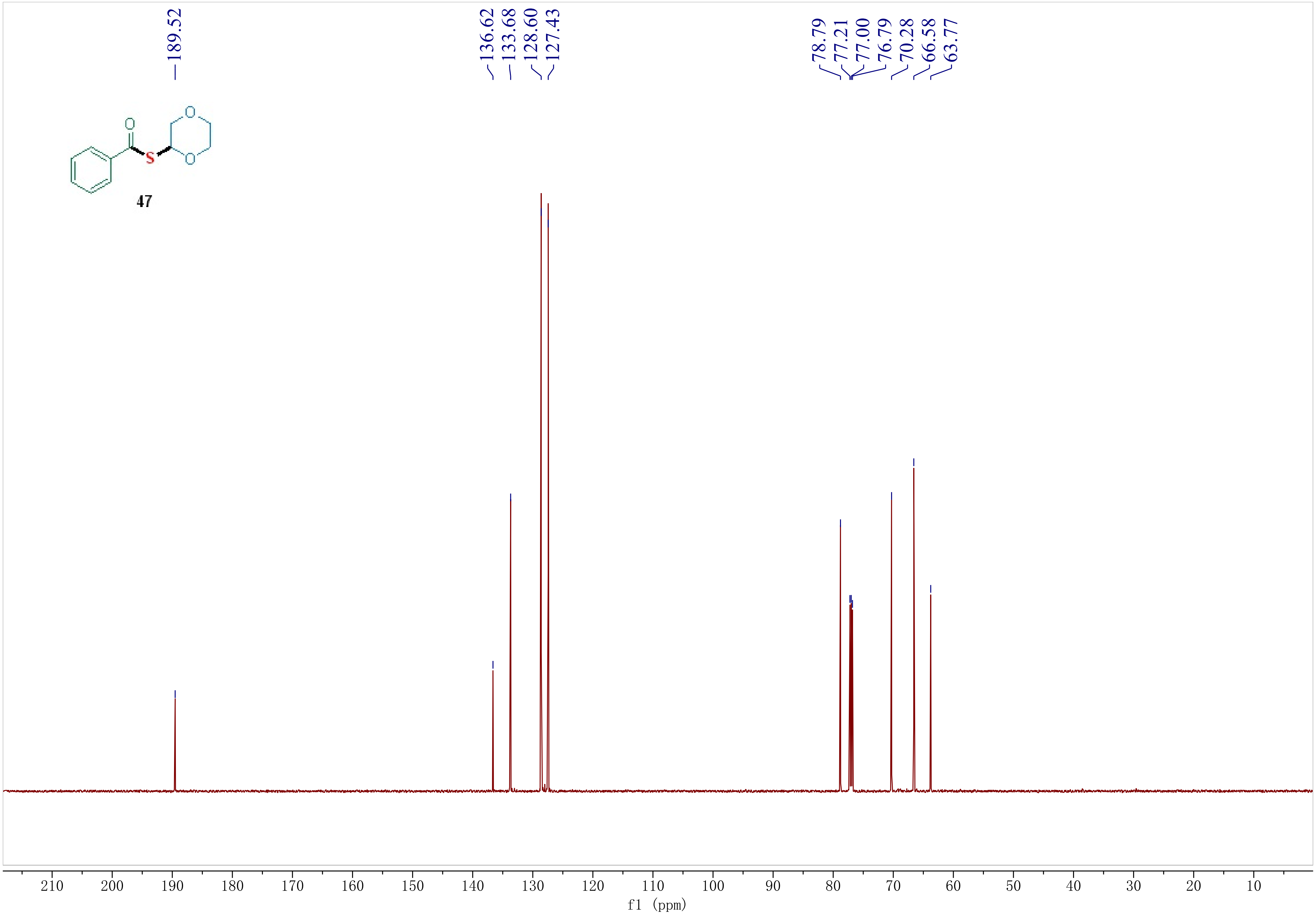
189.52
136.62
133.68
128.60
127.43
78.79
77.21
77.00
76.79
70.28
66.58
63.77
O
S
O
O
47
210
200
190
180
170
160
150
140
130
120
110
100
90
80
70
60
50
40
30
20
10
f1 (ppm)

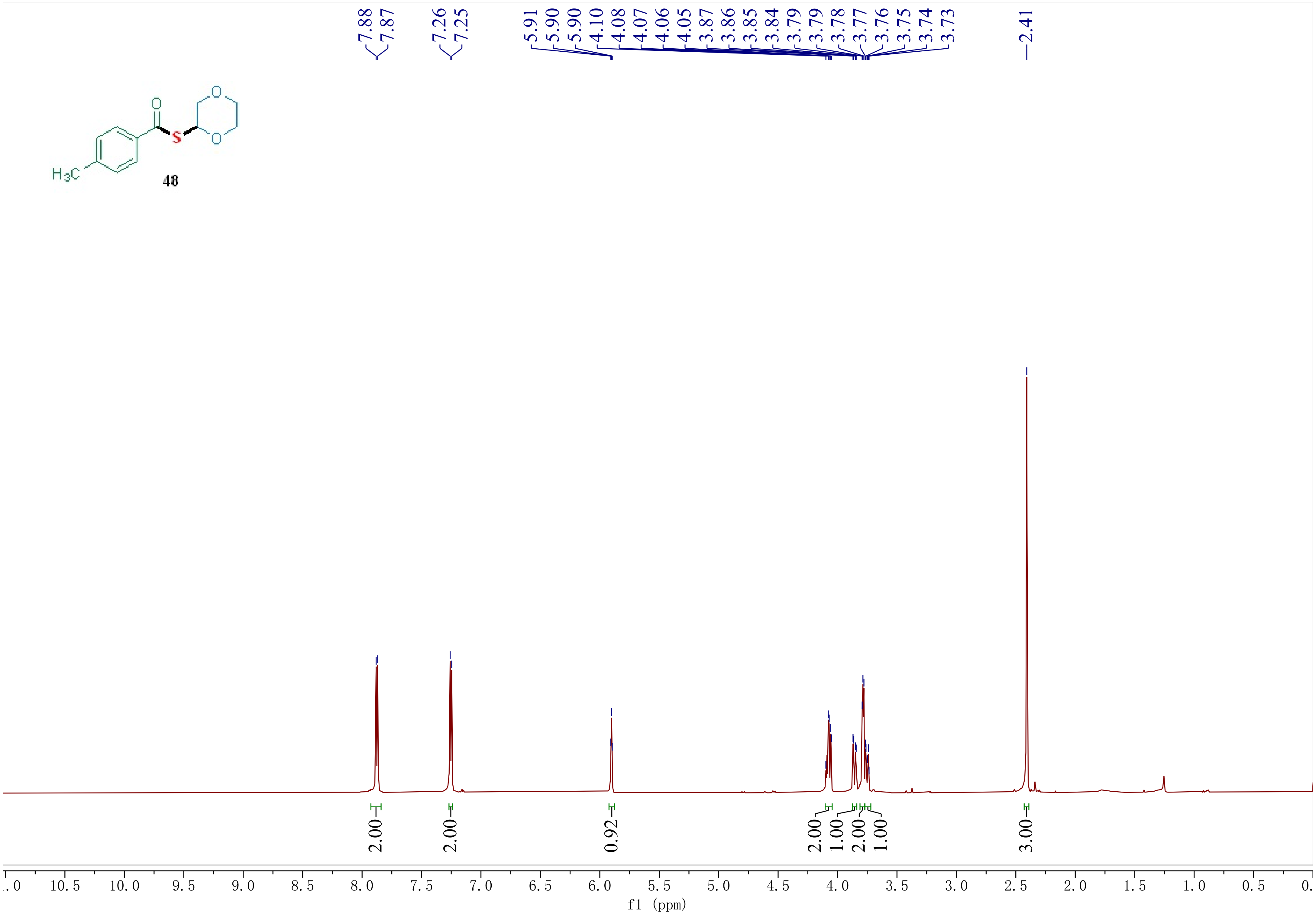
7.88
7.87
7.26
7.25
5.91
5.90
5.90
4.10
4.08
4.07
4.06
4.05
3.87
3.86
3.85
3.84
3.79
3.79
3.78
3.77
3.76
3.75
3.74
3.73
2.41
48
2.00
2.00
0.92
2.00
1.00
2.00
1.00
3.00
10.5
10.0
9.5
9.0
8.5
8.0
7.5
7.0
6.5
6.0
5.5
5.0
4.5
4.0
3.5
3.0
2.5
2.0
1.5
1.0
0.5
f1 (ppm)

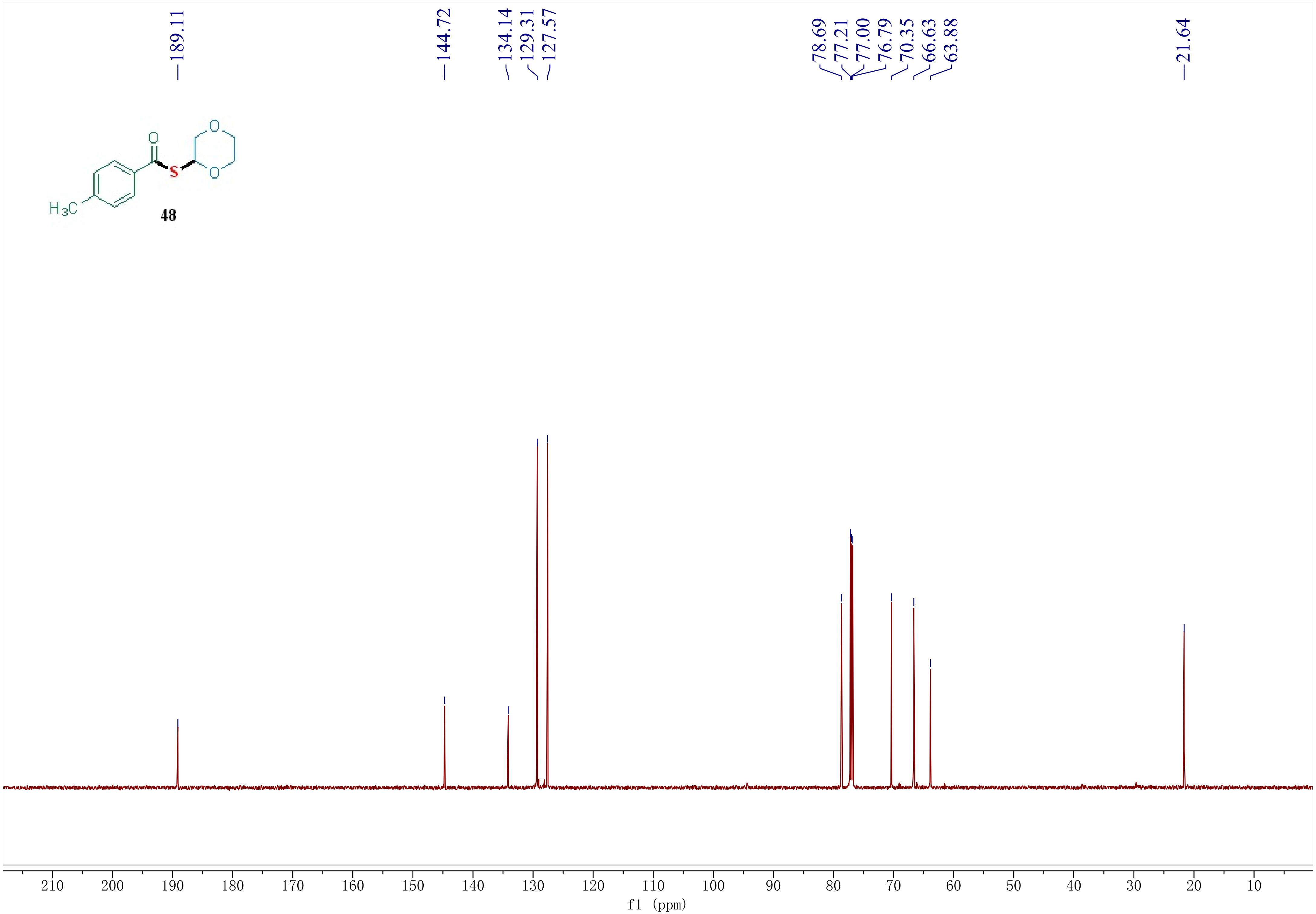
189.11
144.72
134.14
129.31
127.57
78.69
77.21
77.00
76.79
70.35
66.63
63.88
21.64
O
S
O
O
H3C
48
210
200
190
180
170
160
150
140
130
120
110
100
90
80
70
60
50
40
30
20
10
f1 (ppm)
